# Open Source Drug Discovery with the Malaria Box Compound Collection for Neglected Diseases and Beyond

**DOI:** 10.1371/journal.ppat.1005763

**Published:** 2016-07-28

**Authors:** Wesley C. Van Voorhis, John H. Adams, Roberto Adelfio, Vida Ahyong, Myles H. Akabas, Pietro Alano, Aintzane Alday, Yesmalie Alemán Resto, Aishah Alsibaee, Ainhoa Alzualde, Katherine T. Andrews, Simon V. Avery, Vicky M. Avery, Lawrence Ayong, Mark Baker, Stephen Baker, Choukri Ben Mamoun, Sangeeta Bhatia, Quentin Bickle, Lotfi Bounaadja, Tana Bowling, Jürgen Bosch, Lauren E. Boucher, Fabrice F. Boyom, Jose Brea, Marian Brennan, Audrey Burton, Conor R. Caffrey, Grazia Camarda, Manuela Carrasquilla, Dee Carter, Maria Belen Cassera, Ken Chih-Chien Cheng, Worathad Chindaudomsate, Anthony Chubb, Beatrice L. Colon, Daisy D. Colón-López, Yolanda Corbett, Gregory J. Crowther, Noemi Cowan, Sarah D’Alessandro, Na Le Dang, Michael Delves, Joseph L. DeRisi, Alan Y. Du, Sandra Duffy, Shimaa Abd El-Salam El-Sayed, Michael T. Ferdig, José A. Fernández Robledo, David A. Fidock, Isabelle Florent, Patrick V. T. Fokou, Ani Galstian, Francisco Javier Gamo, Suzanne Gokool, Ben Gold, Todd Golub, Gregory M. Goldgof, Rajarshi Guha, W. Armand Guiguemde, Nil Gural, R. Kiplin Guy, Michael A. E. Hansen, Kirsten K. Hanson, Andrew Hemphill, Rob Hooft van Huijsduijnen, Takaaki Horii, Paul Horrocks, Tyler B. Hughes, Christopher Huston, Ikuo Igarashi, Katrin Ingram-Sieber, Maurice A. Itoe, Ajit Jadhav, Amornrat Naranuntarat Jensen, Laran T. Jensen, Rays H. Y. Jiang, Annette Kaiser, Jennifer Keiser, Thomas Ketas, Sebastien Kicka, Sunyoung Kim, Kiaran Kirk, Vidya P. Kumar, Dennis E. Kyle, Maria Jose Lafuente, Scott Landfear, Nathan Lee, Sukjun Lee, Adele M. Lehane, Fengwu Li, David Little, Liqiong Liu, Manuel Llinás, Maria I. Loza, Aristea Lubar, Leonardo Lucantoni, Isabelle Lucet, Louis Maes, Dalu Mancama, Nuha R. Mansour, Sandra March, Sheena McGowan, Iset Medina Vera, Stephan Meister, Luke Mercer, Jordi Mestres, Alvine N. Mfopa, Raj N. Misra, Seunghyun Moon, John P. Moore, Francielly Morais Rodrigues da Costa, Joachim Müller, Arantza Muriana, Stephen Nakazawa Hewitt, Bakela Nare, Carl Nathan, Nathalie Narraidoo, Sujeevi Nawaratna, Kayode K. Ojo, Diana Ortiz, Gordana Panic, George Papadatos, Silvia Parapini, Kailash Patra, Ngoc Pham, Sarah Prats, David M. Plouffe, Sally-Ann Poulsen, Anupam Pradhan, Celia Quevedo, Ronald J. Quinn, Christopher A. Rice, Mohamed Abdo Rizk, Andrea Ruecker, Robert St. Onge, Rafaela Salgado Ferreira, Jasmeet Samra, Natalie G. Robinett, Ulrich Schlecht, Marjorie Schmitt, Filipe Silva Villela, Francesco Silvestrini, Robert Sinden, Dennis A. Smith, Thierry Soldati, Andreas Spitzmüller, Serge Maximilian Stamm, David J. Sullivan, William Sullivan, Sundari Suresh, Brian M. Suzuki, Yo Suzuki, S. Joshua Swamidass, Donatella Taramelli, Lauve R. Y. Tchokouaha, Anjo Theron, David Thomas, Kathryn F. Tonissen, Simon Townson, Abhai K. Tripathi, Valentin Trofimov, Kenneth O. Udenze, Imran Ullah, Cindy Vallieres, Edgar Vigil, Joseph M. Vinetz, Phat Voong Vinh, Hoan Vu, Nao-aki Watanabe, Kate Weatherby, Pamela M. White, Andrew F. Wilks, Elizabeth A. Winzeler, Edward Wojcik, Melanie Wree, Wesley Wu, Naoaki Yokoyama, Paul H. A. Zollo, Nada Abla, Benjamin Blasco, Jeremy Burrows, Benoît Laleu, Didier Leroy, Thomas Spangenberg, Timothy Wells, Paul A. Willis

**Affiliations:** 1 Departments of Medicine, Microbiology, and Global Health, Center for Emerging and Re-emerging Infectious Diseases (CERID) University of Washington, Seattle, Washington, United States of America; 2 Center for Global Health and Infectious Diseases Research, Department of Global Health, University of South Florida, Tampa, Florida, United States of America; 3 Medical Parasitology and Infection Biology, Swiss Tropical and Public Health Institute, Basel, Switzerland; 4 University of Basel, Basel, Switzerland; 5 Howard Hughes Medical Institute, Department of Biochemistry and Biophysics, University of California, San Francisco, California, United States of America; 6 Departments of Physiology & Biophysics, Neuroscience and Medicine, Albert Einstein College of Medicine, New York, New York, United States of America; 7 Dipartimento Malattie Infettive, Parassitarie ed Immunomediate Istituto Superiore di Sanità, Roma, Italia; 8 BBD BioPhenix SL–BIOBIDE, Donostia, Gipuzkoa, Spain; 9 Bigelow Laboratory for Ocean Sciences, East Boothbay, Maine, United States of America; 10 Molecular and Cellular Therapeutics, Royal College of Surgeons in Ireland, Dublin, Ireland; 11 Eskitis Institute for Drug Discovery, Griffith University, Nathan, QLD, Australia; 12 QIMR Berghofer Medical Research Institute Herston, Brisbane, Australia; 13 School of Life Sciences, University of Nottingham, Nottingham, Nottinghamshire, England, United Kingdom; 14 Institut Pasteur Korea, Pangyo Techno-Valley, Gyeonggi Province, Korea; 15 Clinical Pharmacology, Novartis Consumer Health, Nyon, Switzerland; 16 Oxford University Clinical Research Unit, Wellcome Trust Major Overseas Programme, The Hospital for Tropical Diseases, Ho Chi Minh City, Vietnam; 17 Nuffield Department of Clinical Medicine, Centre for Tropical Medicine, Oxford University, Oxford, England, United Kingdom; 18 The London School of Hygiene and Tropical Medicine, London, England, United Kingdom; 19 Internal Medicine, Yale University, New Haven, Connecticut, United States of America; 20 Health Sciences and Technology/Institute for Medical Engineering and Science, Massachusetts Institute of Technology, Cambridge, Massachusetts, United States of America; 21 Department of Immunology & Infection, London School of Hygiene and Tropical Medicine, London, England, United Kingdom; 22 Museum of National History, Sorbonne Universities, Paris, France; 23 SCYNEXIS, Inc., Durham, North Carolina, United States of America; 24 Department of Biochemistry and Molecular Biology and Johns Hopkins Malaria Research Institute, Johns Hopkins Bloomberg School of Public Health, Baltimore, Maryland, Untied States of America; 25 Department of Biochemistry, University of Yaoundé, Yaoundé, Cameroon; 26 CIMUS Research Centre, University of Santiago de Compostela, Santiago de Compostela, A Coruña, Spain; 27 Center for Discovery and Innovation in Parasitic Diseases, Department of Pathology, University of California San Francisco, San Francisco, California, United States of America; 28 Department of Biochemistry and Molecular Biology and Huck Center for Malaria Research, Pennsylvania State University, University Park, Pennsylvania, United States of America; 29 School of Life and Environmental Sciences, University of Sydney, Darlington New South Wales, Australia; 30 Department of Biochemistry and Virginia Tech Center for Drug Discovery, Virginia Polytechnic Institute and State University, Blacksburg, Virginia, United States of America; 31 National Center of Advancing Translational Sciences, NIH, Bethesda, Maryland, United States of America; 32 Department of Biochemistry, Mahidol University, Bangkok, Thailand; 33 Department of Molecular Medicine, Morsani College of Medicine, University of South Florida, Tampa, Florida, United States of America; 34 Department of Pharmacological and Biomolecular Sciences, Università degli Studi di Milano, Milano, Italy; 35 Department of Pathology and Immunology, Washington University in St Louis, St. Louis, Missouri, United States of America; 36 Department of Life Sciences, Imperial College London, London, England, United Kingdom; 37 Division of Pharmacology and Drug Discovery, Department of Pediatrics, School of Medicine University of California San Diego, La Jolla, California, United States of America; 38 National Research Center for Protozoan Diseases, Obihiro University of Agriculture and Veterinary Medicine, Obihiro, Hokkaido, Japan; 39 Department of Biochemistry and Chemistry of nutrition, Mansoura University, Mansoura City, Egypt; 40 Eck Institute for Global Health, Department of Biological Sciences, University of Notre Dame, Notre Dame Indiana, United States of America; 41 Department of Microbiology & Immunology and Division of Infectious Diseases, Department of Medicine, Columbia University Medical Center, New York, New York, United States of America; 42 Broad Institute, Cambridge, Massachusetts, United States of America; 43 Biochemistry and Parasitology Department, Malaria DPU, Diseases of the Developing World (DDW), GlaxoSmithKline R&D, Tres Cantos, Madrid, Spain; 44 Tropical Parasitic Diseases Unit, Northwick Park Institute for Medical Research, Harrow, Middlesex, England, United Kingdom; 45 Department of Microbiology and Immunology, Weill Cornell Medical College, New York, New York, United States of America; 46 Medical Scientist Training Program, University of California, San Diego, San Diego, California, United States of America; 47 Department of Chemical Biology & Therapeutics, St. Jude Children’s Research Hospital, Memphis, Tennessee, United States of America; 48 Dept. of Biology and South Texas Center for Emerging Infectious Diseases, University of Texas, San Antonio, San Antonio, Texas, United States of America; 49 Instituto de Medicina Molecular, Lisboa, Portugal; 50 Institute of Parasitology, University of Berne, Bern, Switzerland; 51 Medicines for Malaria Venture, Geneva, Switzerland; 52 Global Health Research Section, hhc Data Creation Center, Eisai Co., Ltd, Tsukuba-shi, Ibaraki, Japan; 53 Institute for Science and Technology in Medicine, Keele University, Keele, Staffordshire, United Kingdom; 54 Department of Medicine, College of Medicine, University of Vermont, Burlington, Vermont, United States of America; 55 Department of Pathobiology, Faculty of Science, Mahidol University, Bangkok Thailand; 56 Medical Research Centre, Institute for Pharmacogenetics, Essen, Germany; 57 Department of Biochemistry, University of Geneva, Geneva, Switzerland; 58 Department of Biochemistry and Molecular Biology, LSU Health Sciences Center, New Orleans, Louisiana, United States of America; 59 Research School of Biology, Australian National University, Canberra, Australian Capital Territory, Australia; 60 Department of Molecular Microbiology & Immunology, Oregon Health & Science University, Portland, Oregon, United States of America; 61 Department of Medicine, University of California San Diego, San Diego, California, United States of America; 62 Department of Biochemistry and Molecular Biology, Monash University, Clayton, Australia; 63 University of Antwerp, Department of Biomedical Sciences, Antwerp, Belgium; 64 Biosciences Unit, Council for Scientific and Industrial Research, Pretoria, South Africa; 65 Department of Microbiology, Monash University, Clayton, Australia; 66 Chemotargets S.L. and Research Group on Systems Pharmacology, Research Program on Biomedical Informatics (GRIB), IMIM Hospital del Mar Institute of Medical Research and University Pompeu Fabra, Barcelona, Catalonia, Spain; 67 Division of Cancer Therapeutics and Diagnosis, Drug Synthesis and Chemistry Branch, National Cancer Institute, National Institutes of Health, Bethesda, Maryland, United States of America; 68 Graduate Program in Bioinformatics, Universidade Federal de Minas Gerais, Belo Horizonte, Minas Gerais, Brazil; 69 ChEMBL group, European Molecular Biology Laboratory—European Bioinformatics Institute (EMBL-EBI), Hinxton, Cambridgeshire, United Kingdom; 70 Genomics Institute of the Novartis Research Foundation, San Diego, California, United States of America; 71 Department of Internal Medicine and Infectious Diseases, Faculty of Veterinary Medicine, Mansoura University, Mansoura City, Egypt; 72 Department of Biochemistry and Stanford Genome Technology Center, Stanford University, Palo Alto, Calilfornia, United States of America; 73 Departamento de Bioquímica e Imunologia, Instituto de Ciências Biológicas, Universidade Federal de Minas Gerais, Belo Horizonte, Minas Gerais, Brazil; 74 Laboratoire de Chimie Moléculaire, CNRS,—UMR 7509, COB-IRJBD, Mulhouse Cedex, France; 75 The Jenner Institute, University of Oxford, Oxford, England, United Kingdom; 76 Department of Chemistry, University of Capetown, Capetown, South Africa; 77 H. Feinstone Department of Molecular Microbiology and Immunology, Johns Hopkins School of Public Health, Baltimore, Maryland, United States of America; 78 Molecular, Cell and Developmental Biology, University of California, Santa Cruz, Santa Cruz, California, United States of America; 79 Department of Synthetic Biology and Bioenergy, J. Craig Venter Institute, La Jolla, California, United States of America; 80 School of Natural Sciences, Griffith University, Nathan, Queensland, Australia; 81 Hudson Institute of Medical Research; Walter and Eliza Hall Institute of Medical Research, Parkville, Victoria, Australia; 82 SYNthesis Research, Parkville, Victoria, Australia; U Tex SouthWestern, UNITED STATES

## Abstract

A major cause of the paucity of new starting points for drug discovery is the lack of interaction between academia and industry. Much of the global resource in biology is present in universities, whereas the focus of medicinal chemistry is still largely within industry. Open source drug discovery, with sharing of information, is clearly a first step towards overcoming this gap. But the interface could especially be bridged through a scale-up of open sharing of physical compounds, which would accelerate the finding of new starting points for drug discovery. The Medicines for Malaria Venture Malaria Box is a collection of over 400 compounds representing families of structures identified in phenotypic screens of pharmaceutical and academic libraries against the *Plasmodium falciparum* malaria parasite. The set has now been distributed to almost 200 research groups globally in the last two years, with the only stipulation that information from the screens is deposited in the public domain. This paper reports for the first time on 236 screens that have been carried out against the Malaria Box and compares these results with 55 assays that were previously published, in a format that allows a meta-analysis of the combined dataset. The combined biochemical and cellular assays presented here suggest mechanisms of action for 135 (34%) of the compounds active in killing multiple life-cycle stages of the malaria parasite, including asexual blood, liver, gametocyte, gametes and insect ookinete stages. In addition, many compounds demonstrated activity against other pathogens, showing hits in assays with 16 protozoa, 7 helminths, 9 bacterial and mycobacterial species, the dengue fever mosquito vector, and the NCI60 human cancer cell line panel of 60 human tumor cell lines. Toxicological, pharmacokinetic and metabolic properties were collected on all the compounds, assisting in the selection of the most promising candidates for murine proof-of-concept experiments and medicinal chemistry programs. The data for all of these assays are presented and analyzed to show how outstanding leads for many indications can be selected. These results reveal the immense potential for translating the dispersed expertise in biological assays involving human pathogens into drug discovery starting points, by providing open access to new families of molecules, and emphasize how a small additional investment made to help acquire and distribute compounds, and sharing the data, can catalyze drug discovery for dozens of different indications. Another lesson is that when multiple screens from different groups are run on the same library, results can be integrated quickly to select the most valuable starting points for subsequent medicinal chemistry efforts.

## Author Summary

Malaria leads to the loss of over 440,000 lives annually; accelerating research to discover new candidate drugs is a priority. Medicines for Malaria Venture (MMV) has distilled over 25,000 compounds that kill malaria parasites *in vitro* into a group of 400 representative compounds, called the "Malaria Box". These Malaria Box sets were distributed free-of-charge to research laboratories in 30 different countries that work on a wide variety of pathogens. Fifty-five groups compiled >290 assay results for this paper describing the many activities of the Malaria Box compounds. The collective results suggest a potential mechanism of action for over 130 compounds against malaria and illuminate the most promising compounds for further malaria drug development research. Excitingly some of these compounds also showed outstanding activity against other disease agents including fungi, bacteria, other single-cellular parasites, worms, and even human cancer cells. The results have ignited over 30 drug development programs for a variety of diseases. This open access effort was so successful that MMV has begun to distribute another set of compounds with initial activity against a wider range of infectious agents that are of public health concern, called the Pathogen Box, available now to scientific labs all over the world (www.PathogenBox.org).

## Introduction

Preclinical development for drugs in neglected diseases remains a slow process due to a lack of access to compounds, and legal complications over intellectual property ownership. One way to accelerate drug discovery is to provide open access to bioactive molecules with public disclosure of the resulting biological data. The data from open access of bioactive molecules can help prioritize which compounds to investigate further through medicinal chemistry for the original indication and can also uncover other indications for compound development. It was in this spirit of providing open access of malaria-bioactive compounds, and disseminating the results in the public domain, that the Malaria Box project was initiated by the Medicines for Malaria Venture.

### Origins of the ‘Malaria Box’ compound set

Since 2007, over 6 million compounds were screened against asexual-stage *Plasmodium falciparum*, at two pharmaceutical companies (GlaxoSmithKline [1] and Novartis [2]), and two academic centers (St. Jude, Memphis [3], and Eskitis, Australia [4]), resulting in over 20,000 compounds active in the low- to sub-micromolar range. The structures of the 20,000 anti-malaria hits were made available in ChEMBL (www.ebi.ac.uk/chembl), but discussions with biology groups had underlined the importance of access to the compounds themselves for testing. Cluster analysis and commercial availability reduced this to a set of 400 representative compounds, the ‘Malaria Box’, which was distributed freely to researchers who provided a rationale for screening [5]. This paper presents a summary and analysis of the collected results of the Malaria Box screening from 55 groups who performed a wide variety of assays, the large majority of which are presented in this paper. The collective results are greater than the sum of the individual assays, because each compound can be queried for activity, pharmacokinetic, and safety data to gauge its suitability as a starting point for subsequent medicinal chemistry optimization efforts.

## Results

**Fig 1:**
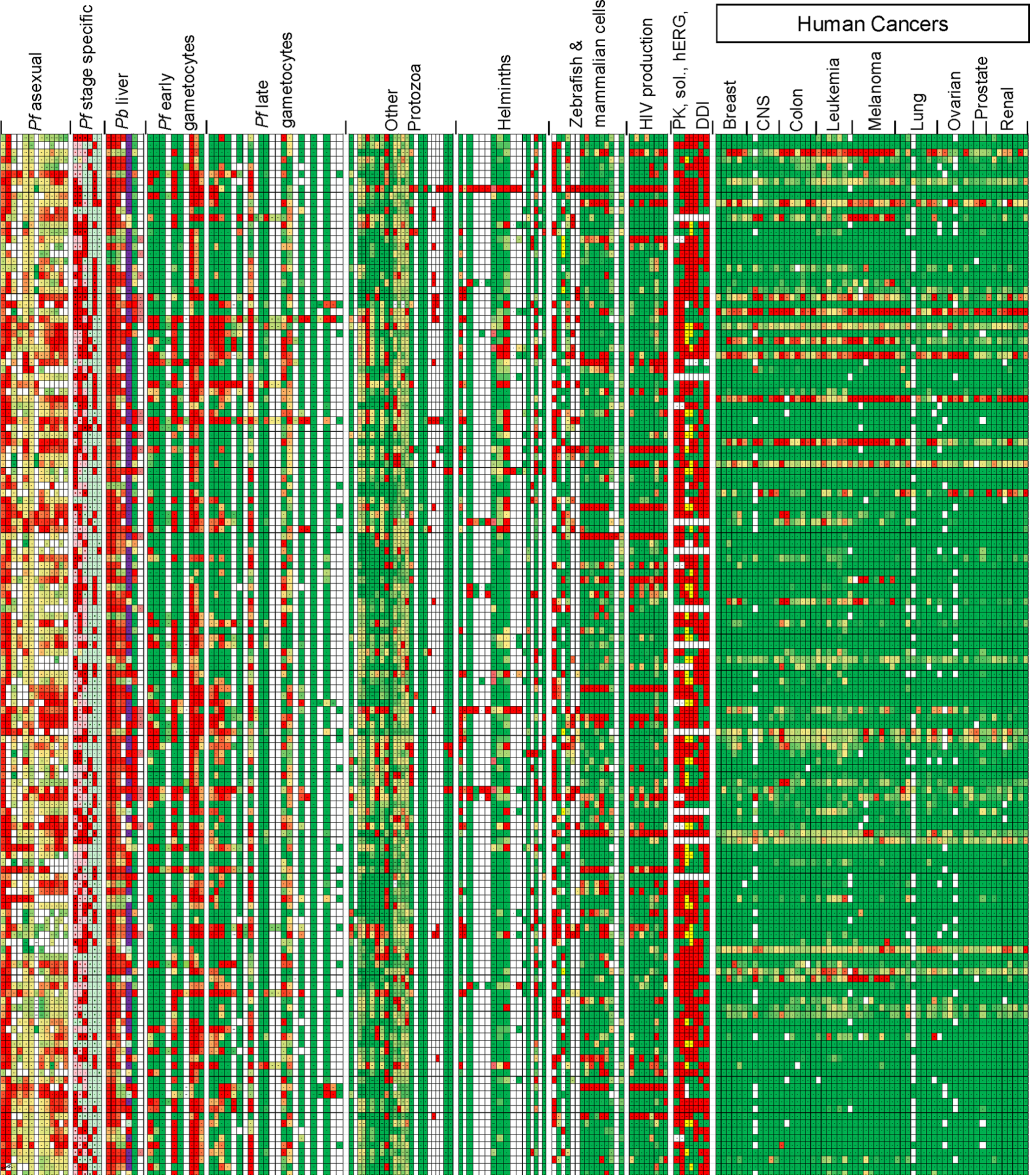
Malaria Box Heatmap. Shown are selected data from the HeatMap (S1 Table) for the 400 Malaria Box compounds. Each column represents an assay (grouped by category), compounds are represented in rows. The red-green gradient represents higher to lower activity. Favorable PK activities are scored green. *Pf*: *Plasmodium falciparum*, *Pb*: *Plasmodium berghei*, PK: pharmacokinetics, sol.: solubility, hERG: human ether-a-go-go channel inhibition, DDI: drug-drug interactions (predicted).

The Heat Map (S1 Table) reports the data from over 290 assays run on the Malaria Box compounds; a snapshot is shown in Fig 1. The results are color coded, where the compounds with the highest activity are coded red and those with relative inactivity green. In the center of the box in S1 Table, the numerical value for the compound is given. It can be seen immediately that some compounds have activities in several biological assays across multiple species and these tend to have activity against mammalian cells as well, whereas other compounds have a rather limited spectrum of activity and are less toxic to mammalian cells.

The data demonstrated in S1 Table are provided by 55 groups who have performed 291 assays to screen the Malaria Box. The vast majority of the data are presented for the first time in this paper. In supplementary data S1 Table, note that columns with data presented for the first time in this paper, representing 236 assays, are colored pink on the top row; published /in press data columns, 55, are grey, with citations provided. Presenting the combined dataset provides insights into the hit rates in these various assays while allowing rapid access to the data by the wider scientific community.

The Heat Map (S1 Table) presents the Malaria Box chemicals grouped by chemical relatedness. Of the 400 compounds, over 100 are closely-related paired molecules so immediate structure-activity-relationships (SAR) can often be seen from hits with these pairs. The Heat Map identified obvious correlations in chemistry and biology between compounds (both Mechanism-of-Action and phenotypic activity). Some biological assays are relatively similar; for example, there were a large number of different *P*. *falciparum* gametocyte assays (S1 Table, columns AV-CB), which also cluster, although not perfectly. As such, the aggregate screening data help overcome inter-laboratory bias and identify outstanding activities. For example, compounds that were active in multiple gametocyte assays represent more solid positives than a compound that was active in only one screening assay. However, the gametocyte assays were often performed using different techniques and screening concentrations (see S1 Methods and Results, for details) and one assay may be preferred over another to select compounds with gametocyte activity. Thus having the aggregate data presented together with the individual protocols is more valuable than just having each individual data set to look at sequentially.

### Malaria Box safety and pharmacokinetic data

Early safety data were obtained by testing all compounds against 73 human cell lines at 10 μM or above, and developing zebrafish embryos were exposed at 5 μM, providing further clues on potential safety issues. A frequent cardiotoxicity safety concern is QTc prolongation, and all compounds were screened for hERG inhibition [6], which is a proxy for this risk (S1 Table column GI). The efficacy and safety of anti-malarial compounds could be altered in endemic regions when administered to patients who are also treated for HIV (Human Immunodeficiency Virus) or TB (tuberculosis), due to drug-drug interactions in the liver. To flag such interactions, we employed two recent breakthrough models: a bioengineered microscale human liver in a high-throughput assay format that accurately captures human drug-drug interactions not detectable in animals or cell lines [7] and a custom-made, robotic high-throughput Luminex bead-based method for profiling the expression of 83 human liver drug-metabolizing enzymes [8]. Combining these tools, we profiled the Malaria Box compounds for induction or inhibition of drug-metabolizing pathways (S1 Table, columns GL-HA) and thereby ranked compounds for potential for drug interactions with existing HIV and TB regimens, to enhance selection of compounds with the lowest safety risks. We also scored the Malaria Box compounds for acute hepatoxicity by monitoring morphology and daily albumin and urea secretion from hepatocytes (S1 Table, columns FQ-FS).

G protein-coupled receptors (GPCRs) represent the largest human drug target class [9]; they affect neurological and cardiovascular physiology and are included in routine safety pharmacology panels [10]. Therefore, *in vitro* affinity determinations on 23 selected human off-target GPCRs were performed on a subset (10%) of MMV compounds (S1 Table, columns HC-HZ). One of the most severe GPCR-related adverse effects is cardiac valvulopathy linked to 5-HT_2B_ activation [11, 12]. Therefore, some of the MMV compounds with significant binding affinity for the 5-HT_2B_ receptor were also tested on the corresponding functional assay to determine a potential agonistic effect. In addition, predictions of compound glutathione reactivity and epoxidation potential were calculated for each of the Malaria Box compounds (S1 Table, columns IB-IC). These combined safety results alert us to compounds with issues that hopefully can be resolved in subsequent medicinal chemistry programs.

Prior to *in vivo* pharmacology evaluation it is important to know that an effective plasma concentration can be reached; this exposure was measured in rodents for all compounds, from a single high oral dose (140 μmol/kg). Around one third of the compounds generated high plasma C_max_ (>1 μg/ml) and/or high overall exposure (S1 Table, columns GD-GE). This is a higher than expected percentage of compounds with measureable oral bioavailability than if compounds were randomly selected, and probably reflects the large number of drug-like leads selected for the Malaria Box. The combination of *in vitro* potency and bioavailability provides a rough dosing estimate, informing subsequent decision-making around selection of development leads.

The combined analysis of all of these safety and pharmacokinetic data allows selection of the most promising compounds to advance to medicinal chemistry, and which parameters should be monitored and improved during a medicinal chemistry program.

### New insights into malaria

The activity of Malaria Box compounds against the asexual, erythrocytic stage of *P*. *falciparum* was confirmed by five laboratories on seven different *P*. *falciparum* strains. There were sometimes 5-10-fold differences in the effective concentration that caused a 50% reduction in growth (EC_50_) in each assay, and these may have been due to variations in the readouts for the screening assays (LDH release, MitoTracker or Sybr Green dye incorporation, hypoxanthine incorporation, DAPI imaging assay), variations in the protein concentration in the assay medium (affecting the free compound concentration), the time the compound incubated time, or other differences. However, usually the results were consistent and strain-independent. We have documented which sub-stage of the asexual lifecycle the compounds acted upon (S1 Table, columns AA-AE). This information is important in identifying compounds that may overcome existing resistance against artemisinin and other antimalarials. For instance, compounds that target early ring stage intra-erythrocytic parasites and have fast-killing dynamics are sought after because, like artemisinins, they kill parasites rapidly and may reduce patient mortality. Table 1 shows compounds that also target liver stages of the parasite’s life cycle.

**Table 1:** Malaria Box compounds with activity in biological assays (malaria, helminths, *Wolbachia*, and cancer cells) and lacking toxicity at therapeutic levels. Selectivity Index, SI, is toxicity level/activity level; p, probe-like; d, drug-like.

Antimalarial positives		Antihelminthic positives			Anti-*Wolbachia* positives	Anticancer positives		
All drug-like (d) *P*. *falciparum* early ring stage compounds (EC50 <200 nM, SI >10) with gametocyte activity	*P*. *falciparum* asexual, liver, & gametocyte activity	*Brugia malayi*	*Trichuris trichuria*	*Ancylostoma ceylanicum*	*Wolbachia*	MMV Number	MGLI50 = Mean Growth Log I50	Notes
MMV000248^d^	MMV006913^d^	MMV007907^d^	MMV008294^p^	MMV666607^p^	MMV000642^p^	MMV007384^p^	-7.34	Colon, differential, potent
MMV006087^d^	MMV007116^d^	MMV019241^p^	MMV666601^p^		MMV008138^d^	MMV019074^d^	-4.91	Specific lines sensitive
MMV006455^d^	MMV007199^p^	MMV665831^p^			MMV396664^p^	MMV665803^d^	-5.01	
MMV006706^d^	MMV007907^d^	MMV666054^p^			MMV665824^p^	MMV665796^d^	-5	
MMV011567^d^	MMV020700^d^				MMV665841^p^	MMV020275^d^	-4.93	
MMV011795^d^	MMV665843^d^				MMV665890^d^	MMV000760^d^	-6.02	Differential
MMV020505^d^	MMV665977^p^				MMV665897^d^	MMV666020^p^	-4.9	
MMV020660^d^	MMV666095^p^				MMV665948^d^	MMV665969^p^	-6.29	
MMV396749^d^					MMV666075^d^	MMV666597^p^	-5.57	Differential
MMV396794^d^					MMV666597^p^	MMV006962^p^	-5.61	Differential, CNS
MMV665805^d^					MMV666601^p^			
MMV665878^d^					MMV666607^p^			
MMV665915^d^								

### Targeting disease-relevant malaria stages

*P*. *berghei* liver stage (LS) inhibition, using parasite-encoded luciferase activity as a readout of infection in HepG2 cells, was independently determined by two groups at very different screening concentrations (Hanson: 5 μM, Winzeler: 50 μM). Forty-three compounds, roughly 10% of the compound library, inhibited infection by at least 50% at 5 μM and 90% at 50 μM (referred to as LS double actives). HepG2 cell toxicity, (50% or greater reduction in HepG2 abundance based on direct or indirect readouts) was observed with 63% of Malaria Box compounds at 50 μM, while only 10% were toxic at the 5 μM concentration. After excluding those that showed significant toxicity in HepG2 cells at both 5 and 50 μM, Malaria Box compounds were stratified by potential mode-of-action annotation (S1 Table, column M). Five potential modes of action stood out as enriched in LS double actives: (*i*) cysteine protease inhibitors (cruzain, rhodesain): 1.8% of all Malaria Box compounds (7/400) and 4.7% (2/43) of LS double actives; (*ii*) possible respiratory-dependent targets (Δ IC_50_ in low oxygen *vs*. normal oxygen): 0.8% (3/400) of all Malaria Box compounds and 4.7% (2/43); (*iii*) targeting yeast respiration: 3.5% of all Malaria Box compounds (14/400) and 9.3% of LS double actives (4/43); (*iv*) suspected or known *Pf*DHODH (dihydroorotate dehydrogenase) inhibitors: 2.5% of all Malaria Box compounds (10/400) and 9.3% of LS double active (4/43); and (*v*) suspected or known cytochrome bc1 inhibitors: 4.3% of all Malaria Box compounds (17/200) and 16.3% of LS double actives (7/43). Compounds with activity against *Pf*ATP4, now the most common intra-erythrocytic asexual target seen in phenotypic screens, were not found amongst the LS double actives.

There is a great need for antimalarials that kill *dormant*, liver-stage *P*. *vivax* (hypnozoites), but there is a lack of assays that measure this activity. Only nine compounds (Table 1) show simultaneous activity against gametocytes, liver, and asexual stages, whilst lacking evidence of toxicity in zebrafish and broad cytotoxicity to mammalian cells. These would be compounds to prioritize for *in vitro* and *in vivo* screening against *P*. *vivax* hypnozoites and would benefit from additional MoA studies.

Gametocytocidal drugs would block transmission from the human to the mosquito and break the parasite’s life cycle. The data shown in Table 1 include series with activities on both gametocyte and liver stages, and some of the data intriguingly challenges existing assumptions. For instance, MMV007116 in this category is a mitochondrial (bc1) inhibitor (S1 Table, column M, line 168) and has activity in a number of gametocytocidal assays, but other bc1 inhibitors are not generally gametocytocidal, suggesting another MoA for this compound. We also see 4-aminoquinolines as inhibitors of some gametocyte assays, although the parent 4-aminoquinoline compound chloroquine is known not to be gametocytocidal for *P*. *falciparum*. Again, this may imply a different MoA for some 4-aminoquinoline compounds or perhaps multiple modes of action for certain compounds. These findings re-emphasize the strength of looking at assay data in a wider context in Open Source drug discovery.

### Mechanism-of-action screening

Data from one-hundred-nineteen MoA assays for compounds from the Malaria Box are included, identifying potential targets for 135 of them (S1 Table and S1 Methods and Results). The MoA assay data are presented in Column M of S1 Table, and further information about the screens and their results are given in S1 Methods and Results. These screens included biochemical screens for enzyme inhibition, protein-protein interactions, behavior by altered yeast or malaria organisms, and a variety of other screens. Some associations are strong and have been followed up with additional experimentation (*e*.*g*. MMV008138 and its target *Pf*-IspD [13–15]), but most target associations are still tentative. Indeed, some listed MoA activities occur only at higher concentrations than activity in cell-based screens and therefore are unlikely to explain that compound’s activity against a pathogen or tumor cell. In addition, many MoAs have been inferred for malaria, but are less likely to apply to the diverse groups of organisms screened with the Malaria Box compounds.

Surface plasmon resonance (SPR) was used to identify nine compounds which inhibit four sets of protein-protein interactions (PPI), without overlap between sets (S1 Methods and Results), suggesting that molecules were identified that specifically target these protein-protein interfaces. Compounds inhibiting *P*. *falciparum* (autophagy-related proteins) Atg8-Atg3 PPI were MMV007907, MMV001246 and MMV665909 (S1 Table, column M). They had a pronounced effect on all stages of gametocyte development, which supports the idea of *Pf*Atg8-Atg3 being involved in remodeling and vesicular trafficking in gametocyte development. Six compounds inhibited *in vitro* translation in *P*. *falciparum* lysates by more than 60% at a concentration of 1 μM (S1 Table, column L; [16]). One of these protein translation-inhibiting compounds, MMV007907, is interesting in that it had activity against both liver and gametocyte stages as well as a broad range of other pathogens, and has low toxicity to human cell lines. Twenty-six compounds either inhibited the mitochondrial electron transport chain (bc1, 11 compounds) or DHODH (15 compounds). Since both the bc1 and DHODH pathways converge on pyrimidine biosynthesis, it is interesting that almost all bc1 inhibitors had anti-liver stage and anti-male gametocyte activity, while the anti-male gametocyte property was generally lacking in most DHODH inhibitors [17–19].

*Pf*ATP4 is a *P*. *falciparum* plasma membrane protein with genetic variants that confer resistance to several new clinical and preclinical antimalarials [20–24]. *Pf*ATP4 has been proposed to function as a Na^+^:H^+^ pump, effluxing Na^+^ from (and importing H^+^ into) the malaria parasite [21]. Parasites exposed to 28 MMV Malaria Box compounds have shown ion-homeostasis changes similar to those observed with likely *Pf*ATP4 inhibitors (indicated in column K, S1 Table) [25], and thus are inferred to be *Pf*ATP4 inhibitors. Analysis of the 281 assays’ results with these compounds, reported here, allows detailed conclusions about the potential effects of ATP4 inhibition in *Plasmodium* as well as other organisms. From the Malaria Box data summarized here, it is evident that the 28 *Pf*ATP4-associated hits tended to be inactive against the variety of non-Apicomplexan protozoa, helminths, insects, yeast and bacteria that were tested. An exception was *Trypanosoma cruzi*, that was growth-inhibited by almost 40% of the *Pf*ATP4 inhibitors (11/28), compared to an overall hit rate of 20%. It should be noted that the non-*Plasmodium* Apicomplexan parasites against which the majority of the compounds were tested–*Cryptosporidium parvum*, *Toxoplasma gondii*, *Theileria equi* and three species of *Babesia*–were not, in general, particularly susceptible to the *Pf*ATP4-associated hits. There is not, to our knowledge, any evidence that the other Apicomplexan parasites against which the Malaria Box was tested are exposed to a high-Na^+^ environment within their host cells, and this may explain the lower sensitivity to inhibition of a Na^+^ efflux mechanism. In contrast, infection of an erythrocyte by *Plasmodium* is followed by an increase in the Na^+^ concentration in the erythrocyte cytosol as a result of the induction of broad-specificity (Na^+^-permeable) ‘New Permeability Pathways’ in the host erythrocyte membrane [26–28]. This suggests that perturbation of Na^+^ efflux through inhibition of *Pf*ATP4 is uniquely, highly detrimental to intra-erythrocytic malaria parasites.

There is prior evidence that *Pf*ATP4-associated compounds are active against gametocyte stages of *P*. *falciparum* [5, 22–24, 29–32]. Twenty-five of the 28 *Pf*ATP4-associated hits (89%) caused some inhibition of male gamete formation at 1 μM (*i*.*e*. had positive % inhibition values; S1 Table). It should be noted, however, that approximately half of the *Pf*ATP4-associated hits have IC_50_ values for the killing of asexual parasites that are similar to or higher than the 1 μM concentration used in the gamete formation assay. Only 65% of the *Pf*ATP4 non-hits tested had positive values for inhibition of male gamete formation at 1 μM. An increase in extracellular pH is known to trigger the exflagellation of male *P*. *falciparum* gametes, raising the possibility that an increase in intracellular pH in male gametocytes or gametes, resulting from PfATP4 inhibition, triggers premature exflagellation, leading to parasite death. Thus, it is possible that an increase in intracellular pH in male gametocytes or gametes resulting from *Pf*ATP4 inhibition triggers premature exflagellation leading to their death.

**Fig 2:**
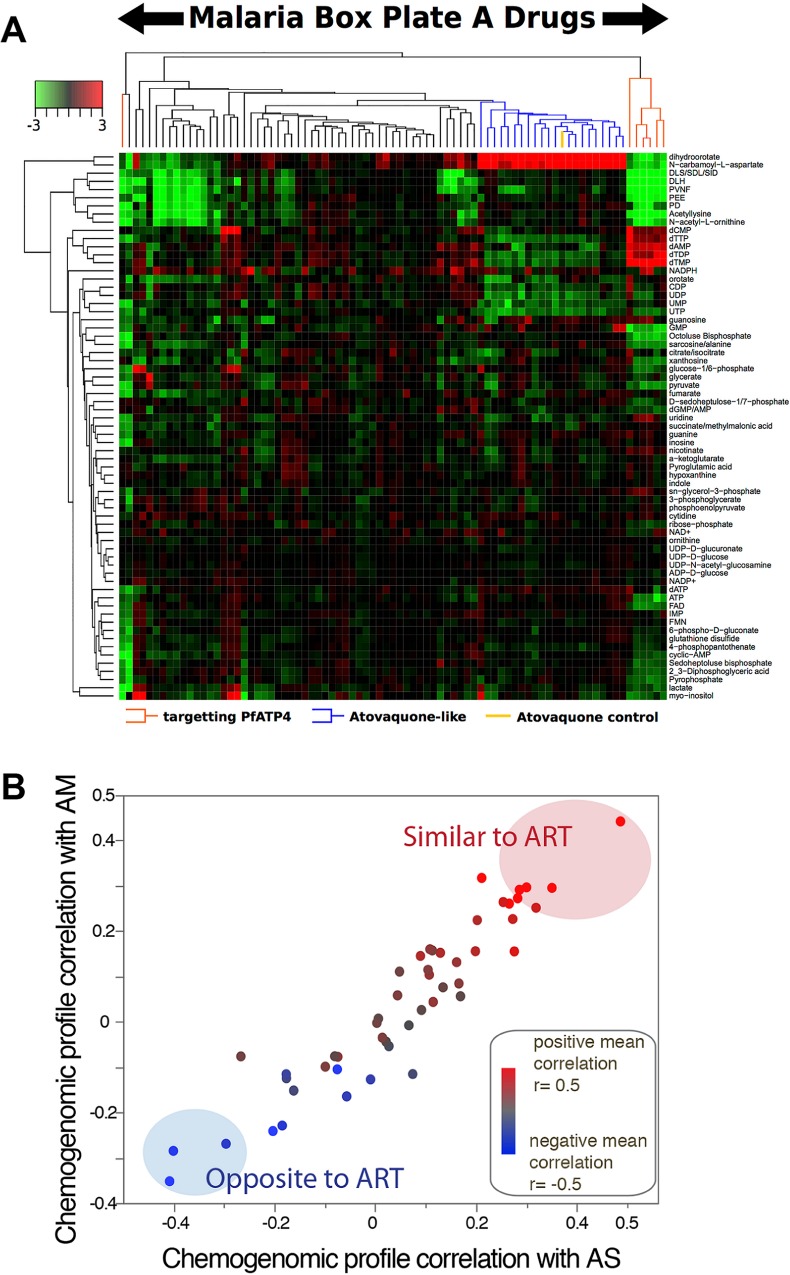
Metabolomic and chemogenomic profiling. (A) Metabolic profiling: Heat map showing metabolic fingerprints of 80 Malaria Box compounds and atovaquone control. Parasite extracts were analyzed by LC-MS, and changes in metabolite pools were calculated for drug-treated parasites as compared to untreated controls. Hierarchical clustering was performed on ^2^log-fold changes in metabolites (data in S2 Table), scaled from -3 to +3. Six of seven compounds (indicated in red) reported to target *Pf*ATP4 [25] showed a distinct metabolic response characterized by the accumulation of dNTPs and a decrease in hemoglobin-derived peptides. A large cluster of compounds (indicated in blue) clustered with the atovaquone control (indicated in orange), and exhibit an atovaquone-like signature characterized by dysregulation of pyrimidine biosynthesis, and showed a distinct metabolic response characterized by the accumulation of dNTPs and a decrease in hemoglobin-derived peptides. (B) Chemogenomic profiling: A collection of 35 *P*. *falciparum* single insertion *piggyBac* mutants were profiled with 53 MMV compounds and 3 artemisinin (ART) compounds [Artesunate (AS), Artelinic acid (AL) and Artemether (AM)] for changes in IC_50_ relative to the wild-type parent NF54 (data in S3 Table, genes queried in S4 Table). Clone PB58 carried a *piggyBac* insertion in the promoter region of the K13 gene and has an increased sensitivity to ART compounds as do PB54 and PB55 [33]. Drug-drug relationships based on similarities in IC_50_ deviations of compounds generated with *piggyBac* mutants created chemogenomic profiles used to define drug-drug relationships. The significance of similarity in MoA between Malaria Box compounds and ART was evaluated by Pearson’s correlation calculations from pairwise comparisons. The X axis shows the chemogenomic profile correlation between a Malaria Box compound and AS, the Y axis with AM; the color gradient indicates the average correlation with all ART derivatives tested. Five Malaria Box compounds (MMV006087, MMV006427, MMV020492, MMV665876, MMV396797) were identified as having similar drug-drug chemogenomic profiles to the ART sensitivity cluster.

Malaria box compounds were also screened against asexual stages using metabolomic and chemogenomic profiling (Fig 2). Using metabolomic profiling to examine the metabolic responses to the 80 compounds in plate A, six of seven compounds believed to target *Pf*ATP4 [25] showed a distinct metabolic response characterized by an accumulation of dNTPs, and a decrease in hemoglobin-derived peptides (Fig 2A, S2 Table). Twenty-one compounds clustered with atovaquone, an inhibitor of the bc1 complex of the electron transport chain, exhibiting an atovaquone-like signature characterized by the dysregulation of pyrimidine synthesis. Of these 21 atovaquone-like compounds, 17 were also identified by other groups as targeting the electron transport chain or pyrimidine synthesis. For chemogenomic profiling, a collection of 35 *P*. *falciparum* single insertion *piggyBac* [33] mutants were profiled with 53 MMV compounds and three artemisinin (ART) compounds [Artesunate (AS), Artelinic acid (AL) and Artemether (AM)] for changes in IC_50_ relative to the wild-type parent NF54 (Fig 2B, S3 Table, S4 Table). Five Malaria Box compounds (MMV006087, MMV006427, MMV020492, MMV665876 and MMV396797) were identified as having similar drug-drug chemogenomic profiles to the ART-sensitivity cluster (Fig 2B). These compounds may be rapid killers, like artemisinin, and should be explored further for confirmation, and whether they can overcome artemisinin-resistance for ring-stage killing.

### Screening on yeast to suggest MoAs

Four groups carried out screens on *S*. *cerevisiae* strains engineered to help elucidate the MoA of test compounds. One screen established that 35 Malaria Box compounds were active on a multiple ABC-transporter deficient strain (also known as the ‘monster strain’) *S*. *cerevisiae* [34]. Since yeasts are generally resistant to compound inhibition due to transporters, this monster strain can now be analyzed for MoA of inhibition by these 35 compounds. A second study measured selective growth inhibition of *S*. *cerevisiae* using different carbon sources. Growth was measured in three different growth media: rich or minimal media using dextrose as a carbon source, or minimal media using ethanol and glycerol as carbon sources. Compounds affecting growth in a media-specific manner may represent inhibitors of key metabolic pathways. A third group used a yeast strain expressing the *Pf* phosphoethanolamine methyltransferase (*Pf*PMT) to screen for phosphocholine (PC) synthesis inhibitors. This screen relies on the incapability of this yeast strain to synthesize PC in the absence of exogenous choline, and thus depends on the malaria *Pf*PMT for survival. Screening the Malaria Box compounds, and a variety of controls including wild-type PMT and choline supplemented media, led to the identification of MMV007384, MMV007041 MMV396736, MMV396723, MMV000304, MMV000570, MMV000704, MMV666071, MMV000445, MMV667491, and MMV666080 as possible *Pf*PMT inhibitors. Finally, a fourth group screened *S*. *cerevisiae* grown either on ethanol-containing media requiring respiration or glucose-fermentative media not requiring respiration, and identified 12 compounds that gave superior inhibition on ethanol media suggesting that these compounds inhibit a respiratory target. Seven of these were not associated with any other targets; the others were potential inhibitors of DHODH (3, 49), bc1, and IspD.

### Activity against protozoa other than Plasmodium

The Malaria Box was screened against 16 additional protozoa, all of which are of medical or veterinary significance. Compounds with activity against three or more protozoa were usually toxic for the zebrafish or non-cancer mammalian cell lines, underlining the need to limit the concentrations used in assays, to avoid meaningless positives. Table 2 lists compounds with activity against protozoa that were nontoxic to zebrafish and most mammalian cells. In the *Cryptosporidium parvum* assay there were numerous active compounds, but none were completely devoid of toxicity for zebrafish and mammalian cell lines. MMV665917 had a >20-fold Selectivity Index (SI) for *C*. *parvum* over mammalian cells. *Trypanosoma cruzi* actives were non-overlapping between groups, and are listed separately, but *T*. *brucei* actives overlapped extensively with other screens and are presented together. There were seven non-toxic hits that were active against extracellular amastigotes of *Leishmania infantum*, but no non-toxic compounds were active on intracellular macrophage growth of *L*. *infantum*. There were five non-toxic Malaria Box compounds active against *T*. *gondii* (MMV666095, MMV007363, MMV007791, MMV007881 and MMV006704). Many of the compounds that were active on *Neospora caninum* raised no toxicity flags on the accompanying host cell fibroblast screen, but many were toxic at 10 μM or below for mammalian cells and zebrafish. The remaining non-toxic *N*. *caninum* actives that bear further investigation include: MMV019670, MMV000911 and MMV006309. Most compounds active against *Entamoeba histolytica*, *Naegleria fowleri*, or exflagellation of *Chromera velia* were toxic. An exception was MMV665979, an outstanding hit for *Naegleria fowleri*, with limited toxicity elsewhere in the dataset. With respect to screening *Babesia* and *Theileria*, ten novel anti-*Babesia* and anti-*Theileria* hits with nanomolar IC_50_s were identified: MMV666093, MMV396794, MMV006706, MMV665941, MMV085203, MMV396693, MMV006787, MMV073843, MMV007092 and MMV665875. The most interesting hits were MMV396693, MMV073843, MMV666093, MMV665875 and MMV006706 with mean SIs greater than 230 and IC_50_s ranging from 43 to 750 nM for both bovine *Babesia* and equine *Babesia* and *Theileria* parasites. Additionally, 64, 45 and 49 Malaria Box compounds exhibited IC_50_s lower than those of diminazene aceturate (the most widely used antibabesial drug) against the *in vitro* growth of *B*. *bovis*, *B*. *bigemina* and *T*. *equi*, respectively.

**Table 2:** Antiprotozoal Malaria Box compounds with activity in biological assays and lacking toxicity at therapeutic levels. Selectivity Index, SI, is toxicity level/activity level; p, probe-like; d, drug-like.

Antiprotozoal positives										
*Cryptosporidium parvum* (SI>20)	*Trypanosoma brucei* (positive in all three screens, and SI >10)	*T*. *cruzi* (Scynexis screen)	*T*. *cruzi* (Maes screen)	*Leishmania donovani* amastigotes axenic (extracellular):	*Toxoplasma gondii*	*Babesia spp*. (3) AND *Theileria equi*	*Babesia spp*. (3) OR *Theileria equi* (excluding active on all 4 *spp*.)	*Naegleria fowleri*	*Neospora caninum*	*Perkinsus marinus*
MMV665917^p^	MMV020505^d^	MMV006558^p^	MMV001230^d^	MMV000356^d^	MMV007363^d^	MMV006706^d^	MMV000620^*Bc*^	MMV665979^d^	MMV000911^d^	MMV666081^d^
	MMV020548^d^	MMV006706^d^	MMV007907^d^	MMV007907^d^	MMV007791^d^	MMV666093^d^	MMV000653^*Bbi+Bbo*^		MMV006309^p^	
		MMV006764^p^		MMV011438^p^	MMV666095^p^	MMV396693^p^	MMV000911^*Bc*^		MMV019670^d^	
		MMV007571^d^		MMV019127^d^	MMV007881^d^	MMV073843^p^	MMV006764^*Bbo+Bbi*^			
		MMV011256^d^		MMV665979^d^	MMV006704^d^	MMV665875^p^	MMV007374^*Bc*^			
		MMV019199^p^		MMV665987^p^			MMV007557^*Bc+Bbi*^			
		MMV498479^p^		MMV666686^p^			MMV007808^*Bc*^			
		MMV665841^p^					MMV007906^*Bc*^			
		MMV665843^d^					MMV008173^*Bbo+Bbi*^			
		MMV665878^d^					MMV008455^*Bbi*^			
		MMV665890^d^					MMV011438^*Bbo+Bbi*^			
		MMV665899^d^					MMV018984^*Bc+Bbo*^			
		MMV665908^p^					MMV019199^*Bbo+Bbi*^			
							MMV019202^*Bc*^			
							MMV019670^*Bc*^			
							MMV019995^*Bbi*^			
							MMV396681^*Bc*^			
							MMV665878^*Bbi*^			
							MMV665879^*Bbo+Bbi*^			
							MMV665890^*Bbo+Bbi*^			
							MMV665908^*Bbi+Bbo+Te*^			
							MMV665939^*Bbo+Bbi*^			
							MMV665946^*Te*^			
							MMV666009^*Bc+Bbi*^			
							MMV666025^*Bbo*^			
							MMV666095^*Bc*^			
							MMV666116^*Te+Bbi*^			
							MMV666689^*Bbo+Bbi*^			

*In vitro* screening of Open Access Malaria Box compounds against *Babesia bovis*, *B*. *bigemina*, *Theileria equi* and *B*. *caballi* has led to the discovery of 10 novel potent anti-babesial hits exhibiting submicromolar potency against both bovine *Babesia* and equine *Babesia* and *Theileria*. *In vitro* follow up of the many of the hits identified in this study for *B*. *bovis*, *B*. *bigemina*, *B*. *caballi*, and *T*. *equi* parasites, revealed IC_50_s lower than that obtained with the previously described drug-leads luteolin, pyronaridine, nimbolide, gedunin and enoxacin [35]. The ten potent hits for bovine *Babesia* and equine *Babesia* and *Theileria* identified in this study exhibited IC_50_s lower than that obtained with the apicoplast-targeting antibacterials (ciprofloxacin, thiostrepton, and rifampin), miltefosine, fusidic acid or allicin [36–39].

### Activity on helminths, mycobacteria, and bacteria

Many Malaria Box compounds were active on helminths at 10 μM, but most of these were also toxic for mammalian cells or zebrafish. The remaining non-toxic compounds had activity against *Brugia malayi* (lymphatic filariasis) and *Ancylostoma ceylanicum* (hookworm; Table 1). But no non-toxic compounds were found with consistent activity against *Schistosoma mansoni*, *Strongyloides stercoralis*, *Trichuris muris*, *Haemonchus contortus*, or *Onchocerca linenalis*. There remains the possibility that some of the toxic hits against these species can be addressed by medicinal chemistry.

With respect to activity against mycobacteria and bacteria, although every screen delivered actives, the majority were again discarded because of a toxicity signal against zebrafish and/or mammalian cells. The exceptions were non-toxic Malaria Box compounds that were active against *Wolbachia* (Table 1). *Wolbachia* bacteria are targeted as anti-filarials in order to deprive nematodes causing river blindness and elephantiasis from essential nutrients provided by this bacterium [40].

### Activity on cancer cells

The US National Cancer Institute has screened 59 human tumor cell lines (‘NCI60’) against the Malaria Box compounds at 10 μM (S1 Table and S1 Methods and Results). Among the 133 compounds further evaluated for dose-responses, and the ten of these then tested in confirmatory assays (S1 Text), MMV007384 was selected for potency and focused activity against colon cancer cells, and has been advanced to an *in vivo* proof-of-concept experiment.

## Discussion

Academic drug discovery is highly fragmented. Many biology groups, especially those in disease-endemic countries, excel in developing highly disease-relevant pathogen models suitable for low- to medium-high throughput screening, but suffer from lack of access to innovative compounds. If they do have access to compounds, then they may fail to share the results, or lack drug development skills. The Malaria Box Project demonstrates how an open source approach allows effective data sharing: this publication serves as much to share the data among the 180+ co-authors as with the wider scientific community. By publishing in concert this ensures early publication and also sharing of ideas and expertise in drug discovery. New insights and series have been obtained for malaria (nine pan-stage active molecules which had not been previously prioritized). Moreover, screening against pathogens for additional neglected diseases has been catalyzed and hits found. The sharing of data from safety screens flags compounds that probably work through a general toxicity mechanism, and those compounds can be down-prioritized at an early stage. This is key to prioritizing compounds for medicinal chemistry, since the paucity of good starting points against some parasites has encouraged groups to screen at what may be inappropriately high drug concentrations. Another advantage of having a standardized, publically available library and dataset is that this allows benchmarking assay sensitivity, setting compound concentrations for expanded screens and deciding on acceptable hit criteria [41].

We saw some discrepancies in the values obtained for the same compounds in similar assays that were carried out by multiple groups, such as activity against asexual or gametocyte forms of *P*. *falciparum*, *Trypanosoma spp*., and mammalian cells. In this sense, compounds that were positive in more than one assay would clearly be more likely to represent a true positive than compounds that were positive in only one screen. Some of these apparent discrepancies were probably due to variations in the techniques used for the screens. For instance, many methods used to measure gametocytocidal activity measure a specific metabolic activity. Because the metabolism will be affected by many factors that will lead to differences in output, including media composition (albumax versus serum), how old the media used was, purity of the gametocytes (how much asexual contamination and cell debris is present). In addition, the tested compounds varied widely in their propensity to bind to protein in the assay medium, and large differences in the protein content in two assays could lead to differences in unbound compound. Only the free compound would likely be available for activity in biological assays. Some assays had extensive follow up, and if a compound was tested and activity confirmed with a dose-response, it is more likely to be a true positive than a compound flagged as positive from a single screening run. This complex dataset highlights the need to consider integrating more standardized criteria, such as similar (free) compound concentrations, assay media, or compound exposure duration, into future screening initiatives of this nature. This could potentially reduce inter-assay differences, and facilitate more direct data comparison across the different platforms. However it is clear in the case of gametocyte screens, that different assays that interrogate different biological processes do not necessarily achieve the same result for a given compound, even when the assay conditions have been standardized [42]. And trying to standardize assays may be counterproductive with the goal of convincing multiple groups to run their assays on a given set of compounds.

The MoAs associated with compounds (S1 Table, Column M) vary from very strong associations such as chemical-genetic evidence, to relatively weak associations, such as activity in a single biochemical screen at relatively high compound concentration. Thus most of the associations should not be taken as definitive MoA of the compounds for their biological activities. All associations were presented because not only could they be hypothesis-building for the discovery of a compound’s disease-relevant MoA, but also because the Malaria Box compounds now represent a rich source of bioactive compound tools.

With its outcomes continually evolving, the Malaria Box has already made an impact by stimulating medicinal chemistry for many diseases. We are aware of such new medicinal chemistry programs against pathogens such as *Plasmodium* [43–45], *Babesia*, *Toxoplasma* [46], *Trypanosoma* [47–49], *Cryptosporidium* [31], *Schistosoma* [50], filaria, *Echinococcus*, helminths, bacteria, cancer and other diseases [30]. Ensuring that data becomes freely available is a challenge, and this paper represents the first such summary of over 290 screens against the compound collection, highlighting new activities and new MoAs. For the future, three goals are important. First, to track these compound series to ascertain whether any of these hits do become leads of drug development candidates. Second, data must be rapidly published, even with follow-up incomplete. Finally and most importantly, this model can be taken further. A second collection of 400 compounds, the Pathogen Box, (www.PathogenBox.org) based on compounds known to be active in phenotypic screens against an expanded set of pathogens responsible for neglected and tropical diseases has now become available from the Medicines for Malaria Venture. It is hoped this can be the start of equally fruitful collaborative networks.

## Methods

See S1 Methods and Results for further details.

The Malaria Box is a set of 400 compounds that were previously shown to be active against asexual stages of *P*. *falciparum in vitro*. The process for Malaria Box compound selection was published previously [5], with 200 drug-like compounds as starting points for oral drug discovery and development and 200 diverse probe-like compounds for use as bioactive tools research. The selection was made to represent the broadest cross-section of structural diversity and, in the case of the drug-like compounds, properties commensurate with excellent oral absorption and the minimum presence of known toxicophores. One limiting factor was that compounds had to be commercially available; this limited the chemical space displayed in the original set of 20,000 malaria bioactives.

The Malaria box was shipped to 193 different research groups in 29 different countries as frozen 96-well plates with the compounds dissolved at 10 mM in 20 μl DMSO (dimethylsulphoxide). Two years after shipping the first Malaria Box, the 193 groups were re-contacted and asked if they wanted to participate in a group publication disseminating and comparing the results from the Malaria Box screens. Forty-seven of these groups did not reply to our multiple requests. Fifty-nine groups had not yet initiated screening, but 26 of these had only received the Malaria Box in the preceding three months. Thirty-one groups had publications in preparation and 39 papers have already been published [5, 14, 16, 25, 30–32, 42–46, 48–75]. Fifty-five groups agreed to contribute data and participate in this paper and provided data from 291 assays.

The compounds were then screened in biochemical and biological screens as documented in detail in S1 Methods and Results. More detailed methods are provided for screens presented in this paper than for those whose results have already published. In addition, S1 Methods and Results provides data for both positive and negative controls obtained for each assay. In most assays, a single-concentration screen was run first and bioactives were identified. Some work was stopped after the primary screen, but most groups went on to perform confirmatory assays, and many provided hit concentrations that achieve 50% activity (S1 Table). The assays included a variety of cell-based pathogen screens covering multiple taxonomic groups, including *Plasmodium* (multiple life-stages), other protozoa, bacteria, mycobacteria, HIV, and also multicellular-organism screens such as helminths and a mosquito (See Fig 1 and S1 Table).

## Supporting Information

S1 Methods and Results Supplementary Methods and Results. (DOCX)

S1 Table Malaria Box HeatMap. (Reference numbers refer to S1 Methods and Results references; Pink Headers signify data presented first in this paper data; Grey headers are published, submitted, or in press). Red shading means active and green means inactive and values are provided in each square. Favorable PK activities are scored green.
(XLSX)

S2 Table Metabolomic data. The file is ordered as shown in Fig 2A and shows log2 fold changes compared to an untreated control. Positive and negative values indicate increase and decrease, respectively, as compared to the control.
(XLSX)

S3 Table Chemigenomic data. (XLSX)

S4 Table Genes queried in Chemogenomic approach. (XLSX)

S1 Text Supplementary Dose-Response data on selected cancer cell lines. (PDF)
