# Supplementary material for: Open Source Drug Discovery with the Malaria Box Compound Collection for Neglected Diseases and Beyond
Source: PLoS Pathog. 2016 Jul 28;12(7):e1005763. doi: 10.1371/journal.ppat.1005763 (PMC4965013; doi:10.1371/journal.ppat.1005763)
Supplement: S1 Methods and Results — (DOCX) [file ppat.1005763.s001.docx]

**Open-source drug discovery with the Malaria Box compound collection for neglected diseases and beyond**

**Supplementary Methods and Results:**

**Keyed to the columns on the HeatMap (Supplementary data S3).** Much of the data, 135 assays on the Malaria Box compounds, have been deposited and are curated in ChEMBL (www.ebi.ac.uk/chembldb). See at the end of this document for more details.

Most of the data is data presented for the first time in this paper but when the data was previously published, and presented for meta-analysis, the reference is given.

**Column J,** Swamidass group (data presented first in this paper). Chemical similarity: The procedure that was used to make this order consists of 3 steps: First, the Scaffold Network Generator (SNG) was used [1], a tool developed in the Swamidass lab, to search for molecules’ scaffolds (column I). Second, the scaffold similarity matrix was calculated using path fingerprints [2, 3]. Third, hierarchical clustering was used to cluster molecules by their distance matrix (1 - [similarity matrix]). The final order is the order of branches in the hierarchical tree.

**Column K,** Lehane/Kirk group**,** (published in ref [4]). *Pf* ATP4 mechanism of action. Methods: Each of the 400 Malaria Box compounds was tested at a concentration of 1 µM for an effect on the resting intracellular Na^+^ concentration ( [Na^+^]_i_) of asexual *P. falciparum* trophozoites (Dd2 strain), functionally isolated from their host cells by brief exposure of infected cells to saponin then loaded with the Na^+^-sensitive fluorescent dye SBFI as described [5]. Compounds that disrupted parasite [Na^+^]_i_ or that gave rise to confounding ‘optical effects’ in these assays were subsequently tested for their effects on intracellular pH, using saponin-isolated trophozoites loaded with the pH-sensitive fluorescent dye BCECF [6]. Full details of the approach taken and of the further characterization of ion-disrupting ‘hits’ can be found in here [4]. Result/Analysis: In screening the Malaria Box compounds for their effects on parasite ion regulation, Lehane *et al.* [4] reported that 28 compounds (*i.e.* 7% of the collection), belonging to 16 unique chemotypes, had effects consistent with inhibition of *Pf*ATP4, a *P. falciparum* plasma membrane protein [7] that has been proposed to function as a Na^+^:H^+^ pump, effluxing Na^+^ from (and importing H^+^ into) the parasite [5]. The ‘*Pf*ATP4-associated’ compounds caused an immediate-onset increase in both the Na^+^ concentration and pH in the parasite cytosol, as well as a reduction in the extent of cytosolic acidification seen on inhibition of the parasite’s plasma membrane V-type H^+^-ATPase with concanamycin A [4]. None of these 28 *Pf*ATP4-associated ‘hits’ affected parasite ATP levels on the timescale of the experiments, ruling out the possibility that ion disruption was a secondary consequence of ATP depletion [4]. Spiroindolones and dihydroisoquinolones comprised two of the 16 ion-disrupting chemotypes. Promising lead compounds from these chemical classes (as well as other classes not represented among the *Pf*ATP4-associated hits in the Malaria Box [8, 9]) have been reported in separate studies to disrupt parasite Na^+^ and pH homeostasis [5, 10]. The data available thus far are consistent with, but not proof of, *Pf*ATP4 being the direct target of diverse chemotypes. In addition to the finding that the chemically diverse Malaria Box hits altered the Na^+^ concentration and pH in the parasite, six hits (belonging to five separate chemotypes) selected for further study were found to have a lower antiplasmodial potency against a spiroindolone-resistant *Pf*ATP4-mutant line (from [7]) than against its matched parental line [4]. Two additional compounds identified by Lehane *et al*. as disrupting parasite Na^+^ and pH regulation [4], but not investigated further, were shown by a different group to have antiplasmodial potencies that varied depending on the *Pf*ATP4 variant of the strain tested [8]. In the study by Lehane *et al*. two of the Malaria Box hits were used to generate resistant parasites *in vitro*, and in each case the resistant parasites generated were found to harbour a mutant variant of *Pf*ATP4 and to show cross-resistance to the spiroindolone KAE609 [4]. Activity on other microorganisms: From the Malaria Box data summarized here it is evident that the 28 *Pf*ATP4-associated hits tended to be less active than non-hits against the variety of non-Apicomplexan protozoa, helminths, insects, yeast and bacteria that were tested. This is perhaps to be expected given that *Pf*ATP4 belongs to “a subclass unique to Apicomplexan organisms” [11]. One exception was the *T. cruzi* screen at 5μM by Scynexis in which 11 of 28 *Pf*ATP4-associated hits (36%) gave >65% growth inhibition at a concentration of 5 μM. The overall hit rate for this screen was 80/400, *i.e.* 20% of the Malaria Box compounds gave rise to >65% inhibition at 5 μM. Interestingly, the non-*Plasmodium* Apicomplexan parasites against which the majority of the compounds were tested – *Cryptosporidium parvum, Toxoplasma gondii, Theileria equi* and three species of *Babesia* – were not, in general, particularly susceptible to the *Pf*ATP4-associated hits. For these organisms the average growth inhibition (measured as either the percentage inhibition seen at a particular concentration or the 50% inhibitory concentration) observed for the *Pf*ATP4-associated hits was similar to that seen for non-hits. For both the *Pf*ATP4-associated hits and the non-hits from the Malaria Box the potency was, in general, lower for the non-*Plasmodium* organisms than for *Plasmodium*. Unlike *Plasmodium*, these Apicomplexan parasites may spend little time in a high-Na^+^ environment, and this may render them less sensitive to inhibition of a Na^+^ efflux mechanism. Infection of an erythrocyte by *Plasmodium* is followed by an increase in the Na^+^ concentration in the erythrocyte cytosol, as a result of the induction of broad-specificity (Na^+^-permeable) ‘New Permeability Pathways’ on the host erythrocyte membrane [12-14]; the intra-erythrocytic malaria parasite is therefore exposed to a high-Na^+^ environment. There is not, to our knowledge, any evidence that the other *Apicomplexan* parasites against which the Malaria Box was tested are exposed to a high-Na^+^ environment within their host cells. Activity on *Plasmodium*: There is prior evidence that *Pf*ATP4-associated compounds are active against sexual stages of *P. falciparum*. *In vitro* assays have revealed that the lead spiroindolone KAE609 inhibits gametocyte development [15], and that the lead pyrazoleamide PA21A092 inhibits the formation of gametes from both male and female Stage V gametocytes [9]. Furthermore, KAE609 and a Na^+^-disrupting aminopyrazole inhibit the formation of oocysts when added directly to the mosquito blood meal [8, 15]. The lead dihydroisoquinolone (+)SJ733 was shown to inhibit the transmission of *Plasmodium* *berghei* from mice to mosquitoes at doses lower than those needed to kill asexual parasites [10]. With the exception of some experiments performed on Stage IV and/or V gametocytes, *Pf*ATP4-associated hits are, in general, somewhat more potent against gametocytes than non-hits, although the level of inhibition seen for the different hits is highly variable. A more detailed analysis can be found here [4]. Significantly, in the “Dual Gamete Formation Assay” [16], the *Pf*ATP4-associated hits tended to be more potent at inhibiting male gamete formation than female gamete formation. Twenty-five of the 28 *Pf*ATP4-associated hits (*i.e.* 89%) caused some inhibition of male gamete formation at 1 µM (*i.e.* had % inhibition values > 0). By contrast, only ten of the hits had % inhibition values >0 for female gamete formation at the same concentration. The average (± SD) level of inhibition of male gamete formation by the hits at a 1 µM concentration was 23 ± 20%; for female gamete formation it was -6 ± 18%. It should be noted that nearly half of the *Pf*ATP4-associated hits have IC_50_ values for the killing of asexual parasites (in at least two of the asexual killing assays shown in Heat Map columns P-Z) that are higher than the 1 µM concentration used in the gamete formation assay. Only 65% of the *Pf*ATP4 non-hits tested had positive values for inhibition of male gamete formation at 1 µM, with the average level of inhibition being 7 ± 36%. An increase in extracellular pH (from ~pH 7 to 8), which at least in asexual *P. falciparum* leads to a (smaller) increase in intracellular pH [17], has been shown to trigger the exflagellation of male *Plasmodium* gametes [18]. Thus, one possibility is that an increase in intracellular pH in male gametocytes or gametes resulting from *Pf*ATP4 inhibition triggers exflagellation before the cells are ready, leading to their death

**Column L,** DeRisi group [19], possible translation inhibitors defined with *P. falciparum in vitro* translation capacity, with 100% activity normalized to 1.0. Methods: *P. falciparum* W2 strain was grown in hyper flasks to late trophozoite stage. Parasites were lysed and homogenized and the supernatant was used for *in vitro* translation assays. The lysate was combined with 1μM of drug and used to *in vitro* transcribe mRNA encoding luciferase. The assay was carried out for 1.5h before being assayed with firefly luciferin. The results are normalized translation to the no drug control averages. Each Malaria Box plate was assayed with 3 replicates.

**Column M,** Possible Mechanism (s) of Action:

**Protease inhibitors *Pf*A-M1 & -M17,** Florent group (data presented first in this paper): Experimental procedures: Recombinant proteins. A genomic DNA fragment encoding residues 192-1085 of native *Pf*A-M1 [20, 21] was amplified from genomic DNA of the FcB1 strain of *P. falciparum,* with primers appending a N-terminal hexahistidine tag followed by cleavage site for the tobacco etch virus (TEV) protease (ENLYFQS), and was cloned into the *Bam*H1 and *Not*I sites of the pET45b vector (Novagen). A synthetic gene (Genecust, Luxembourg) encoding a cleavage site for the tobacco etch virus (TEV) protease fused to residues 84-605 of native *Plasmodium falciparum* leucyl aminopeptidase *Pf*A-M17 (PlasmoDB *PF*3D7_1446200), was cloned into *Bam*HI and *Sal*I sites of the pET45b vector (Novagen), which appended an N-terminal hexahistidine tag (Revelant *et al*., in preparation). Both plasmids were transformed into *Escherichia coli* BL21 (DE3) Rosetta 2 (Novagen) after appropriate validations by Sanger sequencing (Beckman Coulter Genomics). Bacterial cultures were grown in auto-induced LB medium (Merck) supplemented with appropriate antibiotics (carbenicillin 50 µg.ml-1, chloramphenicol 34 µg.ml-1) during 24 hours at 25°C under vigorous agitation. The clarified lysates were loaded onto Ni^2+^-charged HisTrap column (GE Healthcare) equilibrated in phosphate buffer supplemented with 20 mM imidazole, extensively washed in phosphate buffer supplemented with 20 mM imidazole, and bound recombinant proteins were eluted in respectively 80 mM imidazole in phosphate buffer (r*Pf*A-M1) and 200 mM imidazole in phosphate buffer (r*Pf*A-M17). Eluted fractions were extensively dialyzed at 4°C into respectively 50mM Tris-HCl, 200 mM NaCl, 10 µM ZnCl_2_, pH 7.4 (r*Pf*A-M1) and 50 mM Tris-HCl, 200 mM NaCl, 10 µM ZnCl_2_ pH 8 (r*Pf*A-M17). Enzymology. Activities of r*Pf*A-M1 (400 nM) and r*Pf*A-M17 (250-600 nM) were determined by measuring the release of fluorogenic leaving group NHMec (excitation: 370 nm, emission: 460 nm) as described previously [21]. Tests were carried out using a FL600 Fluorescence Microplate reader (BioTek Instruments, Winooski, Vermont) at 37°C, during 40 min in 50mM Tris-HCl, pH 7.4 for r*Pf*A-M1 and 50mM Tris-HCl, 1mM CoCl_2_ pH8 for r*Pf*A-M17. Reactions were started by the addition of L-Leu-NHMec (equivalent to 1 Km), after 20min of incubation (RT) with each MMV inhibitor at a final concentration of 100µM (1% DMSO final concentration). Each MMV inhibitor was tested at least three times; bestatin, a classical aminopeptidase inhibitor targeting aminopeptidase inhibitor targeting M1 (57% inhibition at 100µM) and M17 (100% inhibition at 100µM) aminopeptidases was used as a positive control [22].

Results. MMV666102, MMV667490 and MMV006825 inhibitors were particularly effective on r*Pf*A-M1. MMV666102 can be considered as a dual-inhibitor because of its effectiveness to also inhibit r*Pf*A-M17, while MMV667490 and MMV006825 were only moderately inhibiting r*Pf*A-M17. These three molecules are however different from the ones identified by the McGowan group on the same targets [23].

**Protease inhibitors M1, M17, M18,** McGowan group (published in Ref [23]) : Compounds were screened against the *P. falciparum* M1, M17 and M18 aminopeptidases in a single dose, multiplex assay [23]. Compounds exhibiting inhibition of any of the three enzymes at 100 μM were analyzed further. MMV020750 and 666023 were determined to be moderate competitive inhibitors of both M1 and M17. There were no inhibitors of M18 identified in the screen.

**Cysteine Protease Inhibitors Cruzain and Rhodesain**, Ferreira group (data presented first in this paper). Recombinant cruzain was gently provided by Allison Doak and Dr. Brian Shoichet (*University of California, San Francisco*), while rhodesain was kindly provided by Dr. Conor Caffrey (*Center for Discovery and Innovation in Parasitic Diseases, Skaggs School of Pharmacy and Pharmaceutical Sciences, University of California San Diego*). Cruzain and rhodesain activity were measured as previously described [24], (Ferreira *et al*., 2010) by monitoring the cleavage of the fluorogenic substrate Z-Phe-Argaminomethylcoumarin (Z-FR-AMC) in a Synergy 2 (Biotek), from the Center of Flow Cytometry and Fluorimetry at the Biochemistry and Immunology Department (Universidade Federal de Minas Gerais, UFMG). Formation of fluorescent 4-amino-7-metyhlcoumarin was measured employing 340 nm wavelength for excitation and 440 nm for emission. Stock solutions of compounds at 1 mM in DMSO were prepared. 1 µL of the stock solution was added to a total assay volume of 200 µL, resulting in a compound concentration of 5 µM in the screening. All assays were performed in sodium acetate buffer 0.1 M pH5.5 and in the presence of 1 mM β-mercaptoethanol and 0.01% Triton X-100, at 25°C. The final concentration of cruzain and rhodesain was 0.5 nM, and the substrate concentration was 2.5 μM. Initially compounds were pre-incubated with the enzyme for 10 minutes in 100 µL assay buffer, then 100 µL of 5 μM substrate solution was added and the reaction was immediately monitored. Assays were performed in duplicate, followed for 5 min and activity was calculated based on initial velocities. Percentages of inhibition were calculated in comparison with DMSO controls, which were employed in each assay to allow controlling for variations in enzyme activity from one assay to another. Reported values refer to average and standard errors calculated based on duplicates. In all assays the general cysteine protease inhibitor E-64 was employed as a positive control, at the concentration of 500 nM, and throughout all assays it consistently inhibited enzyme activity by approximately 100%. Data was analyzed with Prism 5.0 (GraphPad). Malaria Box compounds were marked as possible inhibitors if they exceeded 74% inhibition at 5 µM. Eighteen compounds meet this criterion for cruzain, twenty for rhodesain and nine for both enzymes.

**CDPK1 and CDPK4 Protein Kinases**, Ojo & Van Voorhis groups (Published in ref [25]): METHOD: Measurement of inhibition of *Pf*CDPK1 and *Pf*CDPK4 protein kinase phosphorylation properties by small molecule inhibitors was done by a non-radioactive Kinase-Glo luciferase assay reagent (Promega, Madison, WI) as previously described [26]. Dimethyl sulfoxide (DMSO) at a final concentration of 4% was used as negative control while BKI-1 was used as positive control. None of the tested inhibitors or the negative control (4% DMSO) was found as a hit for *Pf*CDPK1. *Pf*CDPK4 had only one hit, MMV666599, at 0.7µM IC_50_. The calculated IC_50_ value for the BKI-1 positive control was 4 nM as earlier described [26].

**MAPK2, PK6, PK7 Protein Kinases,** Crowther & Van Voorhis groups (Published in ref [25])). METHOD: We tested Malaria Box compounds for possible inhibition of catalytic activity of three *P. falciparum* protein kinases: MAPK2 (*PF*3D7_1113900/*PF*11_0147), PK6 (*PF*3D7_1337100/MAL13P1.185), and PK7 (*PF*3D7_0213400/*PF*B0605w). Each kinase’s activity was considered proportional to ATP consumed, as determined from measurements of residual [ATP] with the luciferase-based reagent Kinase-Glo (Promega) following incubation periods of 3-4 hours. The PK7 assay was an autophosphorylation assay; substrates for MAPK2 and PK6 were histone III-S and myelin basic protein (MBP), respectively [25]. Compounds were screened at a final concentration of 1 µM. Control inhibitors were 100 µM AMP-PNP (MAPK2), 10 µM staurosporine (PK6), and 100 µM 1NA-PP1 (PK7) and all showed >80% inhibition under the assay conditions. Wells with DMSO only served as additional controls. Results: none of the 400 compounds inhibited MAPK2, PK6, or PK7 >20% at 1 µM.

***Pf*CDPK1, *Pf*PK6, *Pf*CK2, *Pf*GSK3, *Pf*CLK1, *Pf*map2, *Pf*PK7, *Pf*Nek2, *Pf*Nek4, FIKK4.2, FIKK10.2, *Pf*CK1, *Pf*PK5 Protein kinases,** Lucet & Wilks group (data presented first in this paper). METHOD: Two assays were performed. We first tested the Malaria Box compounds on purified recombinant *Pf* kinases by Thermal Shift Assay [27] with 2 μM of the individual kinase protein and a 5 molar excess of compound. SYPRO Orange was used as fluorescent probe. Compounds were considered positive if a shift of more than 2° C was observed and were further evaluated to confirm inhibition of kinase activity using an enzymatic assay (ADP-Glo luciferase assay , Promega). All Pf proteins were expressed overnight at 16°C for 16 to 20 hours in *E. coli* C41(DE3). All kinases were purified in two steps by means of a 6XHis/Ni column, followed by a size exclusion chromatography. *Pf*CDPK1, *Pf*GSK3, *Pf*CK2, *Pf*map2, *Pf*PK7, *Pf*Nek2, *Pf*Nek4, *Pf*PK6, *Pf*PK5 were expressed as full-length. FIKK4.2, FIKK10.2, *Pf*CK1, PfCLK1 were expressed as kinase domain**.** Two assays were performed. We first tested the Malaria Box compounds on purified recombinant *Pf* kinases by Thermal Shift Assay as described in [27]. Briefly, thermal-shift assays were with purified proteins diluted in 20 mM Tris, pH 8, 150 mM NaCl, 1 mM DTT to 2 µM of the purified protein and assayed with a 5 molar excess of compound. SYPRO Orange was used as fluorescent probe and detected at 530 nm. Two generic kinase inhibitors, DAP ((N*2*-(4-Aminomethyl-phenyl)-5-Fluoro-N*4*-Phenyl-Pyrimidine-2,4-Diamine) and VI16832 were used a positive controls at a concentration of 40 µMμM [27]. Sample fluorescence was plotted as a function of increasing temperature. The melting temperature (T_m_) and changes in the unfolding transition temperature compared to the control curve (ΔT_m_) were calculated as described in [27]. Compounds were considered positive if a ΔT_m_ of more than 2° C was observed. Results: Using this approach, three compounds MMV665852, MMV665853 and MMV403679 were shown to induce a shift of approximately 2° C by thermal shift assay. These compounds were further evaluated for possible inhibition of kinase activity using an enzymatic assay (ADP-Glo luciferase assay, Promega). However, none of these compounds showed inhibition when tested at a final concentration of 5 µM.

**Aminoacyl-tRNA ligases,** Nakazawa Hewitt & Van Voorhis group (data presented first in this paper). Method: *Plasmodium falciparum*-tRNA ligases *Pf* ProRS Prolyl-tRNA synthetase and *Pf* KRS Lysyl-tRNA Synthetase were screened against Malaria Box compounds using the Kinase-Glo Luminescent Kinase Assay. The PfProRS was protein PlfaA.18681.a.B2 Batch 3 and was an amino-terminus truncation of 7 amino acids from the full-length predicted cytoplasmic *Pf*ProRS and was produced as detailed on the Seattle Structural Genomics for Infectious Diseases (SSGCID.org) website. The PfKRS was protein PlfaA.00612.a.MB2 PW37450 an amino-terminus truncation of 77 amino acids from the full-length predicted cytoplasmic *Pf*KRS and was produced as detailed on the SSGCID.org website. All components required for *in vitro* amino acid charging by the cognate tRNA ligase were added in the reaction well and enzyme progression was determined by measuring ATP consumption. Reaction conditions for PfProRS assays were 75 nM ProRS, 60 μM L-proline, 6 μM ATP, 400 μg/ml bulk yeast tRNA (Sigma), .05% IGEPAL, 0.2 mM Spermine, 0.1 mg/ml BSA, 2.5 mM DTT, 5.3 U/ml pyrophosphatase, 25 mM Hepes-KOH, 20mM MgCl2, 50mM KCl, pH 7.8 and incubation for 2 h at 37 °C. Reaction conditions for KRS experiments were 150 nM Pfal KRS enzyme, 60 μM L-lysine, 3 μM ATP, 400 μg/ml bulk yeast tRNA, .05% IGEPAL, 0.2 mM Spermine, 0.1 mg/ml BSA, 2.5 mM DTT, 5.3 U/ml pyrophosphatase, 25 mM Hepes-KOH, 20mM MgCl2, 50mM KCl, pH 7.8 and incubation for 3 h at 37 °C. Positive controls were halofuginone (PfProRS) and Cladosporin (PfKRS) and both gave 100% inhibition at 1µM. Kinase-Glo substrate reacts with free ATP resulting in luminescent signal; thus, enzyme inhibition is directly related to higher luminescence signal detection [28]. Results: None of the 400 Malaria Box compounds inhibited more than 20% at the highest concentration tested, 10 µM.

***Pf*/PvEg5 (Kinesin 5) inhibitor,** Wojcik group (published in ref [29]). Methods: We screened each compound for their effect on the ATPase activity of recombinant *Plasmodium falciparum* (*Pf*Eg5) and *P. vivax* (PvEg5) Kinesin-5 microtubule motor proteins. Our screen utilized the isolated motor domains of these enzymes, and was screened in parallel with the human Kinesin-5 homolog as a control for selectivity. Two different, but widely used, high-throughput ATPase assay methods were used in our screen, a colorimetric Malachite Green assay, and the NADH-coupled ATPase assay. We identified one compound, MMV666693 from the Malaria Box collection as a selective inhibitor of both *Pf*Eg5 and PvEg5 that does not affect HsEg5 or several other human kinesins. We find that MMV66693 does not compete with ATP or microtubules for interaction with the *Plasmodium* motors. This indicates that MMV66693 is an allosteric inhibitor of the *Plasmodium* Kinesin-5 motors and, as expected, exhibits a high degree of selectivity for this specific Kinesin-5 homolog. The overall pattern of effects on the *Plasmodium* life cycle is consistent with an impact by MMV66693 on nuclear division. That is, this compound is effective in halting the proliferation of blood stage parasites and inhibiting proliferation in liver stage parasites as well [29].

**Possible *Pf*HT-1 inhibitor**, Landfear and Guy groups (published in [30]). Testing Malaria Box Compounds for Selective Inhibition of the Malaria Hexose Transporter *Pf*HT. Methods: Malaria Box compounds were screened for those that selectively inhibited *Pf*HT versus the human glucose transporter GLUT1. The approach employed a transgenic strain of *Leishmania mexicana* in which the endogenous hexose transporters had been deleted by targeted gene replacement and then complemented with either *Pf*HT or GLUT1 expressed on an episomal vector. Compounds were first screened for their ability to inhibit growth of the *Pf*HT expressing line of *L. mexicana* growing on glucose as the carbon source and were then tested for their ability to inhibit uptake of radiolabeled glucose by each of the two transgenic lines. Methods and results were previously reported [30].

**Possible PfHDAC1 and PfHSP90 inhibitors,** Brennan and Chubb groups (data presented first in this paper). Methods: Histone deacetylase enzyme assay: *P. falciparum* HDAC1 was purchased from Sigma Aldrich Ireland. The enzyme was used at a concentration of 2 ng/ml. The enzyme activity was assessed using the HDAC glo I/II assay system (Promega, Madison, USA). Compounds were screened at a concentration of 20 µM and normalized to the enzyme control with no inhibitor added (n=4). The known inhibitor trichostatin A inhibited the assay at 50nM (89.9 ± 0.5% inhibition, p<0.05). The DMSO vehicle control did not significantly inhibit the assay (6.4 ± 2.1% inhibition, NS). Statistical analysis was performed using repeated measures ANOVA with a Dunnett’s post-test using GraphPad Prism, GraphPad Software, San Diego, California USA. Differential Scanning Fluorimetry: Thermal shift assays were performed using a 7900HT Fast Real-Time PCR System (Applied Biosciences, San Francisco, USA) according to Lo et al. 2004. *P. falciparum* HSP90 was used at 0.5mg/ml diluted in a HEPES buffer (100 mM HEPES (pH7.4), 150 mM NaCl buffer). Compounds were tested at 25 µM. Sypro Orange dye (Life Technologies, California, USA) was used at 10 X concentration. Data was analyzed by non-linear regression using Prism to identify the melting temperature (Tm). The Tm of each compound incubated with the protein was compared to the melting temperature of the protein incubated with the DMSO vehicle control. The experiment was performed three times and statistical analysis performed using one-way ANOVA followed by the Dunnett’s post-test comparing the Tm’s for the MMV compound treatments to the Tm for the DMSO vehicle control. Reproducible changes in temperature (ΔTm) of >2º C with p- values <0.001 are reported [31].

**Possible *Pf* Deoxyhypusine-hydrolase inhibitors,** A. Kaiser group (published in refs [32, 33]). Over recent years we established different analytical assays for the identification and quantification of the hypusine metabolites in *Plasmodium* [32-35]. The post translational modification (PTM) of hypusine is unique and present in eukaryotic initiation factor 5A (eIF-5A). The enzymes of this pathway, *i.e.* deoxyhypusine synthase (DHS) and deoxyhypusine hydroylase (DOHH) committing the sequential two enzymatic steps of this pathway were intensively characterized from different *Plasmodium* species. For the identification of deoxyhypusine and hypusine two different rapid and robust, non-radioactive analytical methods were implemented. Ultraperformance liquid chromatography (UPLC) enabled us to separate deoxyhypusine and hypusine with retention times of 7.44 min and 7.30 min, respectively after derivatization of the primary amino groups with 6-aminoquinolyl-N-hydroxysuccinimidyl carbamate (AccQ-Tag, Waters Germany). Secondly, a GC-MS method was employed to screen for efficient inhibitors from the Malaria Box which determined the hypusine metabolites in a peptide hydrolysate after derivatization of the primary amino groups with methyl chloroformate resulting in a molecular ion [M]^+^ at 347 and esterification of the free carboxylic groups with a molecular fragment of [M-OCH3] with a molecular ion [M-31]^+^. This analytical method was further developed to quantify inhibition of the hypusine biosynthetic enzymes by selective derivatization of the amino groups with a mixture of trifluoroacetic acid anhydride and the carboxylic group with trifluoroacetic acid ethyl ester extracting the mass ions for quantification. In a final step a selective derivatization of the hydroxyl group present in hypusine was obtained with hexamethylsilazane. Absolute quantification was performed by using the internal standard norvaline and chemically synthesized hypusine and deoxyhypusine.

**Possible dUTPase & protein 6-Phosphogluconolactonase inhibitors**, Quinn group (Manuscript in preparation, R Quinn & H. Vu). Bioaffinity Mass Spectrometry Screening (BMSS) method is a novel screening approach using electrospray ionization Fourier Transform Mass Spectrometry (ESI-FTMS) under physiological condition as a detection means [36, 37]. Each spectrum was an average of 32 transients (scans) composed of 256,000 data points. The FTMS spectrum shows the protein and the non-covalent protein-ligand complex (Figure).

Bioaffinity Mass Spectrometry Screening Method

Protein preparation

Recombinant proteins were prepared by the Van Voorhis group according to the same protocol as in [25]. Proteins were buffer-exchanged into a suitable volatile buffer (ammonium acetate or ammonium bicarbonate) for native ESI-MS. Depending on the protein, the buffer and its concentration is chosen to obtain the highest sensitivity in the mass spectrometer. We have chosen ammonium acetate buffer (10mM, pH 7) for these two proteins, dUTPase and 6-Phosphogluconolactonase.

6-Phosphogluconolactonase

MAHHHHHHMGTLEAQTQGPGSMDYENFVKSAEEINNLHNVNYLETKDLNDFNWKAAYYICKEIYDKQQINKDGYVVIGLSGGRTPIDVYKNMCLIKDIKIDKSKLIFFIIDERYKSDDHKFSNYNNIKFLFHNLNINEKEQLYKPDTTKSIVDCILDYNDKIKIMIEKYKKVDIAILGMGSDFHIASLFPNIFYNIYMNNYQNNYIYNEKTLDFINNDQDNDNLKYLKEYVYFTTTNQFDVRKRITVSLNLLANASSKIFLLNSKDKLDLWKNMLIKSYIEVNYNLYPATYLIDTSCTNENVNINNNNNNNNKNKNNYCYSNTTVISCGYENYTKSIEEIYDSKYALS

dUTPase

MAHHHHHHMHLKIVCLSDEVREMYKNHKTHHEGDSGLDLFIVKDEVLKPKSTTFVKLGIKAIALQYKSNYYYKCEKSENKKKDDDKSNIVNTSFLLFPRSSISKTPLRLANSIGLIDAGYRGEIIAALDNTSDQEYHIKKNDKLVQLVSFTGEPLSFELVEELDETSRGEGGFGSTSNNKY

Instrument Control

Agilent Electrospray tuning mix (G2431A), which gives signals at m/z 322, 622, 922, 1522 and 2122, was used for instrument control. The one-tenth dilution in methanol of the mix was infused by a syringe pump into the ESI source. Instrument parameters were tuned to achieve the highest signal intensity.

Experiment Control

The instrument was set up to detect weak protein-ligand non-covalent complexes. The protein bovine carbonic anhydrase II (bCAII) was used to tune the instrument and optimize the instrument parameters to obtain the highest signal intensity in native electrospray ionization condition. The specific weak inhibitor of bCAII, sulfanilamide, was mixed with the bCAII. The mass spectrometer was tuned to obtain the best signal of the [bCAII-sulfanilamide] complex.

The bCAII (EC 4.2.1.1, 29089 Da) was purchased from Sigma-Aldrich and used without further purification. It was dissolved in ammonium acetate (10mM, pH 7) to generate stock solution (34 µM). The sulfanilamide (C6H8N2O2S, 172 Da) was purchased from Sigma-Aldrich. It was dissolved in methanol to make a stock solution (5.8mM). The bCAII (100µL, 3.4µM) in ammonium acetate (10mM) was mixed with the sulfanilamide (10 µL, 581µM) and incubated for one hour at room temperature (20˚C). The inhibitor:protein ratio was 16:1.

Detection and Analysis

All experiments were performed at the optimum condition found for the detection of weak non- covalent complexes on a Bruker 4.7 Tesla Electrospray Ionization source Fourier Transform mass spectrometer. Mass spectra were recorded in the positive ion mode with a mass range from 50 to 6000 m/z for broadband low resolution acquisition. Each spectrum was an average of 32 transients (scans) composed of 512,000 datapoints. All aspects of pulse sequence control and data acquisition were controlled by Xmass control software under Windows operating system. The FTMS spectrum shows the protein and the noncovalent protein-ligand complex (Figure 1).


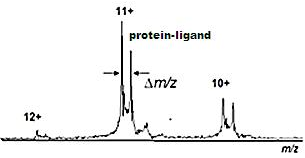


Figure 1 A mass spectrum of a protein and its protein-ligand complex

High-resolution Fourier transform mass spectrometry (FTMS) can identify the molecular weight of the bound ligand. The difference between the mass-to-charge ratio (Δm/z) for the unbound protein and the [protein-bound ligand] complex ions was multiplied by the charge state (z) to directly afford the molecular weight of the bound ligand, using the following equation.

**MW_ligand_ = *Δm/z* x *z***

The BMSS assay was performed on 400 Malaria Box compounds against 25 *Plasmodium falciparum* proteins, which are critical in malaria cell growth and invasion. Pools of eight Malaria Box compounds (each compound 1 µL, 1 mM in methanol) were incubated with a *P. falciparum* protein one hour at room temperature. When a non-covalent complex was found, the molecular weight of the binding compound was deducted from the spectrum. On the basis of this information, a binding confirmation on the selected compound was performed. For the confirmation binding assay two compounds, compound MMV008138 and compound MMV666599 were binding against the 6-Phosphogluconolactonase and the dUTPase, respectively. Compound MMV008138 (5 µL, 1 mM in MeOH) was incubated with 6-Phosphogluconolactonase (80 µL, 13 µM). The final compound:protein ratio was ~5:1. Compound MMV666599 (5 µL, 1 mM in MeOH) was incubated with dUTPase (50 µL, 17.8 µM). The final compound:protein ratio was ~5:1.

***Pf*PMT, *P. falciparum* phosphoethanolamine methyltransferase**, Ben Mamoum group (data presented first in this paper): see **Columns DV-DY methods below**

**Atg8-Atg3, Aldolase-TRAP, and Aldolase-MTRAP, MTIP-MyoA, GAP50-FactorH protein-protein interaction (PPI) inhibitor and Surface Plasmon Resonance screening to identify protein-protein interaction inhibitors,** Bosch group (Atg8-Atg3 interactions are published in ref [38], rest are not or as noted below). In a primary screen, the Malaria Box compounds were screened at 5 µM final concentration in 1x PBS supplemented with 0.01% (v/v) P20 and 1% (v/v) DMSO using a Biacore 3000 instrument [38]. While one protein was covalently attached to the chip surface via amine coupling, the analyte was passed over the chip in the presence and absence of Malaria Box compounds. Every 12th injection a positive control was passed over the chip to ensure constant binding of the partner protein during the duration of the experiment. Malaria Box compounds resulting in more than 25% decrease in binding of the two proteins to each other were further considered in a dose-response screen ranging from 50 µM to 200 nM. Similar to the primary screen, a positive control, consisting of the protein partner alone in the absence of small molecule compounds, was passed over the flow cells to ensure that the chip surface had not decayed over the course of the experiment. All SPR interaction studies were performed as previously described [38]. We have applied this primary screening method to several other important protein-protein interactions of the *Plasmodium* parasite and identified different sets of Malaria Box compounds capable of inhibiting a particular interaction (Figure 1). A detailed description of our findings of Malaria Box PPI inhibitors of the Aldolase-TRAP and Aldolase-MTRAP interaction is in revision (L.E. Boucher *et al*., submitted) as well as a manuscript describing the discovery of MTIP-MyoA and GAP50-FactorH inhibitors (D.C. Colón-López *et al*., in preparation). In all examples except of the GAP50-FactorH interaction, a short peptide sequence was expressed in *E. coli* BL21 DE3 cells as previously described [39]. A dodecapeptide, corresponding to the C-terminal residues of either *P. falciparum* or *P. vivax* TRAP (PlasmoDB codes PF3D7_1335900, PVX_082735) was expressed using the His_12_-Avitag expression vector (Boucher and Bosch 2013). A 16mer peptide corresponding to the C-terminal residues of *P. falciparum* MTRAP (PlasmoDB code PF3D7_1028700) was expressed to investigate the Aldolase-MTRAP interaction. To investigate the MTIP-MyoA interaction, we expressed the 15 C-terminal amino acids, corresponding to the previously described MyoA-peptide in the co-crystal structure [40] in the His_12_-Avitag expression vector. Expression and purification of the constructs was followed as previously described (Boucher and Bosch 2013). A single step TALON affinity purification was applied to capture the His_12_-peptide from the lysate. The Avitag-sequence contains a single Lysine residue that is biotinylated during the expression in *E. coli* and serves to capture the peptide to a CM5 SPR chip immobilized with Neutravidin. Complement FactorH Domains 5-7 were codon optimized for expression in *E. coli* fused to an N-terminal His_12_-Avitaq sequence, expression and purification was performed similar to the purification described for the peptides. Only the inhibitors that repeatedly led to the most potent PPI inhibition were noted in Column H. MMV007907, MMV01246 and MMV665909, identified as PPI inhibitors of the *Plasmodium falciparum* Atg8-Atg3 interaction, show a pronounced effect on all stages of gametocyte development, which supports the idea of *Pf*Atg8-PE being involved in remodeling and vesicular trafficking. MMV666071 and MMV666080, both identified against the *Pf*MTIP-MyoA interaction, and part of the glideosome inhibit gametocytes as well at the concentrations tested by other groups (S.M.S Stamm *et al*., manuscript in preparation).
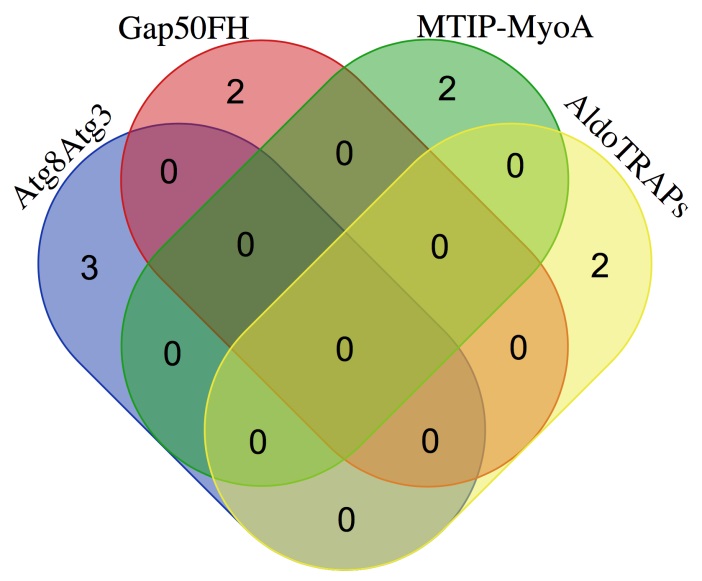


Figure 1: Identification of protein-protein interaction inhibitors using SPR. Five protein-protein interactions were tested. For simplicity the Aldolase-TRAP and Aldolase-MTRAP interaction was considered as one group. The Venn diagram reveals no overlap of compounds between the different interactions, indicating that specific inhibitors were identified that probe the respective protein-protein interfaces.

**Yeast screening for enhanced drug effects in combination with artemisinin**, Jensen group (data presented first in this paper). Selection for compounds that exhibit enhanced toxicity in combination with artemisinin. Yeast (*S. cerevisiae*) was used as a proxy for *P. falciparum,* because the killing of *S. cerevisiae* and *P. falciparum* appear to have similarities [41, 42]. *Saccharomyces cerevisiae* strain WC081 (Mat a, leu2∆0, lys2∆0, ura3∆0, his3∆1, pdr1∆::HIS3, pdr3∆::kanMX4) was utilized and was originally derived from strain BY4742. Regulators of Multi-drug resistance transporters have been deleted from this strain to enhance the toxicity of compounds. Assay utilized the disc diffusion method with the observance of a clear zone indicating inhibition of growth. Synthetic complete medium containing 3% glycerol as a carbon source (SCG) was used in each assay. 10 µL of each compound at 1 mM was added to a paper disc which was placed on SCG and SCG^+^artemisinin (0.2 µM) plates seeded with 10e6 yeast cells. Plates were incubated at 30˚C for 5 days and clear zones were measured. Compounds that produced a larger clear zone in the presence of artemisinin were considered to have an additive interaction with artemisinin. Results are reported as diameter of clear zone on SCG^+^artemisinin plates - diameter of clear zone on SCG plates without artemisinin. Only 2 compounds of the 80 compounds (plate A) tested displayed the ability to enhance toxicity of artemisinin: MMV001246 (3 mm larger zone size [lzs]) and MMV011099 (7 mm lzs). Both MMV001246 and MMV011099 did not produce an observable clear zone in the absence of artemisinin under conditions tested and appear to potentiate the toxicity of artemisinin in yeast cells. MMV665941 had an extremely large zone size with yeast but there was no enhanced effect with artemisinin.

**ETC (electron transport chain) mitochondrial inhibition MoA. (Annotated MoA in Column M, data in Column KM, KN),** Gamo group (data presented first in this paper**). Method:** To investigate if compounds act through the inhibition of the *Plasmodium* mitochondrial electron transport chain (ETC), similarly to atovaquone, or via inhibition of the pyrimidine biosynthesis pathway, parasite strains expressing either endogenous (*P. falciparum*) or yeast (*Saccharomyces cerevisiae*) dihydroorotate dehydrogenase (DHODH) were utilized. 3-fold dilutions were used in dose responses assays at concentrations ranging from 10 µM to 0.0002 µM. LDH activity was used as a surrogate of *Plasmodium* viability. Pre-dispensed plates with 50 nl of compounds were inoculated with 25 μL of a parasitized RBC suspension (2% hematocrit, 0.25% parasitaemia) and incubated 72 h. LDH activity was monitored in a final volume of 100 μl of 100 mM Tris-HCl buffer, pH 8.0, containing 0.5% Tween 20, 100 mM lactate, 100 μM APAD^+^,125 μM NBT (Nitro blue tetrazolium) and 1 U/ml Diaphorase. Absorbance at 650 nm was measured to follow the reaction. The IC_50_ calculations were performed with Grafit 5 software. We observed that 9 chemical clusters and 5 singletons exhibited reduced activity against the transgenic *P. falciparum* Dd2-ScDHODH strain as compared to the parental *P. falciparum* Dd2-attB strain as shown by a ratio value >5 (Column M) suggesting that these compounds could inhibit the *Plasmodium* mitochondrial ETC and/or DHODH. Those compounds which shown an IC_50_ higher than 7 μM in the *P. falciparum* Dd2_attB strain were discarded to establish the ratio against the *P. falciparum* Dd2_ScDHODH due to the lack of potency which impairs a proper analysis (NC - Not calculated values). To specifically address if they were targeting the mitochondrial ETC by inhibition of the cytochrome bc1 complex, we assessed the susceptibility of *P. falciparum* Dd2_ScDHODH transgenic parasites in the presence of proguanil. It was previously demonstrated that proguanil restores the sensitivity of *Pf*Dd2_ScDHODH to cytochrome bc1 inhibitors (antimycin A, myxothiazol and atovaquone) [43] but not to specific *Pf*DHODH inhibitors (triazolopyrimidines). We observed that the supplementation of 1 μM proguanil was able to restore the susceptibility of *P. falciparum* Dd2-ScDHODH to 5 chemical clusters and 3 singletons, such that their IC_50_s were similar to those of the parental strain *P. falciparum* Dd2-attB (Column N).

**Folate pathway inhibition MoA. (Annotated MOA in Column M, data in Column KO) Gamo group (**data presented first in this paper**), Method:** To identify compounds inhibiting the folate biosynthesis pathway, we utilized the *P. falciparum* clone 3D7A. This strain was adapted to grow in low levels (100 ng/ml) of folic acid (FA) in a depleted para-aminobenzoic acid (pABA) and folinic acid (FA) RPMI media until normal growth rates compared to a *P. falciparum* 3D7A strain in a standard RPMI media were observed. We assessed whether compounds were targeting the Folate pathway comparing the IC_50_s in FA/pABA depleted media and standard RPMI using 3D7A and a 3D7A strain adapted to grow in low levels of FA and pABA. Antifolates are identified due to the IC_50_ shift in the growth medium supplemented with folinic acid, a ratio higher than 10 could suggest that the compound might act through the folate pathway. 3-fold dilutions were used in dose responses assays at concentrations ranging from 10 µM to 0.0002 µM. LDH activity was used as a surrogate of *Plasmodium* viability. Pre-dispensed plates with 50 nl of compounds were inoculated with 25 μL of a parasitized RBC suspension (2% hematocrit, 0.25% parasitaemia) and incubated 72 h. LDH activity was monitored in a final volume of 100 μl of 100 mM Tris-HCl buffer, pH 8.0, containing 0.5 % Tween 20, 100 mM lactate, 100 μM APAD^+^,125 μM NBT (Nitro blue tetrazolium) and 1 U/ml Diaphorase. Absorbance at 650 nm was measured to follow the reaction. The IC_50_ calculations were performed with Grafit 5 software. As shown in Column KO and M, we identified two compounds with a ratio >10 in FA supplemented medium as compared to reduced medium (pABA and FA deficient), suggesting a mode of action via inhibition of the folate biosynthesis pathway. The remaining compounds had no significant effects on this pathway under the conditions tested.

**Possible *Pf*Gwt1 inhibitors** (none found), Horii group (data presented first in this paper). Glycosylphosphatidylinositol (GPI) is a common molecule among eukaryotes to anchor the protein on cell membrane. We had developed a new antifungal E1210 [44, 45], which inhibits fungal GPI biosynthesis by inhibition of the Gwt1 protein, which catalyzes the acylation of inositol in the biosynthesis pathway [46, 47]. GWT1 gene was also found to be conserved in *Plasmodium* protozoa and its inhibitor is expected to be an antimalarial with the novel mode-of-action. In this study, we used four *Saccharomyces cerevisiae* strains that express each GWT1 gene of *Plasmodium* *falciparum* (*Pf*GWT1), *S. cerevisiae* (ScGWT1, lower and higher levels of expression) and human (PIG-W) in place of endogenous ScGWT1, and screened Malaria Box in order to discover new inhibitors of *Pf*Gwt1. YPD broth (Becton, Dickinson and Company) was used for the susceptibility testing of each GWT1-expressing yeast strains. DMSO solutions of compounds were 100-fold diluted with YPD broth containing yeast cells. Inoculum sizes were 2×10^3^ cells/mlml. After 48 h at 30˚C, the turbidity was spectrophotometrically read at 660 nm. Using the reference compound, it was confirmed that IC_50_ of Gwt1p-inhibitor against ScGwt1-overproducer is extremely higher (>80-fold) than that against the ScGwt1p-producer in normal level. As the primary screen, growth inhibition of *Pf*Gwt1-expressing yeast by 400 Malaria Box compounds at a concentration of 20 μM was tested. Forty compounds showed >50% inhibition, and were subjected to the dose-response yeast assay (data not shown) and anti-*Plasmodium* assay (Column O in the heat map). Four of 400 compounds showed high inhibitory activity against *Pf*Gwt1-expressing yeast with IC_50_s of <5 μM. However, each compound inhibited other GWT1-expressing yeasts at the same levels of concentration at which it inhibited *Pf*Gwt1-expressing yeast. Therefore, it seems unlikely that these compounds are *Pf*Gwt1 inhibitors. As a conclusion, no *Pf*Gwt1 inhibitors were found in Malaria Box.

**Delayed Death Phenotype** (none found). Jadhav and Fidock groups (data presented first in this paper). Methods: Malaria Box compounds were tested for delayed death activity, defined as a five-fold or greater reduction in the concentration that produced half-maximal growth inhibition (IC50) value when parasites were exposed to a compound for two generations as opposed to one (*i.e.* 96 h vs. 48 h). Compounds were tested at seven or eight five-fold dilutions, beginning at 29 μM, as described [48]. Azithromycin and artemisinin were included on each plate as positive and negative controls respectively for the delayed death phenotype. Growth inhibition data and IC50 values were derived from the concentration-response curves generated from the luciferase activities measured at the end of the 48 or 96 h period, as described [49]. Assays were conducted with the *P. falciparum* Dd2^attB^/HLH line that expresses a luciferase gene regulated by the *hrp3* promoter and the *hrp2* terminator [50]). This luciferase expression cassette was stably integrated into the *cg6* attB genomic site in these Dd2 parasites [50].

***Pf*ENT-1 Equilibrative Nucleoside Transporter 1** (none found) Akabas group (published in ref [51]), methods: A codon-optimized version of the *P. falciparum* Equilibrative Nucleoside Transporter 1 (*Pf*ENT1, *PF*3D7_1347200) gene was inserted into *Saccharomyces cerevisiae* by homologous recombination to replace the endogenous yeast uridine transporter, FUI1. The resultant *Pf*ENT1-expressing fui1Δ yeast were sensitive to killing by the cytotoxic pyrimidine analog 5-flurouridine (5-FUrd) which entered the cells through *Pf*ENT1. With 125 μM 5-FUrd in the growth media, the yeast would only grow if a *Pf*ENT1 inhibitor was present. Yeast growth was detected by GFP fluorescence and turbidity (OD_600_). The Malaria toolbox compounds were screened at 10 μM in 384 well format plates. None of the compounds displayed any activity in the assay. Screening a 65,000 compound library yielded 171 hits. Nine of the top hits killed *P. falciparum* 3D7 and Dd2 parasites in culture. Thus, the yeast-based assay is capable of successfully identifying *Pf*ENT1 inhibitors. Experimental details were published earlier [51].

**Columns O-Q:** V. Avery group (data presented first in this paper). Asexual Blood Stage Imaging Assay. The asexual HCI assay utilizes the DNA-intercalating dye DAPI (4’, 6’,-diamidino-2-phenylindole) to monitor changes in the parasite number observed within infected erythrocytes. Briefly, compounds are incubated in the presence of 2 or 3% parasitemia (3D7 or K1) and 0.3% hematocrit in a total assay volume of 50 μL, for 72 h at 37°C and 5% CO_2_, in poly-D-lysine coated imaging plates. After incubation, the plates are stained with DAPI in the presence of saponin and Triton X-100 and incubated for a further 5 h at room temperature in the dark before imaging using the Opera™. The digital images obtained from each well are analyzed using spot detection software (Acapella) where fluorescent spots which fulfilled the criteria established for a stained parasite are counted. The percent inhibition of parasite replication is calculated using 0.4% DMSO (0% inhibition) and 2 µM Artemisinin (100% inhibition) control data. The HCI assay was developed in 384-well format for use on the Opera™ confocal imaging system with Twister ARM, and is capable of screening over 70,000 assay wells (200 plates) per day. The assay typically yields Z’-factor values ≥0.6, with signal-to noise ratios ≥ 10:1 [52]. Reference compound activities for 3D7 and K1 strains of *P. falciparum*: chloroquine 3nM 3D7, 199nM K1; pyronaridine 3nM 3D7, 5nM K1; artemisinin 1nM 3D7, 1nM K1; epoxomicin 1nM 3D7, 2nM K1; and, pyrimethamine 2nM 3D7, 7200nM K1.

**Column S:** Horrocks group (data presented first in this paper). Asexual Dd2 inhibition of growth (IC50s): IC50 for the Dd2 clone of *Plasmodium falciparum* were determined using the Malaria Sybr Green I Fluorescence assay [47]. Drug assays were initiated using a 2% trophozoite stage parasitaemia and 2% hematocrit and fluorescence measured after 48 h as fully described in Hasenkamp *et al*.[53]. Log dose-response curves were plotted in GraphPad Prism (v5.0). The controls for Dd2 using our 48hr Sybr Green I assay were (rounded to nearest nM): Chloroquine 209nM, Mefloquine 11nM, Quinine 246nM, Dihydroartemisinin 4nM, Artemether 10nM, Atovaquone 3nM.

**Column T:** Horii group (data presented first in this paper). IC_50_ against asexual blood stage *P. falciparum* FCR3. Methods: For measurement of anti-*Plasmodium* activities, *P. falciparum* FCR3 strain was cultured in the RPMI 1640 medium supplemented with 10% human type A serum from in-house volunteers at 37°C, 5% O_2_ and 5% CO_2_ (0.5% parasitemia and 2% hematocrit) for 72 h in the presence of the compounds (DMSO final concentration, 0.25%). The growth inhibition of *P. falciparum* was determined using a SYBR Green I assay [54]. Lysis buffer (20 mM Tris (pH 7.5), 5 mM EDTA, 0.008% (w/v) saponin and 0.08% (v/v) Triton X-100) containing SYBR Green I was added to each well and incubated at room temperature and dark condition for 1-5 hours. After the incubation, amounts of parasite DNA were measured as fluorescence at 485 nm of excitation and 530 nm of emission. An unpublished antimalarial compound was used as positive control in the assay. In many of the repetions, chloroquine and artemisinin were tested, and showed IC_50_s of 125 and 47 nM, respectively.

**Columns U-AF:** Ayong group (data presented first in this paper). Asexual activity and stage-specificity of Malaria Box compounds. The antimalarial activity of all 400 Malaria Box compounds were assessed against six laboratory strains of *P. falciparum* by using a novel image acquisition and data mining system for quantifying culture parasitaemia and parasite stage proportions in 384-well plate formats [55, 56]. Parasitological EC_50_s were determined from plots of drug concentrations (2-fold dilutions in DMSO, 10 µM down to 0.31nM) against 72-hour post-treatment parasitaemia, whereas the effect of each compound on life cycle progression was determined by quantifying the proportion of each distinguishable parasite stage (early rings, late rings, trophozoites and schizonts) in culture following 36-hour treatment with compounds at maximum effect concentrations of 10 µM, and at four distinct post-invasion time-points (6, 18, 30 and 42 hpi). Drug effects on parasite mitochondrial integrity was also investigated by measuring the proportion of Mitotracker-positive parasites in treated wells relative to those in solvent control wells [52]. The reference drug controls included 1) Artemisinin, used at 2-fold dilutions (400nM down to 0.012nM) in all dose-response assay plates, and 2) Artemisinin (ART), Chloroquine (CQ), trans-Epoxysuccinyl-L-leucylamido-(4-guanidino) butane(E64), and N-acetylglucosamine (GlcNAC), each used at 10 µM maximum effect concentrations for studies of drug effects on asexual life cycle progression. The EC_50_ values were determined using a nonlinear regression curve-fitting method with a variable slope sigmoidal dose-response option. Compounds were considered active against a parasite stage if the treatment resulted in a stage accumulation index (stage proportion in test well relative to the same stage proportion in solvent control wells) greater than 1.5. Compounds were regarded as having an anti-merozoite invasion effect if treatment at the 42-hpi time-point resulted in significant reduction in total parasitaemia with no significant accumulation in schizont stage parasites. Meanwhile, compounds were selected as having an effect on parasite mitochondrial integrity if the treatment resulted in more than 50 per cent Mitotracker-negative parasites amongst the infected red blood cell population.

Results with Controls:

1) ART EC_50_ values: 3D7: 11.7+/-1.4nM, HB3: 8.6+/-1.2nM, Dd2: 5.3+/-1.2nM, W2: 5.7+/-0.5nM, K1: 3.5+/-0.4nM, FCR3: 5.0+/-0.4nM.

2) Effects on asexual life cycle progression following treatment at ER, LR, T, S). Last two columns represent effects on merozoites and on mitochondrial integrity, respectively: ART: LR-X-S-X-o-o, CQ: LR-X-S-o-o-o, E64: X-S-S-X-o-o, GlcNAC: LR-o-o-o-X-o.

NB: Symbols are as also defined in Suppl S3, considering the parasite stage with the highest positive stage accumulation index at each treatment timepoint,

**Columns AG-AJ:** Poulsen/Andrews/Tonissen groups (data presented first in this paper). Differential inhibition under hypoxic or reduced bicarbonate conditions. Methods: *P. falciparum* growth inhibition assays (GIAs) were carried out with MMV Malaria Box Plate A compounds using an isotopic [^3^H]-hypoxanthine incorporation assay, as previously described [57]. Briefly, synchronous ring-stage *P. falciparum* 3D7 (sensitive to chloroquine and other antimalarial drugs) [58] infected erythrocytes at 0.25% parasitemia and 2.5% hematocrit were seeded into 96 well tissue culture plates (Corning, USA), in duplicate wells, with and without test compounds in an 8 point dose response. Vehicle only (0.5% DMSO) and the antimalarial compound chloroquine (Sigma) were used as negative and positive controls, respectively (Tables 1-2 below). After incubation for 48 h under standard (or modified, as specified) *P. falciparum* culture conditions, [^3^H]-hypoxanthine was added (0.5 µCi per well) and plates incubated for a further 24 h. Cells were harvested onto 1450 MicroBeta filter mats (Wallac) and [^3^H] incorporation determined using a 1450 MicroBeta Trilux scintillation counter (Perkin Elmer). Percentage inhibition of growth compared to DMSO controls was determined and IC_50_ values calculated using linear interpolation of inhibition curves [59]. Statistical analysis of differences in IC_50_ values was determined using a two-tailed student’s t-test. For hypoxia experiments, cultures were maintained in a specialized hypoxic C-Chamber (Biospherix, New York, USA). Plate A compounds were grouped based on MMV reported IC_50_s to guide assay concentration range for GIA dose-response. Two different growth conditions were assessed, HCO_3_^-^ modified culture and O_2_ modified culture. (A) *HCO_3_^-^ Modified culture (columns AG and AH):* GIAs were carried out under standard culture conditions (37°C in 5% O_2_, 5% CO_2_, 90% N_2_) using O-positive human erythrocytes (A gift from the Australian Red Cross Blood Service) in RPMI 1640 media (Life Technologies; cat # 23400-013) supplemented with 10% heat-inactivated human serum (A gift from the Australian Red Cross Blood Service) and gentamycin (5µg/ml). Media with (HeatMap. supplementary data S1: column AG) and without added HCO_3_^-^ [2 g/L; (HeatMap, supplementary data S1: column AH)] were compared. The average pH of normal media was 7.49 (range 7.02 to 8.13) while the average pH of HCO_3_^-^ reduced media was 7.12 (range 6.98 to 7.38). IC_50_s were determined for two to three independent experiments, each carried out in duplicate (eight point dose-response). (B) O_2_ *Modified culture:* GIAs were carried out at 37^o^ C in RPMI 1640 media (Life Technologies; cat #11875-093) supplemented with 10% heat inactivated human serum and gentamycin (5µg/ml) under normal gas conditions (5% O_2_, 5% CO_2_ in N_2_) or reduced oxygen (1% O_2_, 5% CO_2_ in N_2_). IC_50_s were determined for two independent experiments, each carried out in duplicate (eight point dose-response). Summary of assay findings: (*i*) Two MMV compounds were not tested owing to solubility issues (MMV085203 and MMV665874). (*ii*) 11 MMV compounds had no effect in the GIAs over the concentration range selected. (*iii*) Eight MMV compounds exhibited >2-fold differences in IC_50_s in standard versus HCO_3_^-^ reduced media, with four of these compounds having a significant difference (P<0.05; Table 1). All eight compounds had reduced potency in HCO_3_^-^ depleted media. (*iv*) Chloroquine exhibited a >2-fold difference in IC_50_s in standard versus HCO_3_^-^ reduced media (P<0.05). (*v*) Ten MMV compounds exhibited >2-fold differences in IC_50_s in standard 5% O_2_ versus 1% O_2_ media, with three of these compounds having a significant difference (P<0.05; Table 2). All ten compounds had increased potency in 1% O_2_. (*vi*) Two MMV compounds exhibited >2-fold differences in IC_50_s in both HCO_3_^-^ reduced media and in 1% O_2_ (Table 3). (*vii*) Only compounds with a significant difference from the control cultures are marked in the Heat Sheet. These are also noted in column G as a possible respiratory target because of differences in IC_50_ in low oxygen and/or reduced bicarbonate (Poulsen/Andrews/Tonissen groups, columns AG-AJ).

**Table 1 (Poulsen/Andrews/Tonissen groups) (**data presented first in this paper)**.** Eight Malaria Box compounds and chloroquine exhibited >2-fold difference in IC_50_s in standard versus HCO_3_^-^ reduced media. (*n=2; **n=3, n.s.d. - not statistically different)

| **COMPOUND_ID** | **COMPOUND STRUCTURE** | ***P. falciparum* 3D7** IC_50_ **(**μM) | | **fold change**  **(**IC_50_**reduced/**IC_50_**normal)** | **P value** |
| --- | --- | --- | --- | --- | --- |
|  |  | **HCO_3_^-^ (2g/L)** | **HCO_3_^-^ reduced** |  |  |
| MMV011259** |  | 0.059 | 0.132 | 2.2 | 0.045 |
| MMV396672* |  | 0.425 | 1.121 | 2.6 | n.s.d. |
| MMV665915* |  | 0.192 | 0.464 | 2.4 | n.s.d. |
| MMV000448* |  | 0.19 | 0.594 | 3.1 | n.s.d. |
| MMV020500** |  | 0.04 | 0.155 | 3.9 | 0.004 |
| MMV006172** |  | 0.098 | 0.269 | 2.7 | n.s.d. |
| MMV006087** |  | 0.046 | 0.094 | 2.0 | 0.001 |
| MMV396703* |  | 0.326 | 0.851 | 2.6 | n.s.d. |
| Chloroquine |  | 0.016 | 0.036 | 2.3 | 0.015 |

**Table 2 (Poulsen/Andrews/Tonissen groups) (**data presented first in this paper**).** Ten Malaria Box compounds exhibited >2-fold differences in IC_50_s in standard 5% O_2_ versus 1% O_2_ media (n=2 assays, n.s.d. - not statistically different).

| **COMPOUND_ID** | **COMPOUND STRUCTURE** | ***P. falciparum* 3D7** IC_50_ **(µM)** | | **fold change**  **(**IC_50_**normal/** IC_50_**hypoxia)** | **P value** |
| --- | --- | --- | --- | --- | --- |
|  |  | **5% O_2_** | **1% O_2_** |  |  |
| MMV019406 |  | 0.54 | 0.236 | 2.3 | n.s.d. |
| MMV008416 |  | 0.397 | 0.065 | 6.1 | n.s.d. |
| MMV006203 |  | 0.489 | 0.132 | 3.7 | n.s.d. |
| MMV665977 |  | 0.345 | 0.085 | 4.1 | 0.022 |
| MMV000448 |  | 0.36 | 0.059 | 6.1 | n.s.d. |
| MMV020788 |  | 0.136 | 0.063 | 2.2 | n.s.d. |
| MMV006087 |  | 0.04 | 0.018 | 2.2 | 0.003 |
| MMV665782 |  | 0.019 | 0.009 | 2.1 | n.s.d. |
| MMV665876 |  | 0.415 | 0.066 | 6.3 | n.s.d. |
| MMV011256 |  | 0.349 | 0.07 | 5.0 | 0.010 |
| Chloroquine |  | 0.011 | 0.007 | 1.6 | n.s.d. |

**Table 3 (Poulsen/Andrews/Tonissen groups) (**data presented first in this paper)**.** Two Malaria Box compounds exhibited >2-fold differences in IC_50_s in both HCO_3_^-^ depleted media and in 1% O_2_.

|  | ***P. falciparum* 3D7** IC_50_ **(µM)** | | **fold change**  **(**IC_50_**reduced/** IC_50_**normal)** | ***P. falciparum* 3D7** IC_50_ **(µM)** | | **fold change**  **(**IC_50_**normal/** IC_50_**hypoxia)** |
| --- | --- | --- | --- | --- | --- | --- |
| **COMPOUND_ID** | **HCO_3_^-^ (2g/L)** | **HCO_3_^-^ reduced** |  | **5% O_2_** | IC_50_ **hypoxia 1% O_2_** |  |
| MMV000448 | 0.19 | 0.594 | 3.1 | 0.36 | 0.059 | 6.1 |
| MMV006087 | 0.046 | 0.094 | 2.0 | 0.04 | 0.018 | 2.2 |

**Column AK:** DeRisi group (published in ref [60]). Shift of inhibition by IPP addition. Methods: A 96-well plate assay format was used to grow parallel 200 µL cultures of *P. falciparum* W2 cultures (MRA-157 from MR4), with and without IPP supplementation (200 µM). Parasites were grown in human erythrocytes (0.5% hematocrit) in RPMI 1640 media supplemented with 0.25% Albumax II (GIBCO Life Technologies), 2 g/L sodium bicarbonate, 0.1 mM hypoxanthine, 25 mM HEPES (pH 7.4), and 50 μg/L gentamicin, at 37 °C, 5% O2, and 5% CO_2_. Cultures were treated with 5 µM Malaria Box compound, along with “no drug” and “no parasite” controls. The parasites were maintained for 72 h, growth was terminated by fixation with 1 % formaldehyde, and parasitized cells were stained with 50 nM YOYO-1 (Invitrogen). Parasitemia was determined by flow cytometry using a BD LSRII. Data was analyzed using FlowJo and EC_50_ curves plotted by GraphPad Prism. Compounds that “rescue” upon IPP supplementation target the apicoplast or apicoplast-related pathways, whereas compounds that do not target the apicoplast do not display the rescue phenotype upon IPP supplementation. MMV08138 showed the highest level of rescue by IPP supplementation and was later determined to inhibit *Pf*-IspD of the apicoplast-localized isoprenoid biosynthesis pathway [60].

**Columns AL and BM-BN:** Cassera group (published in ref [61]). Apicoplast function analysis and gametocyte screen. Method: We aimed to identify a potential anti-apicoplast drug candidate among the compounds of the Malaria Box using reversal of growth inhibition by isopentenyl diphosphate (IPP) supplementation. Fosmidomycin was used as positive control. We identified one drug-like compound (MMV008138) that targets the apicoplast [61], and further medicinal chemistry has been applied to this scaffold in order to improve its antimalarial activity [62]. Further analyses revealed that the mechanism of action of this compound was more similar to that of fosmidomycin than antibiotics such as doxycycline suggesting the isoprenoid biosynthesis as potential target [61]. In addition, we investigated whether the compounds from the Malaria Box were active in killing late-stage (stage V) gametocytes [61], using the Alamar Blue viability assay and epoxomicin as positive control as described previously [63]. Under the assay conditions used, MMV000248 and MMV006172 were the most active compounds with IC_50_s of <0.35 μM against late-stage gametocytes [61].

**Columns AN-AR:** Winzeler group [64]. *P. berghei* liver stage assay. *P. berghei* Luciferase sporozoites from MR4 (MRA-868 [65] were obtained from dissected infected *Anopheles stephensi* mosquito salivary glands. 3x10^3^ HepG2-A16-CD81EGFP cells (originally, HepG2 or human hepatocellular carcinoma cell line HepG2, were obtained D. Mazier, INSERM U511 Immunobiologie Cellulaire et Moléculaire des Infections Parasitaires, CHU Pitié-Salpêtrière, Université Pierre et Marie Curie, Paris, France) [66] in 5 μl of supplemented DMEM medium (2x10^5^ cells/ml, 5% FBS, 5x Pen/Strep/Glu) were seeded in 1,536-well plates (Greiner BioOne white solid bottom) 20 h prior to the actual infection. 18 hours prior to infection, 50 nl of compound in DMSO (0.5% final DMSO concentration per well) were transferred with a PinTool (V&P Scientific, Inc) into the assay plates. Atovaquone, puromycin and 0.5% DMSO were used as positive and negative controls. The HepG2 cells were then infected with 750 sporozoites per well (5 μl) with a single tip Bottle Valve liquid handler (GNF Systems). The plates were spun down at 37 °C for 3 minutes in an Eppendorf 5810 R centrifuge with a centrifugal force of 330xg on lowest acceleration and brake setting. The plates were then incubated at 37 °C for 48 hours in 5% CO_2_ with high humidity to minimize media evaporation and edge effect. After incubation, the parasite EEF growth was quantified by bioluminescence measurement. Media was removed from the plates and 2 μl BrightGlo (Promega) were dispensed with the MicroFlo (BioTek) liquid handler. Immediately after addition of the luminescence reagent, the plates were read by an EnVision Multilabel Plate Reader (PerkinElmer). HepG2 compound toxicity was assessed in separate plates that were not infected with sporozoites but otherwise treated the same way except that 2 μl CellTiterGlo (Promega - diluted 1:2 with deionised water) per well were added at the end with the MicroFlo (BioTek) liquid handler. Immediately after addition of the luminescence reagent, the plates were read by an EnVision Multilabel Reader (PerkinElmer). The last column shows a summary of the *P. berghei* liver stage activity at 50 μM, with breakpoints being: tox (toxic) if >50% HepG2, weak if >10% liver stage growth and not toxic (tox), active with <10% liver stage growth and not tox, NA not active. IC_50_ values were obtained using measured bioluminescence intensity and a non-linear variable slope four parameter regression curve fitting model in Prism 6 (GraphPad Software Inc).

**Columns AS-AT:** Hanson group (data presented first in this paper). *P. berghei* liver stage growth. Phenotypic analysis of Malaria Box compound activity against *P. berghei* liver stages and assay development will be reported elsewhere (Medina Vera, Itoe, *et al*; in preparation). Methods: Screening was carried out in 96 well plates, with 10,000 HepG2 cells (ATCC) seeded one day prior to infection with 10,000 *P. berghei*-Luciferase (676m1cl1) sporozoites per well in the presence of 1:300 Fungizone (Life Technologies). Medium was replaced 2 hours post-infection (hpi) and the Malaria Box compounds were added at a final concentration of 5 μM at this time. At 48 hpi, parasite load, by way of luminescence, was quantified after a 10 minute incubation with 50 μl of 150 μg/ml D-Luciferin on a Tecan Infinite M200 plate reader. Plates included 8 wells of DMSO (compound vehicle and positive control for infection), along with 8 non-infected wells serving as negative controls. Luminescence values for individual wells were normalized to the mean of the in-plate DMSO controls, which was set at 100. Over the entire Malaria Box screen, the standard deviation for the normalized positive control was 12.8. The normalized mean of non-infected control wells was 2.5, with a standard deviation of 2. Data presented are the average of 3 independent experiments, each conducted in technical triplicate. Toxicity of the compounds was determined at 66 hpi based on HepG2 cell abundance in 9 non-overlapping images per well, generated with a Molecular Devices IXM using a 40x objective. Briefly, EEFs were identified based on 2E6 immunolabeling and used to mask widefield images of Hoechst 33452-labeled nuclei, and the percentage of each 2,160 x 2,160 pixel image occupied by Hoechst^+^ pixels, representing HepG2 nuclei only, was then determined for all images in CellProfiler. Data is from one representative experiment. The mean area occupied by HepG2 nuclei in the 8 DMSO controls wells was set to 1, and used to normalize those treated with compounds.

**Columns AV-AY & BK-BL:** V. Avery/Fidock groups (publ in ref [67-69]. Early and Late Stage Gametocytocidal Luciferase Assays. *P. falciparum* NF54^Pfs16^ parasites, expressing GFP (Green Fluorescent Protein)-luciferase under the control of the *psf16* promoter were used [70]. Gametocytogenesis was induced as previously described [67]. Early Stage Gametocyte Luciferase Assay: MMV Malaria Box compounds were screened at 5, 2.5 and 0.5 µM in 384-wells white luminescence plates (Culturplate, PerkinElmer). Stage I gametocytes on day 1 of gametocytogenesis, obtained by a standardized induction protocol were seeded at 10% parasitemia and 0.5% hematocrit in a final volume of 50 µl/well and exposed to the compounds (0.4% final DMSO concentration) for 72h. At the end of incubation, on day 4 of gametocytogenesis, 25 µl medium was replaced with 15 µl Steadylite plus (PerkinElmer). Luminescence was measured after 1 hr incubation at room temperature using a MicroBeta Trilux (PerkinElmer) multidetector luminometer. Raw luminescence data were normalized by detector efficiency and by the signal window given by the in-plate positive (5 µM puromycin) and negative (0.4% DMSO) controls. Hits were selected using a 50% inhibition threshold at 5 µM. Inhibition data from dose-response experiments were fitted with 4 parameter nonlinear regression in GraphPad Prism v. 5.0, to obtain IC_50_ values [67]. A panel of 25 antimalarial compounds was tested for the validation of the assay. Examples of early stage gametocytocidal activities of reference antimalarials include (rounded to the nearest nM): artemisinin 13 nM; methylene blue 30 nM; chloroquine 77 nM. Late Stage Gametocyte Luciferase Assay: Stage IV gametocytes were magnetically purified on day 8 of gametocytogenesis and used at 0.1% hct and 10% gametocytemia. Gametocytes were incubated with MMV Malaria Box compounds at 5 µM for 72h. Luminescence readout was carried out as described above on day 11 of gametocytogenesis [69]. A panel of 39 antimalarial compounds was tested for the validation of the assay. Examples of late stage gametocytocidal activities of reference antimalarials include (rounded to the nearest nM): artemisinin 26 nM; methylene blue 38 nM; chloroquine did not inhibit late stage gametocytes up to 10 µM.

**Columns AZ-BB & BH-BJ:** V Avery Group (published in ref [71])**:** The early and late gametocyte imaging assays utilized two fluorophores to define a viable gametocyte. For the HTS assay, transgenic parasites expressing GFP associated with a marker of gametocytogenesis (*Pf*s16) were used to define the gametocyte, plus a specific mitochondrial dye was included which indicates a viable, respiring gametocyte.

Essentially, transgenic *Plasmodium falciparum* NF54 parasites which expressed a GFP-*Pf*s16 marker were induced to undergo gametocytogenesis. The parasites were cultured through development from early- to late-stage / mature gametocytes.

Early gametocyte (stage I-III) assay protocol (ESG): Five µl of diluted compound in 4% DMSO was transferred in 384 well format into PerkinElmer Cell carrier Poly-D-Lysine imaging plates using a MinitrackTM liquid handler. The day 2 isolated gametocytes (ESG) prepared at 10% parasitaemia (P) and 0.1% hematocrit (H) were dispensed in 45 µl volumes into imaging plates using a multidrop dispensing instrument. The plates were sealed with gas-permeable membranes and incubated for 72 h in 5% CO_2_, 5% O_2_ and 60% relative humidity at 37° C. After 72 h incubation, 5 µl of 1/750 diluted reduced MitoTracker Red CM-H2XRos (MTR) was added to each well, plates were resealed with membranes and incubated overnight. The following day, the plates were brought to room temperature for at least 1 h before being measured on the Opera QEHS Imager, utilizing a Twister II microplate handler for automated plate feeding.

Late gametocyte (stage IV-V) assay protocol (LSG)/ Mature gametocyte assay (MG) [71]: Highly synchronous gametocytes were harvested by magnetic purification on day 8 post gametocytogenesis induction when demonstrating a typical stage IV morphology (LSG). For the mature gametocyte (MG) assay, day 12 gametocytes were used instead of the day 8 gametocytes. The gametocytes were diluted to 33,000 per well at 0.1% H. This number of LSG per well is comparable to the calculated number of asexual assay parasites used in the 3D7 and Dd2 assays so a direct comparison can be applied for activities. Gametocytes were incubated with the test compounds for a period of 72 h, then a mitochondrial viability marker (MitoTracker Red CM-H2XRos (MTR)) was added and the plates imaged using confocal microscopy. Based on GFP fluorescence intensity and morphology (elongated), LSG and MG were identified and their viability demonstrated by co-localisation of the MTR associated fluorescence intensity. Please see below for data analysis. The number of viable LSG / MG per well was calculated and used to determine the % inhibition of each individual compound in relation to 0.4% DMSO and 5 µM Puromycin, as the negative and positive controls, respectively. . The activity of 36 anti-malarial and candidate anti-malarial drugs were also published in ref [71]. Three examples of activity (early:late) are Artemisinin 1:5nM, methylene blue 280:287nM and pyrimethamine demonstrated no activity at 40uM.

**Columns BC-BG**: Winzeler group [72]. Gametocyte assay at UCSD: Stage-specific gametocyte cultures of a clonal strain of NF54 were prepared as previously described [73] with some modifications. At each given stage 20µl of gametocyte culture in complete media with N-Acetyl-D-Glucosamine for stages I-IV at 0.5-0.75% gametocytemia was dispensed into compound-loaded black, clear bottom 384-well plates (Greiner) at a final compound concentration of 12.5 µM, a total volume of 40µl and a final hematocrit of 1.25% and incubated at 37ºC for 72 hours under low oxygen conditions. DMSO was used as negative control, Puromycin as positive control. 5 µl from a 5 µM solution of the dye MitoTracker® Red CMXRos (Life Technologies) in screening media was dispensed into the assay plate and incubated for 20 minutes at 37°C. Into a new 384-well imaging plate 40 µl from a 500 nM solution of MitoTracker Red at 37° C in screening was dispensed into the wells at 37ºC. Then, using a 16 channel 12.5 µl matrix pipette, 5 µl was transferred from the assay plate to the imaging plate to form a monolayer. After another incubation of 30 minutes at 37ºC the plates were imaged using an Operetta high content imaging system (Operetta R, Perkin Elmer) at room temperature with a 20x objective and an excitation/emission filter of 560-580nm/ 590-640nm. The gametocytocidal activity was determined using a high content image analysis software (Harmony, Perkin Elmer) by identification of number of red fluorescent gametocytes in each well. The gametocyte count was normalized based on the number of gametocytes in the DMSO wells.

**Columns BO-BR:** Taramelli group (Columns BO-BP data presented first in this paper, Columns BQ-BR are new data except as noted below). Gametocytocidal activity at UMIL. The Malaria Box compounds were used for primary screening against *P. falciparum* 3D7 gametocytes and subsequently for IC_50_ determination against *Pf* gametocytes obtained from the 3D7 and the 3D7elo1-pfs16-CBG99 transgenic strain [74], as previously described [75]. The primary screening was performed on stage IV-V gametocytes from the 3D7 strain at 3.7 µM, in 96 well plates, using 100 nM epoxomicin and 1 µM DHA as positive controls and 0.5% DMSO as negative untreated control. Gametocyte viability was measured by the pLDH assay at both 72h (column BO in Heat Map) and 144 h with drug removal after the first 72h (72+72h, column BP in HeatMap) [75]. Based on the primary screening, 36 compounds with activity higher than 50% at 72+72h were selected for IC_50_ determination. The dose-response experiments were performed in 96 well plates using the 3D7 strain (pLDH, 72+72h, column BR in heat map) and the 3D7elo1-pfs16-CBG99 (luciferase assay, 72h, column BQ in heat map) [74]. Data analysis was performed as described [76].

**The IC_50_ for the following compounds (n= 25) of columns BQ/BR are new/data presented first in this paper:**

MMV compound ID (raw number)

MMV000760 (178)

MMV000787 (179)

MMV000788 (180)

MMV001246 (111)

MMV006787 (28)

MMV007092 (130)

MMV007907 (120)

MMV019555 (307)

MMV019690 (150)

MMV019780 (278)

MMV019995 (279)

MMV020505 (256)

MMV073843 (367)

MMV396794 (329)

MMV403679 (368)

MMV665814 (194)

MMV665878 (229)

MMV665902 (291)

MMV665969 (187)

MMV666026 (339)

MMV666054 (189)

MMV666079 (13)

MMV666080 (190)

MMV666095 (172)

MMV666687 (309)

**Column BS:** Mancama group (data presented first in this paper). Gametocytocidal activity. Methods: Induction of gametocytogenesis and maintenance of gametocyte cultures. This method was adapted from Carter *et. al.* [77] and the precise method is described [78]. Gametocytogenesis was induced by a combination of nutrient starvation and a drop in hematocrit. *P. falciparum* strain NF54 asexual parasites were cultured in 10% serum-rich media to a 6–10 % parasitaemia, which was then decreased to 0.5 % (at 6 % hematocrit) and the culture transferred to glucose-free medium. Cultures were maintained in an atmosphere of 5 % CO_2_,5%O2 and 90 % N2, at 37 °C, without shaking. Cultures were also kept at 37 °C during daily medium changes. After 72 h, the hematocrit was dropped to 3 % (day 0). Gametocytogenesis was subsequently monitored microscopically with daily medium (glucose-free) changes. On days 6–9, residual asexual parasites were eliminated by continuous 50 mM N-acetyl glucosamine (NAG), Sigma-Aldrich) treatment, now in the presence of 0.2 % glucose. Glucose enrichment was maintained from day 10 onwards and gametocytes monitored daily by microscopy until they were predominantly stage V and were used in the various assays. Resazurin-based dye assay: The PrestoBlue® (Life Technologies) assay was based on an adaptation of the method described by Tanaka and colleagues [63, 79]. Drug dilutions were placed in triplicate in 96-well plates in a volume of 50 μl/well. Semi-synchronous gametocyte cultures (50 μl/well, stage IV/V) were added to the 96-well plates to achieve a final gametocytaemia and hematocrit of 2 % and 5 % respectively, in a total incubation volume of 100 μl. The plate was encased in an airtight chamber and gassed for 5 min with a 5 % CO_2_,5% O_2_, balance N_2_ mixture (Afrox, Johannesburg, South Africa). Following incubation at 37 °C for 48 h, 10 μl of PrestoBlue® reagent was added to each well and left to incubate at 37 °C for 2 h. Finally, the plate was centrifuged at 120xg (1 min), and 70 μl of the supernatant transferred to a clean 96-well plate before reading in a multi-well spectrophotometer (Infinite F500, Tecan, USA) by fluorescence detection at 612 nm. A detailed narrative describing the validation of this assay is provided in Reader and colleagues [78]. For the data presented in this work, dihydroartemisinin (DHA) was used as the reference control compound based on the validation criteria described in [78]. DHA inhibition (84% (+/- 0.81%)) of stage IV/V gametocytes was in line with the activity previously described for this compound using PrestoBlue® (Life Technologies) [29]. The average standard deviation (SD) across the dataset was +/- 1.09% (min 0.0%; max 5.5%).

**Columns BT & BU:** Tripathi group (Published in ref [80]). Gametocytocidal assay We developed a robust gametocytocidal assay utilizing the nucleic acid dye SYBR Green I in conjunction with a background suppressor of green fluorescence from CyQUANT to preferentially measure live gametocytes after drug treatment. Signal to noise ratio was improved by increasing the male gametocyte DNA content through exflagellation in addition to the quenching of dead gametocyte signal with the background suppressor. We used *P. falciparum* NF54 for all the assays, which included five steps: 1) Culture, enrich and plate gametocytes. 2) Incubate gametocytes with drug for 48 hr. 3) Add exflagellation media and incubate 30 min. 4) Add SYBR Green I and background suppressor and incubate 2 hr. 5) Read SYBR Green I fluorescence at excitation 485 nm and emission 535 nm [80]. The MMV Malaria Box was screened with our SYBR Green I/CyQUANT suppressor assay at 10 µM using pyrvinium pamoate as our 100% killing control. Eighteen compounds with greater than 80% inhibition were identified, seventeen of which had IC_50_s less than 10 µM. The mean Z-factor calculated for the SYBR green/CyQUANT assay in this screen was 0.57. Ten of the top hits were probe-like and eight were drug-like in nature. In the Malaria Box data summarized here our top hits were generally highly conserved among the other gametocytocidal assays including those using luciferase, resazurin, LDH and Mitotracker red. Furthermore many of the top hits in the SYBR Green/CyQUANT assay also demonstrated activity against later *Plasmodium* stages including gamete and ookinete formation. Based on the various assays, we found several of our top hits were male-specific and also active against both early and late stage gametocytes, though at least one compound (665941) was female specific, confirming that our assay detects killing of both male and female gametocytes. All hits showed activity against other microorganisms, including other apicomplexans and flagellates such as *Babesia sp., Neospora, Trypanosoma brucei and T. cruzi,* as well as blood nematodes (*Brugia malayi)* and blood trematodes (*Schistosoma)*, suggesting the targets of these compounds may be conserved. Further pursuit of compounds targeting various pathogens may lead to development of broad-spectrum anti-parasitic or anti-microbial agents.

**Columns BV & BW:** V. Avery/Alano groups, Acridine Orange Image Based Gamete Assay (published [81]): *P. falciparum* 3D7A mature stage V gametocytes on day 12 of gametocytogenesis were seeded in pre-warmed 384 black clear-bottom imaging plates (Viewplate, PerkinElmer) to a final volume of 50 µl and 0.1% hematocrit (~40,000 gametocytes per well) and immediately returned to standard incubation conditions (37°C; 5% O2, 5% CO_2_ and 90% N_2_). In each assay plate 5 μM CMPD-2 and 0.4% DMSO were used as positive and negative controls, respectively. After 48 hours incubation, plates were brought to room temperature (22.7 ± 0.3°C) and the gametocytes exposed to 40 µM XA and 60 nM Acridine Orange. Plates were imaged after 2.5 hours light-protected incubation at room temperature on an Opera QEHS micro-plate confocal imaging system (PerkinElmer) using a 20x water immersion objective using 488 nm excitation and 520/535 nm emission, and an exposure time of 280 - 400 msec. A custom script based on the spot detection algorithm was developed using the high-volume image data storage and analysis system Columbus 2.5 (PerkinElmer). Lucantoni L, Silvestrini F, Signore M, Elderingname M, Dechering KJ, Avery VM, Alano P., (Publ http://www.ncbi.nlm.nih.gov/pubmed/26553647)).

**Columns BX- CA, CD-DF:** Delves, Ruecker, & Sinden group (Data and methods published in ref [82] except Column CE which is new data) The data represent our primary screening data in the *P. falciparum* Dual Gamete formation assay (*Pf* DGFA). They show the functional viability of mature Stage V male and female gametocytes as reported by their ability to undergo gamete formation as reported in the reference. **Column CD** represents primary screening data in the *P. berghei* Ookinete Development Assay (Pb ODA) at 1 µM as reported in the reference. **Column CE** represents a parallel screen in the Pb ODA at 10 µM that has not been published. **Column CF** represents the EC_50_s generated for selected compounds in the Pb ODA as reported in the reference except for the below compounds which represent new data.

| Compound position (row) in heat map | MMV Code |
| --- | --- |
| 40 | MMV000304 |
| 41 | MMV006172 |
| 84 | MMV665831 |
| 96 | MMV665886 |
| 107 | MMV665805 |
| 208 | MMV665850 |
| 306 | MMV019881 |
| 316 | MMV009127 |
| 318 | MMV006309 |
| 322 | MMV666693 |
| 360 | MMV000563 |
| 389 | MMV019266 |

**Column CB:** Vinetz group (data presented first in this paper). *Plasmodium falciparum* strain NF54 was maintained in continuous asexual culture according to standard protocol [83]. Gametocytes were cultured as previously described [84]. The original compounds (10 µl of each compound with 10 mM concentrations in DMSO) in the MMV Box were serially diluted, prepared as 50 μM aliquots and stored at -20^0^C. Multiple flasks of gametocyte cultures (14-18 days) were pooled for the assay to which small batches of compounds and controls were added, in duplicate. Briefly, pooled culture (200 µl) was placed into pre-warmed (37^0^C) microfuge tubes, quickly microcentrifuged (500 *g* for 1 min at 37^0^C), and supernatants removed and replaced with 100 µl of exflagellation solution in gametocyte culture medium containing MMV Box compounds (final concentration of compound and DMSO were 1 µM and 0.01%, respectively). As a negative control, exflagellation solution containing the same amount of DMSO (0.01%) in the absence of MMV Box was used; two sets of control tests were carried out (one at the beginning and other at end of sample addition) per batch. After 15 min incubation at room temperature, exflagellation centers were quantified under 400X-magnification light microscopy. Exflagellation centers of male gametocytes were defined as having distinct features (e.g., rapidly dancing-like movements due to the whipping motion of the flagella and also adherence to neighboring erythrocytes) as visualized in negative control experiments (0.01% DMSO) at the beginning and end of each batch of compound screening. Values obtained by the number of exflagellation centers observed in the test divided by negative control (full exflagellation is 1.0, reductions show as <1.0 and enhanced is >1.0) are presented in the tables. Differences in number of exflagellation centers were analyzed by Student’s t test (parametric test) using GraphPad Prism Software (GraphPad Software Inc, La Jolla, CA). Statistical significance of differences between test and negative controls was taken as *p* < 0.01; those with non-significant changes in exflagellation are marked as NS (not significant).

**Columns CH & CI:** Huston group (column CH published in ref [85] and CI in preparation for submission). *Cryptosporidium parvum* screening at 2.3 µM and IC_50_s. The Malaria Box compounds were screened for anti-*Cryptosporidium parvum* activity using an established cell-based high content microscopy assay [85, 86]. *C. parvum* oocysts (Iowa strain) (Bunch Grass Farms, Deary, ID) stored in phosphate-buffered saline (PBS) with 1,000 IU/ml penicillin and 1 mg/ml streptomycin were prepared for use by treatment with 10 mM HCl (37°C, 10 min), followed by 2 mM sodium taurocholate (Sigma-Aldrich) in PBS with Ca2+ and Mg2+ (16°C, 10 min) in order to stimulate excystation [87]. The screen was carried out in clear bottomed 384-well microtiter plates by inoculating >90% confluent human ileocecal adenocarcinoma (hct-8) cells (ATCC) with ∼5.5 × 10^3^ primed oocysts per well. Experimental compounds were added 3 h after infection at a final concentration of approximately 2.3 µM, and the cells were incubated for 48 h. Wells incubated with an equal volume of DMSO alone served as a negative control. After being washed and fixed, the cells were probed with a mixture of biotinylated *Vicia villosa* lectin (VVL) (0.67 μg/ml) (Vector Laboratories) and streptavidin-conjugated Alexa Fluor 568 (1.33 μg/ml) (Invitrogen) diluted in 1% bovine serum albumin (BSA)-PBS with 0.1% Tween 20 for 1 h at 37°C to label vegetative forms of *C. parvum*, and the nuclei were counterstained with Hoechst 33258 [85, 87]. Imaging was performed using a Nikon Eclipse TI2000 epifluorescence microscope with a motorized stage and an EXi Blue digital camera (QImaging, Surrey, British Columbia, Canada); NIS-Elements Advanced Research software (Nikon USA, Melville, NY) was used to direct the acquisition of a three-by-three 20× field photo of the center of each well (covering approximately 13% of the surface area of each well). The images were exported as .tif files into NIH ImageJ software and host nuclei and parasites were enumerated by using the batch process function to execute previously validated macros [85]. Results: There were numerous active compounds, but none were completely clean of toxicity for zebrafish and mammalian cell lines. MMV665917 had a >20x SI and was listed in Table 1.

**Columns CJ, CK, CM, CO & FH:** Maes group (published in ref [88])**.** Screening of Malaria Box compounds for growth inhibition (IC_50_s) of *Trypanosoma brucei rhodesiense* STIB 900, *T. b. brucei* Squib 427, intracellular amastigotes *of Leishmania infantum* MHOM/MA/BE/67 in primary macrophages, intracellular amastigotes *of T. cruzi* Tulahuen in MRC5_sv2_ cell line (MRC), and MRC5_SV2_ human fibroblasts (MRC) were performed as described [89, 90]. A full dose-titration was performed to permit calculation of an exact IC_50_ and an independent repeat experiment was run (which was fully confirming). Results: Reference controls are listed in the table below for the two screening runs. There were no compounds that selectively inhibited intracellular growth of *L. infantum* and there was poor correlation of macrophage growth with axenic *L. donovani* inhibition (column CN) largely because macrophage growth inhibition was higher compared to axenic amastigote inhibition. There was good correlation with the *T. brucei* screens performed both by the Maes group and the Scynexis group, and the non-toxic compounds inhibiting all the *T. brucei* screens (SI >10) are MMV020505 (note: this compound inhibited replication of some breast, colon, lung, leukaemia, and melanoma cancer cell lines) and MMV020548 (note: MMV020549 is similar but more toxic). There was poor correlation with the Scynexis *T. cruzi* screen and two active compounds were identified with an IC_50_ less than 0.5µM: MMV001230 (IC_50_ = 156 nM) and MMV007907 (IC_50_ = 281 nM).

| **Screening** | | **Positive Control Compound** | Run 1 | Run 2 |
| --- | --- | --- | --- | --- |
|  |  |  | IC_50_ | IC_50_ |
| 1 | MRC-5 | Tamoxifen | 11.06 | 11.35 |
| 2 | *T. cruzi* | Benznidazole | 3.22 | 3.06 |
| 3 | *L. infantum* | Miltefosine | 7.1 | 9.0 |
| 4 | *T. brucei brucei* | Suramin | 0.06 | 0.05 |
| 5 | *T. rhodesiense* | Suramin | 0.07 | 0.05 |

**Column CQ & CK:** Boyom group (published in ref [91]). *Toxoplasma gondii* and *Entamoeba histolytica* growth inhibition. The Malaria Box compounds were used at a 30 µM top concentration and diluted as needed in respective culture media for individual experiments. Standard strains *Toxoplasma gondii* TS-4 and *Entamoeba histolytica* Rahman were obtained from BEI Resources (Manassas, VA). *T. gondii* TS-4 parasites were cultured in Human foreskin fibroblast cells (American Type Culture Collection, Manassas, VA) and drugs tested for anti-Toxoplasma activity by targeting the parasite tachyzoite form as described [92], with some modiﬁcations. Anti-*E. histolytica* activity was determined as described [93] with some modiﬁcations. Activity was expressed as 50% inhibitory concentrations (IC_50_). Results: There were five nontoxic Malaria Box compounds active against *T. gondii* (MMV666095, MMV007363, MMV007791, MMV007881, MMV006704) but no nontoxic Malaria Box compounds active against *Entamoeba histolytica.*

**Columns CR-CU:** Igarashi group (data presented first in this paper). *Babesia* and *Theileria* antiproliferative activity. Method: The inhibitory effects of 400 compounds (200 drug-like and 200 probe-like) from the Malaria Box upon *Babesia* and *Theileria* parasites growth were tested using the fluorescence-based assay previously described [94]. Briefly, *B. bovis* (Texas strain), *B. bigemina* (Argentine strain), *T. equi* (U.S. Department of Agriculture (USDA) strain) and *B. caballi* (USDA) pRBCs were cultivated in 96-well plates at 1% parasitemia with non-parasitized RBCs to 2.5% hematocrit (HCT) or 5% HCT for bovine *Babesia* parasites (*B. bovis* and *B. bigemina*) and equine *Babesia* and *Theileria* parasites (*B. caballi* and *T. equi*), respectively. M199 or RPMI 1640 media alone or with indicated concentrations of drugs: 0.1, 1, 10, and 25 µM for 400 compounds in 100 µl final volume, were added to the culture. Non-parasitized RBCs were loaded into each well in triplicate and used as a blank control. The RBCs were cultivated for 4 days without daily replacement of the medium in triplicate wells for each concentration of the drug. On the 4th day of culture, 100 µl of a lysis buffer was mixed with a 2×SGI (SYBR Green I) nucleic acid stain (Lonza, USA; 10,000x) and added directly to each dilution on each plate. The plates were then incubated for 6 hours in a dark place at room temperature, and the fluorescence values were determined using a fluorescence plate reader (Fluoroskan Ascent, Thermo Labsystems, USA) at 485 nm and 518 nm excitation and emission wavelengths, respectively. Gain values were set to 100. Then, 50% inhibitory concentration (IC_50_) values were calculated.

Results: MMV396693, MMV073843, MMV666093, MMV665875 and MMV006706 hits were the most interesting with mean selectivity indices (SI) greater than 230 and IC_50_s ranged from 43 to 750 nM for both bovine *Babesia* and equine *Babesia* and *Theileria* parasites. Additionally, 64, 45 and 49 MMV compounds were exhibited IC_50_s lower than those of diminazene aceturate (most widely used antibabesial drug) against the *in vitro* growth of *B. bovis, B. bigemina and T. equi*, respectively. The *in vitro* growth of Bovine and equine *Babesia* and *Theileria* parasites were significantly inhibited (p ˂ 0.05) by exposure to 100 nM of MMV396693. Additionally, *in vitro* exposure to MMV006706 significantly inhibited (p ˂ 0.05) the growth of *B. bovis*, *T. equi* and *B. caballi* by 100 nM and significantly inhibited (p ˂ 0.05) the growth of *B. bigemina* by 1 µM (data not shown). *In vitro* exposure to MMV666093 significantly inhibited (p ˂ 0.05) the growth of Bovine *Babesia* and *T. equi* parasites by 100 nM. The *in vitro* growth of Bovine *Babesia* parasites were significantly inhibited (p ˂ 0.05) by 100 nM of MMV073843, whereas this compound significantly inhibited (p ˂ 0.05) the *in vitro* growth of equine *Babesia* and *Theileria* parasites by 1 µM (Data not shown). Moreover, the *in vitro* growth of bovine Babesia and equine *Babesia*/ *Theileria* were significantly inhibited (p ˂ 0.05) by exposure to 1 µM and 100 nM of MMV665875, respectively (Data not shown). *In vitro* screening of Open Access Malaria Box compounds against *Babesia bovis*, *B. bigemina*, *Theileria equi* and *B. caballi* helped in the discovery of 10 novel potent anti-babesial hits which exhibit submicromolar potency against both bovine *Babesia* and equine *Babesia* and *Theileria*. This represents a 2.5% hit rate, somewhat higher than that observed by the *in vitro* screening of Malaria Box compounds against either *Toxoplasma gondii* and *Entamoeba histolytica* (1.75% hit rate) [91] or *Schistosoma mansoni* (0.75% hit rate) [95]. However, our hit rate is similar to that obtained by the MMV compounds in vitro screening against *Cryptosporidium parvum* [85]. This may be due to high target similarity shared among *Plasmodium, Cryptosporidium,* and *Babesia* protozoan parasites [91]. The *in vitro* treatment by the 10 hits identified in this study for *B. bovis* and *T. equi* parasites, revealed IC_50_s lower than that obtained by luteolin, pyronaridine tetraphosphate, nimbolide, gedunin and enoxacin treatment [94]. Moreover, the IC_50_ values of MMV666093, MMV006706, MMV665941, MMV085203, MMV396693, MMV073843, MMV007092 and MMV665875 compounds for *B. bigemina* were lower than those for luteolin, pyronaridine tetraphosphate, nimbolide, gedunin and enoxacin [94]. While, the IC_50_ values of MMV396794 and MMV006787 compounds for *B. bigemina* was nearly similar to those for luteolin and pyronaridine tetraphosphate, respectively [94]. On the other hand, the *in vitro* treatment of *B. bigemina* by MMV396794 and MMV006787 compounds revealed IC50s lower than those obtained by nimbolide, gedunin and enoxacin treatment [94]. Additionally, the IC_50_s of MMV666093, MMV396794, MMV665941, MMV085203, MMV396693 and MMV073843 for *B. caballi* were lower than those for luteolin, pyronaridine tetraphosphate, nimbolide, gedunin and enoxacin [94]. Whereas, the IC_50_s of MMV006706, MMV006787, MMV007092 and MMV665875 compounds for *B. caballi* were nearly similar to those for pyronaridine tetraphosphate [94] and lower than those for luteolin, nimbolide, gedunin and enoxacin [94]. The ten potent hits identified in this study exhibited IC_50_s for bovine *Babesia* and equine *Babesia* and *Theileria* lower than that obtained with the treatment by apicoplast-targeting antibacterials (ciprofloxacin, thiostrepton, and rifampin), miltefosine, fusidic acid, or allicin [96-99].

**Columns CV-CW:** Fernández Robledo group (published in ref [100]). Screening of *Perkinsus marinus.* “Dermo” disease caused by the protozoan parasite *Perkinsus marinus* (*Perkinsozoa*) is one of the main obstacles to the restoration of oyster populations in the USA. Based on the phylogenetic affinity between the Perkinsozoa and *Apicomplexa*, we exposed *Perkinsus* trophozoites to the Malaria Box compounds. For the primary screening, *Perkinsus* trophozoites were exposed 20 µM and the effect evaluated at day 4 post-exposure as reported elsewhere [101]. The IC_50_ of the best six hits was calculated in an 8-point dose-response curve (10 µM to 0.156 µM) and the same compounds were tested in eight *P. marinus* strains and five *Perkinsus spp*. (2 µM at day 2 post-exposure) [100].

**Columns CY-CZ:** Kyle group (data presented first in this paper). *Naegleria fowleri* trophozoite growth inhibition. Methods: The CellTiter-Glo (CTG) 2.0 luminescent assay is a quantitative assay that assesses the presence of cellular ATP in lysed cells, displaying an output in Relative Light Units (RLU’s). This was previously standardised [102], for the growth and development of *Naegleria fowleri* trophozoites in-vitro to assess drug susceptibility on this parasite. Initial screening of the Malaria Box was carried out as a single point assay screened at 1 µM; from these data we identified 5 active compounds. EC_50_s for these hits were assessed from 62.5 nM to 2 µM in 6x doubling dilutions. Results: Of the 400 Drug- & Probe-like molecules in the Malaria Box screened, only 5 compounds showed activity against pathogenic *Naegleria fowleri* at 1 µM concentration in the single-point screening method. These included two drug-like molecules MMV665807 (St. Jude’s Children’s Hospital) and MMV665979 (The Genomics Institute of the Novartis Research Foundation - GNF) and two Probe-like molecules, MMV006203 (GNF) & MMV006172 (GNF; GlaxoSmithKline - GSK). All other compounds showed poor activity against *N. fowleri*, therefore these were not considered for further assessment. The EC_50_ for each of the compounds were found to be MMV665807 (1,090 nM), MMV665979 (190 nM toxic), MMV006203 (420 nM toxic) & MMV006172 (450 nM, toxic). However, these Malaria Box compounds were all toxic to zebrafish and mammalian cells.

**Columns DA-DC:** Hemphill group (data presented first in this paper). *Neospora caninum* and human fibroblasts antiproliferative effect. Methods: Assessment of the effects of Malaria Box compounds on human foreskin fibroblasts (HFF, ATCC) was done in flat bottomed 96 well plates inoculated with 103 HFF and grown overnight. Cells were exposed to 1 µM of each compound for a period of 5 days. Viability was assessed by measuring fluorescence at 590 nm after the addition of resazurin (10 mg/ml) at various time points according to Müller *et al.* [103]. *In vitro* efficacy screening of transgenic *Neospora caninum* Nc-1 tachyzoites expressing β-galactosidase (Nc-Betagal) grown in HFF monolayers was performed as previously described [104]. Primary screening was done at 1 µM, and the IC_50_ of selected compounds were further assessed for determination of the IC_50_ by treating with different concentrations ranging from 0.5nM to 1µM. Negative control wells were exposed to DMSO only (max 0.1%) and no effect on N. caninum proliferation was observed. As a positive control, BKI-1294 [105] was used, leading to complete block of proliferation at 1 µM. Results: Many of the compounds that were active on *Neospora* could be ruled out by toxicity on the accompanying host cell fibroblast screen, but most of the others positive on Neospora but negative on the host cell screen were toxic at 10μM or below for mammalian cells and zebrafish. The remaining non-toxic *Neospora* actives that bear further investigation include: MMV19670, MMV000911, & MMV006309.

**Columns DD-DE:** Carter group (data presented first in this paper). *Chromera velia* antiproliferative effect. *Chromera velia* cultures and maintenance conditions: Chromera velia strain CvLp_vc08/1 isolated from the stony coral *Leptastrea purpurea* from One Tree Island, Great Barrier Reef, Queensland, Australia was used throughout this study [106]. Cells were grown in T75 culture flasks (Corning, USA, 431464) with 50 ml Mf/2, a filtered modified f/2 medium [107]. Iron was added in the form of ferric citrate to a final concentration of 1 μM. Primary cultures were incubated at 25° C under 12:12 hours light:dark cycle with light intensity of 70 μmol.m^-2^.s^-1^ (PARsensor, LI-190, Li-Cor Biosciences) switching at 7 h and 19 h, and were maintained by fortnightly transfers into new culture media. Flagellation motility assay: Compounds from the Malaria Box were tested on the flagellated state of *C. velia* [108], with the level of flagellated cells present used as a measure of cell health. The flagellated state was chosen over the immotile coccoid state as it lacks the thick cell wall that could prevent drugs entering the cell, and it is structurally similar to the infective form of *Plasmodium* and may therefore be more biologically relevant. *Chromera velia* cells from the primary culture were transferred to 48 well plates in 1 ml aliquots at a starting concentration of 10^5^ cells/ml in Mf/2 media. Plates were kept in the dark at a constant temperature of 25 °C for 4 days, when extent of flagellation was observed using an inverted microscope at 2-4 pm (previously found to have highest level of flagellation, Weatherby unpublished). Only plates with at least 25% of cells in the flagellated state were used in the assay. Compounds from the Malaria Box were diluted to 1 mM in DMSO then diluted a further 1/10 in Mf/2 before being added to the wells at a final concentration of 1 μM. Each of the 400 compounds from the Malaria Box was tested in duplicate. Controls for growth consisted of a) *C. velia* with no added compound; and b) *C. velia* with DMSO added to a final concentration of 0.1%, which is the same concentration as was present in the test wells. Controls for inhibition included 0.1 μM and 1 μM triclosan, which has been found to completely block flagellation and inhibit growth (Weatherby, unpublished). All controls were present in duplicate on each 48 well plate. Incubation was continued in the dark at 25 ˚C, and flagellation was enumerated visually using an inverted microscope and camera 24 and 72 hours following the addition of compound.

Results and interpretation: The growth controls with no added compound and with 0.1% DMSO produced the same level of flagellates (standardised as ++ in Table S3). The inhibition control containing 0.1 µM triclosan had a reduced number of flagellates, while the 1 µM triclosan control had no flagellates (scored as + and 0 respectively; Table S3). Only seven of the Malaria Box compounds completely inhibited flagellation of *C. velia* and were scored 0. This was verified in a second experiment and found to be consistent. Some compounds induced more flagellates than the growth controls and were scored +++. All of the seven compounds that completely inhibited flagellation also inhibited the growth of at least some of the protozoan and helminth species tested, and overall inhibition patterns were more similar to helminths than to protozoa. All compounds that affected flagellation were also toxic in at least one of the mammalian cell culture or zebrafish experiments.

**Columns DI-DJ, DL:** Bickle group. (columns DI and DL data presented first in this paper, column DJ partially published in ref [95].). Schistosomiasis assays: *In vitro* screening of *S. mansoni* adult worms run at LSHTM (London) Adult worm drug testing was performed by as previously reported [109] with some modification as described. Male CD1 mice (aged 5–6 weeks) were bred on site using SPF conditions with access of food and water ab libitum. Worms were obtained by portal perfusion of CD1 mice (Charles River, UK) 6 weeks post-infection and 3 worm pairs added to the wells of 48 well plates (Nunc, UK) in 1 ml complete DMEM medium supplemented with 10% fetal calf serum (Gibco, UK), 2 mM L-glutamine, 100 U/ml penicillin, 100 µg/ml streptomycin (cDMEM). A primary adult worm screen was performed in which 400 MMV compounds were tested at 15 µM in 0.15% DMSO. Negative controls contained worms cultured in cDMEM alone and in cDMEM with 0.15% DMSO. Positive control wells contained worms cultured in Praziquantel (Sigma-Aldrich, UK) at 10 µM. Cultures were incubated at 37ºC and 5% CO_2_. Effects were assessed on day 5 of culture using an inverted microscope (Leitz Diavert Wetzlar, Germany). Any compounds producing complete immobility or ≥ 70% worm motility inhibition plus severe morphological damage were considered hits in the primary screen. Active compounds were then tested for IC_50_ at 15 – 0.55 µM. IC_50_ for the compounds was calculated using Microsoft XLfit version 5.1.0.0 (2006-2008 ID Business Solutions Ltd). *In vitro* screening of *S. mansoni* schistosomula run at LSHTM (London): Schistosomula were prepared as previously reported [110] with the modification of using a 45% and 70% Percoll gradient and the cercarial heads recovered from the 70% layer. All centrifugation steps were done at 350 *xg*. An appropriate volume of drug Stock was pre-added to wells in 96-well microtiter culture plates (Nunc, UK) containing 100 µl complete M169 (cM169). The wells were then topped up with another 100 µl of cM169 containing 100 schistosomula to give final concentrations of 15 µM of compound per well. Negative controls contained larvae cultured in cM169 alone and in cM169 with 0.5% DMSO. Positive control wells contained schistosomula cultured in Praziquantel (Sigma-Aldrich, UK) at 10 µM. Cultures were incubated at 37ºC and 5% CO_2_. Effects were assessed on day 3 of culture using an inverted microscope (Leitz Diavert Wetzlar, Germany). Any compounds producing complete immobility or ≥ 70% motility inhibition plus severe morphological damage were considered hits in the larval screen.

**Columns DK, DM, DS-DT:** J. Keiser group (columns DS-DT data presented first in this paper, columns DK & DT were published in ref [111]). Helminth assays. We tested the MMV Open Access Malaria Box on a panel of parasitic helminths: *Schistosoma mansoni*, *Ancylostoma ceylanicum* and *Trichuris muris*. The methods by which screening was conducted on *S. mansoni* newly transformed schistosomula (NTS) and adult worms, *in vitro* and in vivo, are well described [95]. Praziquantel served as positive control (NTS IC50 2.2 µM; adult IC50 0.2 µM) [95]. The nematodal screening was first done on *A. ceylanicum* L3 larvae at a concentration of 200 μM. The screen was repeated on *T. muris* L1 larvae at a concentration of 100 µM. Levamisole and ivermectin were included as positive controls in the *A. ceylanicum*(200 μM) and *T. muris*(100 μM) assays, which caused death of all worms. Details on assay set up, maintenance of *A. ceylanicum* and *T. muris* life cycles, and animal conditions have been previously described [111, 112].

**Columns DN-DQ**: Caffrey group (data presented first in this paper). Schistosomiasis assays: Schistosome material. At UCSF, a Puerto Rican isolate of *Schistosoma mansoni* is maintained by passage through albino *Biomphalaria glabrata* snails and infection of 3-5 week-old, female *Mesoricetus auratus* (Golden Syrian hamsters) [113, 114]. Methods for harvesting cercariae, their mechanical transformation to schistosomula (somules) [115] and the establishment of *in vitro* somule screens are described [114, 116]. Methods for harvesting adult schistosomes [113, 117] and the establishment of *in vitro* screens are described [114, 118, 119]. The manifold phenotypic responses possible for this parasite are adjudicated by microscopic observation. We employ simple ‘descriptors’ to record changes in movement, shape, translucence, surface integrity and, for adults specifically, the ability of the parasite to adhere to the culture dish surface [114, 118]. To convert these observations into a partially quantitative output in order to facilitate relative comparisons of compound effects, each descriptor is awarded a ‘severity score’ of one up to a maximum score of four [118-120]. When damage to the adult parasite’s tegument (surface) is evident, the maximum score of four is awarded on the assumption that such damage is lethal to the parasite in the mammalian host [121]. For adult males, we also measure motility as described [122]. The reader is encouraged to view an expanded set of the data presented here on the ChEMBL database (ChEMBL assay IDs: CHEMBL2363023 through CHEMBL2363031). Negative controls contain the compound solvent (DMSO) at the final concentrations used (0.5-1%). Positive controls employ on one more of the following anti-schistosomal compounds, praziquantel, niclosamide and simvastatin. Positive controls employed drugs with known anti-schistosomal activity, namely praziquantel and niclosamide. At the concentrations employed in the somule and adult parasite screens (2.5 and 5 µM, respectively), these drugs yielded the maximum severity score of 4 after 48 h. In the motility assay for adult males, both compounds decreased motility to zero at 48 h.

**Column DW**: Townson group (data presented first in this paper). Screening of *Onchocerca lienalis* microfilariae for motility inhibition in microplates. Methods: Microfilariae are obtained from samples of peri-umbilical skin of freshly slaughtered, naturally infected cattle in the UK. Compounds are initially tested at a concentration of 12.5 µM in a single well of a 96-well plate containing 5 worms and a monkey kidney cell feeder layer, in a 5-day assay. An inverted microscope is used to note the condition and motility of each microfilaria over the 5-day duration; briefly, the motility of the mf is classified as normal (continuous rapid sinuous movement, scored as 3), marginally impaired (slower than normal, scored as 2), severely impaired (scored as 1), or immotile (scored as 0). The results are then calculated as motility index scores and expressed as a percentage of the maximum obtainable. In this study the microfilariae motility levels of negative (untreated) controls maintained 100 % throughout the trial, while those in the positive controls (Immiticide; Merial) were immotile at the end of the trial. [123].

**Column EA:** Suzuki group (data presented first in this paper). Screening of MMV compounds for *Saccharomyces cerevisiae* ABC16-Monster growth inhibition. Methods: Compounds were added at 100 micromolar to YPD medium (1% yeast extract, 2% peptone, and 2% glucose). Only the solvent dimethyl sulfoxide (DMSO) without any MMV compound was added at 1% to the negative control. The initial density of ABC16-Monster cells corresponded to optical density at 600 nanometers (OD_600_) of 0.01. Cells were cultured without agitation in a 96-well plate at 30 degrees Celsius for 18 hours and then resuspended for measurement of OD_600_. For each compound, two cultures were conducted and examined. The average OD_600_ value was used. To calculate percent inhibition, OD_600_ for cells cultured with a compound was divided by OD_600_ for the negative control. The ABC16-Monster yeast strain was previously described [124].

**Columns EB-ED:** St. Onge group (data presented first in this paper). Growth inhibition of *S. cerevisiae:* The effects of Malaria Box compounds on the growth of yeast (*Saccharomyces cerevisiae*) were quantified as described previously [125]. We employed the prototrophic yeast strain JHY222, a derivative of BY4743 in which auxotrophic markers have been repaired [126]. Growth was measured in three different growth media: rich medium using dextrose as a carbon source, minimal medium using dextrose as a carbon source, or minimal medium using ethanol and glycerol as carbon sources. Compounds affecting growth in a media-specific manner may represent inhibitors of key metabolic pathways. For example, the antifolate drug methotrexate was previously shown to inhibit yeast growth far more potently in minimal medium compared to rich medium [127].

**Columns EE-EH:** Ben Mamoun group (data presented first in this paper). Inhibitors of PC biosynthesis via phosphoethanolamine transmethylation. Genetic and metabolic studies in *P. falciparum* have shown that phosphatidylcholine (PC) biosynthesis requires two non-redundant metabolic pathways: the CDP-choline pathway, which uses host choline and fatty acids as precursors, and the SDPM pathway, which uses host serine (derived from haemoglobin catabolism) and fatty acids as precursors [128]. A limiting step in the SDPM pathway is catalyzed by the phosphoethanolamine methyltransferase PfPMT. The gene encoding PfPMT was shown by genetic studies to play an important role in asexual replication and sexual differentiation [129-131]. The reaction catalyzed by PfPMT is unique to a small number of eukaryotes and is absent in mammals and fungi [129-135]. As a result we created a yeast strain that is incapable of synthesizing PC in the absence of exogenous choline and thus relies on the malaria PfPMT for survival [134]. Using this yeast strain we screened the Malaria Box for compounds that inhibit PC biosynthesis. The selectivity of these compounds was determined by performing the screening in the absence as well as the presence of exogenous choline. As a control the same screen was performed using wild type yeast cells in the absence or presence of choline. Chemical screening in yeast. For screening of the Malaria Box, yeast strains were pre-cultured overnight in SD medium without ethanolamine. Cells were then washed in water, diluted and dispensed into 96 well plates at 2x104 cells/ml in SD medium lacking or supplemented with choline in the absence or presence of 100 µM of each compound diluted in 1% DMSO. Controls included wells containing wild type yeast cells, pem1∆pem2∆ harbouring an empty vector in SD medium, either lacking or supplemented with choline and containing 1% DMSO. The plates were incubated at 30˚C and read at A660 48h following inoculation. Compounds found to inhibit the growth of the pem1∆pem2∆+PfPMT strain but now wild type in the absence of choline but not in the presence of choline were considered PfPMT specific. Compounds that inhibited growth of both strains independent of choline supplementation are those with anti-fungal activity. Using these criteria 14 compounds were identified as candidate inhibitors of PfPMT activity using the yeast functional assay. These compounds are: MMV000570, MMV006172, MMV007384, MMV666080, MMV666009, MMV666071, MMV396736, MMV000445, MMV000304, MMV667491, MMV000704, MMV396723, MMV007041 and MMV666687.

**Column EI:** S. Avery Group (data presented first in this paper). Yeast Respiratory screen: MMV drugs were screened for sensitivity or resistance phenotypes with the yeast deletion strain collection [136]. The deletion strains were either in the Saccharomyces cerevisiae BY4741 background or in an isogenic, drug pump-defective strain background (pdr1Δ, pdr3Δ, sqn2Δ). The deletion strain screens were performed essentially as described previously [137, 138], but in 384-well format and with phenotype analysis using ScreenMill software where stated [139] to evaluate relative colony growth on unsupplemented and MMV drug-supplemented solid medium. DMSO at a final concentration of 0.1% (v/v) was included in all media. The media used were YNB (with glucose as fermentable substrate) or YEPG (with glycerol or ethanol as respiratory substrate) [140]. The drug-pump knockout strain was used for specific tests of MMV drug sensitivity in fermentative or respiratory broth media, with OD_600_ monitored continuously during shaking in microplates as described previously [141]. The organism on either medium was classified as either drug hyper-sensitive, sensitive or resistant when growth was <30%, <75% or ˃75%, respectively, in the presence of drug than in the minus-drug control. The drug was considered to produce a respiratory- or fermentative-sensitive phenotype where these outcomes differed for the two growth regimes.

**Columns EK-ER**: Baker group (data presented first in this paper). Bacterial Screening of Malaria Box Compounds. Methods: All of the compounds were dissolved in DMSO (as recommended by the supplier) to form 20mM stock solutions, which were then diluted in sterile distilled water (Sigma-Aldrich) to form 10 μM working solutions for chemical screening. Chemical stocks and working solutions were then stored at -20^o^ C until required. All chemical testing was performed at an initial concentration of 10 μM.

The bacteria (a combination of ATCC strains and clinical isolates) selected for screening were cultured (*Acinetobacter baumannii, Salmonella Typhimurium, Pseudomonas aeruginosa, Escherichia coli, Streptococcus suis, Streptococcus pneumoniae* and *Staphylococcus aureus*) on the Luria-Bertani agar overnight and colonies were picked and separately inoculated into sterile PBS to form either 0.5 McFarland (*A. baumannii, S.* Typhimurium*, P. aeruginosa* and *E. coli*) or 1 McFarland (*S. suis, S. pneumoniae* and *S. aureus*) solutions. The prepared bacterial suspensions were then evenly spread on Mueller-Hinton agar using sterile cotton swabs (Medical Wire – MWE). To test for antibacterial activity of the chemicals, 5μl of the 10 μM solutions were pipetted onto the surface of the media; 5μl of 1 μg/μl of ciprofloxacin was used as a positive control; sterile PBS was used as a negative control. Plates were incubated overnight at 37^o^ C. For the screen, the activity of the chemical was recorded as being active (yes) or non-active (no) for each of the tested chemicals. A non-active result was recorded when there was no evidence of bacterial inhibition and the culture area was indistinguishable from the negative control. An active result was recorded when there was an obvious zone of inhibition of bacterial growth and the observed zone was similar to that of the positive control, *i.e.* an area without bacterial growth where the media could be observed using ciprofloxacin activity as a comparator.

Minimum inhibitory concentrations (MIC) (Micro dilution assay): The MICs of chemicals with antimicrobial activity were determined using a 1mM starting solution of the active chemical. MIC testing was performed using Mueller Hinton broth with organisms grown to an approximate final concentration of 1x10^6^ CFU/ml bacteria. MIC testing was performed by preparing serial dilutions into a 96-well plate, starting with a 10uM dilution in the first column followed by further 2-fold dilutions until the eighth column, up to a volume of 100 μl. 100 μl of the bacterial solution was inoculated to each well and the plate was incubated at 37^o^C overnight (final concentration 5x10^5^ CFU/ml). To measure the MIC of each chemical a 10 μl drop the overnight bacterial culture with chemical was inoculated on a Luria-Bertani agar plate, the plates were incubated overnight at 37^o^C. Results were interpreted as the minimal concentration required for the inhibition of growth. Similar dilutions of ciprofloxacin were used at positive control for each of the organism/chemical combinations.

Results: OUCRU researchers (Investigator-Vinh) screened the 400 Malaria Box chemicals at a 10μm concentration against a range of clinical and reference strains of *Staphyloco*ccu*s aureus,* *Acinetobacter baumannii, Escherichia coli, Pseudomonas aeruginosa, Klebsiella pneumoniae, Salmonella* Typhimurium, *Streptoco*ccu*s pneumoniae* and *Streptoco*ccu*s suis*. We identified sixteen chemicals (Malaria Box nrs: MMV007224, 665794, 665807, 665852, 665882, 665953, 666023, 019017, 403679, 007574, 000445, 000787, 000304, 665797, 006319 and 019690) that had detectable antibacterial activity against one or more of the organisms that were screened. We found out that seven of sixteen hits (007224, 665794, 665807, 665852, 665882, 665953, and 666023) induced large reproducible zones of inhibition (10-20 mm) than the other nine compounds and were considered to have good antibacterial activity. Two of these chemicals (665807 and 665953) had low cytotoxicity and had activity against both *S. aureus* (Gram-positive) and *A. baumannii* (Gram-negative) with Minimum Inhibitory Concentrations (MIC) ranging from 1.25 to 5 μM with three ATCC strains of *S. aureus* and one ATCC strain of *A. baumanii.*

**Column ES:** Sullivan group (data presented first in this paper). *Wolbachia* reduction screen. Methods: The compounds were tested and analyzed using high-throughput cell based screening techniques previously described [142]. Drosophila tissue culture cells constitutively infected with *Wolbachia* called JW18 were used [142]. JW18 cells treated with compounds were compared against untreated JW18 cells as a negative control and untreated uninfected JW18DOX cells (these cells are JW18 cells cured with doxycycline and no longer contain *Wolbachia*). Compounds that significantly reduced *Wolbachia* titre are listed as “Yes” and highlighted yellow. Compounds that significantly reduced *Wolbachia* titre equivalent to the uninfected controls are listed as “Yes” and highlighted red. Compounds that are shown as “Yes” and in yellow are considered hits and reduced *Wolbachia* numbers, but not completely to control uninfected levels. Compounds that did not reduce *Wolbachia* titre are listed as “No.” Compounds were considered a hit if *Wolbachia* titre was significantly reduced in at least 2 out of 3 replicates. Cells were treated at 100 µM final concentration for 5 days at 25^o^ C. Results/Discussion: We found that 7 of 37 hits had a benzimidazole structure. This is important because previous finding indicate that the benzimidazole structure may be important in *Wolbachia* titre reducing compounds [142]. Of these 7 hits with a benzimidazole structure, 4 were shown to be lethal to *Brugia malayi* (Scynexis), 1 was shown to reduce *Onchocerca linenalis* motility (Townson), and 1 inhibited the growth of *Streptoco*ccu*s pneumoniae* (Baker). *Brugia malayi* and *Onchocerca linenalis* are filarial worms that contain *Wolbachia*. Overall, 8 of 37 hits were shown to kill at least 43% *Brugia malayi* after 5 days (Scynexis). 3 of 37 titre-reducing compounds were shown to reduce *Onchocerca linenalis* motility by at least 66% (Townson). As described previously, the finding that a subset of the benzimidazole-like compounds are hits in both filarial nematode and *Wolbachia* screens suggests that the compounds may target both the nematode and its *Wolbachia* symbiont [142-144]. These findings suggest that focusing on benzimidazole derivatives may be a productive approach in identifying novel potent compounds to combat African river blindness and lymphatic filariasis.

**Columns EU-EZ:** Nathan group (data presented first in this paper)**.** *Mycobacterium tuberculosis* screening under replicating and non-replicating conditions. Methods: Replicating and non-replicating *Mycobacterium tuberculosis* mc^2^6220 (∆*panCD*∆*lysA* H37Rv) were screened at 25 µM (single point) or in dose-response format (0.2 to 100 µM) in 384-well format as previously described [145-149]. In brief, replicating *M. tuberculosis* at an OD_580_ of 0.01 in 7H9 medium at pH 6.6 containing tyloxapol (0.02%), glycerol (0.2%), glucose (0.2%), and supplemental CAS amino acids (0.5%), OADC (10%), lysine (240 µg/ml) and pantothenate (24 µg/ml) was exposed to 500 nL test agent (maintaining 1% DMSO final) for 7 days at 37°C with 5% CO_2_ and 20% O_2_. To render cells non-replicating, a mid-log replicating culture was washed with PBS containing 0.02% tyloxapol and then resuspended at an OD_580_ of 0.1 in a Sauton’s-based medium (omitting glycerol, citrate and asparagine) at pH 5.0, consisting of 0.5 g KH_2_PO_4_, 0.5 g MgSO_4_, 0.05 g ferric ammonium citrate, BSA (0.5%), NaCl (0.085%), tyloxapol (0.02%), and 0.5 mM NaNO_2_. Non-replicating *M. tuberculosis* was incubated 3 days at 5% CO_2_ and 1% O_2_, after which 60 µL of drug-free, replicating medium was added. Microplates were then incubated an additional 7 days at 5% CO_2_ and 20% O_2_. For both the replicating and non-replicating assays, the OD_580_ was determined and % inhibition calculated with respect to cells treated with vehicle (DMSO; 100% survival) or a positive control (rifampicin; 0% survival).

**Columns FA-FC**: Soldati Group (data presented first in this paper). *M. marinum, D. discoideum*, and *Acathamoeba castellani* assays. Each compound of the Malaria Box has been assayed at 10 µM in all three assays. First, the antibiotic activity was assayed directly against *Mycobacterium marinum* in its culture medium. Second, the toxicity and growth inhibitory effect of the compounds was assayed against the amoeba *Dictyostelium discoideum* in its culture medium. Finally, the “*in cellulo*” anti-infective activity of each compound was tested using the intracellular infection system *Acanthamoeba castellanii-M. marinum*. Values have been normalized using the DMSO carrier [150]. For each assay, values have been normalized to 1.00 using the DMSO carrier, which has no detectable effect on *D. discoideum* nor *M. marinum* growth, while rifabutin was used as positive control for the antibiotic assay and the infection assay, as it abolishes *M. marinum* growth (normalized value under 0.10) in broth at 10 µM and intracellularly at 30 µM [150]. Nicotine was used as a positive control for the *D. discoideum* toxicity assay as it abolishes its growth at 7.5 µM [150].

**Columns FE-FG:** BIOBIDE group (data presented first in this paper). Zebrafish embryo toxicity, mortality, and behaviour change. 3 days post fertilization (d*Pf*) zebrafish embryos from AB strain (wild type) were used for *in vivo* toxicity and behavioural screening. For this purpose, fish maintained at 28.5° C under a photoperiod of 14:10 hours light:dark, with continuously filtered water at pH 7-7.8, conductivity at 500-800 µS and O_2_ dissolved at 60 - 90 %, were crossed and fertilized eggs collected and incubated in E3 with ampicillin (100 µg/ml) and methylene blue (0.0001%) until 3d*Pf*. Then, embryos were incubated with each compound at 10 µM (1% DMSO) in E3 buffered with HEPES at 10 mM for 48 hours in 24 well plates, 5 embryos per well, 3 wells per condition (n=15). A group treated only with the vehicle was included in each plate as negative control. Afterwards, mortality and morphological alterations were assessed. Toxicity: When an altered phenotype (mortality, developmental abnormalities) appeared in more than 20% of cases it was considered toxic. Behavioural assays were carried out only in the cases where compounds did not induce any toxicity. For this purpose, 5d*Pf* larvae (treated and negative controls) were transferred from 24-well treatment plate to 96-well plate, one embryo per well. 96-well plate was introduced in DanioVision system powered by Ethovision (Noldus). The behaviour was evaluated recording a total of 40 minutes in a round of 10 minutes under light and 10 minutes in darkness repeated twice. As activity is expected to differ under light and dark periods, the analysis was performed for each one of the 10 minutes phases. The differences between treated and controls for each parameter (distance, velocity) and phase was considered significant if p value is less than 0.005 (scored y for yes in HeatMap). Also, the p values between 0.01 and 0.005 were taken into account as an indicator of tendency (scored m for maybe in heat map). The toxic character of a compound was determined by the consistent differences (in both dark and light periods) between treated embryos and controls. If the compound did not change behaviour, as defined above, it was scored n for no. If consistent results could not be obtained, the compound was scored i for indeterminate.

**Column FI:** Nathan & Gold group (data presented first in this paper). HepG2 cells (obtained from M Esperanza Herreros-Aviles at GlaxoSmithKline's Diseases of the Developing World campus in Tres Cantos, Spain) toxicity assays were performed as previously described [147]. The LD_50_ was calculated relative to the survival of HepG2 cells to a 2 day exposure to vehicle (DMSO; 100% survival) or a positive control (digitoxin; 0% survival).

**Columns FK-GB,** Moore group (data presented first in this paper). HIV and HIV-producing cell lines, toxicity and viral suppression. 5 mM MMV compounds were incubated with HIV-1 JR-FL or HIV-1 NL4-3 pseudoviruses and U87-CD4-CCR5 or U87-CD4-CXCR4 cells (obtained through the NIH AIDS Reagent Program, Division of AIDS, NIAID, NIH: U87 CD4+CCR5+ Cells from Dr. HongKui Deng and Dr. Dan R. Littman) respectively for 3 days [151]. Cells were assayed for infection by luciferase production (Bright-Glo Luciferase Substrate, Promega) and cell viability by MTT assay [152]. 5mM MMV compounds were next incubated with HIV-1 JR-FL or HIV-1 NL4-3 viruses and U87-CD4-CCR5 or U87-CD4-CXCR4 cells respectively for 7 days [153]. Cell supernatants were assayed for viral production by p24 ELISA [153] and cell viability by MTT assay [152]. The reverse transcriptase inhibitor AZT (10 µM final concentration) and the entry inhibitor T20 (10 µM) served as positive controls and each completely blocked viral infection, as seen by either luciferase or p24 production. DMSO (5%) was completely toxic to all cells (based on visual inspection) and served as the positive control for MTT assays.

**Columns GD-GJ:** Wuxi Inc. data for MMV (data presented first in this paper) PK/DMPK Parameters: Fasted animals (Male CD-1 mouse, 7-9 weeks age, 30±10 g, n=3) obtained from an approved vendor (SLAC Laboratory Animal Co. Ltd., Shanghai, China or Sino-British SIPPR/BK Laboratory Animal Co. Ltd Shanghai, China) were administered with a single oral dose (140 μmol/kg) using DMSO: 1% HPMC (5:95) as a vehicle. Blood sampling was performed via the submandibular or saphenous vein (t=0.083, 0.25, 1, 2, 4, 6 and 9 h) and transferred into microcentrifuge tubes containing Sodium Heparin (1,000 IU/ml) as anti-coagulant placed on wet ice until processed for plasma by centrifugation at approximately 4°C, 3000×g for 15 min. Plasma samples were stored in polypropylene tubes, quick frozen over dry ice and kept at -70±10°C until LC/MS/MS analysis. LC/MS/MS methods for the quantitative determination compounds plasma levels were developed with an internal standard. Finally concentration in plasma versus time data was analyzed by non-compartmental approaches using the WinNonlin software program [154, 155] (Phoenix WinNonlin, version 6.2.1, Pharsight, Mountain View, CA) and expressed as C_max_ & AUC_0-last_. C_max_ is the maximum concentration in plasma expressed as >1 µM (Yes or No). AUC_0-last_ is the area under the curve expressed as >12 h*μM (Yes or No). Plasma protein binding assay (PPB) (CD-1 mouse and human) expressed as <95% bound, >95% bound, or >99% bound. Kinetic Solubility at pH 7.4 was determined by liquid chromatography/chemiluminescence nitrogen detection (LC/CLND) and was scored as greater than 20µM (Yes or No). hERG inhibition was measured at 11µM by the QPatch- HT system and is expressed as “Yes” if greater than 45% inhibited at 11µM and “No” if less than or equal to 45% inhibited at 11µM. As part of the Drug-Drug Interaction studies (DDI issues), 5 human and 1 mouse CYP isoforms (1A2, 2C9, 2C19, 2D6, 3A4-M, 3A4-T) were tested for inhibition assay at 10 µM. DDI issues were scored as “Yes” when there was >85% inhibition of CYP 1A2, 2C9, 2C19, or 2D6, or >70% inhibition of 3A4 (isoforms M or T) at 10 µM. Otherwise DDI issues were scored as “No”. Positive and negative controls were run for each assay (Table 1).

|  |  |  |
| --- | --- | --- |
| **Table 1 WuXi's Acceptance Range For Positive Controls:** | | |
| **1. Kinetic Solubility at pH 7.4:** | |  |
| **Control** | **Kinetic Solubility pH=7.4 (µg/mL)** |  |
| Amiodarone | <0.68 |  |
| Carbamazepine | 43 ± 4 |  |
| Chloramphenicol | 61 ± 4 |  |
|  |  |  |
| **2. CYP Inhibition:** | |  |
| **CYP Isozyme** | **Standard Inhibitor** | **IC_50_ Acceptance Range (μM)** |
| 1A2 | α-Naphthoflavone | 0.125~0.450 |
| 2C9 | Sulfaphenazole | 0.350~0.700 |
| 2C19 | (+)-N-3-benzylnirvanol | 0.100~0.350 |
| 2D6 | Quinidine | 0.0900~0.233 |
| 3A4-M | Ketoconazole | 0.0250~0.0800 |
| 3A4-T | Ketoconazole | 0.0280~0.0800 |
|  |  |  |
| **3. Plasma Protein Binding** | |  |
| **Control** | **Species** | **Bound%** |
| Warfarin | Human | >98 |
|  | Mouse | >90 |

**Columns GL-HB:**Bhatia group (data presented first in this paper). The efficacy of anti-malarial compounds can be dramatically altered in endemic regions when administered to HIV or TB patients, due to drug-drug interactions in the liver [156]. Existing compound sets should be annotated for this risk to prioritize candidates for further development and minimize failure rates. In this study we leveraged two recent breakthroughs: a bioengineered microscale human liver in a high-throughput format that accurately captures human drug-drug interactions not detectable in animals or cell lines [157] and a custom-made, robotic high-throughput Luminex bead-based method for profiling the expression of 83 human liver drug metabolizing enzymes for pennies a well [158]. Combining these tools, we profiled the “Malaria Box” drugs for induction or inhibition of drug-metabolizing pathways and thereby ranked compounds with potential for drug interactions with existing HIV and TB regimens. We also scored these compounds for acute hepatoxicity by monitoring morphology and daily albumin and urea secretion [157-160]. Methods: Micropatterned Co-Culture: MPCC is an *in vitro* co-culture system of primary human hepatocytes (BioreclamationIVT) organized into colonies and surrounded by supportive stromal cells. Hepatocytes in this format maintain a functional phenotype for up to 4-6 weeks without proliferation, as assessed by major liver-specific functions and gene expression [157, 161]. Briefly, 96-well plates were coated homogenously with rat tail type I collagen (50 μg/ml) and subjected to soft-lithographic techniques to pattern the collagen into microdomains of 500 μm islands that mediate selective hepatocyte adhesion. To create MPCCs, cryopreserved primary human hepatocytes (BioreclamationIVT) were pelleted by centrifugation at 100xg for 6 min at 4°C, assessed for viability using Trypan blue exclusion (typically 70–90%), and seeded on collagen-micropatterned plates (each well contained ~ 10,000 hepatocytes, organized in colony islands of 500 μm) in serum-free DMEM/1% Pen/Strep. The cells were washed with serum-free DMEM/1% Pen/Strep 2–3 hr later and replaced with human hepatocyte culture medium (March *et al* CHM. 2013). 3T3-J2 murine embryonic fibroblasts were seeded (7,000 cells per well) 24 hours post-hepatocyte seeding. 3T3-J2 fibroblasts were courtesy of Howard Green (Harvard Medical School) [162]. Hepatoxicity analysis: MPCCs assembled using a pre-validated cryopreserved human hepatocyte donor, were treated with a single acute dose (10 μM) of the Malaria Box compounds. After 48hrs of exposure, morphological analysis of the hepatocytes was performed, and albumin and urea levels in the supernatants were measured [159]. Urea concentration was assayed using a colorimetric assay kit (Stanbio Labs, Boerne, TX). Albumin content was measured using an enzyme-linked immunosorbent assay with horseradish peroxidase (Bethyl Labs) and 3,3’,5,5’-tetramethylbenzidine (TMB, Pierce) development. Doses that led to a decrease in albumin and/or urea production and an unhealthy hepatocyte morphology were scored as toxic. Ligation Mediated Amplification (LMA)-Luminex analysis: MPCCs assembled using a pre-validated cryopreserved human hepatocyte donor were treated with a single acute dose (10 μM) of the Malaria Box compounds. In each dosed 96 well plate we included DMSO and Rifampin CYP3A4 induction controls. After 48hrs of exposure, total RNA from four wells per compound was purified using Mini-RNeasy kit (Qiagen) and pooled for analysis. LMA-Luminex procedures and probes are previously described [163]. Briefly, 1 technical replicate was included for each biological replicate, results were expressed as the mean fluorescent intensity of at least 100 beads per sample, after scaling relative to the human transferrin gene, and row-normalized for HeatMap representation using Gene Pattern open-source software (Broad Institute) (Figure). Based on our experience, in order to use this data for compound prioritization, additional experiments are needed to cross-validate the results. Such experiments include the testing of compounds with another human hepatocyte donor, validation with another RNA measurement method (e.g quantitative-RTPCR), and measurements of p450 protein abundance and activity.


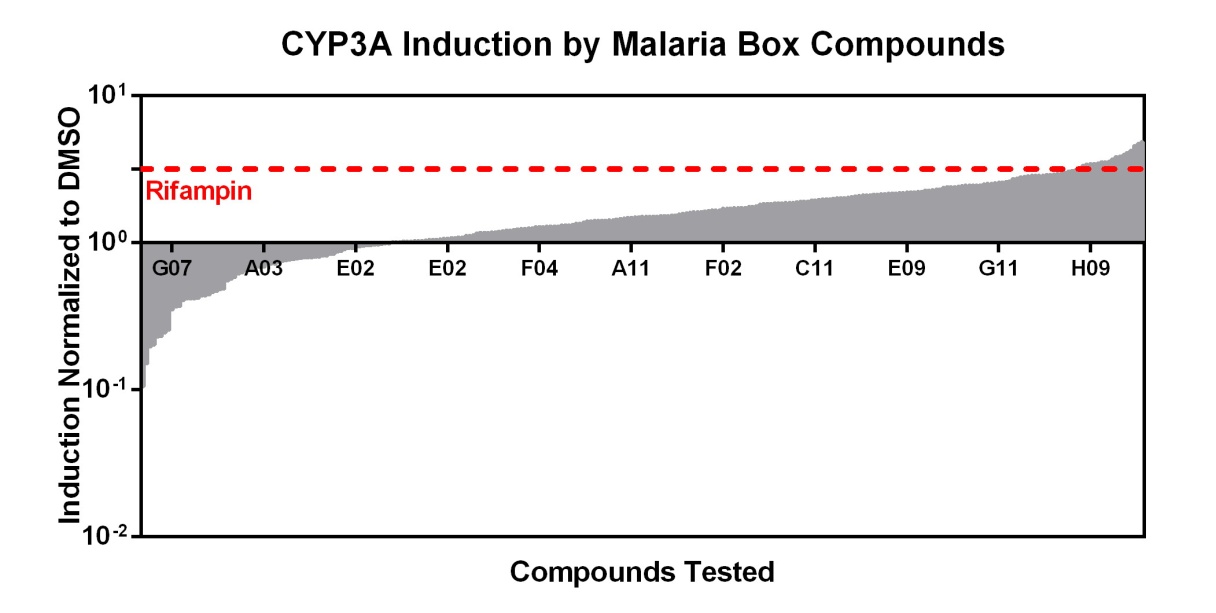


Figure legend: Analysis based on CYP3A4 expression at the mRNA level. MPCCs were assembled in a 96-well format, and treated with a single acute dose (10uM) of the Malaria Box compounds. In each dosed 96-well plate, DMSO and Rifampin CYP3A4 induction controls were included. Data in the graph represents fold induction of CYP3A4 expression by each of the Malaria Box compounds, normalized to DMSO (normalization was done by plate). Red dashed line indicates the average induction levels by Rifampin across all plates.

**Columns HC-HZ:** Mestres group (unpublished). *In vitro* affinity determinations. Many G protein-coupled receptors (GPCRs) have been associated with psychiatric, neurologic, and cardiovascular side effects and they are included in routine safety pharmacology panels [164].Therefore, *in vitro* affinity determinations on 23 selected human off-target GPCRs were performed on a set (10%) of MMV compounds. One of the most severe GPCR-related adverse effects is cardiac valvulopathy linked to 5-HT_2B_ activation [165, 166]. Therefore, some of the MMV compounds with significant binding affinity for the 5-HT_2B_ receptor were also tested on the corresponding functional assay to determine a potential agonistic effect. Methods: All compounds were assayed at 10 µM concentration against 23 radioligand binding assays (7 serotonin receptors: 5-HT_1A_, 5-HT_2A_, 5-HT_2B_, 5-HT_2C_, 5-HT_4_, 5-HT_6_, 5-HT_7_; 4 dopamine receptors: D_1A_, D_1A_, D_2_, D_3_, D_4_; 2 histamine receptors: H_1_, H_2_; 3 muscarinic acetylcholine receptors: M_1_, M_2_, M_3_; 4 alpha adrenergic receptors: α_1A_, α_2A_, α_2B_, α_2C_; and 3 opioid receptors: δ-, κ-, µ-opioid) and 1 functional agonistic assay (serotonin 5-HT_2B_ receptor). cDNAs subcloned into pCDNA3 vector were obtained from Genscript and transfected by calcium phosphate method. Reference compounds were included in all the assays, Ki values obtained for reference compounds are reported in the table below:

| Target | Compound | Ki (nM) |
| --- | --- | --- |
| 5HT1A | 5-CT | 0.46 |
| 5HT2A | Risperidone | 0.47 |
| 5HT2B | methysergide | 8.8 |
| 5HT2B | methysergide | 7.9 |
| 5HT2C | Risperidone | 8.6 |
| 5HT4 | GR113808 | 0.45 |
| 5HT6 | Methiotepine | 5.3 |
| 5HT7 | Methiotepine | 8.9 |
| D1A | Haloperidol | 9.2 |
| D2 | Haloperidol | 4.6 |
| D3 | Haloperidol | 36.7 |
| D4 | Clozapine | 63.2 |
| H1 | Clozapine | 0.49 |
| H2 | Cimetidine | 285.2 |
| M1 | Ipratropium | 0.81 |
| M2 | Atropine | 5.8 |
| M3 | p-F-HHSiD | 34.41 |
| α1A | Risperidone | 1.2 |
| α2A | Rauwolscine | 4.1 |
| α2B | Rauwolscine | 2.2 |
| α2C | Rauwolscine | 1.1 |
| δOP | [D-Pen2,5]Enkephalin | 36.4 |
| κOP | Naltrexone | 2.2 |
| µOP | DAMGO | 1.7 |

**5-HT_1A_ receptor:** competition binding experiments were carried out in HEK-5-HT_1A_ cell (human 5-HT_1A_ cDNA subcloned into pcDNA3 transfected in HEK cell line from ATCC) membranes. On the day of assay, membranes were defrosted and re-suspended in incubation buffer (50 mM Tris-HCl, 4 mM CaCl_2_, 1 mM ascorbic acid, 0.1 mM pargyline pH = 7.4). Each reaction well of a GF/C multiscreen plate (Millipore), prepared in duplicate, contained 10 µg of protein, 2 nM [^3^H]8-OH-DPAT and test compound. Reaction was incubated for 120 min at 37^o^ C, then samples were vacuum filtered and plate washed 4 times with 4^o^ C wash buffer (50 mM Tris-HCl, 4 mM CaCl_2_, 1 mM ascorbic acid, 0.1 mM pargyline pH = 7.4). Non-specific binding was determined in the presence of 10 µM serotonin.

**5-HT_2A_ receptor:** competition binding experiments were carried out in CHO-5-HT_2A_ (a generous gift from William P Clarke, University of San Antonio, Texas) cell membranes. On the day of assay, membranes were defrosted and re-suspended in incubation buffer (50 mM Tris-HCl, pH = 7.5). Each reaction well of a GF/B multiscreen plate (Millipore), prepared in duplicate, contained 160 µg of protein, 1 nM [^3^H]ketanserin and test compound. Reaction was incubated for 30 min at 37^o^ C, then samples were vacuum filtered and plate washed 4 times with 4^o^ C wash buffer (50 mM Tris-HCl, pH = 6.6). Non-specific binding was determined in the presence of 1 µM methysergide.

**5-HT_2B_ receptor:** competition binding experiments were carried out in membranes purchased from Perkin Elmer. On the day of assay, membranes were defrosted and re-suspended in incubation buffer (50 mM Tris-HCl, 4 mM CaCl_2_, ascorbic acid 0.1%, pH = 7.5). Each reaction well of a GF/C multiscreen plate (Millipore), prepared in duplicate, contained 15 µg of protein, 1 nM [^3^H]LSD and test compound. Reaction was incubated for 30 min at 37^o^ C, then samples were vacuum filtered and plate washed 4 times with 4^o^ C wash buffer (50 mM Tris-HCl, pH = 7.4). Non-specific binding was determined in the presence of 50 µM serotonin.

**5-HT_2B_ receptor:** functional agonistic assays were carried out with human 5-HT_2B_ transfected into a CHO cell line (Perkin Elmer) in 96-well plates and inositol phosphate formation was measured by using IPone HTRF kit (Cisbio). Cells were seeded (30000 cells/well) in 100 µL of cell culture medium (DMEM-F12 (Invitrogen) supplemented with 1% FBS (Sigma), 4 mM L-glutamine (Sigma) and 0.1% Penicillin/streptomycin (Sigma)) and maintained at 37^o^ C in a 5% CO_2_ atmosphere for 24 h. Medium was replaced by 60 µL of stimulation buffer provided in the kit. Compounds were added to the cells and incubated for 20 min at 37^o^ C. After this time, the antibodies from the kit were added in lysis buffer and incubated for 60 min at RT protected from light. Later, HTRF signal was measured in a Tecan Ultra Evolution reader (λ_exc_=320 nm, λ_em1_= 615 nm, λ_em2_= 665 nm). IPone formation was calculated following manufacturer’s instructions.

**5-HT_2C_ receptor:** competition binding experiments were carried out in membranes from CHO-5-HT_2C_ (a generous gift from William P Clarke, University of San Antonio, Texas) cells. On the day of assay, membranes were defrosted and re-suspended in incubation buffer (50 mM Tris-HCl, pH = 7.5). Each reaction well of a GF/C multiscreen plate (Millipore), prepared in duplicate, contained 10 µg of protein, 4 nM [^3^H]mesulergin and test compound. Reaction was incubated for 30 min at 37^o^ C, then samples were vacuum filtered and plate washed 4 times with 4^o^ C wash buffer (50 mM Tris-HCl, pH = 6.6). Non-specific binding was determined in the presence of 1 µM mianserin.

**5-HT_4_ receptor:** competition binding experiments were carried out in membranes from HeLa-5-HT_4_ (human 5-HT_4_ cDNA subcloned into pcDNA3 transfected in HeLa cell line from ATCC) cells. On the day of assay, membranes were defrosted and re-suspended in incubation buffer (25 mM Tris-HCl, pH = 7.4). Each reaction well of a GF/C multiscreen plate (Millipore), prepared in duplicate, contained 10 µg of protein, 0.5 nM [^3^H]GR113808 and test compound. Reaction was incubated for 90 min at 25^o^ C, then samples were vacuum filtered and plate washed 4 times with 4^o^ C wash buffer (25 mM Tris-HCl, pH = 7.4). Non-specific binding was determined in the presence of 10 µM GR113808.

**5-HT_6_ receptor:** competition binding experiments were carried out in membranes from HEK-5-HT_6_ cells (human 5-HT_6_ cDNA subcloned into pcDNA3 transfected in HEK cell line from ATCC). On the day of assay, membranes were defrosted and re-suspended in incubation buffer (50 mM Tris-HCl, 10 mM MgCl_2_, 0.5 mM EDTA pH = 7.4). Each reaction well of a GF/C multiscreen plate (Millipore), prepared in duplicate, contained 5 µg of protein, 3 nM [^3^H]LSD and test compound. Reaction was incubated for 30 min at 37^o^ C, then samples were vacuum filtered and plate washed 4 times with 4^o^ C wash buffer (50 mM Tris-HCl, pH = 7.4). Non-specific binding was determined in the presence of 100 µM serotonin.

**5-HT_7_ receptor:** competition binding experiments were carried out in membranes from HEK-293-5-HT_7_ (human 5-HT_7_ cDNA subcloned into pcDNA3 transfected in HEK-293 cell line from ATCC) cells. On the day of assay, membranes were defrosted and re-suspended in incubation buffer (50 mM Tris-HCl, 4 mM CaCl_2_, 1 mM ascorbic acid, 0.1 mM pargyline, pH = 7.4). Each reaction well of a GF/C multiscreen plate (Millipore), prepared in duplicate, contained 5 µg of protein, 2 nM [^3^H]SB269970 and test compound. Reaction was incubated for 30 min at 37^o^ C, then samples were vacuum filtered and plate washed 4 times with 4^o^ C wash buffer (50 mM Tris-HCl, pH = 7.4). Non-specific binding was determined in the presence of 25 µM clozapine.

**D_1A_ receptor:** competition binding experiments were carried out in membranes from Perkin Elmer. On the day of assay, membranes were defrosted and re-suspended in incubation buffer (50 mM Tris-HCl, 5 mM MgCl_2_, pH = 7.4). Each reaction well of a GF/C multiscreen plate (Millipore), prepared in duplicate, contained 12 µg of protein, 0.7 nM [^3^H]SCH-23390 and test compound. Reaction was incubated for 60 min at 27^o^ C, then samples were vacuum filtered and plate washed 4 times with 4^o^ C wash buffer (50 mM Tris-HCl, pH = 7.4). Non-specific binding was determined in the presence of 1 µM butaclamol.

**D_2_ receptor:** competition binding experiments were carried out in membranes from CHO-D_2_ cell line (human D_2_ cDNA subcloned into pcDNA3 transfected in CHO cell line from ATCC). On the day of assay, membranes were defrosted and re-suspended in incubation buffer (50 mM Tris-HCl, 120 mM NaCl, 5 mM KCl, 5 mM MgCl_2_, 1 mM EDTA, pH = 7.4). Each reaction well of a GF/C multiscreen plate (Millipore), prepared in duplicate, contained 20 µg of protein, 0.2 nM [^3^H]spiperone and test compound. Reaction was incubated for 120 min at 25^o^ C, then samples were vacuum filtered and plate washed 4 times with 4^o^ C wash buffer (50 mM Tris-HCl, 0.9% NaCl, pH = 7.4). Non-specific binding was determined in the presence of 10 µM sulpiride.

**D_3_ receptor:** competition binding experiments were carried out in membranes from Perkin Elmer. On the day of assay, membranes were defrosted and re-suspended in incubation buffer (50 mM Tris-HCl, 5 mM MgCl_2_, pH = 7.4). Each reaction well of a GF/C multiscreen plate (Millipore), prepared in duplicate, contained 2 µg of protein, 1 nM [^3^H]spiperone and test compound. Reaction was incubated for 60 min at 25^o^ C, then samples were vacuum filtered and plate washed 4 times with 4^o^ C wash buffer (50 mM Tris-HCl, pH = 7.4). Non-specific binding was determined in the presence of 1 µM haloperidol.

**D_4_ receptor:** competition binding experiments were carried out in membranes from Perkin Elmer. On the day of assay, membranes were defrosted and re-suspended in incubation buffer (50 mM Tris-HCl, 120 mM NaCl, 5 mM KCl, 5 mM MgCl_2_, 1 mM EDTA, pH = 7.4). Each reaction well of a GF/C multiscreen plate (Millipore), prepared in duplicate, contained 35 µg of protein, 1 nM [^3^H]spiperone and test compound. Reaction was incubated for 120 min at 27^o^ C, then samples were vacuum filtered and plate washed 4 times with 4^o^ C wash buffer (50 mM Tris-HCl, 0.9% NaCl, pH = 7.4). Non-specific binding was determined in the presence of 25 µM haloperidol.

**H_1_ receptor:** competition binding experiments were carried out in membranes from Perkin-Elmer. On the day of assay, membranes were defrosted and re-suspended in incubation buffer (50 mM Tris-HCl, 5 mM MgCl_2_, pH = 7.4). Each reaction well of a GF/C multiscreen plate (Millipore), prepared in duplicate, contained 5 µg of protein, 1.5 nM [^3^H]pyrilamine and test compound. Reaction was incubated for 60 min at 27^o^ C, then samples were vacuum filtered and plate washed 4 times with 4^o^ C wash buffer (50 mM Tris-HCl, pH = 7.4). Non-specific binding was determined in the presence of 10 µM triprolidine.

**H_2_ receptor:** competition binding experiments were carried out in membranes from Perkin-Elmer. On the day of assay, membranes were defrosted and re-suspended in incubation buffer (50 mM Tris-HCl, 0.3% BSA, pH = 7.4). Each reaction well of a GF/C multiscreen plate (Millipore), prepared in duplicate, contained 10 µg of protein, 15 nM [^3^H]tiotidine and test compound. Reaction was incubated for 90 min at 27^o^ C, then samples were vacuum filtered and plate washed 4 times with 4^o^ C wash buffer (50 mM Tris-HCl, pH = 7.4). Non-specific binding was determined in the presence of 10 µM cimetidine.

**M_1_ receptor:** competition binding experiments were carried out in membranes from CHO-M_1_ cell line ((human M_1_ cDNA subcloned into pcDNA3 transfected in CHO cell line from ATCC)). On the day of assay, membranes were defrosted and re-suspended in incubation buffer (50 mM HEPES, 5 mM MgCl_2_, 1 mM CaCl_2_, 0.2% BSA, pH = 7.4). Each reaction well of a GF/C multiscreen plate (Millipore), prepared in duplicate, contained 60 µg of protein, 5 nM [^3^H]pirenzepine and test compound. Reaction was incubated for 90 min at 25^o^ C, then samples were vacuum filtered and plate washed 4 times with 4^o^ C wash buffer (50 mM HEPES, 500 mM NaCl, 0.1% BSA, pH = 7.4). Non-specific binding was determined in the presence of 200 µM pirenzepine.

**M_2_ receptor:** competition binding experiments were carried out in membranes from Perkin Elmer. On the day of assay, membranes were defrosted and re-suspended in incubation buffer (Phosphate buffered saline, pH = 7.4). Each reaction well of a GF/C multiscreen plate (Millipore), prepared in duplicate, contained 5 µg of protein, 1 nM [^3^H]scopolamine and test compound. Reaction was incubated for 120 min at 27^o^ C, then samples were vacuum filtered and plate washed 4 times with 4^o^ C wash buffer (50 mM Tris-HCl, 154 mM NaCl, pH = 7.4). Non-specific binding was determined in the presence of 5 µM atropine.

**M_3_ receptor:** competition binding experiments were carried out in membranes from Perkin Elmer. On the day of assay, membranes were defrosted and re-suspended in incubation buffer (phosphate buffered saline, pH = 7.4). Each reaction well of a GF/C multiscreen plate (Millipore), prepared in duplicate, contained 40 µg of protein, 0.6 nM [^3^H]scopolamine and test compound. Reaction was incubated for 120 min at 27^o^ C, then samples were vacuum filtered and plate washed 4 times with 4^o^ C wash buffer (50 mM Tris-HCl, 154 mM NaCl, pH = 7.4). Non-specific binding was determined in the presence of 5 µM atropine.

**α_1A_ receptor:** competition binding experiments were carried out in membranes from HEK-α_1A_ cells (human α_1A_ cDNA subcloned into pcDNA3 transfected in HEK-293 cell line from ATCC). On the day of assay, membranes were defrosted and re-suspended in incubation buffer (50 mM HEPES, 5 mM MgCl_2_, 1 mM CaCl_2_, 0.2% BSA, pH = 7.4). Each reaction well of a GF/C multiscreen plate (Millipore), prepared in duplicate, contained 10 µg of protein, 1 nM [^3^H]prazosin and test compound. Reaction was incubated for 90 min at 25^o^ C, then samples were vacuum filtered and plate washed 4 times with 4^o^ C wash buffer (50 mM HEPES, 500 mM NaCl, pH = 7.4). Non-specific binding was determined in the presence of 1 µM prazosin.

**α_2A_ receptor:** competition binding experiments were carried out in membranes from CHO-α_2A_ cells (human α_2A_ cDNA subcloned into pcDNA3 transfected in HEK-293 cell line from ATCC). On the day of assay, membranes were defrosted and re-suspended in incubation buffer (25 mM Na_2_PO_4_, pH = 7.4). Each reaction well of a GF/C multiscreen plate (Millipore), prepared in duplicate, contained 30 µg of protein, 0.37 nM [^3^H]MK-912 and test compound. Reaction was incubated for 30 min at 25^o^ C, then samples were vacuum filtered and plate washed 4 times with 4^o^ C wash buffer (25 mM Na_2_PO_4_, pH = 7.4). Non-specific binding was determined in the presence of 100 µM Norepinephrine.

**α_2B_ receptor:** competition binding experiments were carried out in membranes from Perkin-Elmer. On the day of assay, membranes were defrosted and re-suspended in incubation buffer (50 mM Tris-HCl, 1 mM EDTA, pH = 7.4). Each reaction well of a GF/C multiscreen plate (Millipore), prepared in duplicate, contained 5 µg of protein, 2.5 nM [^3^H]rauwolscine and test compound. Reaction was incubated for 60 min at 27^o^ C, then samples were vacuum filtered and plate washed 4 times with 4^o^ C wash buffer (50 mM Tris-HCl, pH = 7.4). Non-specific binding was determined in the presence of 50 µM UK10,304.

**α_2C_ receptor:** competition binding experiments were carried out in membranes from Perkin-Elmer. On the day of assay, membranes were defrosted and re-suspended in incubation buffer (50 mM Tris-HCl, 1 mM EDTA, pH = 7.4). Each reaction well of a GF/C multiscreen plate (Millipore), prepared in duplicate, contained 5 µg of protein, 0.65 nM [^3^H]rauwolscine and test compound. Reaction was incubated for 60 min at 27^o^ C, then samples were vacuum filtered and plate washed 4 times with 4^o^ C wash buffer (50 mM Tris-HCl, pH = 7.4). Non-specific binding was determined in the presence of 10 µM prazosin.

**δ-opioid receptor:** competition binding experiments were carried out in membranes from CHO-delta opioid cell line (human delta-opioid cDNA subcloned into pcDNA3 transfected in CHO cell line from ATCC). On the day of assay, membranes were defrosted and re-suspended in incubation buffer (50 mM Tris-HCl, pH = 7.4). Each reaction well of a GF/C multiscreen plate (Millipore), prepared in duplicate, contained 10 µg of protein, 5 nM [^3^H]enkephalin and test compound. Reaction was incubated for 90 min at 25^o^ C, then samples were vacuum filtered and plate washed 4 times with 4^o^ C wash buffer (50 mM Tris-HCl, pH = 7.4). Non-specific binding was determined in the presence of 10 µM DPDPE.

**k-opioid receptor:** competition binding experiments were carried out in membranes from Millipore. On the day of assay, membranes were defrosted and re-suspended in incubation buffer (50 mM Tris-HCl, pH = 7.4). Each reaction well of a GF/C multiscreen plate (Millipore), prepared in duplicate, contained 35 µg of protein, 0.3 nM [^3^H]diprenorphine and test compound. Reaction was incubated for 90 min at 25^o^ C, then samples were vacuum filtered and plate washed 4 times with 4^o^ C wash buffer (50 mM Tris-HCl, pH = 7.4). Non-specific binding was determined in the presence of 200 µM U69-593.

**µ-opioid receptor:** competition binding experiments were carried out in membranes from Perkin Elmer. On the day of assay, membranes were defrosted and re-suspended in incubation buffer (50 mM Tris-HCl, 5 mM MgCl_2_, pH = 7.4). Each reaction well of a GF/C multiscreen plate (Millipore), prepared in duplicate, contained 15 µg of protein, 0.75 nM [^3^H]DAMGO and test compound. Reaction was incubated for 60 min at 27^o^ C, then samples were vacuum filtered and plate washed 4 times with 4^o^ C wash buffer (50 mM Tris-HCl, pH = 7.4). Non-specific binding was determined in the presence of 10 µM naloxone.

**Columns IB-IC:** Swamidass SJ and Hughes TB group (data presented first in this paper)**.** Predictions of compound glutathione reactivity and epoxidation potential: Each molecule of the MB was tested with two recently published models that predict molecule reactivity and epoxidation potential [167, 168]. In the combined spreadsheet, the contributed columns are labeled “Molecule Glutathione Reactivity Prediction Score Swamidass” and “Molecule Epoxidation Prediction Score Swamidass”. The glutathione reactivity prediction scores range from 0 to 1, and reflect the probability that each molecule will be reactive with glutathione. Reactivity is potentially important for two reasons. First, reactivity may be the actual mechanism of action of a drug. Secondly, highly reactive molecules may unexpectedly conjugate to nucleophilic sites, potentially skewing assay results. The molecule epoxidation prediction scores range from 0 to 1, and reflect the probability that each molecule will form an epoxide due to metabolism. Epoxides are frequently highly reactive due to ring tension and polarized carbon-oxygen bonds, and consequently epoxide metabolites are often responsible for drug toxicity. Furthermore, epoxidation predictions can potentially direct rational drug modification to prevent epoxidation, thereby eliminating an important toxicity risk. Results: several molecules are predicted to be epoxidized with high confidence. For example, MMV006188 and MMV666105 have very high Molecule Epoxidation Prediction Scores. Epoxidation was predicted to occur on MMV006188’s furan, and on MMV666105’s thiophene**.** For either molecule, it may be possible to make a small structural change to reduce the chances of epoxidation. For instance, adding a methyl group to the 2 or 3 site on the furan or thiophene ring might increase the chances of hydroxylation instead of epoxidation. Similarly, several molecules were predicted to be reactive, for example MMV007199 and MMV666691. Both molecules share the same core structure, and contain a highly predicted Michael acceptor motif, which is well-known to be often reactive.

**Columns IE-KK.** NCI: Misra group (data presented first in this paper). NCI60 anti-tumor cell activity. Methods: The *in vitro* NCI60 Cell Line Screen is designed to evaluate agents for antiproliferative activity in a panel of 60 well-characterized human tumor cell lines representing leukemia, melanoma and cancers of the lung, colon, brain, ovary, breast, prostate, and kidney (as described previously [169, 170], and on the NCI/DTP website: <https://dtp.cancer.gov/index.html>). These 60 cell lines, along with others, have been developed at NCI and have been maintained and distributed for research purposes by NCI for over 25 years. The experimental readout provides both inhibition of cell growth and cell kill. The screen was originally designed to prioritize agents for further evaluation. For the MB project, compounds were evaluated for activity at a single dose of 10 μM with a drug exposure period of 48 hours. The number reported for the 1-dose assay is growth relative to the no-drug control, and relative to the time zero number of cells. This allows detection of both growth inhibition (values between 0 and 100) and lethality (values less than 0). This is the same as for the 5-dose assay, described on http://dtp.cancer.gov/branches/btb/ivclsp.html. For example, a value of 100 means no growth inhibition. A value of 40 would mean 60% growth inhibition. A value of 0 means no net growth over the course of the experiment, a value of -40 means 40% lethality, a value of -100 means complete lethality (*i.e.* all cells are dead). All 400 compounds were examined in the 1-dose assay at 10 µM. Testing beyond the initial screen: There were 133 compounds that passed threshold to proceed to 5-dose (dose-response) (data are available for the 133 compounds at the DTP public website (dtp.cancer.gov) by searching either the MMV number or NSC number). After examination of the 5-dose data, 10 compounds were selected for repeat testing based generally on potency, differential cell activity or activity on an individual cell lines or cell line panel. A summary of the data for the 10 compounds of interest is in the table below.

| **MMV Number** | **NSC Number** | **MGLI50** | **Notes** |
| --- | --- | --- | --- |
| MMV007384 | 766470 | -7.34 | Colon, differential, potent |
| MMV019074 | 766523 | -4.91 | Specific lines sensitive |
| MMV665803 | 766546 | -5.01 |  |
| MMV665796 | 766556 | -5.00 |  |
| MMV020275 | 766585 | -4.93 |  |
| MMV000760 | 766634 | -6.02 | Differential |
| MMV666020 | 766672 | -4.90 |  |
| MMV665969 | 766692 | -6.29 |  |
| MMV666597 | 766722 | -5.57 | Differential |
| MMV006962 | 766755 | -5.61 | Differential, CNS |

MGLI50 = Mean Growth Log I_50_

A single compound, MMV007384 (NSC766470), was selected for *in vivo* evaluation in the mouse hollow-fibre assay based on potency, differential cell activity and activity in the colon tumour cell panel. This testing is on-going.

**Metabolomics with the Malaria Box** (Not in the HeatMap but in Fig 2a & S5, Llinás group. (data presented first in this paper)): To identify the mode of action (MoA) of the anti-malarial drugs in the MMV Malaria Box and define the relevant target biochemical pathways in the malaria parasite that are perturbed by these drugs, we used metabolomic fingerprint profiling to characterize hundreds of small molecule metabolites from parasitized red blood cells (RBCs purchased from Biological Specialty Corporation, Colmar, PA, was infected with the 3D7 strain) by high resolution liquid chromatography-mass spectrometry (LC-MS). Our method provides sufficient coverage to interrogate a large number of parasite-derived metabolic processes including diagnostic compounds for each of the following pathways: glycolysis, pentose shunt, tricarboxylic acid cycle, amino acid metabolism, nucleotide biosynthesis, fatty acid metabolism, redox balancing, and haemoglobin catabolism. Since many clinically approved pharmaceuticals target cellular metabolic processes, this global LC-MS approach allows us to capture a comprehensive snapshot of perturbations in cellular metabolism due to any drug. This methodology allows us to: 1) compare differences in metabolic profiles of parasites exposed to anti-malarial drugs, 2) establish which compounds act directly on metabolic processes (enzymes) in the parasite 3) compare perturbations to the metabolic network among unrelated chemical classes of molecules, which can be used to categorize them by their MoA 4) predict and test possible effective combinations of drugs targeting disparate pathways (less likely to develop resistance), and importantly, 5) make recommendations of which drugs likely target the same pathways.

Metabolomics methods were adapted from a previously published protocol [171]. Briefly, blood stage 3D7 *Plasmodium falciparum* parasites were cultured using standard methods at 2% hematocrit and 10% parasitemia and synchronized with sorbitol. Trophozoite stage parasites were magnetically purified to >95% parasitemia with a MACS column [172] and subsequently incubated for 1-2 hours at ~0.1% hematocrit to recover. Parasites were then incubated with drug at between 2 and 50 times the IC_50_, subject to limitations in drug volume (see supplemental data file for exact concentrations used), for 2.5 hours with three technical replicates. A negative control with no drug added and a positive control using the clinical antimalarial atovaquone was performed for each drug test. After drug incubation, samples were extracted with 90% methanol with 5 µM isotopically labeled aspartate added as a control. Extracts were analyzed in negative ion mode by ultra-high performance liquid chromatography-mass spectrometry (uHPLC-MS) on a Thermo Exactive mass spectrometer with a reversed phase C18 column using a methanol and acetic acid gradient and a tributylamine ion pairing agent [173]. Pure standards for a library of several hundred metabolites of interest have been run previously to determine retention times for each compound. Mass and retention time were used to identify peaks associated with metabolites of interest for targeted peak detection in MAVEN [174, 175]. The R statistical environment [176] was used to merge data, blank subtract, calculate ^2^log fold changes, and generate a heat map of metabolic profiles for each drug. Supplemental data file S5 shows log2 fold changes compared to an untreated control. Positive and negative values indicate increase and decrease, respectively, as compared to the control.

We have found that drugs that target the metabolic network of the malaria parasite produce characteristic responses that are related to the mode of action of these compounds. Here we present the results of screening all of the compounds in Plate A from the MMV Malaria Box library for metabolic phenotypes using our metabolic profiling methods (Figure 2a). We have also measured the metabolic response of *P. falciparum* to a dihydroorotate dehydrogenase inhibitor (DSM-1) and an electron transport chain inhibitor (atovaquone) and chloroquine, which has a non-metabolic target. We found that the drug-induced metabolic perturbations were localized to the pathway intermediates N-carbamoyl aspartate and dihydroorotate immediately upstream of the target enzymes for DSM-1 (data not shown) and atovaquone (Figure 2a), but no major changes were seen for chloroquine (data not shown). Interestingly, a number of Plate A Malaria Box compounds show a metabolic fingerprint very analogous to that of atovaquone and DSM-1 with a clear phenotype in the pyrimidine biosynthesis pathway, which we call “atovaquone-like” (Figure 2a). The clear metabolic signature of atovaquone makes it an effective positive control for every drug treatment regime. We also find that all Plate A compounds identified by Lehane et al. [4]) as targeting *Pf*ATP4 cluster together and are distinguished by the accumulation of dNTPs and a general decrease in haemoglobin-derived peptides (Figure 2a) further supporting a common mode of action for these compounds.

**Chemogenomics approach with pB knockout strategy (**not in heatmap but data in Fig 2B and S4)**.** Adams/Kyle/Jiang/Ferdig group (data presented first in this paper). Methods: A collection of *Plasmodium falciparum* single insertion piggyBac mutants were profiled with a collection of known and unknown inhibitors for changes in IC_50_ relative to the wild-type parent NF54. Pairwise genotype-phenotype associations based on the response profiles of these compounds and the individual mutants created chemogenomic profiles that were used to determine if the mechanism of action of compounds were unique or similar. A subset of mutants, including a mutant of the K13-propeller gene, had similar shifts in drug response profiles of increased sensitivity to ART compounds, identifying them as an ART sensitivity cluster. This ART sensitivity cluster and several piggyBac mutants with dissimilar responses to ART compounds were used to profile 48 Malaria Box compounds to identify Malaria Box compounds that may share a similar mechanism of action with ART compounds, identifying 5 of the 50 Malaria Box screened (MMV006087, MMV006427, MMV020492, MMV665876, MMV396797) as having similar drug-drug chemogenomic profiles to the ART sensitivity cluster. Summary of preliminary pB chemogenomic screen of the MMV Malaria Box. Introduction: Our chemogenomic study of *P. falciparum* uses a similar approach to the National Cancer Institute NCI60 program. The NCI60 program operated for many years to screen new compounds for efficacy (IC_50_) against the same collection of 60 different cancer cell lines [169]. Drugs and chemical inhibitors with similar mechanisms of action share similar drug response profiles; therefore, unknown compounds could be defined by pair-wise comparison of responses with known drugs. Overview of method: In a similar approach an isogonics panel *P. falciparum* mutants, created by random insertional mutagenesis with the transposon *piggyBac*, was used to profile drug responses of the Malaria Box compounds. Each *P. falciparum piggyBac* (pB) mutant carried a single transposon randomly inserted into its genome and its precise chromosomal location was determined by sequence analysis. The mutation created by the transposon is expected to alter the function (s) of the metabolic processes and pathways linked to the genetic locus (or loci) at the insertion site. These alterations in normal metabolic processes are expected to increase or decrease drug sensitivity to compounds that have a mechanism of action associated with the affected processes and pathways in each pB mutant. Changes in IC_50_ of the pB mutants are calibrated relative to IC_50_’s of the wild-type parent line of NF54. In addition to being able to define drug:drug relations ships, this chemogenomic study of *P. falciparum* can functionally link hypothetical or unknown genes to specific biochemical and metabolic processes by pair-wise associations of drug responses between mutants with known genetic mutations. Results: Forty-seven unique pB mutant clones were used in this chemogenomic profiling study to evaluate drug responses for 49 MMV Malaria Box compounds relative to some common antimalarial drugs, including positive control artemisinin compounds (AS, AQ, AM). An IC_50_ was calculated for each pB mutant:drug combination relative to the parental clone NF54 to create a chemogenomic profile for each drug. Pairwise cluster analysis was used to correlate the Malaria Box compounds (Figure 2b).

**ChEMBL data repository**

ChEMBL has arguably transformed the landscape of the available medicinal chemistry and chemical biology data. In terms of contents, ChEMBL covers a broad range of curated and annotated data, manually extracted from the primary medicinal chemistry literature [177-179]. The data include experimental biological readouts, such as binding, functional and absorption, distribution, metabolism, and excretion assay measurements, standardized to common units where possible, and indexed 2D chemical structures, along with curated linkage to the biological targets and source species. The targets range from single proteins, to protein complexes, then tissues and finally whole organism *in vivo* data. In addition to the literature-extracted information, ChEMBL also integrates deposited screening results from other public databases (*e.g.*, PubChem Bioassay), along with information on approved drugs and their likely efficacy targets. The ChEMBL database is updated on a regular basis and, as of August 2015, the current release (version 20) contains more than 13.5 million assay measurements for more than 1.4 million distinct compounds tested against more than 10,500 biological targets.

ChEMBL serves also an open data-sharing hub for the crucial field of neglected tropical diseases (NTD) research. In particular, ChEMBL hosts a series of special data sets related to tropical pathogens in its ChEMBL-NTD pages [www.ebi.ac.uk/chemblntd]. The data sets are derived from high-throughput screening campaigns, typically against whole organisms such as *Plasmodia*, *Trypanosoma* and *Mycobacteria* species, from organizations such as GlaxoSmithKline, Novartis-GNF, St. Jude Children’s Research Hospital, Harvard, and the Drugs for Neglected Diseases *Initiative* (DND*i*). Moreover, these data sets are fully integrated within the ChEMBL database. Specifically for Malaria research, ChEMBL, in collaboration with the Medicines for Malaria Venture (MMV) hosts screening results from the Open Source Malaria project [http://opensourcemalaria.org], as well as the MMV Malaria Box [180], a physical set of 400 probe- and drug-like compounds with confirmed antimalarial activity. A large number of depositions from academic and industrial laboratories around the world has been already submitted and are available in ChEMBL. Importantly, the data are open and freely shareable by everyone, while their provenance is visible and appropriately acknowledged.

ChEMBL 20 [www.ebi.ac.uk/chembldb/] contains 19 depositions with screening data against the MMV Malaria Box compounds carried out by both academic and industrial groups. Static DOIs and ChEMBL IDs are for individual depositions are listed in the spreadsheet. The depositions include a total of 135 distinct assays - an average of 7 assays per deposition. Users can list these assays on the ChEMBL interface [https://www.ebi.ac.uk/chembldb/] by typing ‘MMV:’ on the search box and then clicking the ‘Assays’ button. These 135 assays contain an impressive 44,745 activity data points (an average of 331 data points per assay), fully curated, standardized and integrated in the ChEMBL database. The individual assay activities are summarised in several charts

(*e.g.* www.ebi.ac.uk/chembl/assay/inspect/CHEMBL2028070) and all data are available to download via the interface.

**Ethics Statements:**

Animals (mice) were required in the WuXi PK experiments and animal ethics approval was provided by WuXi AppTec (Shanghai) Institutional Animal Care and Use Committee Protocol # 20120201-mouse. This review and approval adheres to the China National Standard for Laboratory Animal Care and Use.

Animals were also required in the Bickle group’s propagation of schistosomes. Experimentation was carried out using the NC3Rs and ARRIVE guidelines under the United Kingdom Animal’s Scientific Procedures Act 1986 (under project licence 60/4456) with approval from the London School of Hygiene and Tropical Medicine Ethics committee.

Blood for RBCs was purchased from Biological Specialty Corporation, Colmar, PA, by the Llinás group as approved by the Pennsylvania State University Institutional Biosafety Committee Continued Approval IBC#47097 (Expiration Date May 12, 2017). Annual approval is required.

**References**

1. Matlock MK, Zaretzki JM, Swamidass SJ. Scaffold network generator: a tool for mining molecular structures. Bioinformatics. 2013;29(20):2655-6. Epub 2013/08/07. doi: 10.1093/bioinformatics/btt448. PubMed PMID: 23918250.

2. Swamidass SJ, Baldi P. Mathematical correction for fingerprint similarity measures to improve chemical retrieval. J Chem Inf Model. 2007;47(3):952-64. Epub 2007/04/21. doi: 10.1021/ci600526a. PubMed PMID: 17444629.

3. Swamidass SJ, Chen J, Bruand J, Phung P, Ralaivola L, Baldi P. Kernels for small molecules and the prediction of mutagenicity, toxicity and anti-cancer activity. Bioinformatics. 2005;21 Suppl 1:i359-68. Epub 2005/06/18. doi: 10.1093/bioinformatics/bti1055. PubMed PMID: 15961479.

4. Lehane AM, Ridgway MC, Baker E, Kirk K. Diverse chemotypes disrupt ion homeostasis in the malaria parasite. Mol Microbiol. 2014;94(2):327-39. doi: 10.1111/mmi.12765. PubMed PMID: 25145582.

5. Spillman NJ, Allen RJ, McNamara CW, Yeung BK, Winzeler EA, Diagana TT, et al. Na(+) regulation in the malaria parasite Plasmodium falciparum involves the cation ATPase PfATP4 and is a target of the spiroindolone antimalarials. Cell host & microbe. 2013;13(2):227-37. Epub 2013/02/19. doi: 10.1016/j.chom.2012.12.006. PubMed PMID: 23414762; PubMed Central PMCID: PMCPMC3574224.

6. Saliba KJ, Kirk K. pH regulation in the intracellular malaria parasite, Plasmodium falciparum. H(+) extrusion via a V-type H(+)-ATPase. J Biol Chem. 1999;274(47):33213-9. Epub 1999/11/24. PubMed PMID: 10559194.

7. Rottmann M, McNamara C, Yeung BK, Lee MC, Zou B, Russell B, et al. Spiroindolones, a potent compound class for the treatment of malaria. Science. 2010;329(5996):1175-80. doi: 10.1126/science.1193225. PubMed PMID: 20813948; PubMed Central PMCID: PMC3050001.

8. Flannery EL, McNamara CW, Kim SW, Kato TS, Li F, Teng CH, et al. Mutations in the P-type cation-transporter ATPase 4, PfATP4, mediate resistance to both aminopyrazole and spiroindolone antimalarials. ACS Chem Biol. 2015;10(2):413-20. Epub 2014/10/17. doi: 10.1021/cb500616x. PubMed PMID: 25322084; PubMed Central PMCID: PMCPmc4340351.

9. Vaidya AB, Morrisey JM, Zhang Z, Das S, Daly TM, Otto TD, et al. Pyrazoleamide compounds are potent antimalarials that target Na^+^ homeostasis in intraerythrocytic *Plasmodium falciparum*. Nature communications. 2014;5:5521. Epub 2014/11/26. doi: 10.1038/ncomms6521. PubMed PMID: 25422853.

10. Jimenez-Diaz MB, Ebert D, Salinas Y, Pradhan A, Lehane AM, Myrand-Lapierre ME, et al. (+)-SJ733, a clinical candidate for malaria that acts through ATP4 to induce rapid host-mediated clearance of Plasmodium. Proc Natl Acad Sci U S A. 2014. Epub 2014/12/03. doi: 10.1073/pnas.1414221111. PubMed PMID: 25453091.

11. Krishna S, Woodrow C, Webb R, Penny J, Takeyasu K, Kimura M, et al. Expression and functional characterization of a *Plasmodium falciparum* Ca^2+^-ATPase (*Pf*ATP4) belonging to a subclass unique to apicomplexan organisms. J Biol Chem. 2001;276(14):10782-7. doi: 10.1074/jbc.M010554200. PubMed PMID: 11145964.

12. Ginsburg H, Kutner S, Krugliak M, Cabantchik ZI. Characterization of permeation pathways appearing in the host membrane of Plasmodium falciparum infected red blood cells. Mol Biochem Parasitol. 1985;14(3):313-22. Epub 1985/03/01. PubMed PMID: 3887158.

13. Kirk K, Horner HA, Elford BC, Ellory JC, Newbold CI. Transport of diverse substrates into malaria-infected erythrocytes via a pathway showing functional characteristics of a chloride channel. J Biol Chem. 1994;269(5):3339-47. Epub 1994/02/04. PubMed PMID: 8106373.

14. Staines HM, Ellory JC, Kirk K. Perturbation of the pump-leak balance for Na(+) and K(+) in malaria-infected erythrocytes. Am J Physiol Cell Physiol. 2001;280(6):C1576-87. Epub 2001/05/15. PubMed PMID: 11350753.

15. van Pelt-Koops JC, Pett HE, Graumans W, van der Vegte-Bolmer M, van Gemert GJ, Rottmann M, et al. The spiroindolone drug candidate NITD609 potently inhibits gametocytogenesis and blocks *Plasmodium falciparum* transmission to anopheles mosquito vector. Antimicrob Agents Chemother. 2012;56(7):3544-8. Epub 04/18. doi: AAC.06377-11 [pii] 10.1128/AAC.06377-11. PubMed PMID: 22508309.

16. Ruecker A, Mathias DK, Straschil U, Churcher TS, Dinglasan RR, Leroy D, et al. A male and female gametocyte functional viability assay to identify biologically relevant malaria transmission-blocking drugs. Antimicrob Agents Chemother. 2014. doi: 10.1128/AAC.03666-14. PubMed PMID: 25267664.

17. Lehane AM, Saliba KJ, Allen RJ, Kirk K. Choline uptake into the malaria parasite is energized by the membrane potential. Biochem Biophys Res Commun. 2004;320(2):311-7. Epub 2004/06/29. doi: 10.1016/j.bbrc.2004.05.164. PubMed PMID: 15219828.

18. Carter R, Nijhout MM. Control of gamete formation (exflagellation) in malaria parasites. Science. 1977;195(4276):407-9. Epub 1977/01/28. PubMed PMID: 12566.

19. Ahyong V, Sheridan CM, Leon KE, Witchley JN, Diep J, DeRisi JL. Identification of Plasmodium falciparum specific translation inhibitors from the MMV Malaria Box using a high throughput in vitro translation screen. Malar J. 2016;15(1):173. Epub 2016/03/19. doi: 10.1186/s12936-016-1231-8. PubMed PMID: 26987601; PubMed Central PMCID: PMCPmc4794828.

20. Florent I, Derhy Z, Allary M, Monsigny M, Mayer R, Schrevel J. A Plasmodium falciparum aminopeptidase gene belonging to the M1 family of zinc-metallopeptidases is expressed in erythrocytic stages. Mol Biochem Parasitol. 1998;97(1-2):149-60. Epub 1999/01/08. PubMed PMID: 9879894.

21. Allary M, Schrevel J, Florent I. Properties, stage-dependent expression and localization of Plasmodium falciparum M1 family zinc-aminopeptidase. Parasitology. 2002;125(Pt 1):1-10. Epub 2002/08/09. PubMed PMID: 12166515.

22. Wilkes SH, Prescott JM. The slow, tight binding of bestatin and amastatin to aminopeptidases. J Biol Chem. 1985;260(24):13154-62. Epub 1985/10/25. PubMed PMID: 2865258.

23. Paiardini A, Bamert RS, Kannan-Sivaraman K, Drinkwater N, Mistry SN, Scammells PJ, et al. Screening the Medicines for Malaria Venture "Malaria Box" against the Plasmodium falciparum Aminopeptidases, M1, M17 and M18. PLoS One. 2015;10(2):e0115859. Epub 2015/02/24. doi: 10.1371/journal.pone.0115859. PubMed PMID: 25700165; PubMed Central PMCID: PMCPMC4336144.

24. Ferreira RS, Simeonov A, Jadhav A, Eidam O, Mott BT, Keiser MJ, et al. Complementarity between a docking and a high-throughput screen in discovering new cruzain inhibitors. J Med Chem. 2010;53(13):4891-905. Epub 2010/06/15. doi: 10.1021/jm100488w. PubMed PMID: 20540517; PubMed Central PMCID: PMCPmc2895358.

25. Crowther GJ, Hillesland HK, Keyloun KR, Reid MC, Lafuente-Monasterio MJ, Ghidelli-Disse S, et al. Biochemical screening of five protein kinases from *Plasmodium falciparum* against 14,000 cell-active compounds PLoS ONE. 2016;DOI: 10.1371/journal.pone.0149996.

26. Ojo KK, Pfander C, Mueller NR, Burstroem C, Larson ET, Bryan CM, et al. Transmission of malaria to mosquitoes blocked by bumped kinase inhibitors. J Clin Invest. 2012;122(6):2301-5. Epub 2012/05/09. doi: 10.1172/jci61822. PubMed PMID: 22565309; PubMed Central PMCID: PMCPmc3366411.

27. Murphy JM, Zhang Q, Young SN, Reese ML, Bailey FP, Eyers PA, et al. A robust methodology to subclassify pseudokinases based on their nucleotide-binding properties. Biochem J. 2014;457(2):323-34. Epub 2013/10/11. doi: 10.1042/bj20131174. PubMed PMID: 24107129.

28. Koresawa M, Okabe T. High-throughput screening with quantitation of ATP consumption: a universal non-radioisotope, homogeneous assay for protein kinase. Assay Drug Dev Technol. 2004;2(2):153-60. Epub 2004/05/29. doi: 10.1089/154065804323056495. PubMed PMID: 15165511.

29. Liu L, Richard J, Kim S, Wojcik EJ. Small molecule screen for candidate antimalarials targeting Plasmodium Kinesin-5. J Biol Chem. 2014;289(23):16601-14. Epub 2014/04/17. doi: 10.1074/jbc.M114.551408. PubMed PMID: 24737313; PubMed Central PMCID: PMCPmc4047425.

30. Ortiz D, Guiguemde WA, Johnson A, Elya C, Anderson J, Clark J, et al. Identification of Selective Inhibitors of the Plasmodium falciparum Hexose Transporter PfHT by Screening Focused Libraries of Anti-Malarial Compounds. PLoS One. 2015;10(4):e0123598. Epub 2015/04/22. doi: 10.1371/journal.pone.0123598. PubMed PMID: 25894322; PubMed Central PMCID: PMCPmc4404333.

31. Lo MC, Aulabaugh A, Jin G, Cowling R, Bard J, Malamas M, et al. Evaluation of fluorescence-based thermal shift assays for hit identification in drug discovery. Anal Biochem. 2004;332(1):153-9. Epub 2004/08/11. doi: 10.1016/j.ab.2004.04.031. PubMed PMID: 15301960.

32. von Koschitzky I, Gerhardt H, Lammerhofer M, Kohout M, Gehringer M, Laufer S, et al. New insights into novel inhibitors against deoxyhypusine hydroxylase from plasmodium falciparum: compounds with an iron chelating potential. Amino Acids. 2015. Epub 2015/02/27. doi: 10.1007/s00726-015-1943-z. PubMed PMID: 25715757.

33. von Koschitzky I, Kaiser A. Chemical profiling of deoxyhypusine hydroxylase inhibitors for antimalarial therapy. Amino Acids. 2013;45(5):1047-53. Epub 2013/08/15. doi: 10.1007/s00726-013-1575-0. PubMed PMID: 23943044.

34. Kaiser A, Khomutov AR, Simonian A, Agostinelli E. A rapid and robust assay for the determination of the amino acid hypusine as a possible biomarker for a high-throughput screening of antimalarials and for the diagnosis and therapy of different diseases. Amino Acids. 2012;42(5):1651-9. Epub 2011/03/02. doi: 10.1007/s00726-011-0859-5. PubMed PMID: 21360085.

35. Njuguna JT, von Koschitzky I, Gerhardt H, Lammerhofer M, Choucry A, Pink M, et al. Target evaluation of deoxyhypusine synthase from Theileria parva the neglected animal parasite and its relationship to Plasmodium. Bioorg Med Chem. 2014. Epub 2014/06/10. doi: 10.1016/j.bmc.2014.05.007. PubMed PMID: 24909679.

36. Vu H, Pham NB, Quinn RJ. Direct screening of natural product extracts using mass spectrometry. J Biomol Screen. 2008;13(4):265-75. Epub 2008/03/20. doi: 10.1177/1087057108315739. PubMed PMID: 18349420.

37. Vu H, Roullier C, Campitelli M, Trenholme KR, Gardiner DL, Andrews KT, et al. Plasmodium gametocyte inhibition identified from a natural-product-based fragment library. ACS Chem Biol. 2013;8(12):2654-9. Epub 2013/10/02. doi: 10.1021/cb400582b. PubMed PMID: 24079418.

38. Hain AU, Bartee D, Sanders NG, Miller AS, Sullivan Jr DJ, Levitskaya J, et al. Identification of an Atg8-Atg3 protein-protein interaction inhibitor from the Medicines for Malaria Venture Malaria Box active in blood and liver stage Plasmodium falciparum parasites. J Med Chem. 2014. Epub 2014/05/03. doi: 10.1021/jm401675a. PubMed PMID: 24786226.

39. Boucher LE, Bosch J. Development of a multifunctional tool for drug screening against plasmodial protein-protein interactions via surface plasmon resonance. Journal of molecular recognition : JMR. 2013;26(10):496-500. Epub 2013/09/03. doi: 10.1002/jmr.2292. PubMed PMID: 23996492.

40. Bosch J, Turley S, Daly TM, Bogh SM, Villasmil ML, Roach C, et al. Structure of the MTIP-MyoA complex, a key component of the malaria parasite invasion motor. Proc Natl Acad Sci U S A. 2006;103(13):4852-7. doi: 10.1073/pnas.0510907103. PubMed PMID: 16547135; PubMed Central PMCID: PMCPMC1458759.

41. Li W, Mo W, Shen D, Sun L, Wang J, Lu S, et al. Yeast model uncovers dual roles of mitochondria in action of artemisinin. PLoS genetics. 2005;1(3):e36. doi: 10.1371/journal.pgen.0010036. PubMed PMID: 16170412; PubMed Central PMCID: PMC1201371.

42. Wang J, Huang L, Li J, Fan Q, Long Y, Li Y, et al. Artemisinin directly targets malarial mitochondria through its specific mitochondrial activation. PLoS One. 2010;5(3):e9582. doi: 10.1371/journal.pone.0009582. PubMed PMID: 20221395; PubMed Central PMCID: PMCPMC2833198.

43. Painter HJ, Morrisey JM, Mather MW, Vaidya AB. Specific role of mitochondrial electron transport in blood-stage Plasmodium falciparum. Nature. 2007;446(7131):88-91. doi: 10.1038/nature05572. PubMed PMID: 17330044.

44. Miyazaki M, Horii T, Hata K, Watanabe NA, Nakamoto K, Tanaka K, et al. In vitro activity of E1210, a novel antifungal, against clinically important yeasts and molds. Antimicrob Agents Chemother. 2011;55(10):4652-8. Epub 2011/08/10. doi: 10.1128/aac.00291-11. PubMed PMID: 21825291; PubMed Central PMCID: PMCPmc3186989.

45. Hata K, Horii T, Miyazaki M, Watanabe NA, Okubo M, Sonoda J, et al. Efficacy of oral E1210, a new broad-spectrum antifungal with a novel mechanism of action, in murine models of candidiasis, aspergillosis, and fusariosis. Antimicrob Agents Chemother. 2011;55(10):4543-51. Epub 2011/07/27. doi: 10.1128/aac.00366-11. PubMed PMID: 21788462; PubMed Central PMCID: PMCPmc3187015.

46. Tsukahara K, Hata K, Nakamoto K, Sagane K, Watanabe NA, Kuromitsu J, et al. Medicinal genetics approach towards identifying the molecular target of a novel inhibitor of fungal cell wall assembly. Mol Microbiol. 2003;48(4):1029-42. Epub 2003/05/20. PubMed PMID: 12753194.

47. Watanabe NA, Miyazaki M, Horii T, Sagane K, Tsukahara K, Hata K. E1210, a new broad-spectrum antifungal, suppresses Candida albicans hyphal growth through inhibition of glycosylphosphatidylinositol biosynthesis. Antimicrob Agents Chemother. 2012;56(2):960-71. Epub 2011/12/07. doi: 10.1128/aac.00731-11. PubMed PMID: 22143530; PubMed Central PMCID: PMCPmc3264227.

48. Yuan J, Johnson RL, Huang R, Wichterman J, Jiang H, Hayton K, et al. Genetic mapping of targets mediating differential chemical phenotypes in *Plasmodium falciparum*. Nat Chem Biol. 2009;5(10):765-71. doi: 10.1038/nchembio.215. PubMed PMID: 19734910; PubMed Central PMCID: PMCPMC2784992.

49. Ekland EH, Schneider J, Fidock DA. Identifying apicoplast-targeting antimalarials using high-throughput compatible approaches. Faseb j. 2011;25(10):3583-93. Epub 2011/07/13. doi: 10.1096/fj.11-187401. PubMed PMID: 21746861; PubMed Central PMCID: PMCPmc3177575.

50. Muhle RA, Adjalley S, Falkard B, Nkrumah LJ, Muhle ME, Fidock DA. A var gene promoter implicated in severe malaria nucleates silencing and is regulated by 3' untranslated region and intronic cis-elements. Int J Parasitol. 2009;39(13):1425-39. doi: 10.1016/j.ijpara.2009.05.001. PubMed PMID: 19463825; PubMed Central PMCID: PMCPMC2744600.

51. Frame I, Deniskin R, Rinderspacher A, Katz F, Deng S, Moir RD, et al. Yeast-based High-Throughput Screen Identifies Plasmodium falciparum Equilibrative Nucleoside Transporter 1 Inhibitors That Kill Malaria Parasites. ACS Chem Biol. 2015. Epub 2015/01/21. doi: 10.1021/cb500981y. PubMed PMID: 25602169.

52. Duffy S, Avery VM. Development and optimization of a novel 384-well anti-malarial imaging assay validated for high-throughput screening. Am J Trop Med Hyg. 2012;86(1):84-92. Epub 2012/01/11. doi: 10.4269/ajtmh.2012.11-0302. PubMed PMID: 22232455; PubMed Central PMCID: PMCPmc3247113.

53. Hasenkamp S, Sidaway A, Devine O, Roye R, Horrocks P. Evaluation of bioluminescence-based assays of anti-malarial drug activity. Malar J. 2013;12:58. doi: 10.1186/1475-2875-12-58. PubMed PMID: 23394077; PubMed Central PMCID: PMCPMC3571881.

54. Smilkstein M, Sriwilaijaroen N, Kelly JX, Wilairat P, Riscoe M. Simple and Inexpensive Fluorescence-Based Technique for High-Throughput Antimalarial Drug Screening. Antimicrob Agents Chemother 20004;48:1803–6 (2004). doi: 10.1128/AAC.48.5.1803-1806.2004. Epub 1993/06/01. PubMed PMID: 151051388333566. PubMed Central PMCID: PMC400546.

55. Lee S, Lim D, Lee E, Lee N, Lee HG, Cechetto J, et al. Discovery of carbohybrid-based 2-aminopyrimidine analogues as a new class of rapid-acting antimalarial agents using image-based cytological profiling assay. J Med Chem. 2014. Epub 2014/08/20. doi: 10.1021/jm5009693. PubMed PMID: 25137549.

56. Moon S, Lee S, Kim H, Freitas-Junior LH, Kang M, Ayong L, et al. An image analysis algorithm for malaria parasite stage classification and viability quantification. PLoS One. 2013;8(4):e61812. Epub 2013/04/30. doi: 10.1371/journal.pone.0061812. PubMed PMID: 23626733; PubMed Central PMCID: PMCPmc3634010.

57. Andrews KT, Fisher GM, Sumanadasa SD, Skinner-Adams T, Moeker J, Lopez M, et al. Antimalarial activity of compounds comprising a primary benzene sulfonamide fragment. Bioorg Med Chem Lett. 2013;23(22):6114-7. Epub 2013/10/03. doi: 10.1016/j.bmcl.2013.09.015. PubMed PMID: 24084158.

58. Walliker D, Quakyi IA, Wellems TE, McCutchan TF, Szarfman A, London WT, et al. Genetic analysis of the human malaria parasite Plasmodium falciparum. Science. 1987;236(4809):1661-6. Epub 1987/06/26. PubMed PMID: 3299700.

59. Huber W, Koella JC. A comparison of three methods of estimating EC50 in studies of drug resistance of malaria parasites. Acta Trop. 1993;55(4):257-61. Epub 1993/12/01. PubMed PMID: 8147282.

60. Wu W, Herrera Z, Ebert D, Baska K, Cho SH, DeRisi JL, et al. A chemical rescue screen identifies a Plasmodium falciparum apicoplast inhibitor targeting MEP isoprenoid precursor biosynthesis. Antimicrob Agents Chemother. 2015;59(1):356-64. Epub 2014/11/05. doi: 10.1128/aac.03342-14. PubMed PMID: 25367906; PubMed Central PMCID: PMCPmc4291372.

61. Bowman JD, Merino EF, Brooks CF, Striepen B, Carlier PR, Cassera MB. Antiapicoplast and gametocytocidal screening to identify the mechanisms of action of compounds within the malaria box. Antimicrob Agents Chemother. 2014;58(2):811-9. Epub 2013/11/20. doi: 10.1128/aac.01500-13. PubMed PMID: 24247137; PubMed Central PMCID: PMCPMC3910863.

62. Yao ZK, Krai PM, Merino EF, Simpson ME, Slebodnick C, Cassera MB, et al. Determination of the active stereoisomer of the MEP pathway-targeting antimalarial agent MMV008138, and initial structure-activity studies. Bioorg Med Chem Lett. 2015. Epub 2015/03/11. doi: 10.1016/j.bmcl.2015.02.020. PubMed PMID: 25754494.

63. Tanaka TQ, Williamson KC. A malaria gametocytocidal assay using oxidoreduction indicator, alamarBlue. Mol Biochem Parasitol. 2011;177(2):160-3. Epub 2011/02/15. doi: 10.1016/j.molbiopara.2011.02.005. PubMed PMID: 21316401; PubMed Central PMCID: PMCPmc3075389.

64. Swann J, Corey V, Scherer CA, Kato N, Comer E, Maetani M, et al. High-Throughput Luciferase-Based Assay for the Discovery of Therapeutics That Prevent Malaria. ACS Infectious Diseases. 2016;DOI: 10.1021/acsinfecdis.5b00143. doi: 10.1021/acsinfecdis.5b00143.

65. Janse CJ, Franke-Fayard B, Mair GR, Ramesar J, Thiel C, Engelmann S, et al. High efficiency transfection of Plasmodium berghei facilitates novel selection procedures. Mol Biochem Parasitol. 2006;145(1):60-70. Epub 2005/10/26. doi: 10.1016/j.molbiopara.2005.09.007. PubMed PMID: 16242190.

66. Yalaoui S, Zougbede S, Charrin S, Silvie O, Arduise C, Farhati K, et al. Hepatocyte permissiveness to Plasmodium infection is conveyed by a short and structurally conserved region of the CD81 large extracellular domain. PLoS Pathog. 2008;4(2):e1000010. Epub 2008/04/05. doi: 10.1371/journal.ppat.1000010. PubMed PMID: 18389082; PubMed Central PMCID: PMCPmc2279262.

67. Lucantoni L, Duffy S, Adjalley SH, Fidock DA, Avery VM. Identification of MMV malaria box inhibitors of *Plasmodium falciparum* early-stage gametocytes using a luciferase-based high-throughput assay. Antimicrob Agents Chemother. 2013;57(12):6050-62. doi: 10.1128/AAC.00870-13. PubMed PMID: 24060871; PubMed Central PMCID: PMC3837862.

68. Lucantoni L, Avery V. Whole-cell in vitro screening for gametocytocidal compounds. Future Med Chem. 2012;4(18):2337-60. doi: 10.4155/fmc.12.188. PubMed PMID: 23234555.

69. Lucantoni L, Fidock DA, Avery VM. A Luciferase-Based, High-Throughput Assay For Screening And Profiling Transmission-Blocking Compounds Against Plasmodium falciparum Gametocytes. Antimicrob Agents Chemother. 2016. Epub 2016/01/21. doi: 10.1128/aac.01949-15. PubMed PMID: 26787698.

70. Adjalley SH, Johnston GL, Li T, Eastman RT, Ekland EH, Eappen AG, et al. Quantitative assessment of Plasmodium falciparum sexual development reveals potent transmission-blocking activity by methylene blue. Proc Natl Acad Sci U S A. 2011;108(47):E1214-23. doi: 10.1073/pnas.1112037108. PubMed PMID: 22042867; PubMed Central PMCID: PMC3223476.

71. Duffy S, Avery VM. Identification of inhibitors of Plasmodium falciparum gametocyte development. Malar J. 2013;12(1):408. doi: 10.1186/1475-2875-12-408. PubMed PMID: 24206914; PubMed Central PMCID: PMC3842684.

72. Plouffe DM, Wree M, Du AY, Meister S, Li F, Patra K, et al. High-Throughput Assay and Discovery of Small Molecules that Interrupt Malaria Transmission. Cell host & microbe. 2016;19(1):114-26. Epub 2016/01/11. doi: 10.1016/j.chom.2015.12.001. PubMed PMID: 26749441.

73. Fivelman QL, McRobert L, Sharp S, Taylor CJ, Saeed M, Swales CA, et al. Improved synchronous production of Plasmodium falciparum gametocytes in vitro. Mol Biochem Parasitol. 2007;154(1):119-23. Epub 2007/05/25. doi: 10.1016/j.molbiopara.2007.04.008. PubMed PMID: 17521751.

74. Cevenini L, Camarda G, Michelini E, Siciliano G, Calabretta MM, Bona R, et al. Multicolor bioluminescence boosts malaria research: quantitative dual-color assay and single-cell imaging in Plasmodium falciparum parasites. Analytical chemistry. 2014;86(17):8814-21. Epub 2014/08/08. doi: 10.1021/ac502098w. PubMed PMID: 25102353; PubMed Central PMCID: PMCPmc4151787.

75. D'Alessandro S, Silvestrini F, Dechering K, Corbett Y, Parapini S, Timmerman M, et al. A Plasmodium falciparum screening assay for anti-gametocyte drugs based on parasite lactate dehydrogenase detection. The Journal of antimicrobial chemotherapy. 2013;68(9):2048-58. Epub 2013/05/07. doi: 10.1093/jac/dkt165. PubMed PMID: 23645588.

76. D'Alessandro S, Corbett Y, Ilboudo DP, Misiano P, Dahiya N, Abay SM, et al. Salinomycin and Other Ionophores as a New Class of Antimalarial Drugs with Transmission-Blocking Activity. Antimicrob Agents Chemother. 2015;59(9):5135-44. Epub 2015/06/10. doi: 10.1128/aac.04332-14. PubMed PMID: 26055362.

77. Carter R, Ranford-Cartwright L, Alano P. The culture and preparation of gametocytes of Plasmodium falciparum for immunochemical, molecular, and mosquito infectivity studies. Methods Mol Biol. 1993;21:67-88. Epub 1993/01/01. doi: 10.1385/0-89603-239-6:67. PubMed PMID: 8220736.

78. Reader J, Botha M, Theron A, Lauterbach SB, Rossouw C, Engelbrecht D, et al. Nowhere to hide: interrogating different metabolic parameters of Plasmodium falciparum gametocytes in a transmission blocking drug discovery pipeline towards malaria elimination. Malar J. 2015;14(1):213. Epub 2015/05/23. doi: 10.1186/s12936-015-0718-z. PubMed PMID: 25994518.

79. Tanaka TQ, Dehdashti SJ, Nguyen DT, McKew JC, Zheng W, Williamson KC. A quantitative high throughput assay for identifying gametocytocidal compounds. Mol Biochem Parasitol. 2013;188(1):20-5. doi: 10.1016/j.molbiopara.2013.02.005. PubMed PMID: 23454872; PubMed Central PMCID: PMC3640759.

80. Sanders NG, Sullivan DJ, Mlambo G, Dimopoulos G, Tripathi AK. Gametocytocidal Screen Identifies Novel Chemical Classes with Plasmodium falciparum Transmission Blocking Activity. PLoS One. 2014;9(8):e105817. Epub 2014/08/27. doi: 10.1371/journal.pone.0105817. PubMed PMID: 25157792; PubMed Central PMCID: PMCPMC4144897.

81. Lucantoni L, Silvestrini F, Signore M, Siciliano G, Eldering M, Dechering KJ, et al. A simple and predictive phenotypic High Content Imaging assay for Plasmodium falciparum mature gametocytes to identify malaria transmission blocking compounds. Scientific reports. 2015;5:16414. Epub 2015/11/11. doi: 10.1038/srep16414. PubMed PMID: 26553647.

82. Ruecker A, Mathias DK, Straschil U, Churcher TS, Dinglasan RR, Leroy D, et al. A male and female gametocyte functional viability assay to identify biologically relevant malaria transmission-blocking drugs. Antimicrob Agents Chemother. 2014;58(12):7292-302. doi: 10.1128/AAC.03666-14. PubMed PMID: 25267664; PubMed Central PMCID: PMCPMC4249523.

83. Read M, Hyde JE. Simple in vitro cultivation of the malaria parasite Plasmodium falciparum (erythrocytic stages) suitable for large-scale preparations. Methods Mol Biol. 1993;21:43-55. Epub 1993/01/01. doi: 10.1385/0-89603-239-6:43. PubMed PMID: 8220733.

84. Ifediba T, Vanderberg JP. Complete in vitro maturation of Plasmodium falciparum gametocytes. Nature. 1981;294(5839):364-6. Epub 1981/11/26. PubMed PMID: 7031476.

85. Bessoff K, Spangenberg T, Foderaro JE, Jumani RS, Ward GE, Huston CD. Identification of Cryptosporidium parvum Active Chemical Series by Repurposing the Open Access Malaria Box. Antimicrob Agents Chemother. 2014;58(5):2731-9. Epub 2014/02/26. doi: 10.1128/aac.02641-13. PubMed PMID: 24566188.

86. Bessoff K, Sateriale A, Lee KK, Huston CD. Drug repurposing screen reveals FDA-approved inhibitors of human HMG-CoA reductase and isoprenoid synthesis that block Cryptosporidium parvum growth. Antimicrob Agents Chemother. 2013;57(4):1804-14. doi: 10.1128/AAC.02460-12. PubMed PMID: 23380723; PubMed Central PMCID: PMC3623326.

87. Gut J, Nelson RG. Cryptosporidium parvum: synchronized excystation in vitro and evaluation of sporozoite infectivity with a new lectin-based assay. The Journal of eukaryotic microbiology. 1999;46(5):56s-7s. Epub 1999/10/16. PubMed PMID: 10519247.

88. Kaiser M, Maes L, Tadoori LP, Spangenberg T, Ioset JR. Repurposing of the Open Access Malaria Box for Kinetoplastid Diseases Identifies Novel Active Scaffolds against Trypanosomatids. J Biomol Screen. 2015;20(5):634-45. doi: 10.1177/1087057115569155. PubMed PMID: 25690568.

89. Cos P, Vlietinck AJ, Berghe DV, Maes L. Anti-infective potential of natural products: how to develop a stronger in vitro 'proof-of-concept'. J Ethnopharmacol. 2006;106(3):290-302. Epub 2006/05/16. doi: 10.1016/j.jep.2006.04.003. PubMed PMID: 16698208.

90. Mothana RA, Al-Musayeib NM, Al-Ajmi MF, Cos P, Maes L. Evaluation of the in vitro antiplasmodial, antileishmanial, and antitrypanosomal activity of medicinal plants used in saudi and yemeni traditional medicine. Evidence-based complementary and alternative medicine : eCAM. 2014;2014:905639. Epub 2014/06/26. doi: 10.1155/2014/905639. PubMed PMID: 24963330; PubMed Central PMCID: PMCPmc4055400.

91. Boyom FF, Fokou PV, Tchokouaha LR, Spangenberg T, Mfopa AN, Kouipou RM, et al. Repurposing the open access malaria box to discover potent inhibitors of Toxoplasma gondii and Entamoeba histolytica. Antimicrob Agents Chemother. 2014;58(10):5848-54. doi: 10.1128/AAC.02541-14. PubMed PMID: 25049259; PubMed Central PMCID: PMCPMC4187973.

92. Jin C, Kaewintajuk K, Jiang J, Jeong W, Kamata M, Kim HS, et al. Toxoplasma gondii: a simple high-throughput assay for drug screening in vitro. Exp Parasitol. 2009;121(2):132-6. doi: 10.1016/j.exppara.2008.10.006. PubMed PMID: 18977350.

93. Upcroft JA, Upcroft P. Drug susceptibility testing of anaerobic protozoa. Antimicrob Agents Chemother. 2001;45(6):1810-4. doi: 10.1128/AAC.45.6.1810-1814.2001. PubMed PMID: 11353630; PubMed Central PMCID: PMCPMC90550.

94. Rizk MA, El-Sayed SA, Terkawi MA, Youssef MA, El Said el Sel S, Elsayed G, et al. Optimization of a Fluorescence-Based Assay for Large-Scale Drug Screening against Babesia and Theileria Parasites. PLoS One. 2015;10(4):e0125276. doi: 10.1371/journal.pone.0125276. PubMed PMID: 25915529; PubMed Central PMCID: PMCPMC4411034.

95. Ingram-Sieber K, Cowan N, Panic G, Vargas M, Mansour NR, Bickle QD, et al. Orally active antischistosomal early leads identified from the open access malaria box. PLoS Negl Trop Dis. 2014;8(1):e2610. Epub 2014/01/15. doi: 10.1371/journal.pntd.0002610. PubMed PMID: 24416463; PubMed Central PMCID: PMCPMC3886923.

96. Aboulaila M, Munkhjargal T, Sivakumar T, Ueno A, Nakano Y, Yokoyama M, et al. Apicoplast-targeting antibacterials inhibit the growth of Babesia parasites. Antimicrob Agents Chemother. 2012;56(6):3196-206. doi: 10.1128/AAC.05488-11. PubMed PMID: 22391527; PubMed Central PMCID: PMCPMC3370714.

97. AbouLaila M, Batadoj D, Salama A, Munkhjargal T, Ichikawa-Seki M, M AT, et al. Evaluation of the inhibitory effects of miltefosine on the growth of Babesia and Theileria parasites. Veterinary parasitology. 2014;204(3-4):104-10. doi: 10.1016/j.vetpar.2014.05.023. PubMed PMID: 24938827.

98. Salama AA, Aboulaila M, Moussa AA, Nayel MA, El-Sify A, Terkawi MA, et al. Evaluation of in vitro and in vivo inhibitory effects of fusidic acid on Babesia and Theileria parasites. Veterinary parasitology. 2013;191(1-2):1-10. Epub 2012/09/19. doi: 10.1016/j.vetpar.2012.08.022. PubMed PMID: 22985928.

99. Salama AA, AbouLaila M, Terkawi MA, Mousa A, El-Sify A, Allaam M, et al. Inhibitory effect of allicin on the growth of Babesia and Theileria equi parasites. Parasitol Res. 2014;113(1):275-83. doi: 10.1007/s00436-013-3654-2. PubMed PMID: 24173810.

100. Alemán Resto Y, Fernández Robledo JA. Identification of MMV Malaria Box inhibitors of Perkinsus marinus using an ATP-based bioluminescence assay. PLoS One. 2014;9(10):e111051. doi: 10.1371/journal.pone.0111051. PubMed PMID: 25337810; PubMed Central PMCID: PMCPMC4206467.

101. Shridhar S, Hassan K, Sullivan DJ, Vasta GR, Fernandez Robledo JA. Quantitative assessment of the proliferation of the protozoan parasite Perkinsus marinus using a bioluminescence assay for ATP content. International journal for parasitology -- drugs and drug resistance. 2013;3:85-92. Epub 2014/02/18. doi: 10.1016/j.ijpddr.2013.03.001. PubMed PMID: 24533297; PubMed Central PMCID: PMCPmc3862420.

102. Rice CA, Colon BL, Alp M, Goker H, Boykin DW, Kyle DE. Bis-benzimidazole hits against Naegleria fowleri discovered with new high-throughput screens. Antimicrob Agents Chemother. 2015;59(4):2037-44. Epub 2015/01/22. doi: 10.1128/aac.05122-14. PubMed PMID: 25605363; PubMed Central PMCID: PMCPmc4356813.

103. Muller J, Aguado-Martinez A, Manser V, Balmer V, Winzer P, Ritler D, et al. Buparvaquone is active against Neospora caninum in vitro and in experimentally infected mice. International journal for parasitology -- drugs and drug resistance. 2015;5(1):16-25. Epub 2015/05/06. doi: 10.1016/j.ijpddr.2015.02.001. PubMed PMID: 25941626; PubMed Central PMCID: PMCPmc4412913.

104. Barna F, Debache K, Vock CA, Kuster T, Hemphill A. In vitro effects of novel ruthenium complexes in Neospora caninum and Toxoplasma gondii tachyzoites. Antimicrob Agents Chemother. 2013;57(11):5747-54. doi: 10.1128/AAC.02446-12. PubMed PMID: 23979747; PubMed Central PMCID: PMCPMC3811262.

105. Ojo KK, Reid MC, Kallur Siddaramaiah L, Muller J, Winzer P, Zhang Z, et al. *Neospora caninum* calcium-dependent protein kinase 1 is an effective drug target for neosporosis therapy. PLoS One. 2014;9(3):e92929. doi: 10.1371/journal.pone.0092929. PubMed PMID: 24681759; PubMed Central PMCID: PMCPMC3969379.

106. Moore RB, Obornik M, Janouskovec J, Chrudimsky T, Vancova M, Green DH, et al. A photosynthetic alveolate closely related to apicomplexan parasites. Nature. 2008;451(7181):959-63. doi: 10.1038/nature06635. PubMed PMID: 18288187.

107. Sutak R, Slapeta J, San Roman M, Camadro JM, Lesuisse E. Nonreductive iron uptake mechanism in the marine alveolate Chromera velia. Plant Physiol. 2010;154(2):991-1000. doi: 10.1104/pp.110.159947. PubMed PMID: 20724644; PubMed Central PMCID: PMCPMC2949016.

108. Weatherby K, Murray S, Carter D, Slapeta J. Surface and flagella morphology of the motile form of Chromera velia revealed by field-emission scanning electron microscopy. Protist. 2011;162(1):142-53. doi: 10.1016/j.protis.2010.02.003. PubMed PMID: 20643581.

109. Ramirez B, Bickle Q, Yousif F, Fakorede F, Mouries MA, Nwaka S. Schistosomes: challenges in compound screening. Expert opinion on drug discovery. 2007;2(s1):S53-61. doi: 10.1517/17460441.2.S1.S53. PubMed PMID: 23489033.

110. Mansour NR, Bickle QD. Comparison of microscopy and Alamar blue reduction in a larval based assay for schistosome drug screening. PLoS Negl Trop Dis. 2010;4(8):e795. doi: 10.1371/journal.pntd.0000795. PubMed PMID: 20706580; PubMed Central PMCID: PMCPMC2919390.

111. Tritten L, Silbereisen A, Keiser J. In vitro and in vivo efficacy of Monepantel (AAD 1566) against laboratory models of human intestinal nematode infections. PLoS Negl Trop Dis. 2011;5(12):e1457. doi: 10.1371/journal.pntd.0001457. PubMed PMID: 22216366; PubMed Central PMCID: PMCPMC3246443.

112. Wimmersberger D, Tritten L, Keiser J. Development of an in vitro drug sensitivity assay for Trichuris muris first-stage larvae. Parasit Vectors. 2013;6:42. doi: 10.1186/1756-3305-6-42. PubMed PMID: 23433224; PubMed Central PMCID: PMCPMC3606346.

113. Abdulla MH, Lim KC, Sajid M, McKerrow JH, Caffrey CR. Schistosomiasis mansoni: novel chemotherapy using a cysteine protease inhibitor. PLoS Med. 2007;4(1):e14. Epub 2007/01/12. doi: 10.1371/journal.pmed.0040014. PubMed PMID: 17214506; PubMed Central PMCID: PMCPmc1764436.

114. Abdulla MH, Ruelas DS, Wolff B, Snedecor J, Lim KC, Xu F, et al. Drug discovery for schistosomiasis: hit and lead compounds identified in a library of known drugs by medium-throughput phenotypic screening. PLoS Negl Trop Dis. 2009;3(7):e478. doi: 10.1371/journal.pntd.0000478. PubMed PMID: 19597541; PubMed Central PMCID: PMCPMC2702839.

115. Colley DG, Wikel SK. Schistosoma mansoni: simplified method for the production of schistosomules. Exp Parasitol. 1974;35(1):44-51. PubMed PMID: 4815018.

116. Stefanic S, Dvorak J, Horn M, Braschi S, Sojka D, Ruelas DS, et al. RNA interference in Schistosoma mansoni schistosomula: selectivity, sensitivity and operation for larger-scale screening. PLoS Negl Trop Dis. 2010;4(10):e850. doi: 10.1371/journal.pntd.0000850. PubMed PMID: 20976050; PubMed Central PMCID: PMCPMC2957409.

117. Duvall RH, DeWitt WB. An improved perfusion technique for recovering adult schistosomes from laboratory animals. Am J Trop Med Hyg. 1967;16(4):483-6. PubMed PMID: 4952149.

118. Rojo-Arreola L, Long T, Asarnow D, Suzuki BM, Singh R, Caffrey CR. Chemical and genetic validation of the statin drug target to treat the helminth disease, schistosomiasis. PLoS One. 2014;9(1):e87594. doi: 10.1371/journal.pone.0087594. PubMed PMID: 24489942; PubMed Central PMCID: PMCPMC3906178.

119. Fonseca NC, da Cruz LF, da Silva Villela F, do Nascimento Pereira GA, de Siqueira-Neto JL, Kellar D, et al. Synthesis of a sugar-based thiosemicarbazone series and structure-activity relationship versus the parasite cysteine proteases rhodesain, cruzain, and Schistosoma mansoni cathepsin B1. Antimicrob Agents Chemother. 2015;59(5):2666-77. doi: 10.1128/AAC.04601-14. PubMed PMID: 25712353; PubMed Central PMCID: PMCPMC4394791.

120. Glaser J, Schurigt U, Suzuki BM, Caffrey CR, Holzgrabe U. Anti-Schistosomal Activity of Cinnamic Acid Esters: Eugenyl and Thymyl Cinnamate Induce Cytoplasmic Vacuoles and Death in Schistosomula of Schistosoma mansoni. Molecules. 2015;20(6):10873-83. doi: 10.3390/molecules200610873. PubMed PMID: 26076109.

121. Andrews P, Thomas H, Pohlke R, Seubert J. Praziquantel. Medicinal research reviews. 1983;3(2):147-200. PubMed PMID: 6408323.

122. Marcellino C, Gut J, Lim KC, Singh R, McKerrow J, Sakanari J. WormAssay: a novel computer application for whole-plate motion-based screening of macroscopic parasites. PLoS Negl Trop Dis. 2012;6(1):e1494. doi: 10.1371/journal.pntd.0001494. PubMed PMID: 22303493; PubMed Central PMCID: PMCPMC3269415.

123. Townson S, Ramirez B, Fakorede F, Mouries MA, Nwaka S. Challenges in drug discovery for novel antifilarials. Expert opinion on drug discovery. 2007;2(s1):S63-73. doi: 10.1517/17460441.2.S1.S63. PubMed PMID: 23489034.

124. Suzuki Y, St Onge RP, Mani R, King OD, Heilbut A, Labunskyy VM, et al. Knocking out multigene redundancies via cycles of sexual assortment and fluorescence selection. Nat Methods. 2011;8(2):159-64. Epub 2011/01/11. doi: 10.1038/nmeth.1550. PubMed PMID: 21217751; PubMed Central PMCID: PMCPmc3076670.

125. Schlecht U, Miranda M, Suresh S, Davis RW, St Onge RP. Multiplex assay for condition-dependent changes in protein-protein interactions. Proc Natl Acad Sci U S A. 2012;109(23):9213-8. Epub 2012/05/23. doi: 10.1073/pnas.1204952109. PubMed PMID: 22615397; PubMed Central PMCID: PMCPmc3384208.

126. Lardenois A, Liu Y, Walther T, Chalmel F, Evrard B, Granovskaia M, et al. Execution of the meiotic noncoding RNA expression program and the onset of gametogenesis in yeast require the conserved exosome subunit Rrp6. Proc Natl Acad Sci U S A. 2011;108(3):1058-63. doi: 10.1073/pnas.1016459108. PubMed PMID: 21149693; PubMed Central PMCID: PMCPMC3024698.

127. St Onge R, Schlecht U, Scharfe C, Evangelista M. Forward chemical genetics in yeast for discovery of chemical probes targeting metabolism. Molecules. 2012;17(11):13098-115. doi: 10.3390/molecules171113098. PubMed PMID: 23128089; PubMed Central PMCID: PMCPMC3539408.

128. Bobenchik AM, Augagneur Y, Hao B, Hoch JC, Ben Mamoun C. Phosphoethanolamine methyltransferases in phosphocholine biosynthesis: functions and potential for antiparasite therapy. FEMS Microbiol Rev. 2011;35(4):609-19. doi: 10.1111/j.1574-6976.2011.00267.x. PubMed PMID: 21303393; PubMed Central PMCID: PMCPMC4107886.

129. Bobenchik AM, Witola WH, Augagneur Y, Nic Lochlainn L, Garg A, Pachikara N, et al. Plasmodium falciparum phosphoethanolamine methyltransferase is essential for malaria transmission. Proc Natl Acad Sci U S A. 2013;110(45):18262-7. Epub 2013/10/23. doi: 10.1073/pnas.1313965110. PubMed PMID: 24145416; PubMed Central PMCID: PMCPmc3831454.

130. Pessi G, Kociubinski G, Mamoun CB. A pathway for phosphatidylcholine biosynthesis in Plasmodium falciparum involving phosphoethanolamine methylation. Proc Natl Acad Sci U S A. 2004;101(16):6206-11. doi: 10.1073/pnas.0307742101. PubMed PMID: 15073329; PubMed Central PMCID: PMCPMC395947.

131. Witola WH, El Bissati K, Pessi G, Xie C, Roepe PD, Mamoun CB. Disruption of the Plasmodium falciparum PfPMT gene results in a complete loss of phosphatidylcholine biosynthesis via the serine-decarboxylase-phosphoethanolamine-methyltransferase pathway and severe growth and survival defects. J Biol Chem. 2008;283(41):27636-43. doi: 10.1074/jbc.M804360200. PubMed PMID: 18694927; PubMed Central PMCID: PMCPMC2562060.

132. Bobenchik AM, Choi JY, Mishra A, Rujan IN, Hao B, Voelker DR, et al. Identification of inhibitors of Plasmodium falciparum phosphoethanolamine methyltransferase using an enzyme-coupled transmethylation assay. BMC biochemistry. 2010;11:4. doi: 10.1186/1471-2091-11-4. PubMed PMID: 20085640; PubMed Central PMCID: PMC2824672.

133. Garg A, Lukk T, Kumar V, Choi JY, Augagneur Y, Voelker DR, et al. Structure, function and inhibition of the phosphoethanolamine methyltransferases of the human malaria parasites Plasmodium vivax and Plasmodium knowlesi. Scientific reports. 2015;5:9064. doi: 10.1038/srep09064. PubMed PMID: 25761669; PubMed Central PMCID: PMCPMC4357015.

134. Pessi G, Choi JY, Reynolds JM, Voelker DR, Mamoun CB. In vivo evidence for the specificity of Plasmodium falciparum phosphoethanolamine methyltransferase and its coupling to the Kennedy pathway. J Biol Chem. 2005;280(13):12461-6. doi: 10.1074/jbc.M414626200. PubMed PMID: 15664981.

135. Witola WH, Pessi G, El Bissati K, Reynolds JM, Mamoun CB. Localization of the phosphoethanolamine methyltransferase of the human malaria parasite Plasmodium falciparum to the Golgi apparatus. J Biol Chem. 2006;281(30):21305-11. doi: 10.1074/jbc.M603260200. PubMed PMID: 16704982.

136. Winzeler EA, Shoemaker DD, Astromoff A, Liang H, Anderson K, Andre B, et al. Functional characterization of the S. cerevisiae genome by gene deletion and parallel analysis. Science. 1999;285(5429):901-6. PubMed PMID: 10436161.

137. Khozoie C, Pleass RJ, Avery SV. The antimalarial drug quinine disrupts Tat2p-mediated tryptophan transport and causes tryptophan starvation. J Biol Chem. 2009;284(27):17968-74. doi: 10.1074/jbc.M109.005843. PubMed PMID: 19416971; PubMed Central PMCID: PMCPMC2709357.

138. Islahudin F, Tindall SM, Mellor IR, Swift K, Christensen HE, Fone KC, et al. The antimalarial drug quinine interferes with serotonin biosynthesis and action. Scientific reports. 2014;4:3618. doi: 10.1038/srep03618. PubMed PMID: 24402577; PubMed Central PMCID: PMCPMC3885885.

139. Dittmar JC, Reid RJ, Rothstein R. ScreenMill: a freely available software suite for growth measurement, analysis and visualization of high-throughput screen data. BMC bioinformatics. 2010;11:353. doi: 10.1186/1471-2105-11-353. PubMed PMID: 20584323; PubMed Central PMCID: PMCPMC2909220.

140. Vallieres C, Fisher N, Lemoine M, Pamlard O, Beaupierre S, Guillou C, et al. A rapid in vivo colorimetric library screen for inhibitors of microbial respiration. ACS Chem Biol. 2012;7(10):1659-65. doi: 10.1021/cb3002717. PubMed PMID: 22762126.

141. Islahudin F, Khozoie C, Bates S, Ting KN, Pleass RJ, Avery SV. Cell wall perturbation sensitizes fungi to the antimalarial drug chloroquine. Antimicrob Agents Chemother. 2013;57(8):3889-96. doi: 10.1128/AAC.00478-13. PubMed PMID: 23733464; PubMed Central PMCID: PMCPMC3719739.

142. Serbus LR, Landmann F, Bray WM, White PM, Ruybal J, Lokey RS, et al. A cell-based screen reveals that the albendazole metabolite, albendazole sulfone, targets Wolbachia. PLoS Pathog. 2012;8(9):e1002922. Epub 2012/10/03. doi: 10.1371/journal.ppat.1002922. PubMed PMID: 23028321; PubMed Central PMCID: PMCPmc3447747.

143. Gayen P, Nayak A, Saini P, Mukherjee N, Maitra S, Sarkar P, et al. A double-blind controlled field trial of doxycycline and albendazole in combination for the treatment of bancroftian filariasis in India. Acta Trop. 2013;125(2):150-6. doi: 10.1016/j.actatropica.2012.10.011. PubMed PMID: 23123345.

144. Sharma R, Hoti SL, Vasuki V, Sankari T, Meena RL, Das PK. Filamentation temperature-sensitive protein Z (FtsZ) of Wolbachia, endosymbiont of Wuchereria bancrofti: a potential target for anti-filarial chemotherapy. Acta Trop. 2013;125(3):330-8. doi: 10.1016/j.actatropica.2012.12.004. PubMed PMID: 23262214.

145. Warrier T, Martinez-Hoyos M, Marin-Amieva M, Colmenarejo G, Porras E, Alvarez-Pedraglio A, et al. Identification of Novel Anti-mycobacterial Compounds by Screening a Pharmaceutical Small-Molecule Library against Nonreplicating *Mycobacterium tuberculosis*. ACS Infectious Diseases. 2015;DOI: 10.1021/acsinfecdis.5b00025.

146. Gold B, Warrier T, Nathan C. A multi-stress model for high throughput screening against non-replicating Mycobacterium tuberculosis. Methods Mol Biol. 2015;1285:293-315. doi: 10.1007/978-1-4939-2450-9_18. PubMed PMID: 25779324.

147. Zheng P, Somersan-Karakaya S, Lu S, Roberts J, Pingle M, Warrier T, et al. Synthetic calanolides with bactericidal activity against replicating and nonreplicating Mycobacterium tuberculosis. J Med Chem. 2014;57(9):3755-72. doi: 10.1021/jm4019228. PubMed PMID: 24694175.

148. Bryk R, Gold B, Venugopal A, Singh J, Samy R, Pupek K, et al. Selective killing of nonreplicating mycobacteria. Cell host & microbe. 2008;3(3):137-45. doi: 10.1016/j.chom.2008.02.003. PubMed PMID: 18329613; PubMed Central PMCID: PMCPMC2423947.

149. Gold B, Pingle M, Brickner SJ, Shah N, Roberts J, Rundell M, et al. Nonsteroidal anti-inflammatory drug sensitizes Mycobacterium tuberculosis to endogenous and exogenous antimicrobials. Proc Natl Acad Sci U S A. 2012;109(40):16004-11. doi: 10.1073/pnas.1214188109. PubMed PMID: 23012453; PubMed Central PMCID: PMCPMC3479555.

150. Kicka S, Trofimov V, Harrison C, Ouertatani-Sakouhi H, McKinney J, Scapozza L, et al. Establishment and validation of whole-cell based fluorescence assays to identify anti-mycobacterial compounds using the Acanthamoeba castellanii-Mycobacterium marinum host-pathogen system. PLoS One. 2014;9(1):e87834. doi: 10.1371/journal.pone.0087834. PubMed PMID: 24498207; PubMed Central PMCID: PMCPMC3909256.

151. Gordon CJ, Muesing MA, Proudfoot AE, Power CA, Moore JP, Trkola A. Enhancement of human immunodeficiency virus type 1 infection by the CC-chemokine RANTES is independent of the mechanism of virus-cell fusion. J Virol. 1999;73(1):684-94. PubMed PMID: 9847374; PubMed Central PMCID: PMCPMC103875.

152. Carmichael J, DeGraff WG, Gazdar AF, Minna JD, Mitchell JB. Evaluation of a tetrazolium-based semiautomated colorimetric assay: assessment of chemosensitivity testing. Cancer Res. 1987;47(4):936-42. PubMed PMID: 3802100.

153. Trkola A, Paxton WA, Monard SP, Hoxie JA, Siani MA, Thompson DA, et al. Genetic subtype-independent inhibition of human immunodeficiency virus type 1 replication by CC and CXC chemokines. J Virol. 1998;72(1):396-404. PubMed PMID: 9420238; PubMed Central PMCID: PMCPMC109387.

154. Shen J, Li S, Bowsher RR, Vick A. Using DTA and DTAARRAY variables and programming in WinNonlin ASCII models to streamline user-defined calculation and data analysis. The AAPS journal. 2015;17(2):474-8. doi: 10.1208/s12248-014-9711-7. PubMed PMID: 25583216; PubMed Central PMCID: PMC4365079.

155. Heatherington AC, Vicini P, Golde H. A pharmacokinetic/pharmacodynamic comparison of SAAM II and PC/WinNonlin modeling software. Journal of pharmaceutical sciences. 1998;87(10):1255-63. doi: 10.1021/js9603562. PubMed PMID: 9758686.

156. Fehintola FA, Akinyinka OO, Adewole IF, Maponga CC, Ma Q, Morse GD. Drug interactions in the treatment and chemoprophylaxis of malaria in HIV infected individuals in sub Saharan Africa. Curr Drug Metab. 2011;12(1):51-6. PubMed PMID: 21222586; PubMed Central PMCID: PMCPMC3233991.

157. Khetani SR, Bhatia SN. Microscale culture of human liver cells for drug development. Nat Biotechnol. 2008;26(1):120-6. Epub 2007/11/21. doi: 10.1038/nbt1361. PubMed PMID: 18026090.

158. Chan TS, Yu H, Moore A, Khetani SR, Tweedie D. Meeting the challenge of predicting hepatic clearance of compounds slowly metabolized by cytochrome P450 using a novel hepatocyte model, HepatoPac. Drug metabolism and disposition: the biological fate of chemicals. 2013;41(12):2024-32. doi: 10.1124/dmd.113.053397. PubMed PMID: 23959596.

159. Khetani SR, Kanchagar C, Ukairo O, Krzyzewski S, Moore A, Shi J, et al. Use of micropatterned cocultures to detect compounds that cause drug-induced liver injury in humans. Toxicological sciences : an official journal of the Society of Toxicology. 2013;132(1):107-17. Epub 2012/11/16. doi: 10.1093/toxsci/kfs326. PubMed PMID: 23152190.

160. Wang WW, Khetani SR, Krzyzewski S, Duignan DB, Obach RS. Assessment of a micropatterned hepatocyte coculture system to generate major human excretory and circulating drug metabolites. Drug metabolism and disposition: the biological fate of chemicals. 2010;38(10):1900-5. doi: 10.1124/dmd.110.034876. PubMed PMID: 20595376.

161. March S, Ng S, Velmurugan S, Galstian A, Shan J, Logan DJ, et al. A microscale human liver platform that supports the hepatic stages of Plasmodium falciparum and vivax. Cell host & microbe. 2013;14(1):104-15. doi: 10.1016/j.chom.2013.06.005. PubMed PMID: 23870318; PubMed Central PMCID: PMC3780791.

162. Rheinwald JG, Green H. Formation of a keratinizing epithelium in culture by a cloned cell line derived from a teratoma. Cell. 1975;6(3):317-30. PubMed PMID: 1052770.

163. Chen AA, Thomas DK, Ong LL, Schwartz RE, Golub TR, Bhatia SN. Humanized mice with ectopic artificial liver tissues. Proc Natl Acad Sci U S A. 2011;108(29):11842-7. doi: 10.1073/pnas.1101791108. PubMed PMID: 21746904; PubMed Central PMCID: PMCPMC3142004.

164. Whitebread S, Hamon J, Bojanic D, Urban L. Keynote review: in vitro safety pharmacology profiling: an essential tool for successful drug development. Drug Discov Today. 2005;10(21):1421-33. doi: 10.1016/S1359-6446(05)03632-9. PubMed PMID: 16243262.

165. Fitzgerald LW, Burn TC, Brown BS, Patterson JP, Corjay MH, Valentine PA, et al. Possible role of valvular serotonin 5-HT(2B) receptors in the cardiopathy associated with fenfluramine. Mol Pharmacol. 2000;57(1):75-81. PubMed PMID: 10617681.

166. Roth BL. Drugs and valvular heart disease. N Engl J Med. 2007;356(1):6-9. doi: 10.1056/NEJMp068265. PubMed PMID: 17202450.

167. Hughes TB, Miller GP, Swamidass SJ. Site of reactivity models predict molecular reactivity of diverse chemicals with glutathione. Chem Res Toxicol. 2015;28(4):797-809. doi: 10.1021/acs.chemrestox.5b00017. PubMed PMID: 25742281.

168. Hughes TB, Miller GP, Swamidass SJ. Modeling Epoxidation of Drug-like Molecules with a Deep Machine Learning Network. ACS Central Science. 2015;1(4):168–80.

169. Shoemaker RH. The NCI60 human tumour cell line anticancer drug screen. Nat Rev Cancer. 2006;6(10):813-23. doi: 10.1038/nrc1951. PubMed PMID: 16990858.

170. Monks A, Scudiero D, Skehan P, Shoemaker R, Paull K, Vistica D, et al. Feasibility of a high-flux anticancer drug screen using a diverse panel of cultured human tumor cell lines. J Natl Cancer Inst. 1991;83(11):757-66. Epub 1991/06/05. PubMed PMID: 2041050.

171. Olszewski KL, Llinás M. Extraction of hydrophilic metabolites from *Plasmodium falciparum*-infected erythrocytes for metabolomic analysis. Methods Mol Biol. 2013;923:259-66. doi: 10.1007/978-1-62703-026-7_17. PubMed PMID: 22990783; PubMed Central PMCID: PMCPMC3529256.

172. Kim CC, Wilson EB, DeRisi JL. Improved methods for magnetic purification of malaria parasites and haemozoin. Malar J. 2010;9:17. doi: 10.1186/1475-2875-9-17. PubMed PMID: 20074366; PubMed Central PMCID: PMCPMC2817699.

173. Lu W, Clasquin MF, Melamud E, Amador-Noguez D, Caudy AA, Rabinowitz JD. Metabolomic analysis via reversed-phase ion-pairing liquid chromatography coupled to a stand alone orbitrap mass spectrometer. Analytical chemistry. 2010;82(8):3212-21. doi: 10.1021/ac902837x. PubMed PMID: 20349993; PubMed Central PMCID: PMCPMC2863137.

174. Melamud E, Vastag L, Rabinowitz JD. Metabolomic analysis and visualization engine for LC-MS data. Analytical chemistry. 2010;82(23):9818-26. doi: 10.1021/ac1021166. PubMed PMID: 21049934.

175. Clasquin MF, Melamud E, Rabinowitz JD. LC-MS data processing with MAVEN: a metabolomic analysis and visualization engine. Current protocols in bioinformatics / editoral board, Andreas D Baxevanis [et al]. 2012;Chapter 14:Unit14 1. doi: 10.1002/0471250953.bi1411s37. PubMed PMID: 22389014; PubMed Central PMCID: PMCPMC4055029.

176. R Core Team. R: A language and environment for statistical computing. R Foundation for Statistical Computing, <http://wwwR-projectorg/>. 2015.

177. Papadatos G, Overington JP. The ChEMBL database: a taster for medicinal chemists. Future Med Chem. 2014;6(4):361-4. Epub 2014/03/19. doi: 10.4155/fmc.14.8. PubMed PMID: 24635517.

178. Gaulton A, Bellis LJ, Bento AP, Chambers J, Davies M, Hersey A, et al. ChEMBL: a large-scale bioactivity database for drug discovery. Nucleic Acids Res. 2012;40(Database issue):D1100-7. doi: 10.1093/nar/gkr777. PubMed PMID: 21948594; PubMed Central PMCID: PMCPMC3245175.

179. Bento AP, Gaulton A, Hersey A, Bellis LJ, Chambers J, Davies M, et al. The ChEMBL bioactivity database: an update. Nucleic Acids Res. 2014;42(Database issue):D1083-90. Epub 2013/11/12. doi: 10.1093/nar/gkt1031. PubMed PMID: 24214965; PubMed Central PMCID: PMCPmc3965067.

180. Spangenberg T, Burrows JN, Kowalczyk P, McDonald S, Wells TN, Willis P. The open access malaria box: a drug discovery catalyst for neglected diseases. PLoS One. 2013;8(6):e62906. doi: 10.1371/journal.pone.0062906. PubMed PMID: 23798988; PubMed Central PMCID: PMC3684613.
