# Supplementary material for: Open Source Drug Discovery with the Malaria Box Compound Collection for Neglected Diseases and Beyond
Source: PLoS Pathog. 2016 Jul 28;12(7):e1005763. doi: 10.1371/journal.ppat.1005763 (PMC4965013; doi:10.1371/journal.ppat.1005763)

**MMV Number**	**NSC Number**	**MGLI50**	**Notes**
007384	766470	-7.34	Colon, differential, potent
019074	766523	-4.91	Specific lines sensitive
665803	766546	-5.01	
665796	766556	-5.00	
020275	766585	-4.93	
000760	766634	-6.02	Differential
666020	766672	-4.90	
665969	766692	-6.29	
666597	766722	-5.57	Differential
006962	766755	-5.61	Differential, CNS

MGLI50 = Mean Growth Log I50

National Cancer Institute Developmental Therapeutics Program Dose Response Curves	NSC: 766470 / 2	SSPL: 0YQS	EXP. ID: 1310NS81
	Report Date: July 13, 2015	Test Date: October 28, 2013	

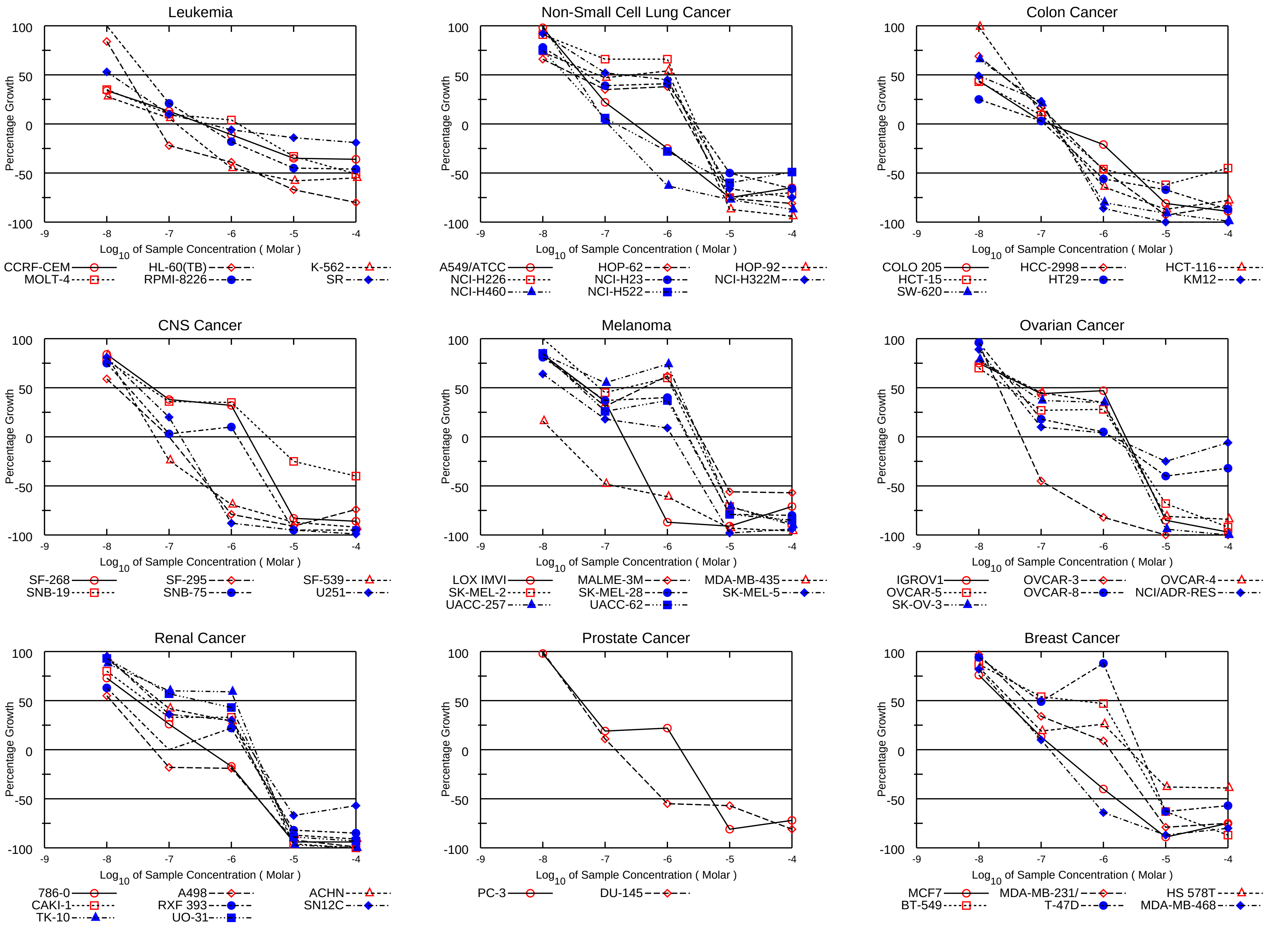

National Cancer Institute Developmental Therapeutics Program Mean Graphs	NSC :766470/2	Units :Molar	SSPL :0YQS	EXP. ID :1310NS81
	Report Date :July 13, 2015		Test Date :October 28, 2013	

Panel/Cell Line	Log_{10}GI50	Log_{10}TGI	Log_{10}LC50
**Leukemia**			
CCRF-CEM	< -8.00	-6.46	> -4.00
HL-60(TB)	-7.68	-7.21	-5.61
K-562	< -8.00	-6.88	-5.63
MOLT-4	< -8.00	-5.89	-4.06
RPMI-8226	-7.37	-6.46	> -4.00
SR	-7.92	-6.37	> -4.00
**Non-Small Cell Lung Cancer**			
A549/ATCC	-7.37	-6.53	-5.50
HOP-62	-7.49	-5.67	-5.23
HOP-92		-5.62	-5.26
NCI-H226	-5.89	-5.53	-5.18
NCI-H23	-7.29	-5.55	-5.00
NCI-H322M	-6.74	-5.59	-5.15
NCI-H460	-7.48	-6.95	-6.20
NCI-H522	-7.64	-6.82	
**Colon Cancer**			
COLO 205	< -8.00	-6.87	-5.52
HCC-2998	-7.63	-6.73	-5.94
HCT-116	-7.44	-6.85	-6.19
HCT-15	< -8.00	-6.84	
HT29	< -8.00	-6.95	-6.10
KM12	< -8.00	-6.79	-6.33
SW-620	-7.64	-6.79	-6.30
**CNS Cancer**			
SF-268	-7.27	-5.72	-5.29
SF-295	-7.84	-6.99	-6.37
SF-539	-7.69	-7.22	-6.42
SNB-19	-7.33	-5.41	> -4.00
SNB-75	-7.65	-5.90	-5.43
U251	-7.49	-6.81	-6.35
**Melanoma**			
LOX IMVI	-7.28	-6.70	-6.30
MALME-3M		-5.48	-5.05
MDA-MB-435	< -8.00	-7.75	-6.83
SK-MEL-2		-5.54	-5.16
SK-MEL-28	-7.29	-5.66	-5.25
SK-MEL-5	-7.70	-5.91	-5.45
UACC-257	-5.84	-5.49	-5.15
UACC-62	-7.40	-5.68	-5.25
**Ovarian Cancer**			
IGROV1	-7.19	-5.65	-5.27
OVCAR-3	-7.68	-7.32	-6.87
OVCAR-4	-7.16	-5.70	-5.27
OVCAR-5	-7.53	-5.71	-5.19
OVCAR-8	-7.41	-5.89	> -4.00
NCI/ADR-RES	-7.51	-5.86	> -4.00
SK-OV-3	-7.31	-5.73	-5.34
**Renal Cancer**			
786-0	-7.51	-6.40	-5.58
A498	-7.93	-7.24	-5.58
ACHN	-7.16	-5.75	-5.32
CAKI-1	-7.36	-5.74	-5.36
RXF 393	-7.80		-5.31
SN12C	-7.23	-5.69	-5.17
TK-10	-5.94	-5.62	-5.30
UO-31	-6.50	-5.67	-5.29
**Prostate Cancer**			
PC-3	-7.39	-5.79	-5.30
DU-145	-7.43	-6.83	-6.08
**Breast Cancer**			
MCF7	-7.59	-6.75	-5.79
MDA-MB-231/ATCC	-7.26	-5.90	-5.33
HS 578T	-7.48	-5.60	> -4.00
BT-549	-6.44	-5.57	-5.12
T-47D		-5.42	-5.09
MDA-MB-468	-7.56	-6.87	-6.20
_MID	-7.42	-6.22	-5.38
Delta	0.58	1.53	1.49
Range	2.16	2.34	2.87

GI50

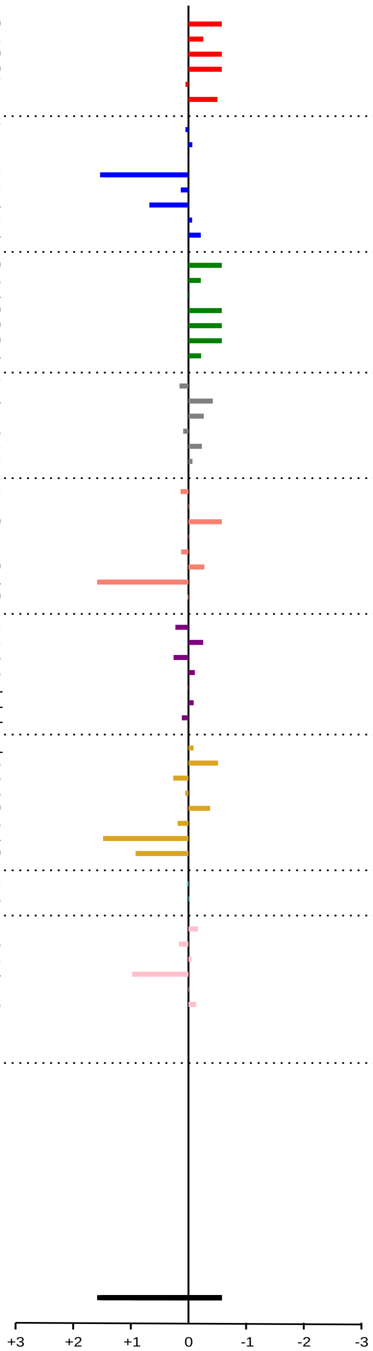

TGI

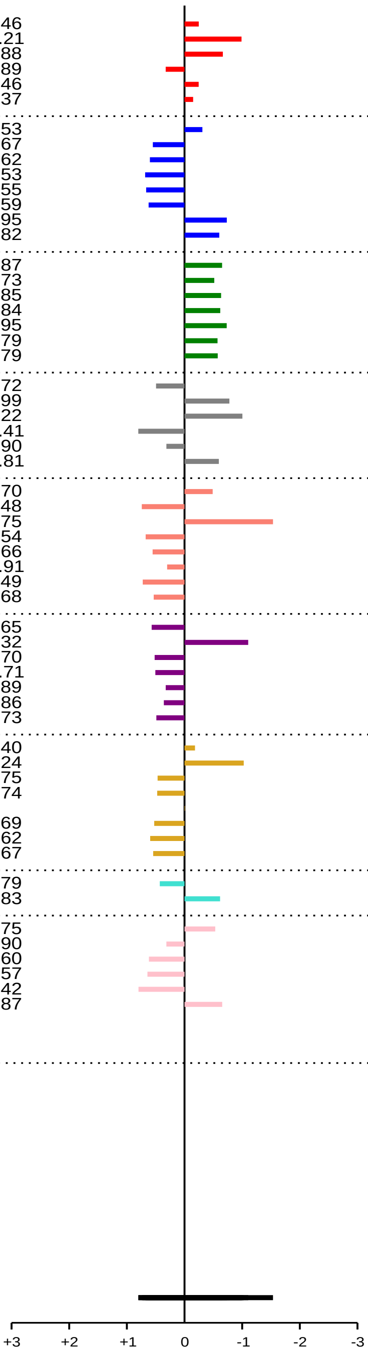

LC50

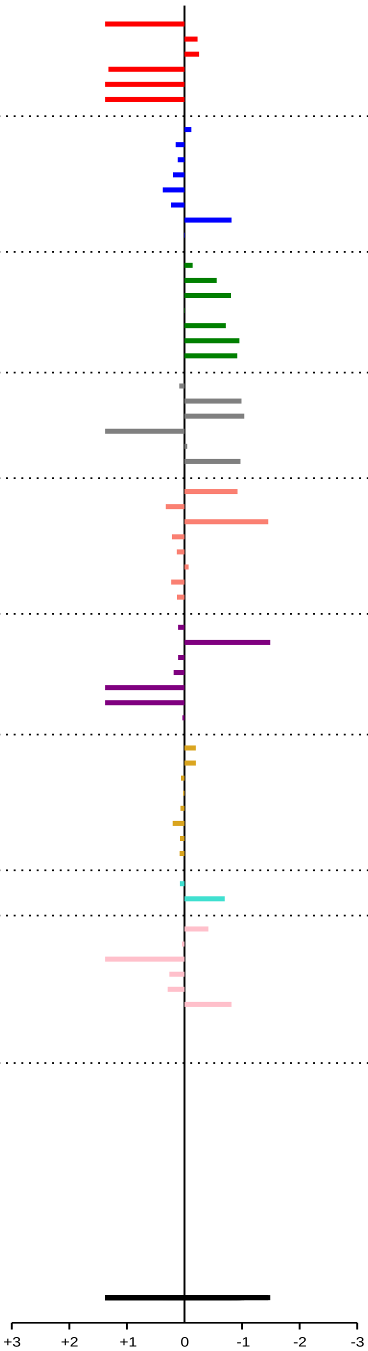

National Cancer Institute Developmental Therapeutics Program<br>Dose Response Curves	NSC:766470/2	SSPL:0YQS	EXP. ID:1310NS81
	Report Date:July 13, 2015	Test Date:October 28, 2013	

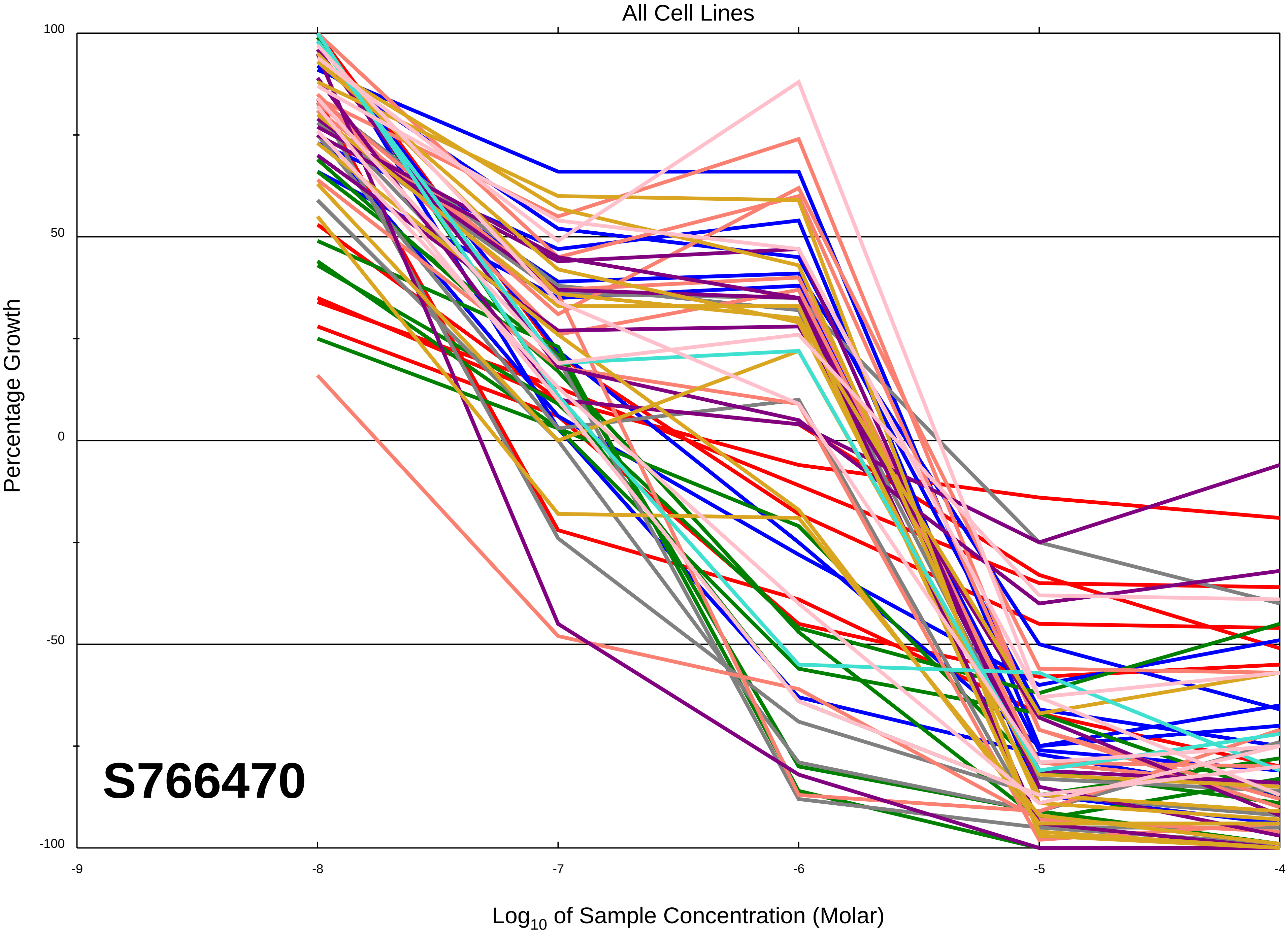

National Cancer Institute Developmental Therapeutics Program
Dose Response Curves

NSC: 766470 / 2 | SSPL: 0YQS | EXP. ID: 1402RS24

Report Date: July 13, 2015 | Test Date: February 03, 2014

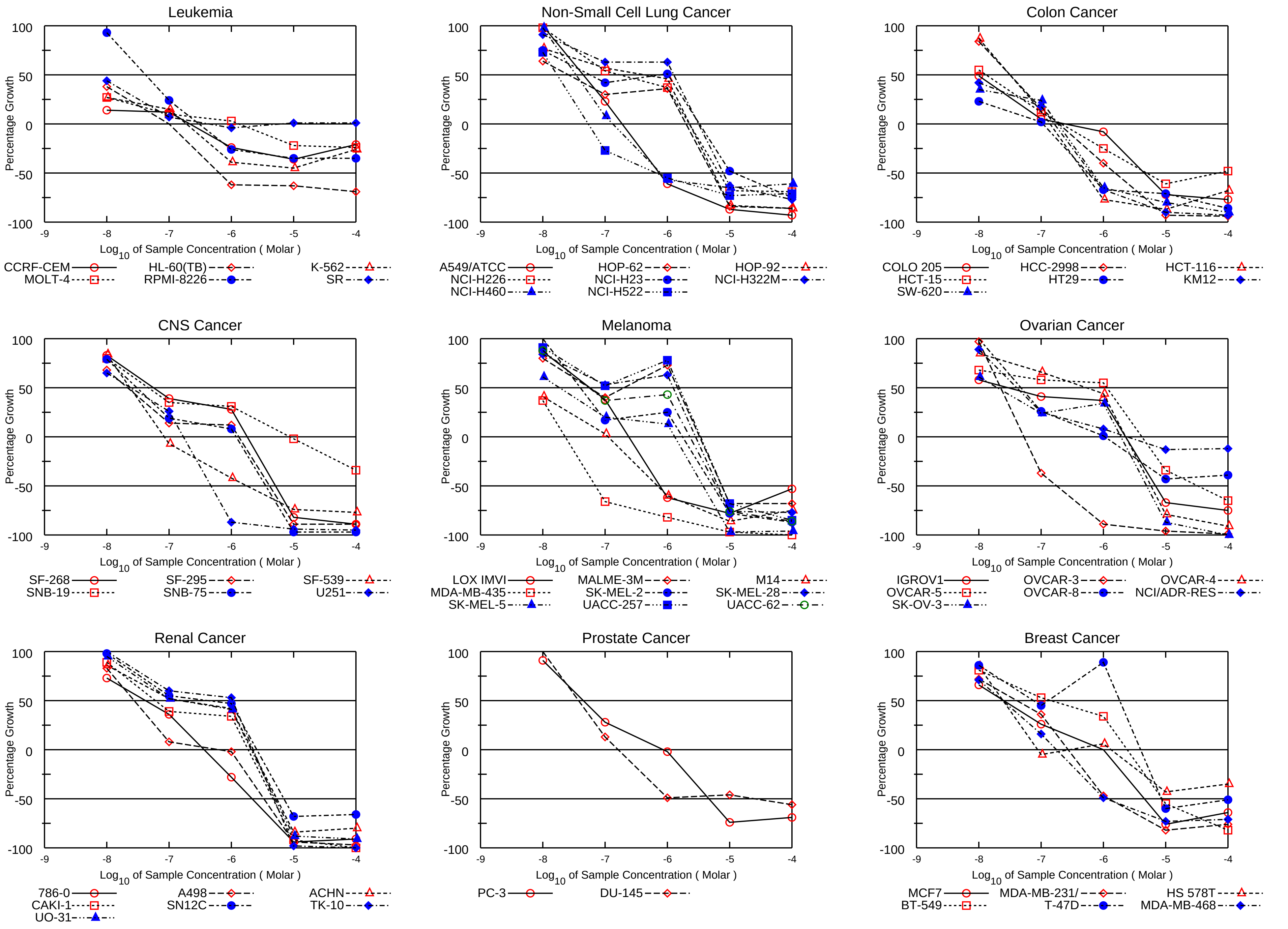

National Cancer Institute Developmental Therapeutics Program Mean Graphs	NSC :766470/2	Units :Molar	SSPL :0YQS	EXP. ID :1402RS24
	Report Date :July 13, 2015		Test Date :February 03, 2014	

Panel/Cell Line	Log_{10}GI50	Log_{10}TGI	Log_{10}LC50
**Leukemia**			
CCRF-CEM	< -8.00	-6.66	> -4.00
HL-60(TB)	< -8.00	-7.00	-6.19
K-562	< -8.00	-6.72	> -4.00
MOLT-4	< -8.00	-5.89	> -4.00
RPMI-8226	-7.37	-6.51	> -4.00
SR	< -8.00		> -4.00
**Non-Small Cell Lung Cancer**			
A549/ATCC	-7.35	-6.72	-6.13
HOP-62	-7.58	-5.70	-5.28
HOP-92	-6.40	-5.65	-5.26
NCI-H226	-6.76	-5.65	-5.17
NCI-H23		-5.49	-4.93
NCI-H322M	-5.89	-5.50	-5.10
NCI-H460	-7.47	-6.88	-6.11
NCI-H522	-7.77	-7.27	-6.18
**Colon Cancer**			
COLO 205	< -8.00	-6.60	-5.35
HCC-2998	-7.50	-6.72	-5.80
HCT-116	-7.50	-6.85	-6.30
HCT-15	-7.88	-6.67	
HT29	< -8.00	-6.97	-6.25
KM12	< -8.00	-6.79	-6.20
SW-620	< -8.00	-6.73	-6.17
**CNS Cancer**			
SF-268	-7.24	-5.74	-5.29
SF-295	-7.66	-5.88	-5.38
SF-539	-7.63	-7.08	-5.75
SNB-19	-7.33	-5.06	> -4.00
SNB-75	-7.52	-5.92	-5.44
U251	-7.62	-6.77	-6.33
**Melanoma**			
LOX IMVI	-7.25	-6.62	-6.12
MALME-3M		-5.48	-5.13
M14	< -8.00	-6.95	-6.16
MDA-MB-435	< -8.00	-7.64	-7.15
SK-MEL-2	-7.39	-5.76	-5.27
SK-MEL-28	-5.90	-5.54	-5.18
SK-MEL-5	-7.73	-5.88	-5.42
UACC-257	-5.81	-5.47	-5.13
UACC-62	-7.25	-5.64	-5.22
**Ovarian Cancer**			
IGROV1	-7.51	-5.64	-5.16
OVCAR-3	-7.65	-7.28	-6.75
OVCAR-4	-6.28	-5.64	-5.24
OVCAR-5	-5.95	-5.38	-4.48
OVCAR-8	-7.31	-5.98	> -4.00
NCI/ADR-RES	-7.39	-5.61	> -4.00
SK-OV-3	-7.70	-5.72	-5.31
**Renal Cancer**			
786-0	-7.38	-6.44	-5.67
A498	-7.56	-6.18	-5.48
ACHN	-6.83	-5.66	-5.27
CAKI-1	-7.22	-5.73	-5.33
SN12C	-6.33	-5.59	-5.16
TK-10	-5.98	-5.65	-5.32
UO-31	-6.82	-5.69	-5.30
**Prostate Cancer**			
PC-3	-7.34	-6.06	-5.33
DU-145	-7.40	-6.79	-4.64
**Breast Cancer**			
MCF7	-7.60	-6.00	-5.34
MDA-MB-231/ATCC	-7.39	-6.56	-5.91
HS 578T	-7.62		> -4.00
BT-549	-6.83	-5.62	-5.06
T-47D		-5.40	-5.07
MDA-MB-468	-7.61	-6.75	-5.94
_MID	-7.34	-6.17	-5.32
Delta	0.66	1.47	1.83
Range	2.19	2.58	3.15

GI50

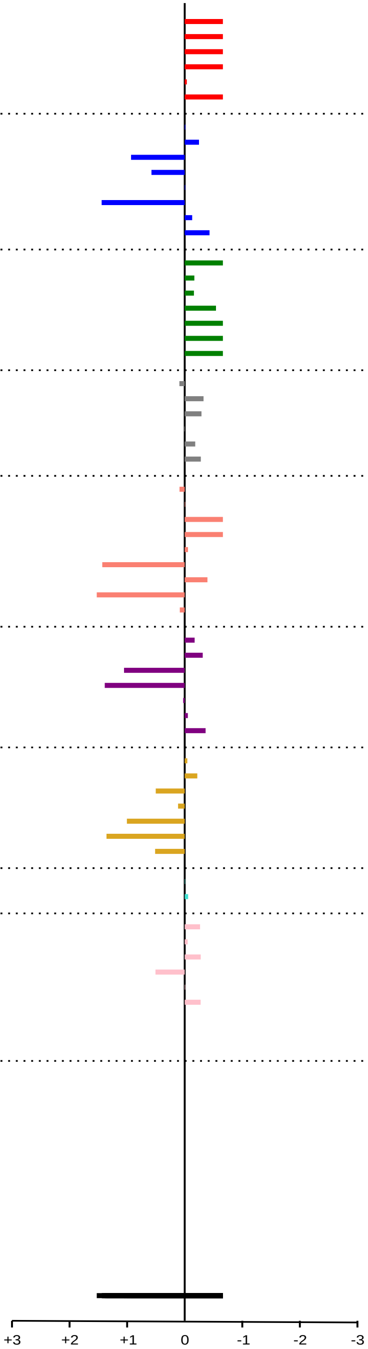

TGI

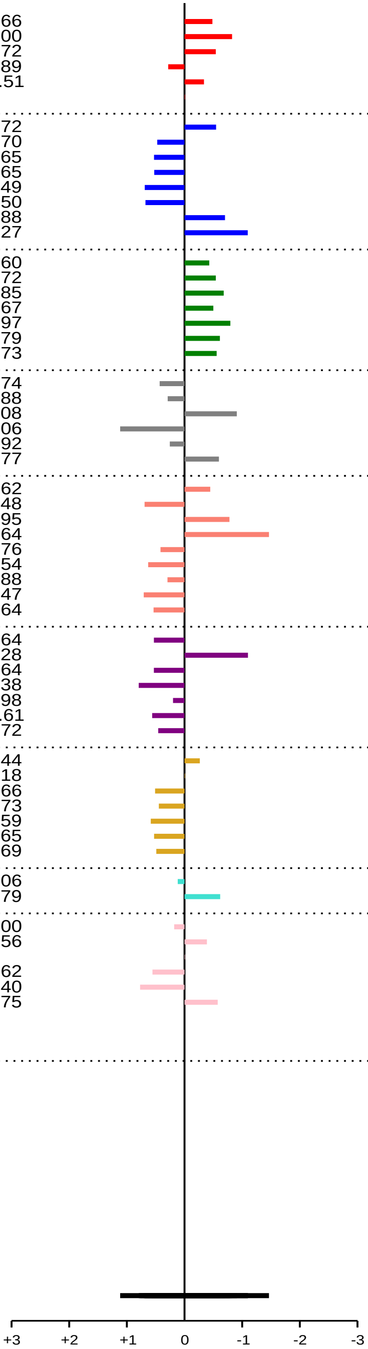

LC50

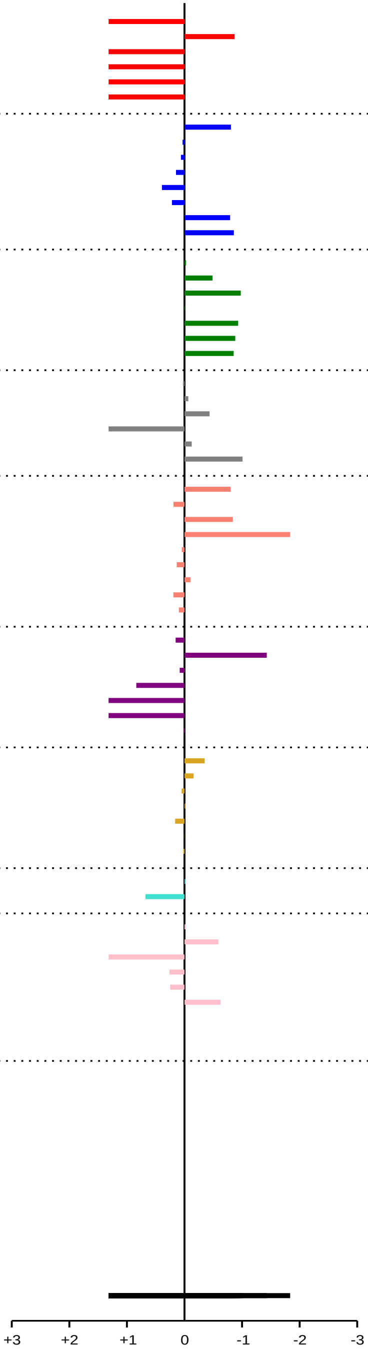

National Cancer Institute Developmental Therapeutics Program<br>Dose Response Curves	NSC:766470/2	SSPL:0YQS	EXP. ID:1402RS24
	Report Date:July 13, 2015	Test Date:February 03, 2014	

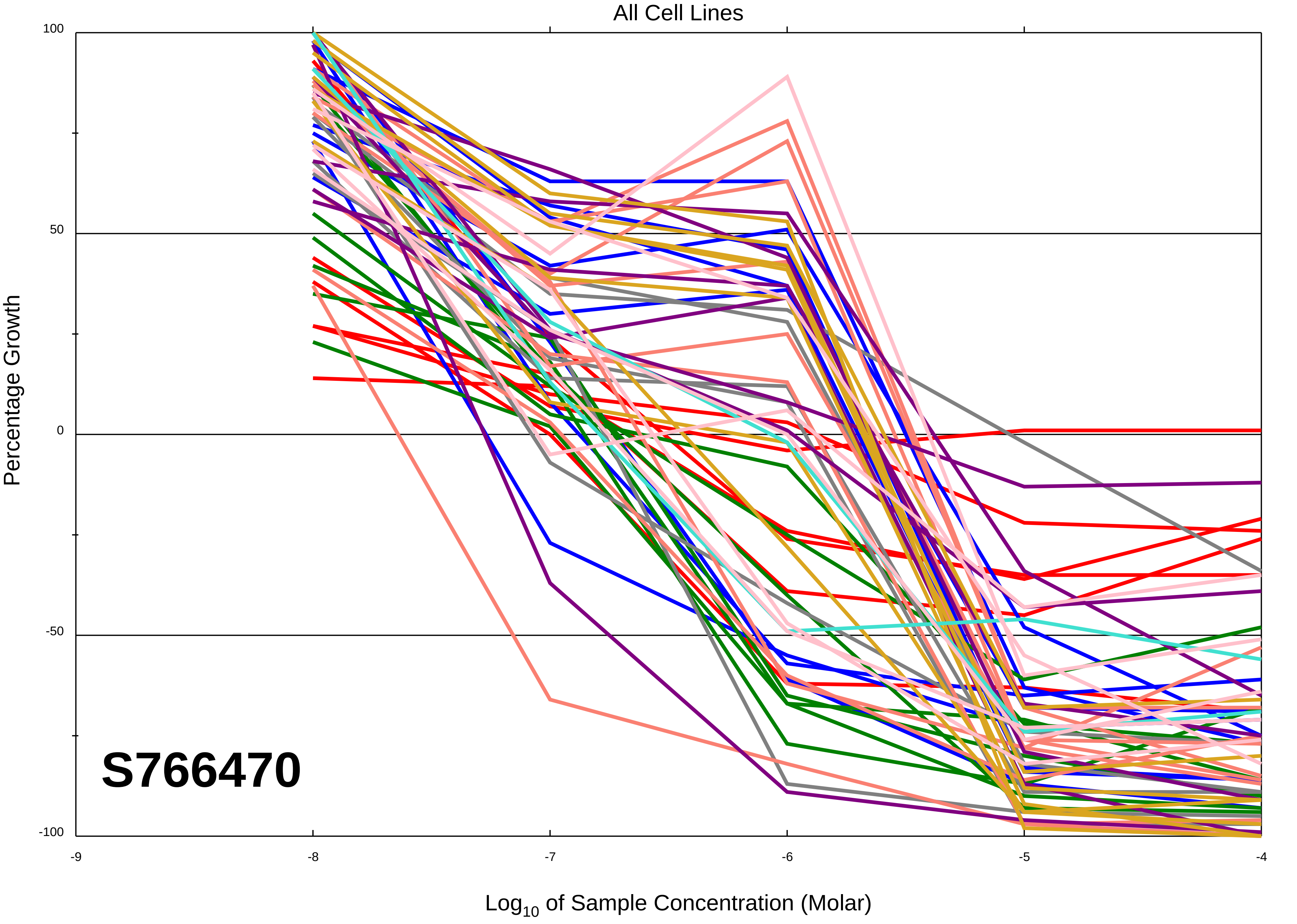

National Cancer Institute Developmental Therapeutics Program Dose Response Curves	NSC: 766523 / 2	SSPL: 0YQS	EXP. ID: 1310NS81
	Report Date: July 13, 2015	Test Date: October 28, 2013	

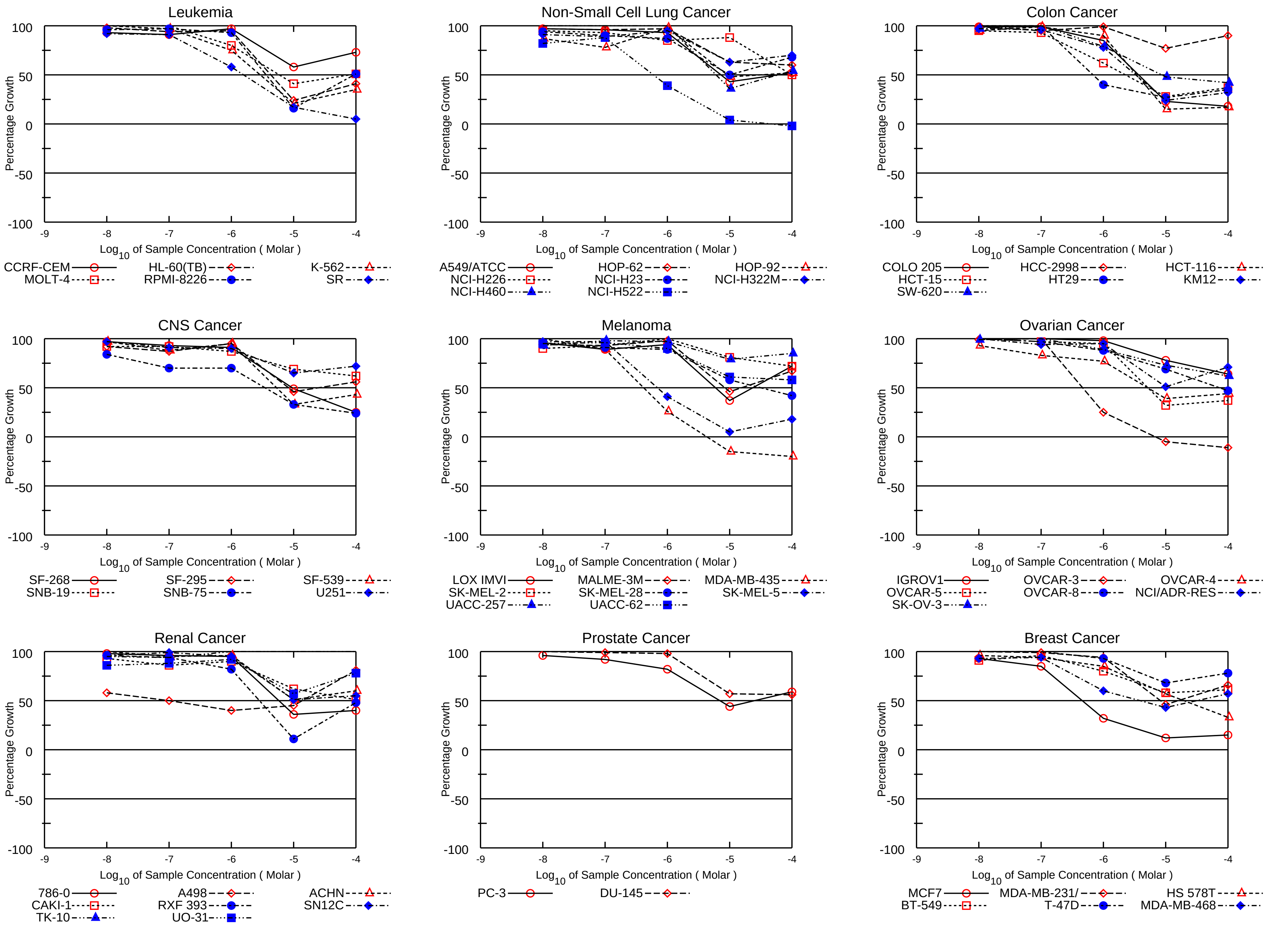

National Cancer Institute Developmental Therapeutics Program
Mean Graphs

NSC :766523/2 | Units :Molar | SSPL :0YQS | EXP. ID :1310NS81

Report Date :July 13, 2015 | Test Date :October 28, 2013

Panel/Cell Line	Log_{10}GI50	Log_{10}TGI	Log_{10}LC50
**Leukemia**			
CCRF-CEM	> -4.00	> -4.00	> -4.00
HL-60(TB)	-5.36	> -4.00	> -4.00
K-562	-5.53	> -4.00	> -4.00
MOLT-4		> -4.00	> -4.00
RPMI-8226		> -4.00	> -4.00
SR	-5.79	> -4.00	> -4.00
**Non-Small Cell Lung Cancer**			
A549/ATCC		> -4.00	> -4.00
HOP-62	> -4.00	> -4.00	> -4.00
HOP-92		> -4.00	> -4.00
NCI-H226	> -4.00	> -4.00	> -4.00
NCI-H23		> -4.00	> -4.00
NCI-H322M	> -4.00	> -4.00	> -4.00
NCI-H460		> -4.00	> -4.00
NCI-H522	-6.23	-4.30	> -4.00
**Colon Cancer**			
COLO 205	-5.44	> -4.00	> -4.00
HCC-2998	> -4.00	> -4.00	> -4.00
HCT-116	-5.47	> -4.00	> -4.00
HCT-15	-5.64	> -4.00	> -4.00
HT29	-6.16	> -4.00	> -4.00
KM12	-5.49	> -4.00	> -4.00
SW-620	-5.06	> -4.00	> -4.00
**CNS Cancer**			
SF-268	-5.02	> -4.00	> -4.00
SF-295		> -4.00	> -4.00
SF-539	-5.27	> -4.00	> -4.00
SNB-19	> -4.00	> -4.00	> -4.00
SNB-75	-5.45	> -4.00	> -4.00
U251	> -4.00	> -4.00	> -4.00
**Melanoma**			
LOX IMVI		> -4.00	> -4.00
MALME-3M		> -4.00	> -4.00
MDA-MB-435	-6.38	-5.36	> -4.00
SK-MEL-2	> -4.00	> -4.00	> -4.00
SK-MEL-28	-4.50	> -4.00	> -4.00
SK-MEL-5	-6.17	> -4.00	> -4.00
UACC-257	> -4.00	> -4.00	> -4.00
UACC-62	> -4.00	> -4.00	> -4.00
**Ovarian Cancer**			
IGROV1	> -4.00	> -4.00	> -4.00
OVCAR-3	-6.32	-5.16	> -4.00
OVCAR-4	-5.28	> -4.00	> -4.00
OVCAR-5	-5.29	> -4.00	> -4.00
OVCAR-8	-4.15	> -4.00	> -4.00
NCI/ADR-RES	> -4.00	> -4.00	> -4.00
SK-OV-3	> -4.00	> -4.00	> -4.00
**Renal Cancer**			
786-0	-5.24	> -4.00	> -4.00
A498		> -4.00	> -4.00
ACHN	> -4.00	> -4.00	> -4.00
CAKI-1	> -4.00	> -4.00	> -4.00
RXF 393	-5.55	> -4.00	> -4.00
SN12C	> -4.00	> -4.00	> -4.00
TK-10	> -4.00	> -4.00	> -4.00
UO-31	> -4.00	> -4.00	> -4.00
**Prostate Cancer**			
PC-3		> -4.00	> -4.00
DU-145	> -4.00	> -4.00	> -4.00
**Breast Cancer**			
MCF7	-6.34	> -4.00	> -4.00
MDA-MB-231/ATCC		> -4.00	> -4.00
HS 578T	-4.70	> -4.00	> -4.00
BT-549	> -4.00	> -4.00	> -4.00
T-47D	> -4.00	> -4.00	> -4.00
MDA-MB-468		> -4.00	> -4.00
_MID	-4.8	-4.05	-4.0
Delta	1.58	1.31	0
Range	2.38	1.36	0.0

GI50

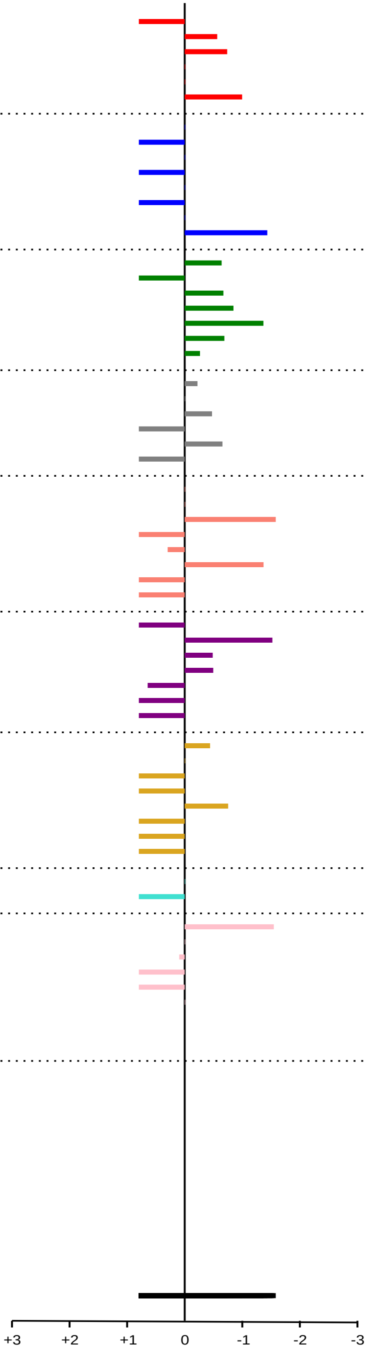

TGI

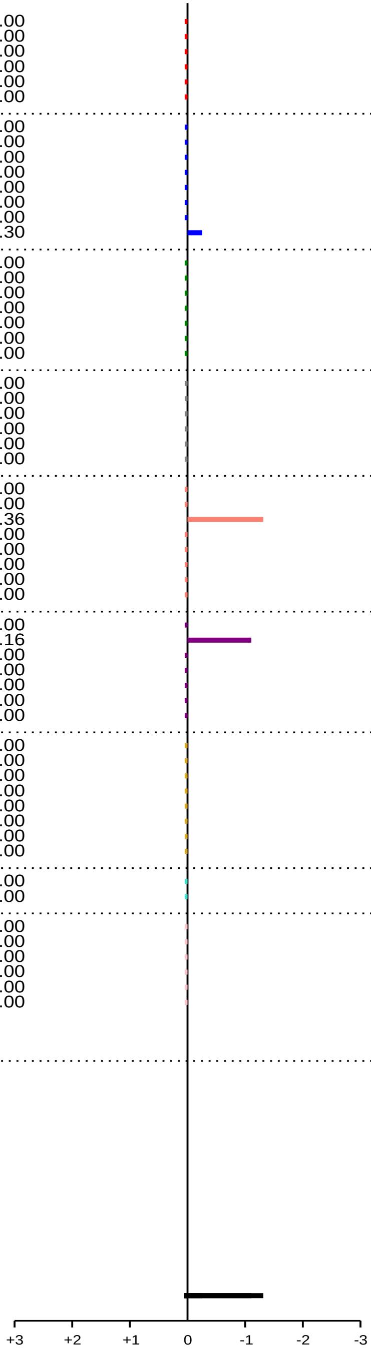

LC50

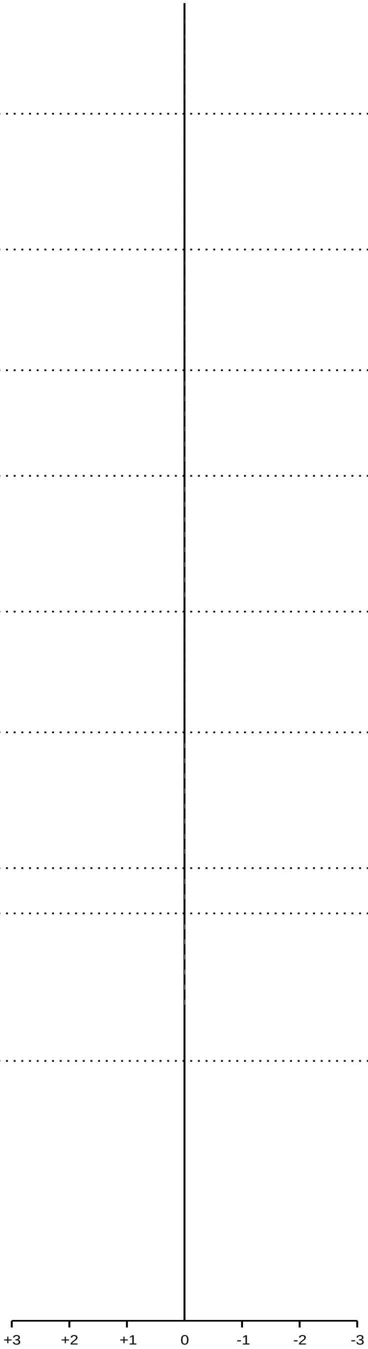

National Cancer Institute Developmental Therapeutics Program Dose Response Curves	NSC:766523/2	SSPL:0YQS	EXP. ID:1310NS81
	Report Date:July 13, 2015	Test Date:October 28, 2013	

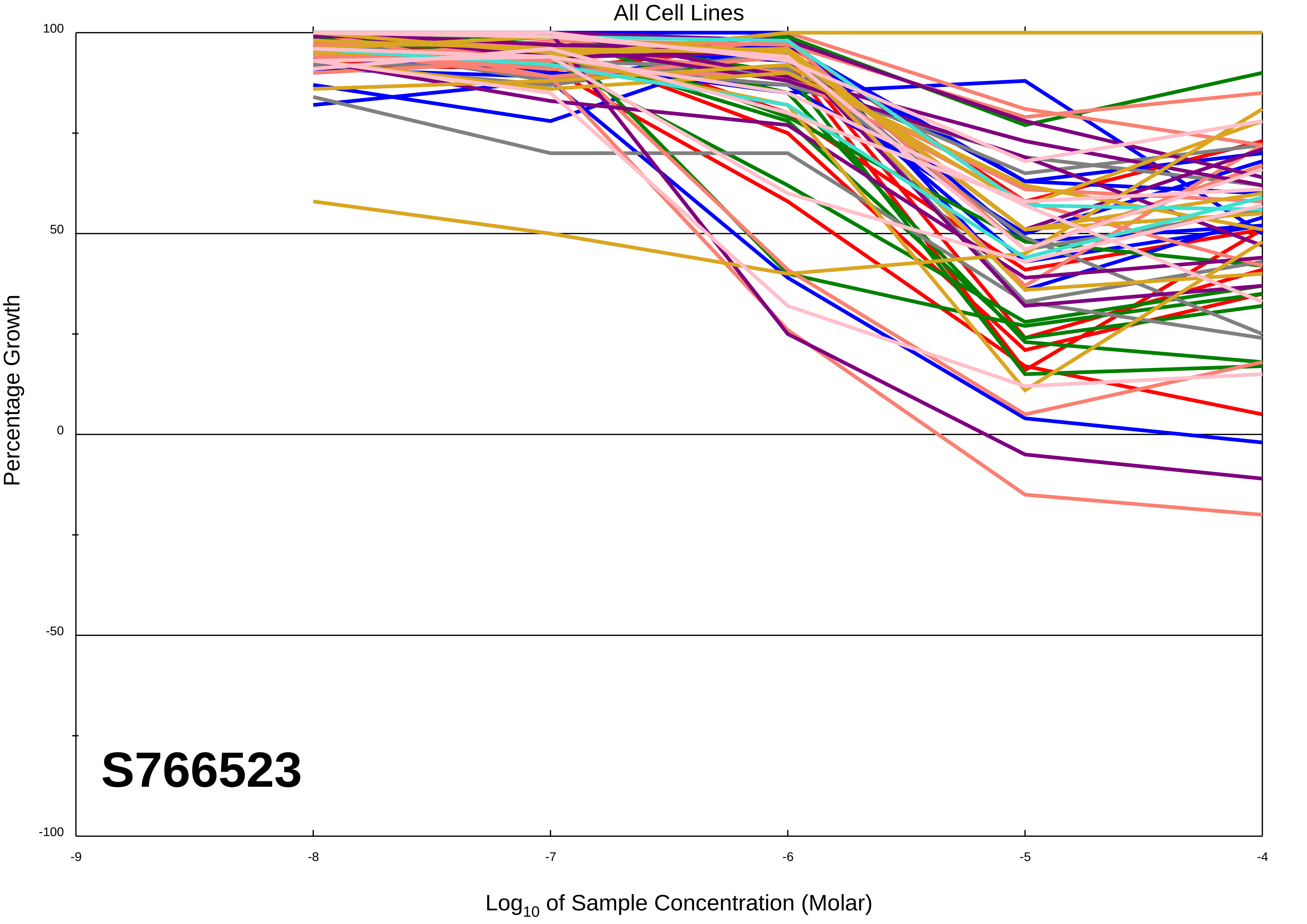

National Cancer Institute Developmental Therapeutics Program<br>Dose Response Curves	NSC: 766523 / 2	SSPL: 0YQS	EXP. ID: 1402RS24
	Report Date: July 13, 2015	Test Date: February 03, 2014	

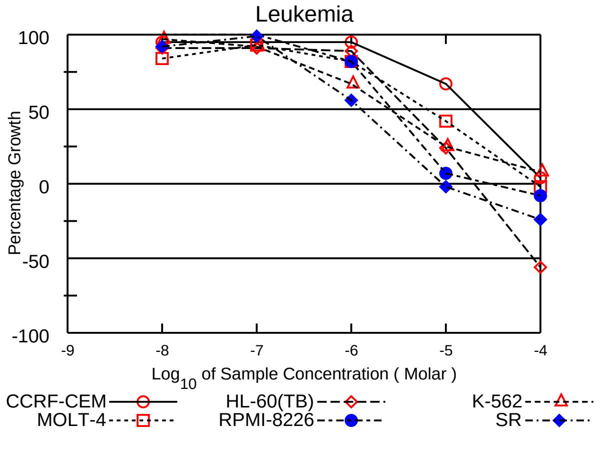

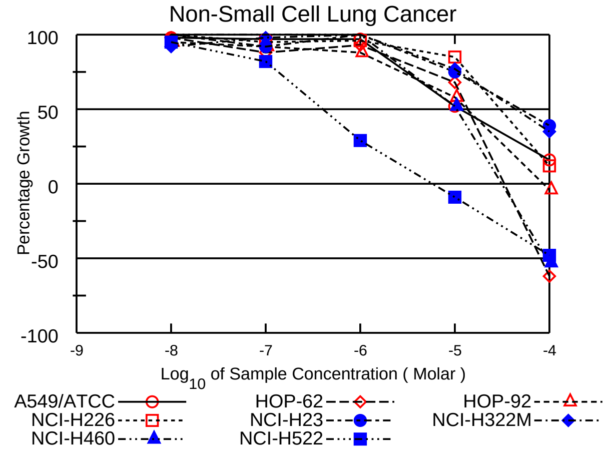

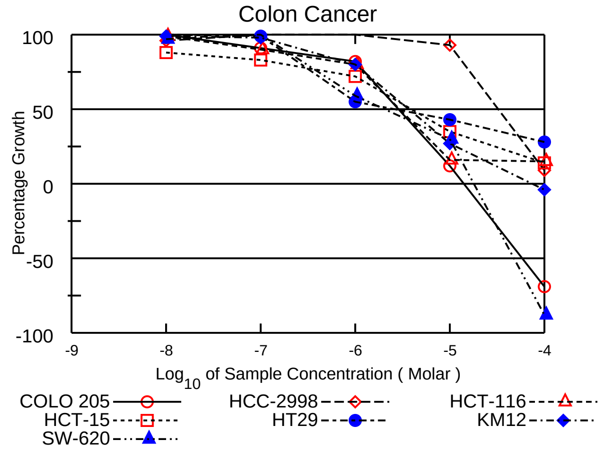

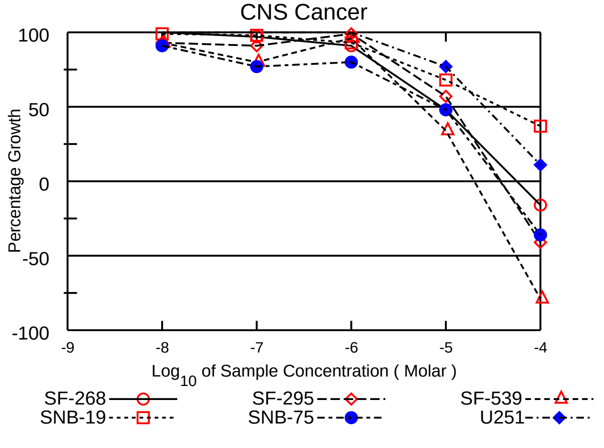

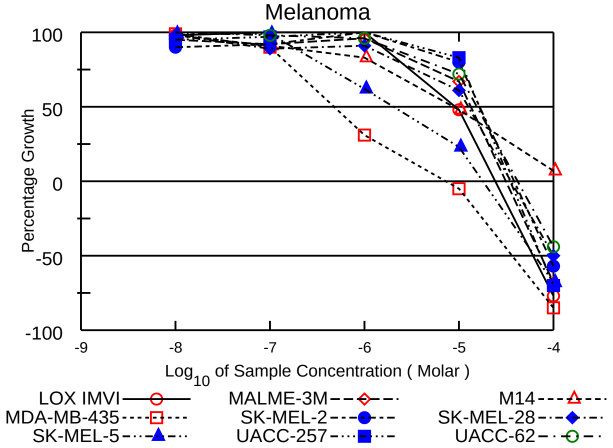

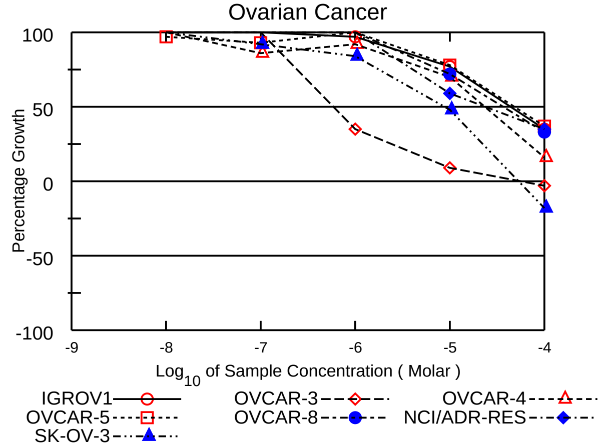

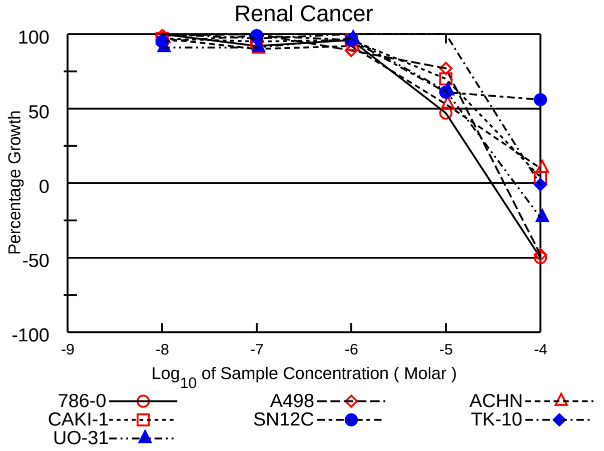

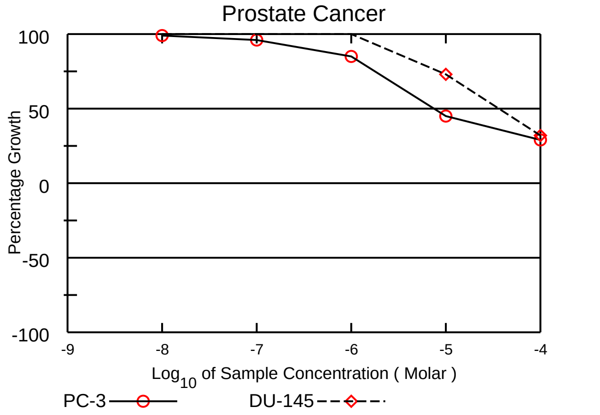

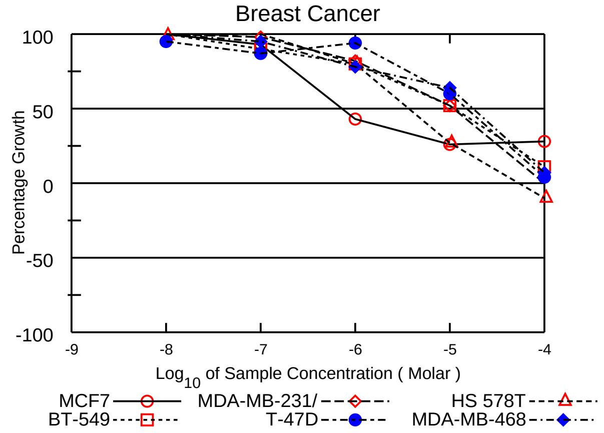

National Cancer Institute Developmental Therapeutics Program Mean Graphs	NSC :766523/2	Units :Molar	SSPL :0YQS	EXP. ID :1402RS24
	Report Date :July 13, 2015		Test Date :February 03, 2014	

Panel/Cell Line	Log_{10}GI50	Log_{10}TGI	Log_{10}LC50
**Leukemia**			
CCRF-CEM	-4.73	> -4.00	> -4.00
HL-60(TB)	-5.39	-4.70	-4.07
K-562	-5.59	> -4.00	> -4.00
MOLT-4	-5.21	-4.05	> -4.00
RPMI-8226	-5.57	-4.53	> -4.00
SR	-5.89	-5.03	> -4.00
**Non-Small Cell Lung Cancer**			
A549/ATCC	-4.95	> -4.00	> -4.00
HOP-62	-4.86	-4.48	-4.09
HOP-92	-4.87	-4.07	> -4.00
NCI-H226	-4.52	> -4.00	> -4.00
NCI-H23	-4.30	> -4.00	> -4.00
NCI-H322M	-4.35	> -4.00	> -4.00
NCI-H460	-4.98	-4.50	-4.03
NCI-H522	-6.40	-5.24	> -4.00
**Colon Cancer**			
COLO 205	-5.54	-4.85	-4.24
HCC-2998	-4.48	> -4.00	> -4.00
HCT-116	-5.53	> -4.00	> -4.00
HCT-15	-5.40	> -4.00	> -4.00
HT29	-5.60	> -4.00	> -4.00
KM12	-5.43	-4.13	> -4.00
SW-620	-5.69	-4.75	-4.32
**CNS Cancer**			
SF-268	-5.05	-4.25	> -4.00
SF-295	-4.93	-4.41	> -4.00
SF-539	-5.26	-4.70	-4.25
SNB-19	-4.43	> -4.00	> -4.00
SNB-75	-5.06	-4.42	> -4.00
U251	-4.59	> -4.00	> -4.00
**Melanoma**			
LOX IMVI	-5.03	-4.61	-4.21
MALME-3M	-4.88	-4.50	-4.13
M14	-5.05	> -4.00	> -4.00
MDA-MB-435	-6.32	-5.15	-4.44
SK-MEL-2	-4.78	-4.42	-4.05
SK-MEL-28	-4.90	-4.45	> -4.00
SK-MEL-5	-5.70	-4.75	-4.20
UACC-257	-4.79	-4.46	-4.13
UACC-62	-4.81	-4.38	> -4.00
**Ovarian Cancer**			
IGROV1	-4.36	> -4.00	> -4.00
OVCAR-3	-6.24	-4.24	> -4.00
OVCAR-4	-4.63	> -4.00	> -4.00
OVCAR-5	-4.32	> -4.00	> -4.00
OVCAR-8	-4.44	> -4.00	> -4.00
NCI/ADR-RES	-4.62	> -4.00	> -4.00
SK-OV-3	-5.04	-4.27	> -4.00
**Renal Cancer**			
786-0	-5.06	-4.51	-4.00
A498	-4.78	-4.39	> -4.00
ACHN	-4.93	> -4.00	> -4.00
CAKI-1	-4.70	> -4.00	> -4.00
SN12C	> -4.00	> -4.00	> -4.00
TK-10	-4.46	-4.01	> -4.00
UO-31	-4.85	-4.27	> -4.00
**Prostate Cancer**			
PC-3	-5.14	> -4.00	> -4.00
DU-145	-4.43	> -4.00	> -4.00
**Breast Cancer**			
MCF7	-6.14	> -4.00	> -4.00
MDA-MB-231/ATCC	-4.95	> -4.00	> -4.00
HS 578T	-5.43	-4.26	> -4.00
BT-549	-4.96	> -4.00	> -4.00
T-47D	-4.82	> -4.00	> -4.00
MDA-MB-468	-4.75	> -4.00	> -4.00
_MID	-5.03	-4.25	-4.04
Delta	1.37	0.99	0.4
Range	2.4	1.24	0.44

National Cancer Institute Developmental Therapeutics Program Dose Response Curves	NSC:766523/2	SSPL:0YQS	EXP. ID:1402RS24
	Report Date:July 13, 2015	Test Date:February 03, 2014	

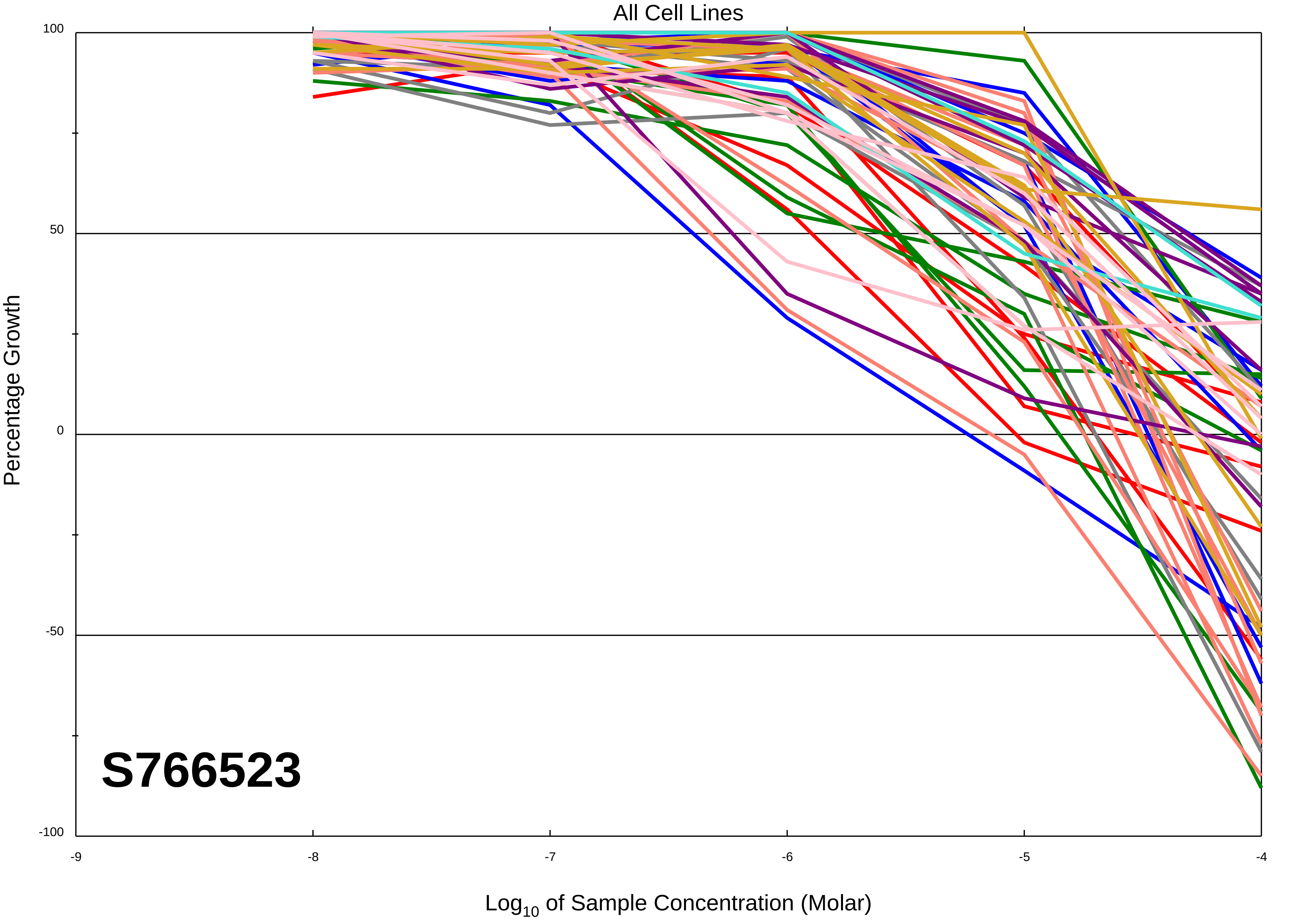

National Cancer Institute Developmental Therapeutics Program
Dose Response Curves

NSC: 766546 / 2

SSPL: 0YQS

EXP. ID: 1310NS81

Report Date: July 13, 2015

Test Date: October 28, 2013

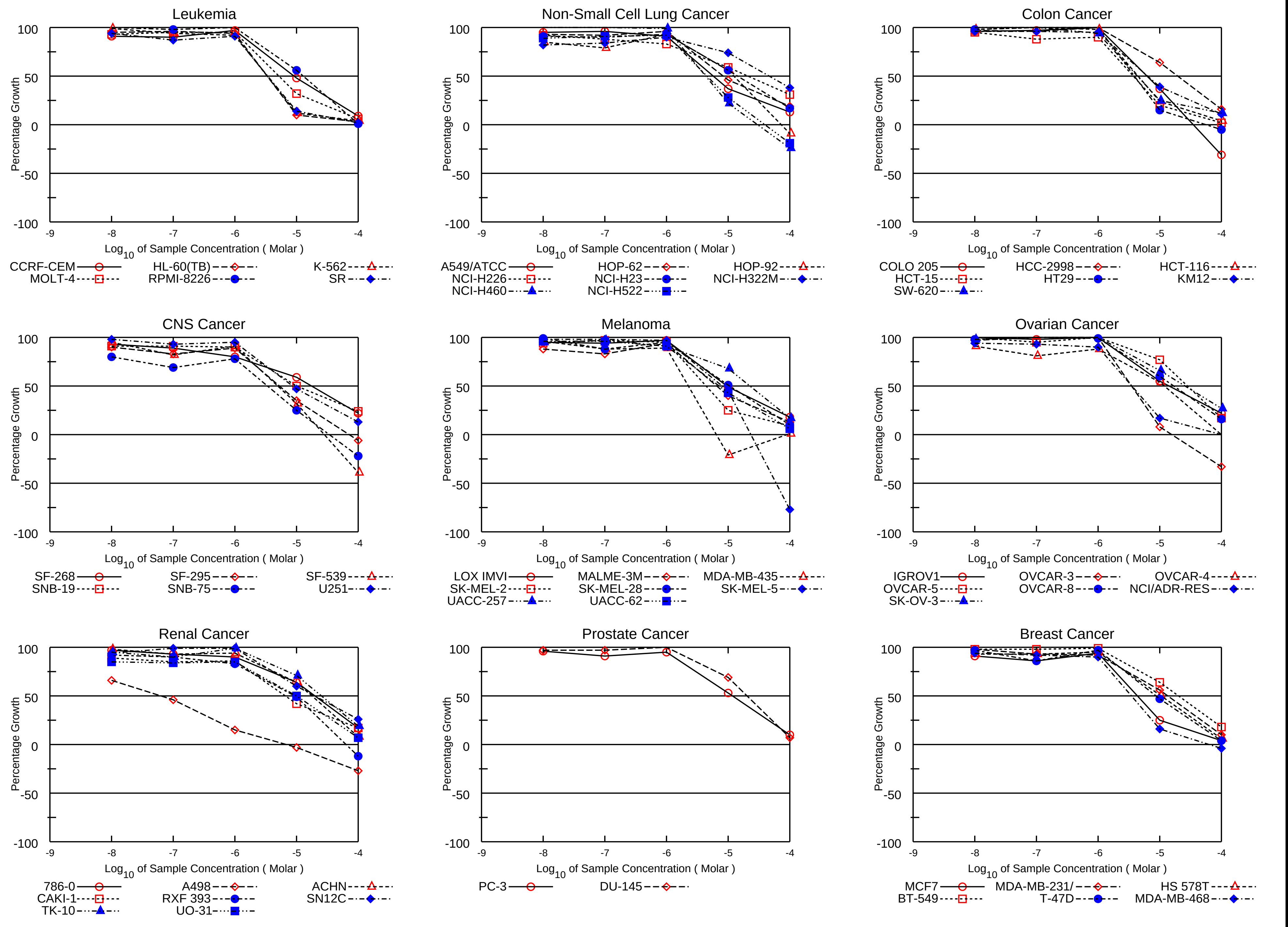

National Cancer Institute Developmental Therapeutics Program
Mean Graphs

NSC :766546/2 | Units :Molar | SSPL :0YQS | EXP. ID :1310NS81
Report Date :July 13, 2015 | Test Date :October 28, 2013

Panel/Cell Line	Log_{10}GI50	Log_{10}TGI	Log_{10}LC50
**Leukemia**			
CCRF-CEM	-5.03	> -4.00	> -4.00
HL-60(TB)	-5.48	> -4.00	> -4.00
K-562	-5.48	> -4.00	> -4.00
MOLT-4	-5.29	> -4.00	> -4.00
RPMI-8226	-4.90	> -4.00	> -4.00
SR	-5.47	> -4.00	> -4.00
**Non-Small Cell Lung Cancer**			
A549/ATCC	-5.24	> -4.00	> -4.00
HOP-62	-5.09	> -4.00	> -4.00
HOP-92	-4.91	-4.14	> -4.00
NCI-H226	-4.66	> -4.00	> -4.00
NCI-H23	-4.84	> -4.00	> -4.00
NCI-H322M	-4.33	> -4.00	> -4.00
NCI-H460	-5.36	-4.52	> -4.00
NCI-H522	-5.34	-4.41	> -4.00
**Colon Cancer**			
COLO 205	-5.20	-4.46	> -4.00
HCC-2998	-4.71	> -4.00	> -4.00
HCT-116	-5.35	> -4.00	> -4.00
HCT-15	-5.43	> -4.00	> -4.00
HT29	-5.41	-4.26	> -4.00
KM12	-5.19	> -4.00	> -4.00
SW-620	-5.36	> -4.00	> -4.00
**CNS Cancer**			
SF-268	-4.77	> -4.00	> -4.00
SF-295	-5.28	-4.15	> -4.00
SF-539	-5.31	-4.55	> -4.00
SNB-19	-5.00	> -4.00	> -4.00
SNB-75	-5.47	-4.47	> -4.00
U251	-5.07	> -4.00	> -4.00
**Melanoma**			
LOX IMVI	-5.03	> -4.00	> -4.00
MALME-3M	-5.18	> -4.00	> -4.00
MDA-MB-435	-5.65		> -4.00
SK-MEL-2	-5.35	> -4.00	> -4.00
SK-MEL-28	-4.98	> -4.00	> -4.00
SK-MEL-5	-5.10	-4.63	-4.22
UACC-257	-4.66	> -4.00	> -4.00
UACC-62	-5.15	> -4.00	> -4.00
**Ovarian Cancer**			
IGROV1	-4.86	> -4.00	> -4.00
OVCAR-3	-5.45	-4.81	> -4.00
OVCAR-4	-4.92	-4.01	> -4.00
OVCAR-5	-4.56	> -4.00	> -4.00
OVCAR-8	-4.77	> -4.00	> -4.00
NCI/ADR-RES	-5.45	> -4.00	> -4.00
SK-OV-3	-4.58	> -4.00	> -4.00
**Renal Cancer**			
786-0	-4.71	> -4.00	> -4.00
A498	-7.21	-5.17	> -4.00
ACHN	-4.75	> -4.00	> -4.00
CAKI-1	-5.18	> -4.00	> -4.00
RXF 393	-5.04	-4.20	> -4.00
SN12C	-4.71	> -4.00	> -4.00
TK-10	-4.60	> -4.00	> -4.00
UO-31	-5.01	> -4.00	> -4.00
**Prostate Cancer**			
PC-3	-4.92	> -4.00	> -4.00
DU-145	-4.69	> -4.00	> -4.00
**Breast Cancer**			
MCF7	-5.36	> -4.00	> -4.00
MDA-MB-231/ATCC	-4.87	> -4.00	> -4.00
HS 578T	-4.97	> -4.00	> -4.00
BT-549	-4.70	> -4.00	> -4.00
T-47D	-5.07	> -4.00	> -4.00
MDA-MB-468	-5.46	-4.20	> -4.00
_MID	-5.1	-4.1	-4.0
Delta	2.11	1.07	0.22
Range	2.88	1.17	0.22

Axis (GI50, TGI, LC50): +3 +2 +1 0 -1 -2 -3

National Cancer Institute Developmental Therapeutics Program<br>Dose Response Curves	NSC:766546/2	SSPL:0YQS	EXP. ID:1310NS81
	Report Date:July 13, 2015	Test Date:October 28, 2013	

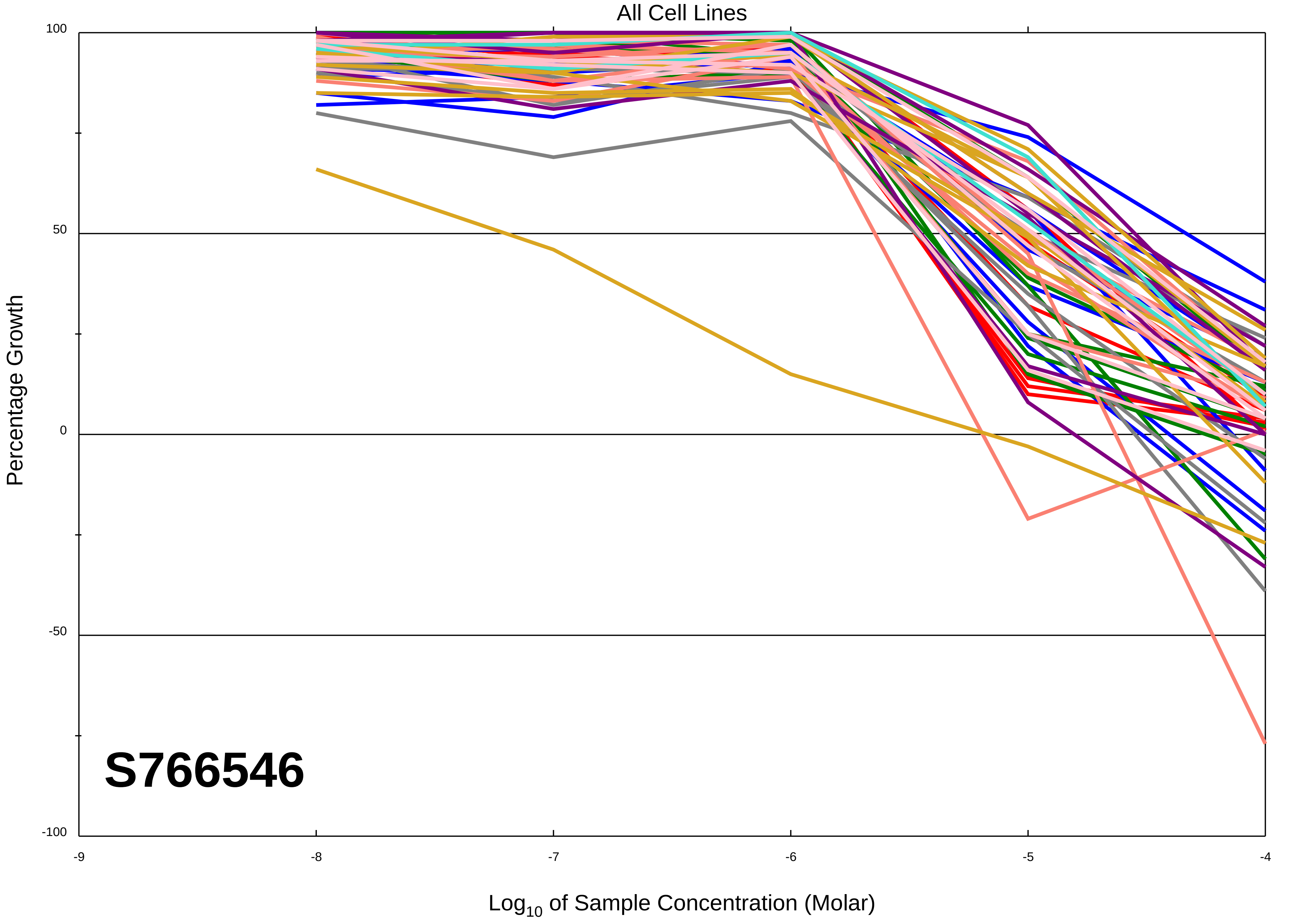

National Cancer Institute Developmental Therapeutics Program<br>Dose Response Curves	NSC: 766546 / 2	SSPL: 0YQS	EXP. ID: 1402RS24
	Report Date: July 13, 2015	Test Date: February 03, 2014	

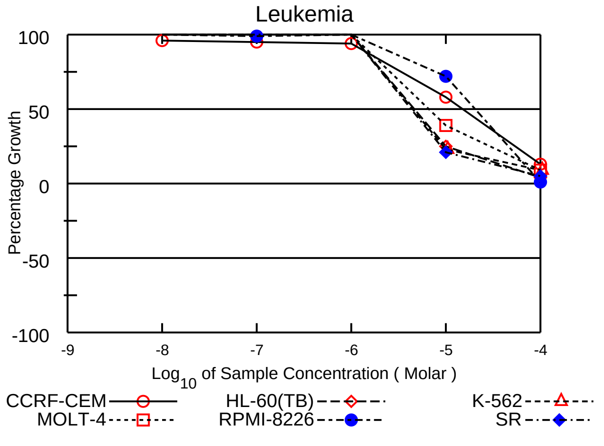

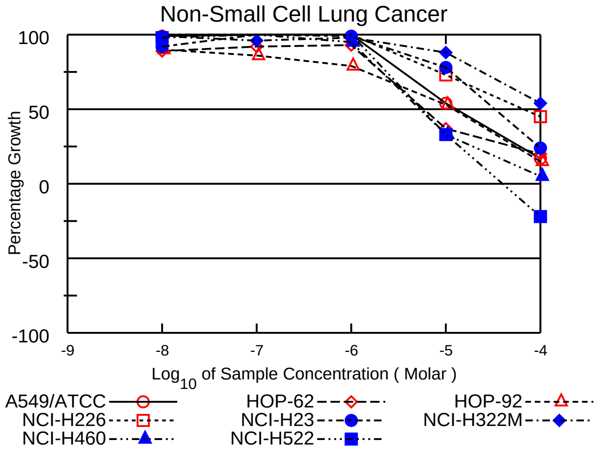

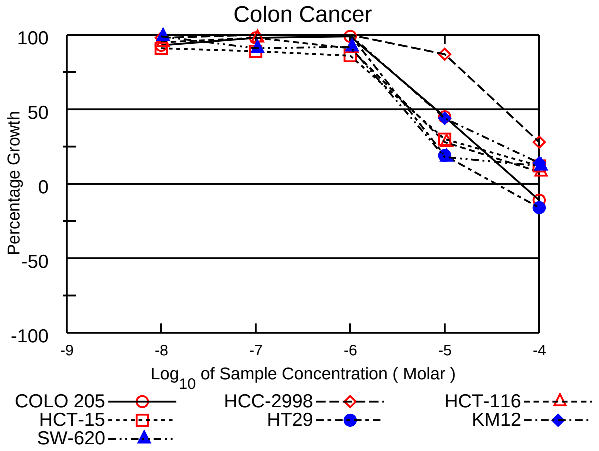

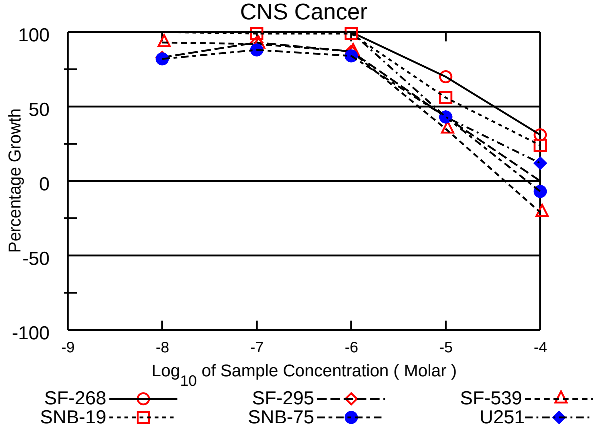

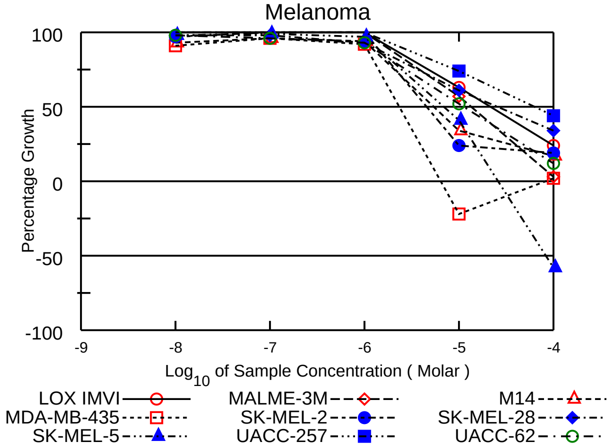

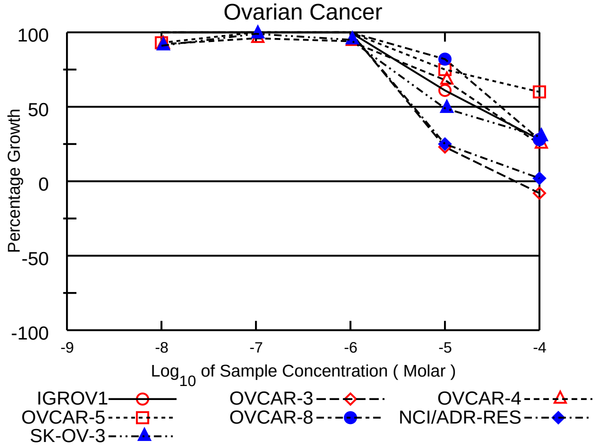

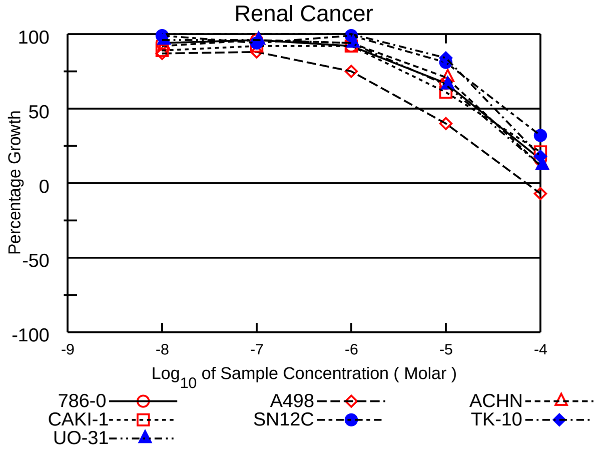

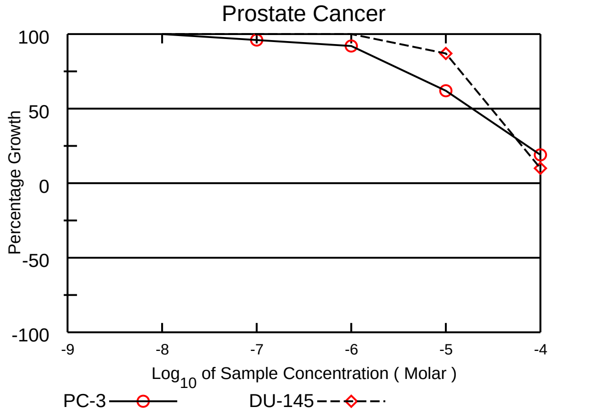

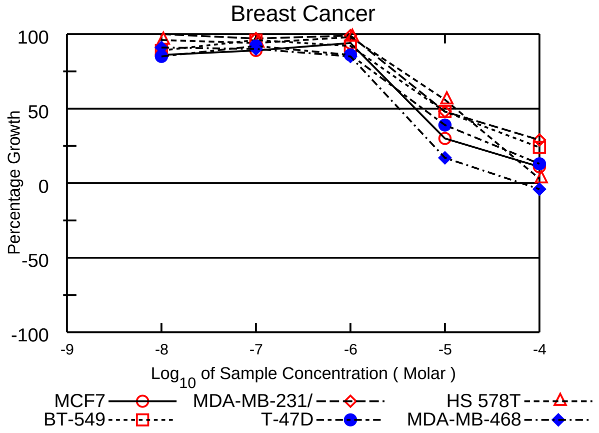

National Cancer Institute Developmental Therapeutics Program Mean Graphs	NSC :766546/2	Units :Molar	SSPL :0YQS	EXP. ID :1402RS24
	Report Date :July 13, 2015		Test Date :February 03, 2014	

Panel/Cell Line	Log_{10}GI50	Log_{10}TGI	Log_{10}LC50
**Leukemia**			
CCRF-CEM	-4.83	> -4.00	> -4.00
HL-60(TB)	-5.33	> -4.00	> -4.00
K-562	-5.34	> -4.00	> -4.00
MOLT-4	-5.17	> -4.00	> -4.00
RPMI-8226	-4.69	> -4.00	> -4.00
SR	-5.35	> -4.00	> -4.00
**Non-Small Cell Lung Cancer**			
A549/ATCC	-4.89	> -4.00	> -4.00
HOP-62	-5.23	> -4.00	> -4.00
HOP-92	-4.92	> -4.00	> -4.00
NCI-H226	-4.19	> -4.00	> -4.00
NCI-H23	-4.49	> -4.00	> -4.00
NCI-H322M	> -4.00	> -4.00	> -4.00
NCI-H460	-5.28	> -4.00	> -4.00
NCI-H522	-5.25	-4.40	> -4.00
**Colon Cancer**			
COLO 205	-5.09	-4.19	> -4.00
HCC-2998	-4.37	> -4.00	> -4.00
HCT-116	-5.35	> -4.00	> -4.00
HCT-15	-5.35	> -4.00	> -4.00
HT29	-5.34	-4.45	> -4.00
KM12	-5.09	> -4.00	> -4.00
SW-620	-5.43	> -4.00	> -4.00
**CNS Cancer**			
SF-268	-4.49	> -4.00	> -4.00
SF-295	-5.16	> -4.00	> -4.00
SF-539	-5.29	-4.38	> -4.00
SNB-19	-4.80	> -4.00	> -4.00
SNB-75	-5.17	-4.13	> -4.00
U251	-5.11	> -4.00	> -4.00
**Melanoma**			
LOX IMVI	-4.67	> -4.00	> -4.00
MALME-3M	-4.87	> -4.00	> -4.00
M14	-5.27	> -4.00	> -4.00
MDA-MB-435	-5.63		> -4.00
SK-MEL-2	-5.31	> -4.00	> -4.00
SK-MEL-28	-4.58	> -4.00	> -4.00
SK-MEL-5	-5.16	-4.58	-4.08
UACC-257	-4.21	> -4.00	> -4.00
UACC-62	-4.96	> -4.00	> -4.00
**Ovarian Cancer**			
IGROV1	-4.66	> -4.00	> -4.00
OVCAR-3	-5.30	-4.25	> -4.00
OVCAR-4	-4.57	> -4.00	> -4.00
OVCAR-5	> -4.00	> -4.00	> -4.00
OVCAR-8	-4.40	> -4.00	> -4.00
NCI/ADR-RES	-5.31	> -4.00	> -4.00
SK-OV-3	-5.03	> -4.00	> -4.00
**Renal Cancer**			
786-0	-4.67	> -4.00	> -4.00
A498	-5.29	-4.15	> -4.00
ACHN	-4.64	> -4.00	> -4.00
CAKI-1	-4.73	> -4.00	> -4.00
SN12C	-4.37	> -4.00	> -4.00
TK-10	-4.49	> -4.00	> -4.00
UO-31	-4.71	> -4.00	> -4.00
**Prostate Cancer**			
PC-3	-4.72	> -4.00	> -4.00
DU-145	-4.52	> -4.00	> -4.00
**Breast Cancer**			
MCF7	-5.31	> -4.00	> -4.00
MDA-MB-231/ATCC	-5.04	> -4.00	> -4.00
HS 578T	-4.89	> -4.00	> -4.00
BT-549	-5.05	> -4.00	> -4.00
T-47D	-5.23	> -4.00	> -4.00
MDA-MB-468	-5.48	-4.18	> -4.00
_MID	-4.93	-4.05	-4.0
Delta	0.7	0.53	0.08
Range	1.63	0.58	0.08

Axis scale (GI50, TGI, LC50): +3 +2 +1 0 -1 -2 -3

National Cancer Institute Developmental Therapeutics Program<br>Dose Response Curves	NSC:766546/2	SSPL:0YQS	EXP. ID:1402RS24
	Report Date:July 13, 2015	Test Date:February 03, 2014	

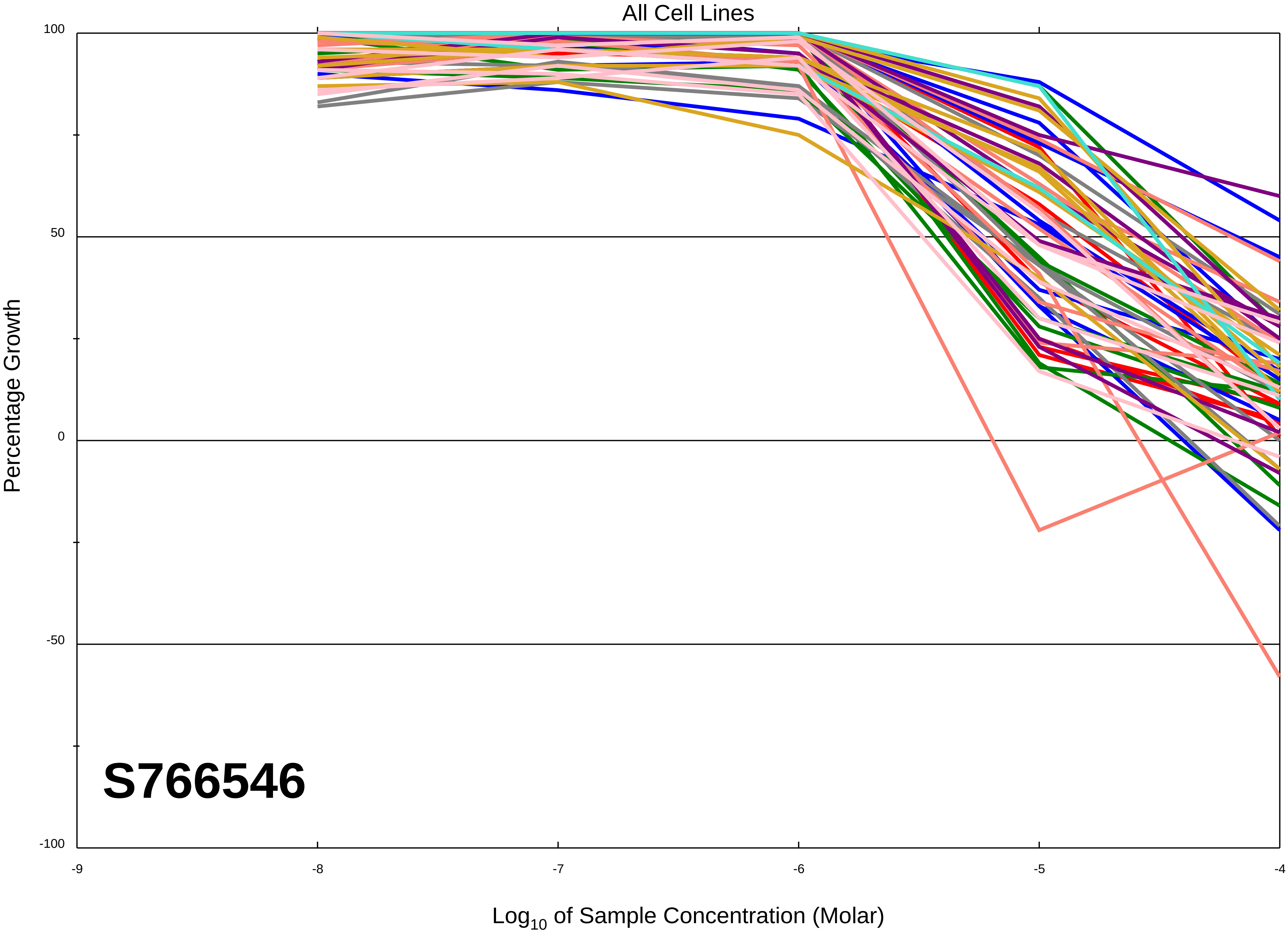

National Cancer Institute Developmental Therapeutics Program Dose Response Curves	NSC: 766556 / 2	SSPL: 0YQS	EXP. ID: 1310NS81
	Report Date: July 13, 2015	Test Date: October 28, 2013	

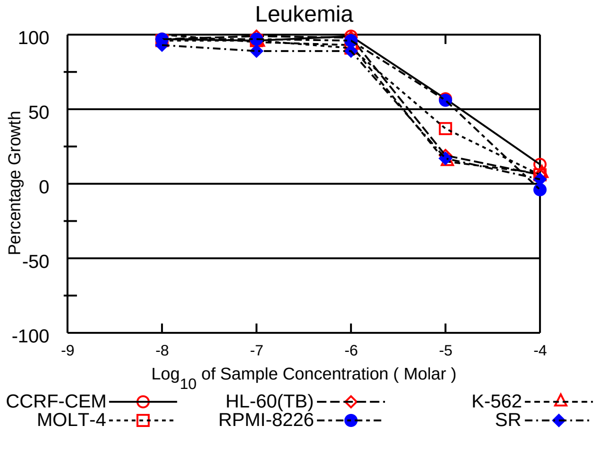

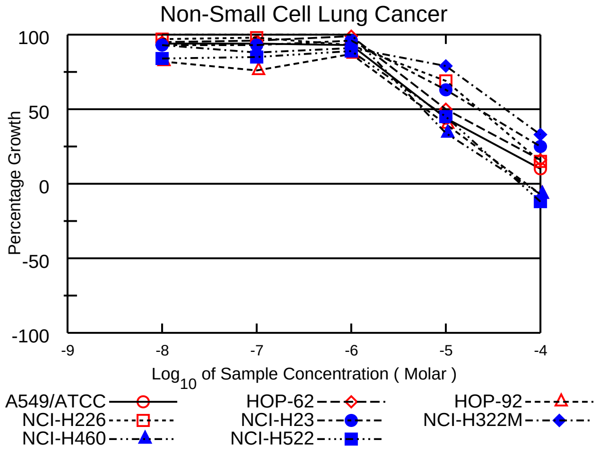

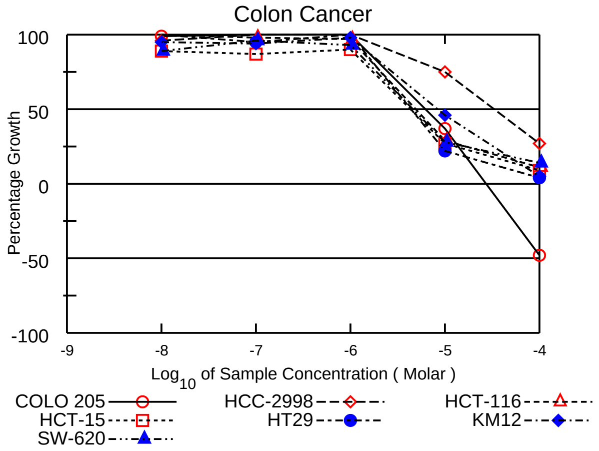

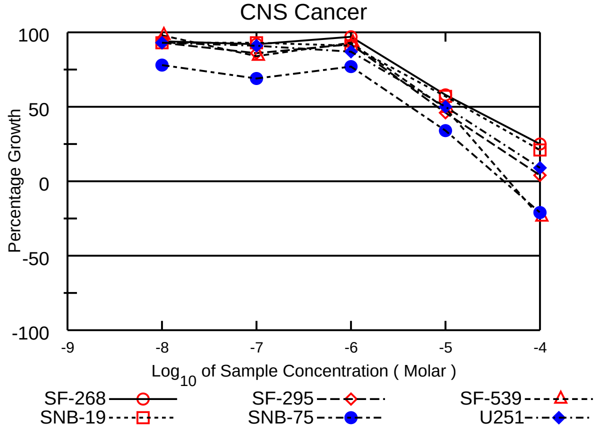

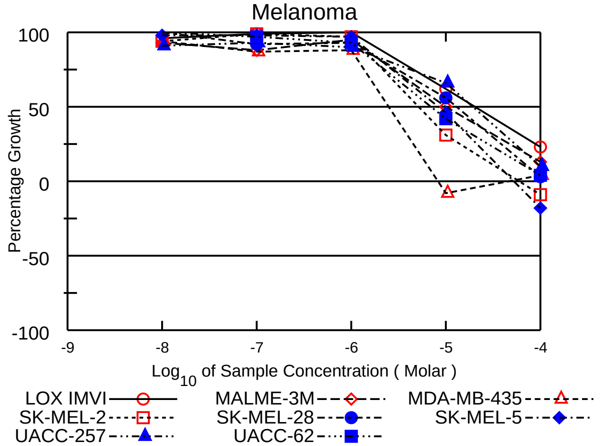

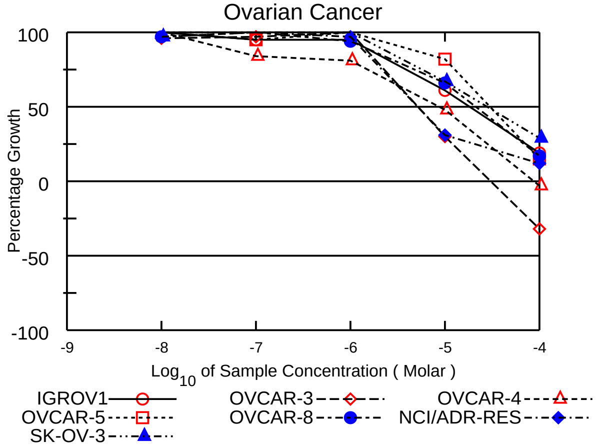

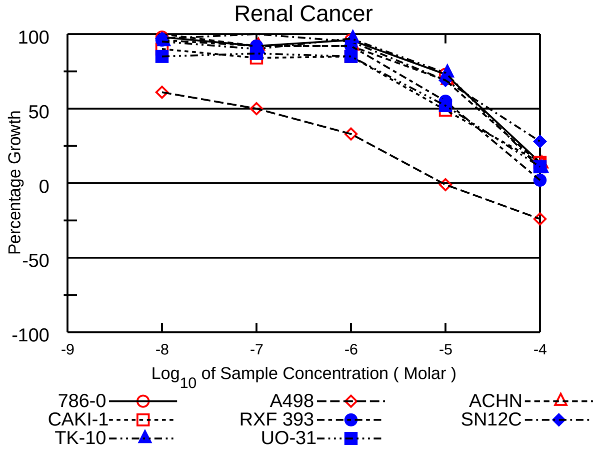

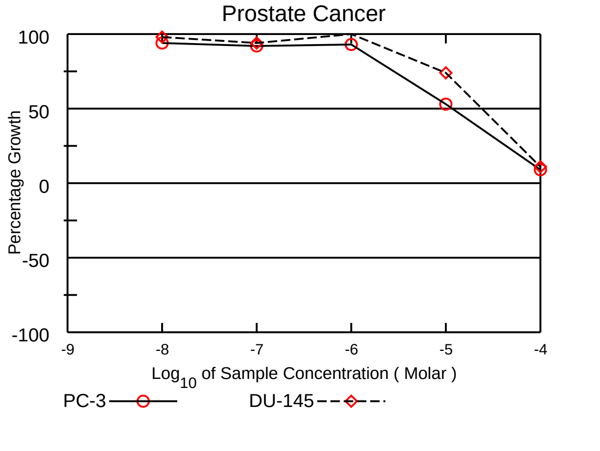

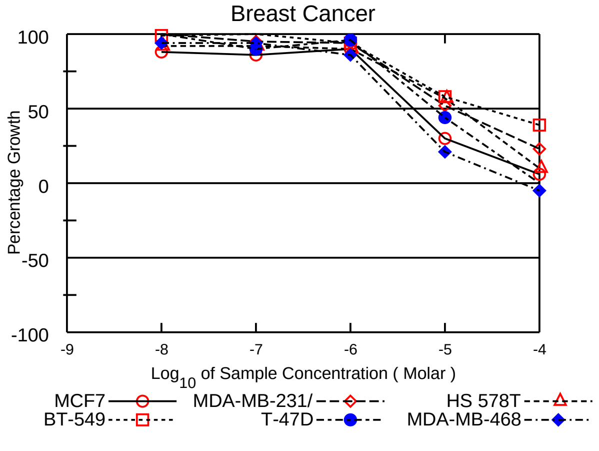

National Cancer Institute Developmental Therapeutics Program
Mean Graphs

NSC :766556/2 | Units :Molar | SSPL :0YQS | EXP. ID :1310NS81
Report Date :July 13, 2015 | Test Date :October 28, 2013

Panel/Cell Line	Log_{10}GI50	Log_{10}TGI	Log_{10}LC50
**Leukemia**			
CCRF-CEM	-4.84	> -4.00	> -4.00
HL-60(TB)	-5.39	> -4.00	> -4.00
K-562	-5.45	> -4.00	> -4.00
MOLT-4	-5.23	> -4.00	> -4.00
RPMI-8226	-4.90	-4.07	> -4.00
SR	-5.46	> -4.00	> -4.00
**Non-Small Cell Lung Cancer**			
A549/ATCC	-5.12	> -4.00	> -4.00
HOP-62	-5.01	> -4.00	> -4.00
HOP-92	-5.21	-4.16	> -4.00
NCI-H226	-4.65	> -4.00	> -4.00
NCI-H23	-4.65	> -4.00	> -4.00
NCI-H322M	-4.37	> -4.00	> -4.00
NCI-H460	-5.24	-4.18	> -4.00
NCI-H522	-5.11	-4.21	> -4.00
**Colon Cancer**			
COLO 205	-5.20	-4.57	> -4.00
HCC-2998	-4.48	> -4.00	> -4.00
HCT-116	-5.30	> -4.00	> -4.00
HCT-15	-5.36	> -4.00	> -4.00
HT29	-5.34	> -4.00	> -4.00
KM12	-5.08	> -4.00	> -4.00
SW-620	-5.34	> -4.00	> -4.00
**CNS Cancer**			
SF-268	-4.75	> -4.00	> -4.00
SF-295	-5.08	> -4.00	> -4.00
SF-539	-5.02	-4.33	> -4.00
SNB-19	-4.80	> -4.00	> -4.00
SNB-75	-5.36	-4.38	> -4.00
U251	-5.00	> -4.00	> -4.00
**Melanoma**			
LOX IMVI	-4.70	> -4.00	> -4.00
MALME-3M	-5.00	> -4.00	> -4.00
MDA-MB-435	-5.60		> -4.00
SK-MEL-2	-5.28	-4.23	> -4.00
SK-MEL-28	-4.89	> -4.00	> -4.00
SK-MEL-5	-5.07	-4.28	> -4.00
UACC-257	-4.71	> -4.00	> -4.00
UACC-62	-5.16	> -4.00	> -4.00
**Ovarian Cancer**			
IGROV1	-4.74	> -4.00	> -4.00
OVCAR-3	-5.28	-4.51	> -4.00
OVCAR-4	-5.06	-4.06	> -4.00
OVCAR-5	-4.52	> -4.00	> -4.00
OVCAR-8	-4.67	> -4.00	> -4.00
NCI/ADR-RES	-5.29	> -4.00	> -4.00
SK-OV-3	-4.56	> -4.00	> -4.00
**Renal Cancer**			
786-0	-4.61	> -4.00	> -4.00
A498	-6.98	-5.02	> -4.00
ACHN	-4.66	> -4.00	> -4.00
CAKI-1	-5.03	> -4.00	> -4.00
RXF 393	-4.90	> -4.00	> -4.00
SN12C	-4.54	> -4.00	> -4.00
TK-10	-4.62	> -4.00	> -4.00
UO-31	-4.96	> -4.00	> -4.00
**Prostate Cancer**			
PC-3	-4.94	> -4.00	> -4.00
DU-145	-4.62	> -4.00	> -4.00
**Breast Cancer**			
MCF7	-5.33	> -4.00	> -4.00
MDA-MB-231/ATCC	-4.93	> -4.00	> -4.00
HS 578T	-4.85	> -4.00	> -4.00
BT-549	-4.57	> -4.00	> -4.00
T-47D	-5.12	> -4.00	> -4.00
MDA-MB-468	-5.45	-4.18	> -4.00
_MID	-5.02	-4.07	-4.0
Delta	1.96	0.95	0
Range	2.61	1.02	0.0

GI50

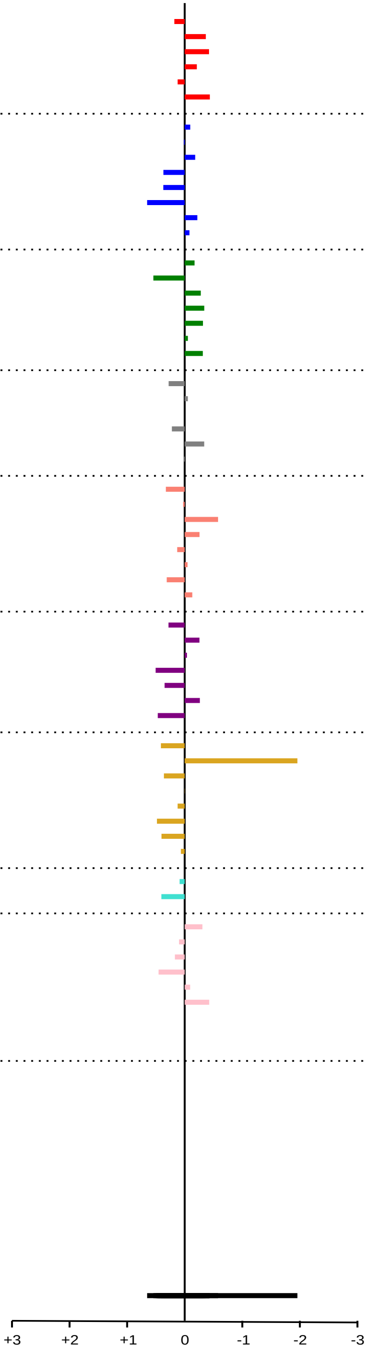

TGI

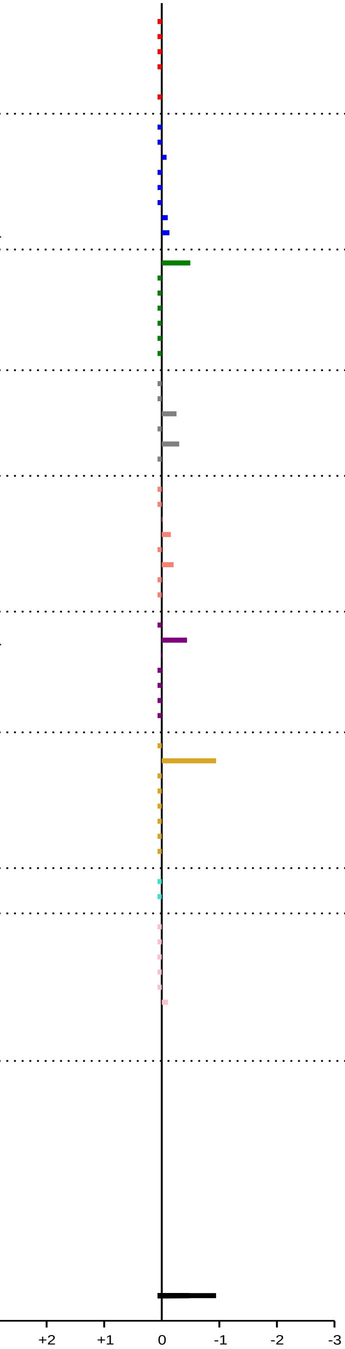

LC50

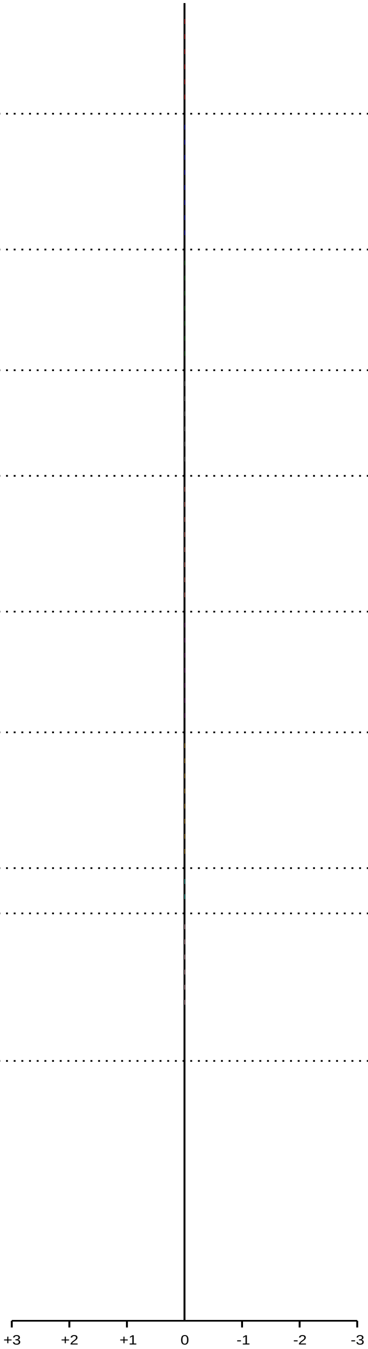

National Cancer Institute Developmental Therapeutics Program<br>Dose Response Curves	NSC:766556/2	SSPL:0YQS	EXP. ID:1310NS81
	Report Date:July 13, 2015	Test Date:October 28, 2013	

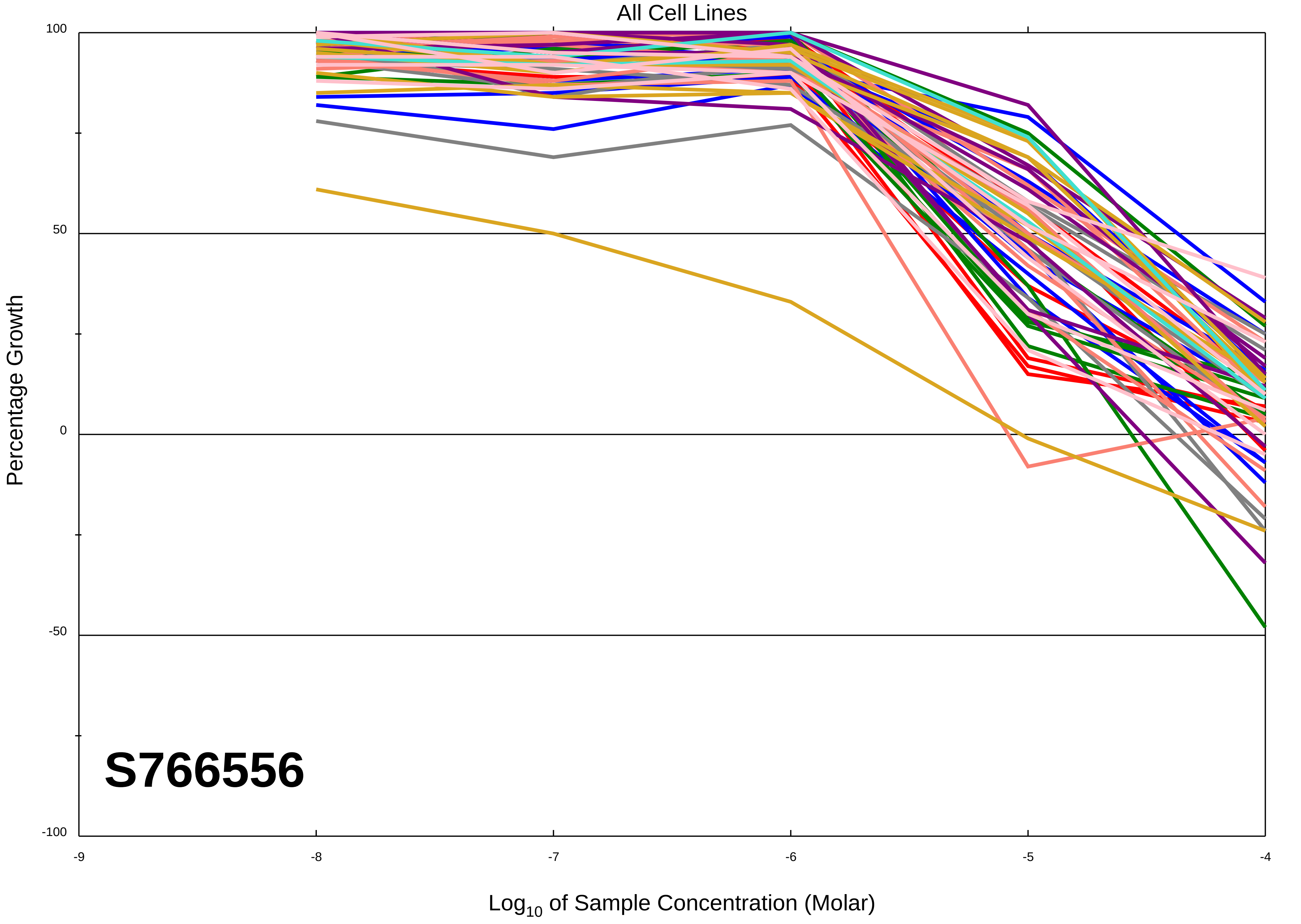

National Cancer Institute Developmental Therapeutics Program
Dose Response Curves

NSC: 766556 / 2	SSPL: 0YQS	EXP. ID: 1402RS24
Report Date: July 13, 2015	Test Date: February 03, 2014	

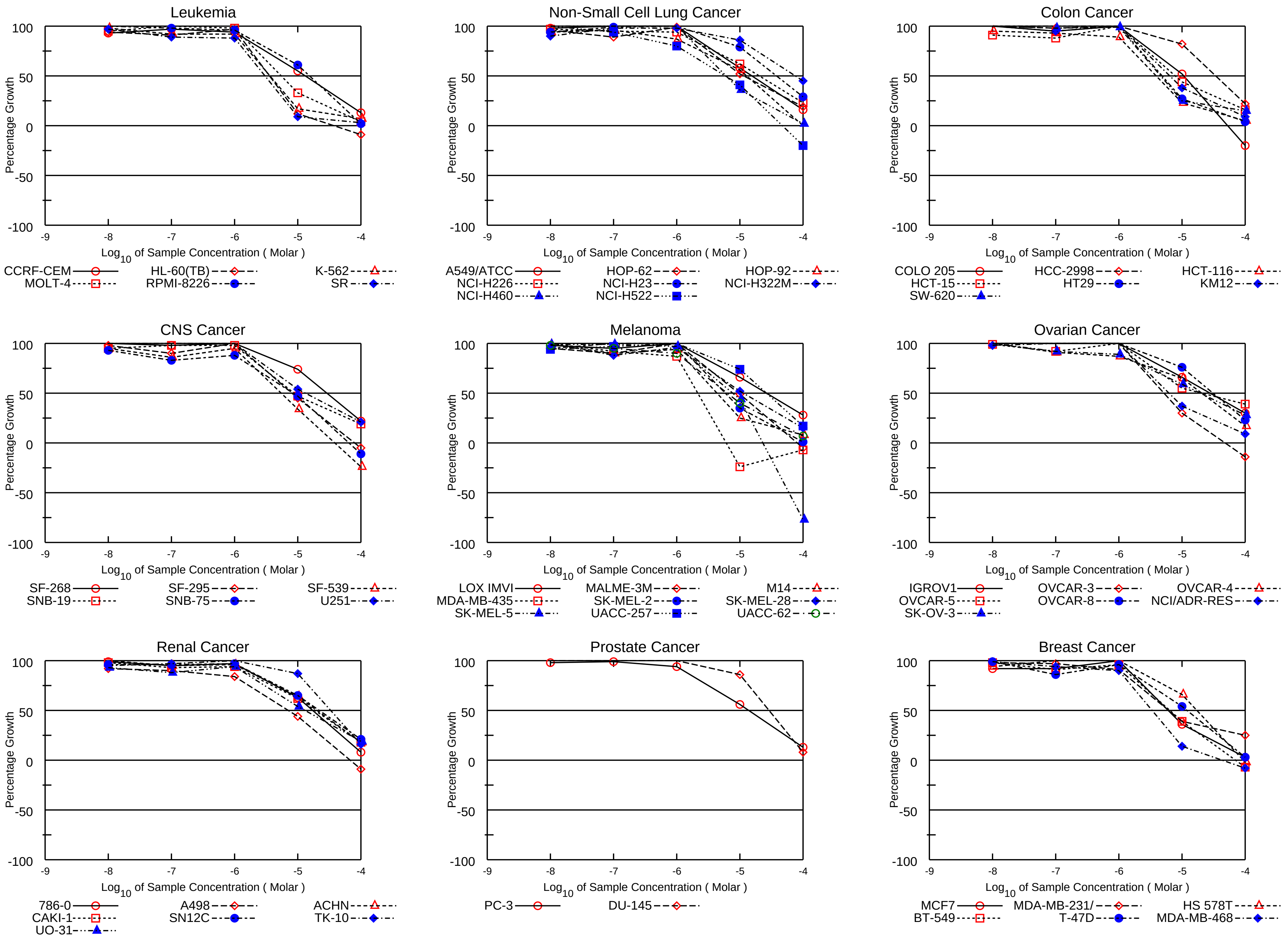

National Cancer Institute Developmental Therapeutics Program Mean Graphs	NSC :766556/2	Units :Molar	SSPL :0YQS	EXP. ID :1402RS24
	Report Date :July 13, 2015		Test Date :February 03, 2014	

Panel/Cell Line	Log_{10}GI50	Log_{10}TGI	Log_{10}LC50
**Leukemia**			
CCRF-CEM	-4.89	> -4.00	> -4.00
HL-60(TB)	-5.45	-4.42	> -4.00
K-562	-5.44	> -4.00	> -4.00
MOLT-4	-5.26	> -4.00	> -4.00
RPMI-8226	-4.81	> -4.00	> -4.00
SR	-5.51	> -4.00	> -4.00
**Non-Small Cell Lung Cancer**			
A549/ATCC	-4.81	> -4.00	> -4.00
HOP-62	-4.94	> -4.00	> -4.00
HOP-92	-4.89	> -4.00	> -4.00
NCI-H226	-4.69	> -4.00	> -4.00
NCI-H23	-4.42	> -4.00	> -4.00
NCI-H322M	-4.12	> -4.00	> -4.00
NCI-H460	-5.20	> -4.00	> -4.00
NCI-H522	-5.22	-4.32	> -4.00
**Colon Cancer**			
COLO 205	-4.97	-4.28	> -4.00
HCC-2998	-4.46	> -4.00	> -4.00
HCT-116	-5.41	> -4.00	> -4.00
HCT-15	-5.11	> -4.00	> -4.00
HT29	-5.26	> -4.00	> -4.00
KM12	-5.19	> -4.00	> -4.00
SW-620	-5.33	> -4.00	> -4.00
**CNS Cancer**			
SF-268	-4.54	> -4.00	> -4.00
SF-295	-5.08	-4.10	> -4.00
SF-539	-5.26	-4.41	> -4.00
SNB-19	-5.05	> -4.00	> -4.00
SNB-75	-5.08	-4.20	> -4.00
U251	-4.88	> -4.00	> -4.00
**Melanoma**			
LOX IMVI	-4.59	> -4.00	> -4.00
MALME-3M	-5.01	-4.10	> -4.00
M14	-5.36	> -4.00	> -4.00
MDA-MB-435	-5.66	-5.21	> -4.00
SK-MEL-2	-5.23	> -4.00	> -4.00
SK-MEL-28	-4.95	> -4.00	> -4.00
SK-MEL-5	-5.11	-4.64	-4.22
UACC-257	-4.58	> -4.00	> -4.00
UACC-62	-5.20	> -4.00	> -4.00
**Ovarian Cancer**			
IGROV1	-4.56	> -4.00	> -4.00
OVCAR-3	-5.25	-4.31	> -4.00
OVCAR-4	-4.69	> -4.00	> -4.00
OVCAR-5	-4.67	> -4.00	> -4.00
OVCAR-8	-4.51	> -4.00	> -4.00
NCI/ADR-RES	-5.20	> -4.00	> -4.00
SK-OV-3	-4.72	> -4.00	> -4.00
**Renal Cancer**			
786-0	-4.77	> -4.00	> -4.00
A498	-5.14	-4.17	> -4.00
ACHN	-4.69	> -4.00	> -4.00
CAKI-1	-4.73	> -4.00	> -4.00
SN12C	-4.65	> -4.00	> -4.00
TK-10	-4.48	> -4.00	> -4.00
UO-31	-4.87	> -4.00	> -4.00
**Prostate Cancer**			
PC-3	-4.86	> -4.00	> -4.00
DU-145	-4.54	> -4.00	> -4.00
**Breast Cancer**			
MCF7	-5.21	> -4.00	> -4.00
MDA-MB-231/ATCC	-5.21	> -4.00	> -4.00
HS 578T	-4.76	-4.03	> -4.00
BT-549	-5.19	-4.15	> -4.00
T-47D	-4.92	> -4.00	> -4.00
MDA-MB-468	-5.47	-4.34	> -4.00
_MID	-4.97	-4.08	-4.0
Delta	0.69	1.13	0.22
Range	1.54	1.21	0.22

National Cancer Institute Developmental Therapeutics Program Dose Response Curves	NSC:766556/2	SSPL:0YQS	EXP. ID:1402RS24
	Report Date:July 13, 2015	Test Date:February 03, 2014	

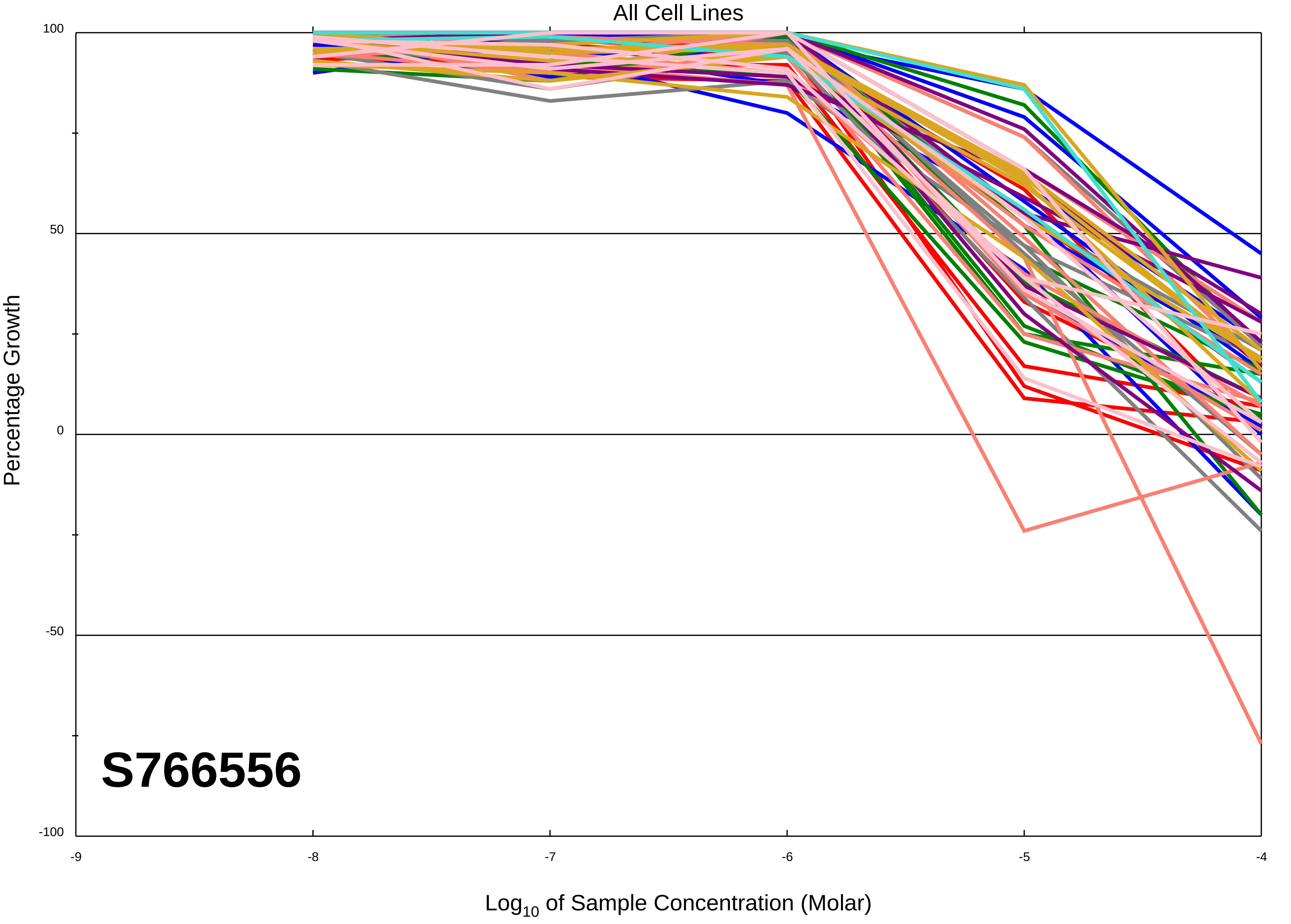

National Cancer Institute Developmental Therapeutics Program Dose Response Curves	NSC: 766585 / 2	SSPL: 0YQS	EXP. ID: 1310NS81
	Report Date: July 13, 2015	Test Date: October 28, 2013	

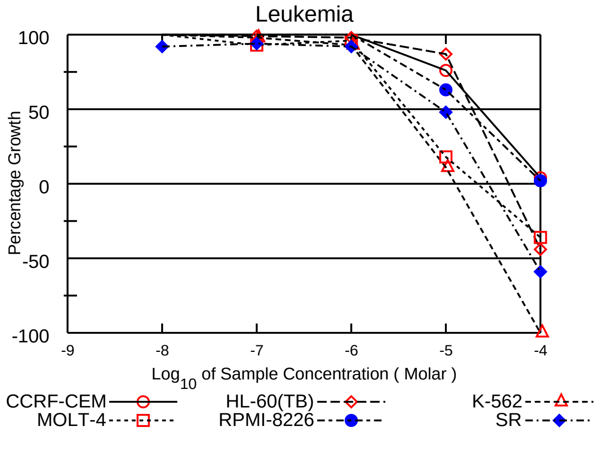

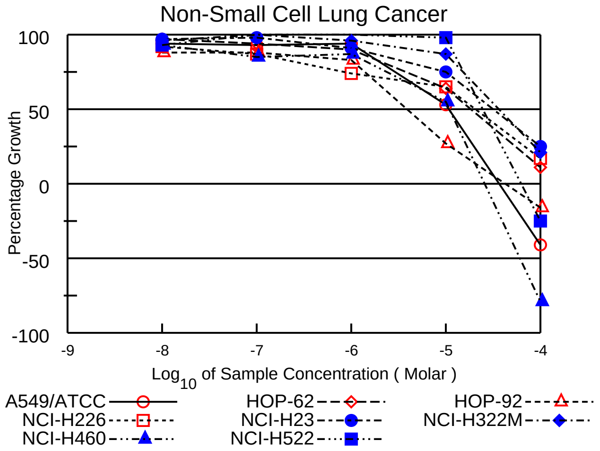

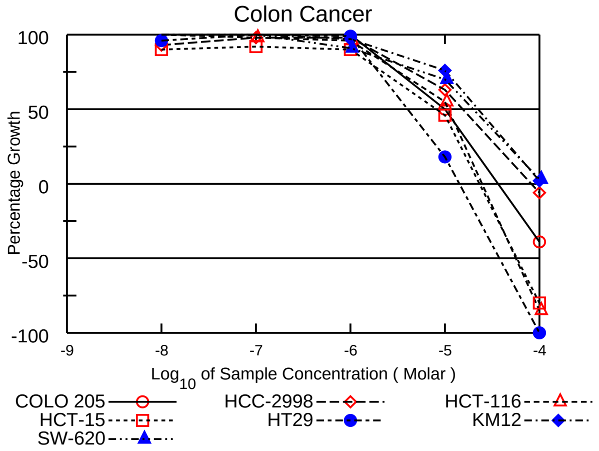

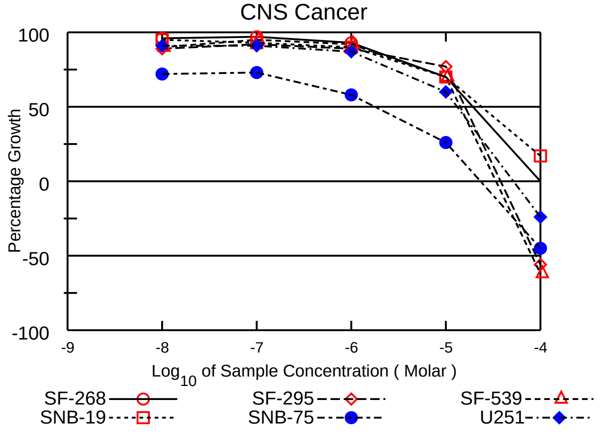

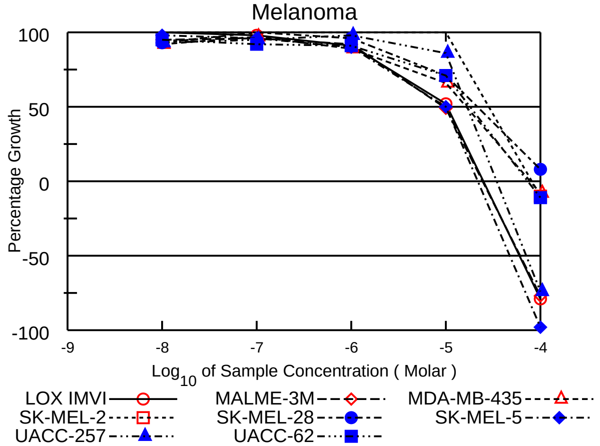

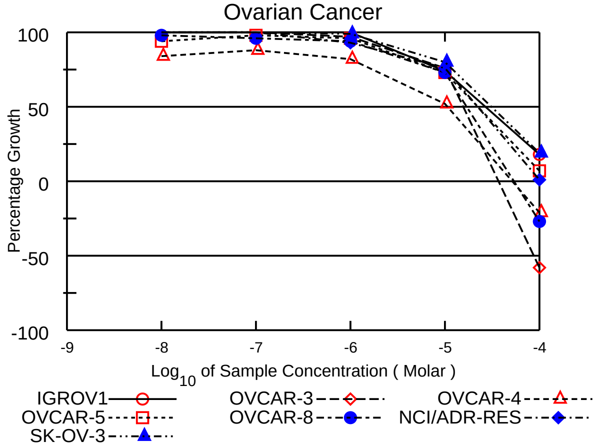

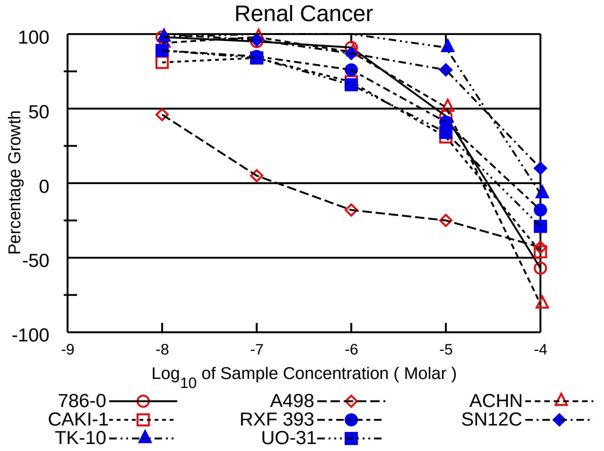

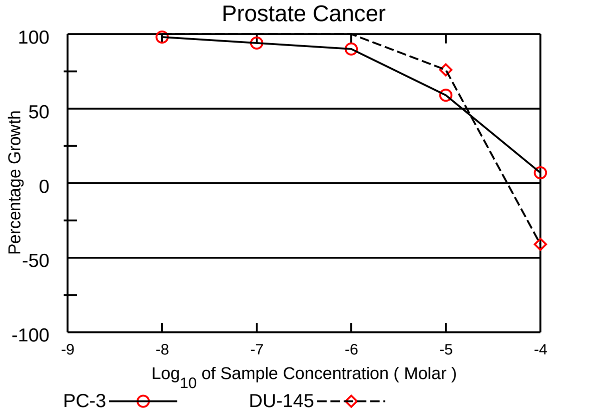

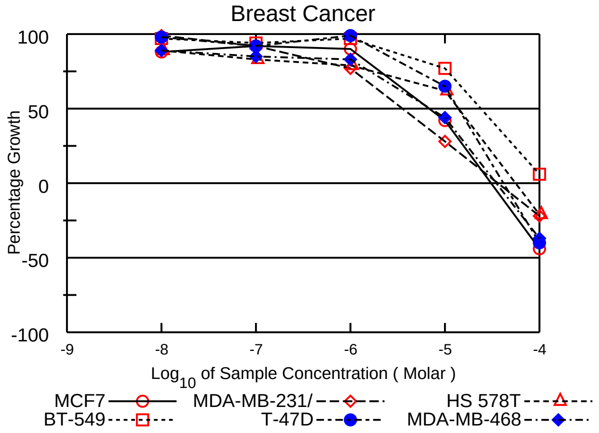

National Cancer Institute Developmental Therapeutics Program Mean Graphs	NSC :766585/2	Units :Molar	SSPL :0YQS	EXP. ID :1310NS81
	Report Date :July 13, 2015		Test Date :October 28, 2013	

Panel/Cell Line	Log_{10} GI50	Log_{10} TGI	Log_{10} LC50
**Leukemia**			
CCRF-CEM	-4.64	> -4.00	> -4.00
HL-60(TB)	-4.72	-4.34	> -4.00
K-562	-5.48	-4.90	-4.45
MOLT-4	-5.41	-4.67	> -4.00
RPMI-8226	-4.79	> -4.00	> -4.00
SR	-5.04	-4.55	-4.08
**Non-Small Cell Lung Cancer**			
A549/ATCC	-4.97	-4.44	> -4.00
HOP-62	-4.73	> -4.00	> -4.00
HOP-92	-5.41	-4.37	> -4.00
NCI-H226	-4.68	> -4.00	> -4.00
NCI-H23	-4.50	> -4.00	> -4.00
NCI-H322M	-4.44	> -4.00	> -4.00
NCI-H460	-4.96	-4.59	-4.21
NCI-H522	-4.61	-4.20	> -4.00
**Colon Cancer**			
COLO 205	-5.01	-4.44	> -4.00
HCC-2998	-4.81	-4.08	> -4.00
HCT-116	-4.97	-4.61	-4.25
HCT-15	-5.09	-4.63	-4.24
HT29	-5.39	-4.84	-4.42
KM12	-4.65	> -4.00	> -4.00
SW-620	-4.70	> -4.00	> -4.00
**CNS Cancer**			
SF-268	-4.71	> -4.00	> -4.00
SF-295	-4.80	-4.42	-4.05
SF-539	-4.85	-4.47	-4.09
SNB-19	-4.63	> -4.00	> -4.00
SNB-75	-5.75	-4.63	> -4.00
U251	-4.88	-4.29	> -4.00
**Melanoma**			
LOX IMVI	-4.98	-4.60	-4.22
MALME-3M	-5.02	-4.61	-4.21
MDA-MB-435	-4.78	-4.10	> -4.00
SK-MEL-2	-4.49	-4.08	> -4.00
SK-MEL-28	-4.67	> -4.00	> -4.00
SK-MEL-5	-5.00	-4.66	-4.32
UACC-257	-4.78	-4.46	-4.15
UACC-62	-4.74	-4.14	> -4.00
**Ovarian Cancer**			
IGROV1	-4.58	> -4.00	> -4.00
OVCAR-3	-4.81	-4.43	-4.06
OVCAR-4	-4.97	-4.29	> -4.00
OVCAR-5	-4.65	> -4.00	> -4.00
OVCAR-8	-4.77	-4.27	> -4.00
NCI/ADR-RES	-4.66	> -4.00	> -4.00
SK-OV-3	-4.50	> -4.00	> -4.00
**Renal Cancer**			
786-0	-5.11	-4.56	-4.07
A498	< -8.00	-6.79	> -4.00
ACHN	-4.99	-4.61	-4.23
CAKI-1	-5.50	-4.59	> -4.00
RXF 393	-5.26	-4.31	> -4.00
SN12C	-4.61	> -4.00	> -4.00
TK-10	-4.58	-4.07	> -4.00
UO-31	-5.50	-4.46	> -4.00
**Prostate Cancer**			
PC-3	-4.83	> -4.00	> -4.00
DU-145	-4.78	-4.35	> -4.00
**Breast Cancer**			
MCF7	-5.16	-4.51	> -4.00
MDA-MB-231/ATCC	-5.45	-4.45	> -4.00
HS 578T	-4.85	-4.25	> -4.00
BT-549	-4.61	> -4.00	> -4.00
T-47D	-4.86	-4.38	> -4.00
MDA-MB-468	-5.14	-4.45	> -4.00
_MID	-4.95	-4.34	-4.05
Delta	3.05	2.45	0.4
Range	3.56	2.79	0.45

GI50

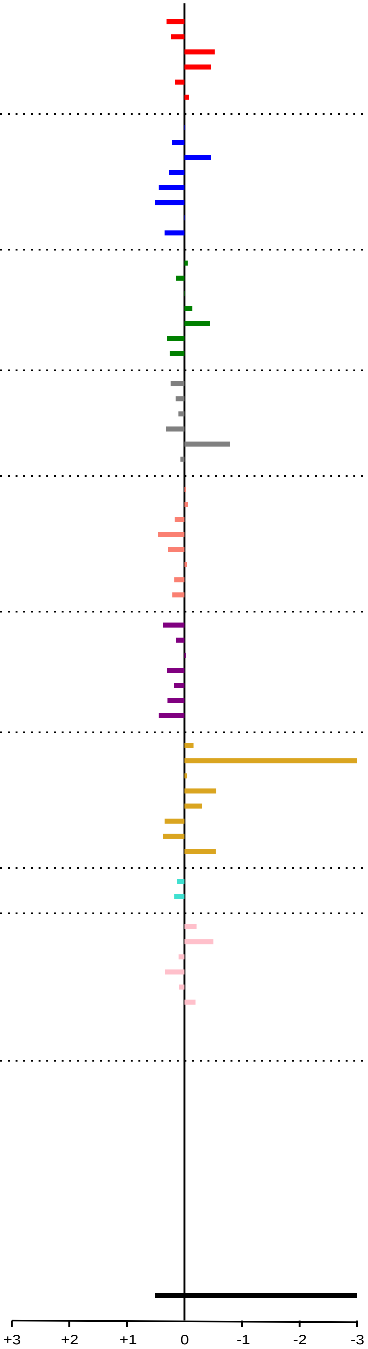

TGI

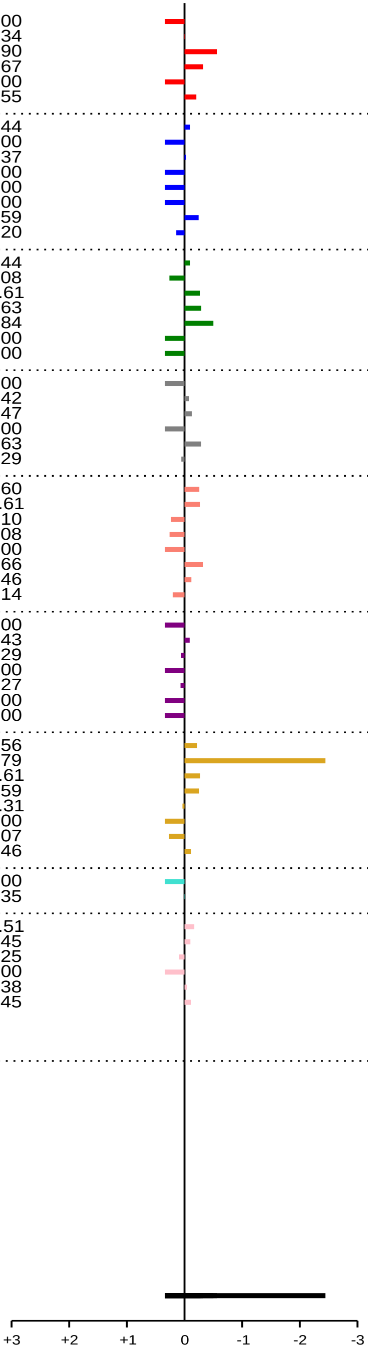

LC50

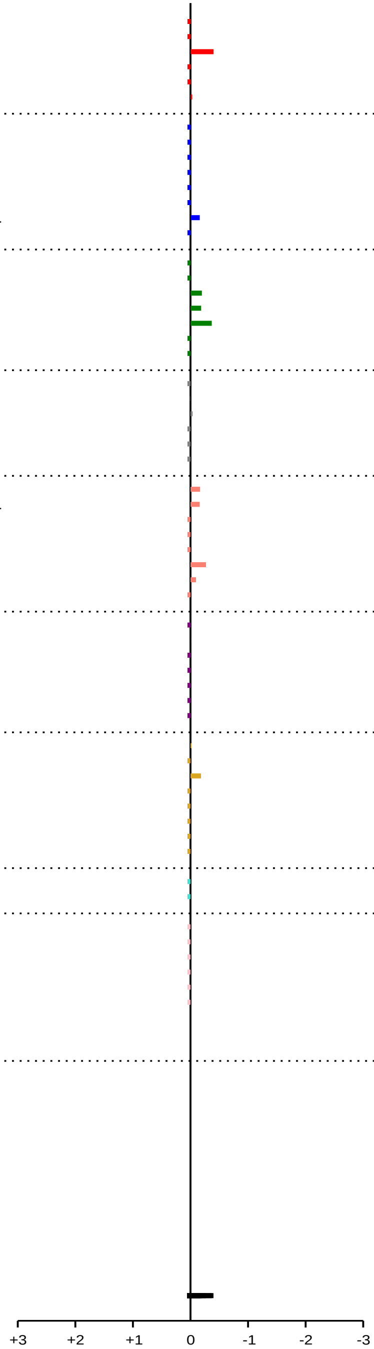

National Cancer Institute Developmental Therapeutics Program Dose Response Curves	NSC:766585/2	SSPL:0YQS	EXP. ID:1310NS81
	Report Date:July 13, 2015	Test Date:October 28, 2013	

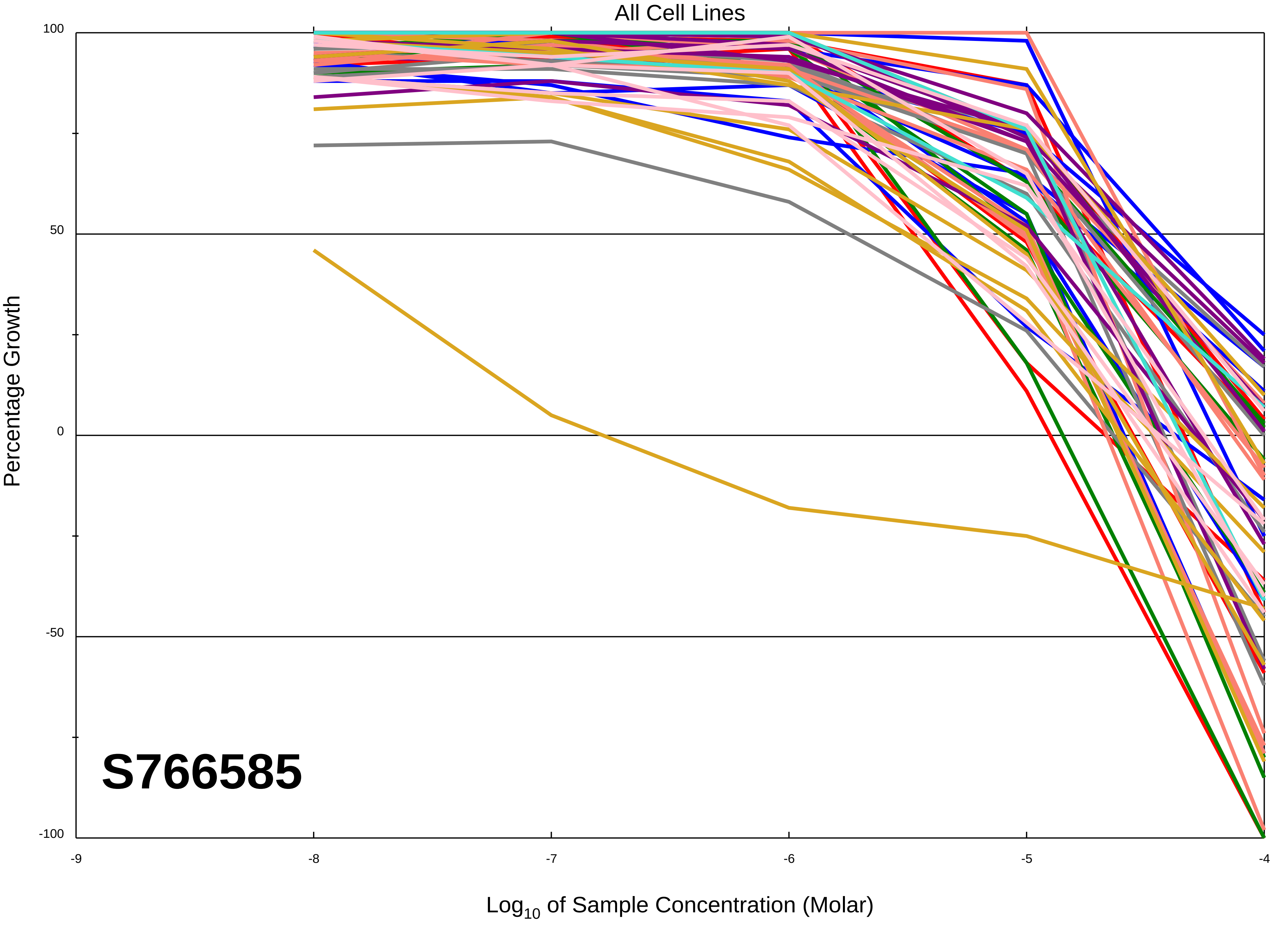

National Cancer Institute Developmental Therapeutics Program Dose Response Curves	NSC: 766585 / 2	SSPL: 0YQS	EXP. ID: 1402RS24
	Report Date: July 13, 2015	Test Date: February 03, 2014	

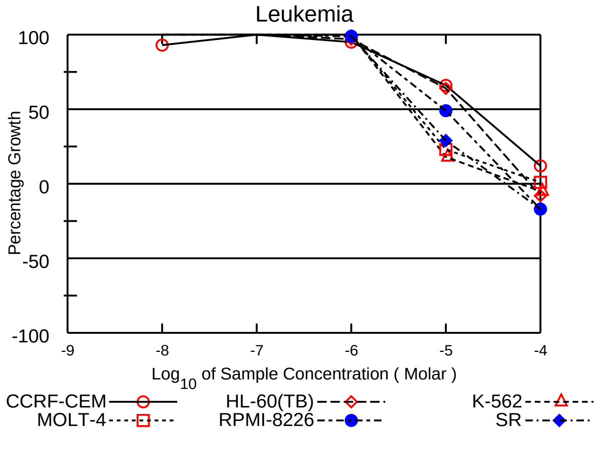

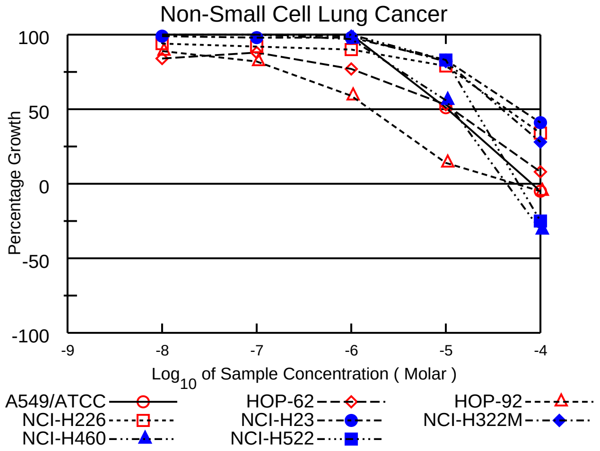

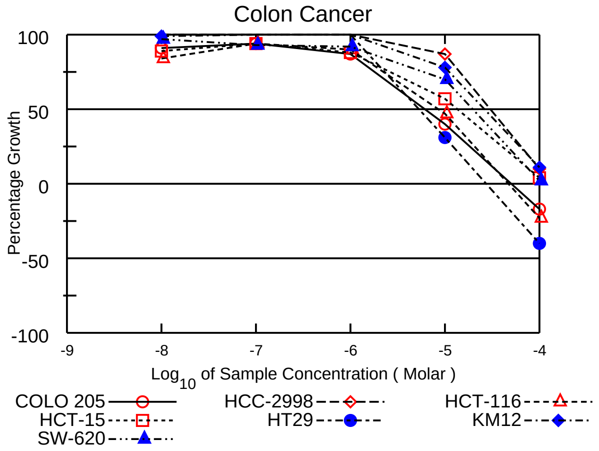

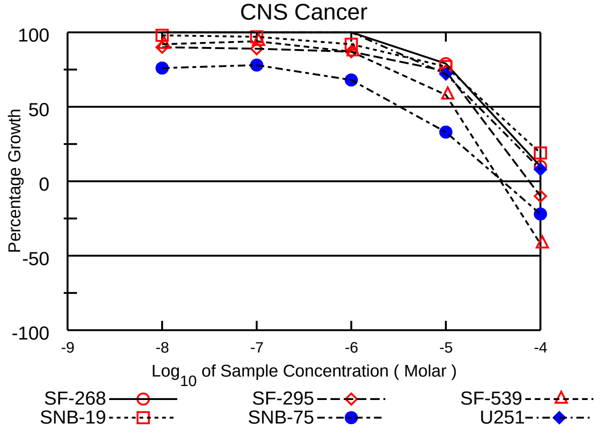

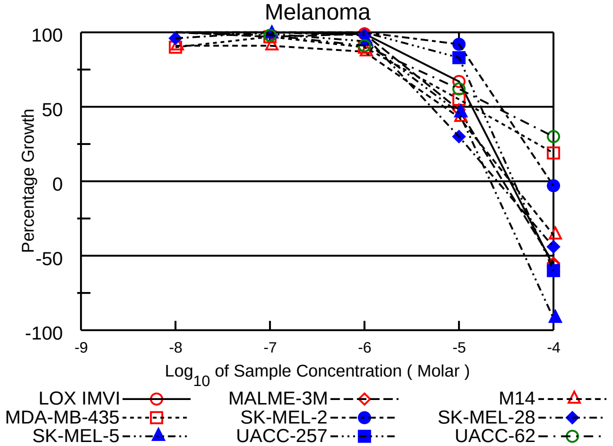

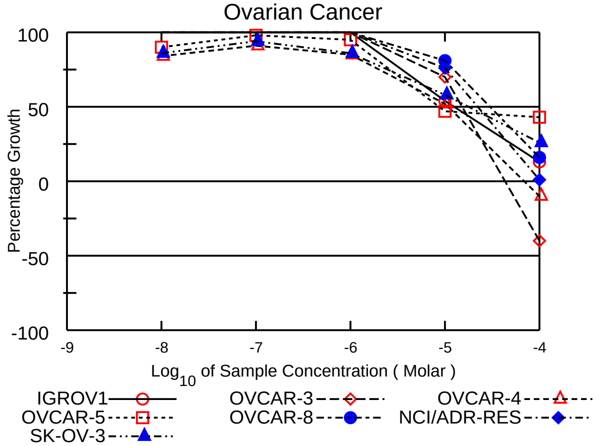

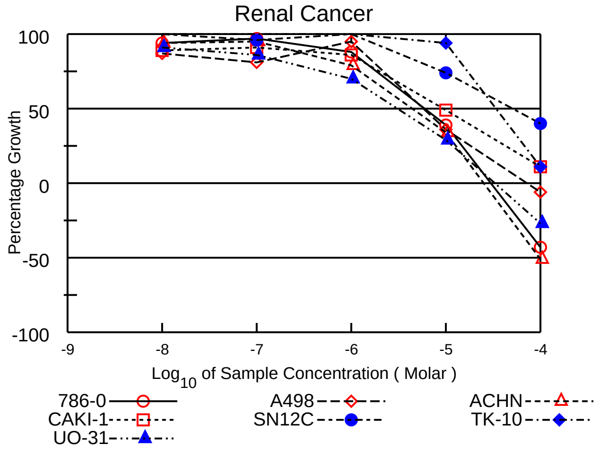

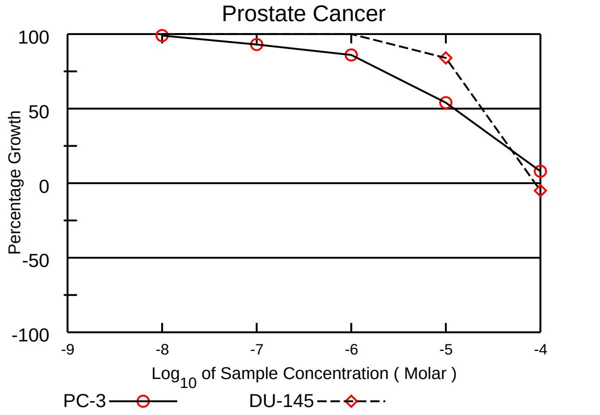

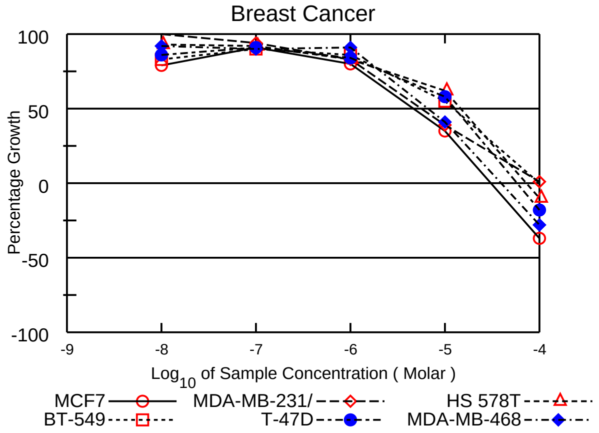

National Cancer Institute Developmental Therapeutics Program Mean Graphs	NSC :766585/2	Units :Molar	SSPL :0YQS	EXP. ID :1402RS24
	Report Date :July 13, 2015		Test Date :February 03, 2014	

Panel/Cell Line	Log_{10}GI50	Log_{10}TGI	Log_{10}LC50
**Leukemia**			
CCRF-CEM	-4.71	> -4.00	> -4.00
HL-60(TB)	-4.80	-4.11	> -4.00
K-562	-5.37	-4.22	> -4.00
MOLT-4	-5.33	> -4.00	> -4.00
RPMI-8226	-5.03	-4.26	> -4.00
SR	-5.30	-4.37	> -4.00
**Non-Small Cell Lung Cancer**			
A549/ATCC	-4.98	-4.09	> -4.00
HOP-62	-4.94	> -4.00	> -4.00
HOP-92	-5.80	-4.28	> -4.00
NCI-H226	-4.36	> -4.00	> -4.00
NCI-H23	-4.22	> -4.00	> -4.00
NCI-H322M	-4.41	> -4.00	> -4.00
NCI-H460	-4.93	-4.36	> -4.00
NCI-H522	-4.70	-4.23	> -4.00
**Colon Cancer**			
COLO 205	-5.21	-4.30	> -4.00
HCC-2998	-4.52	> -4.00	> -4.00
HCT-116	-5.07	-4.33	> -4.00
HCT-15	-4.87	> -4.00	> -4.00
HT29	-5.26	-4.57	> -4.00
KM12	-4.58	> -4.00	> -4.00
SW-620	-4.71	> -4.00	> -4.00
**CNS Cancer**			
SF-268	-4.58	> -4.00	> -4.00
SF-295	-4.72	-4.12	> -4.00
SF-539	-4.92	-4.42	> -4.00
SNB-19	-4.53	> -4.00	> -4.00
SNB-75	-5.48	-4.40	> -4.00
U251	-4.65	> -4.00	> -4.00
**Melanoma**			
LOX IMVI	-4.86	-4.46	-4.05
MALME-3M	-5.04	-4.53	-4.05
M14	-5.15	-4.46	> -4.00
MDA-MB-435	-4.86	> -4.00	> -4.00
SK-MEL-2	-4.56	-4.04	> -4.00
SK-MEL-28	-5.29	-4.59	> -4.00
SK-MEL-5	-5.09	-4.67	-4.30
UACC-257	-4.77	-4.42	-4.07
UACC-62	-4.63	> -4.00	> -4.00
**Ovarian Cancer**			
IGROV1	-4.89	> -4.00	> -4.00
OVCAR-3	-4.82	-4.36	> -4.00
OVCAR-4	-4.96	-4.16	> -4.00
OVCAR-5	-5.06	> -4.00	> -4.00
OVCAR-8	-4.52	> -4.00	> -4.00
NCI/ADR-RES	-4.65	> -4.00	> -4.00
SK-OV-3	-4.76	> -4.00	> -4.00
**Renal Cancer**			
786-0	-5.22	-4.52	> -4.00
A498	-5.24	-4.14	> -4.00
ACHN	-5.35	-4.60	-4.02
CAKI-1	-5.03	> -4.00	> -4.00
SN12C	-4.29	> -4.00	> -4.00
TK-10	-4.47	> -4.00	> -4.00
UO-31	-5.52	-4.48	> -4.00
**Prostate Cancer**			
PC-3	-4.91	> -4.00	> -4.00
DU-145	-4.62	-4.05	> -4.00
**Breast Cancer**			
MCF7	-5.33	-4.51	> -4.00
MDA-MB-231/ATCC	-5.24	> -4.00	> -4.00
HS 578T	-4.83	-4.14	> -4.00
BT-549	-4.91	-4.00	> -4.00
T-47D	-4.90	-4.24	> -4.00
MDA-MB-468	-5.19	-4.41	> -4.00
_MID	-4.91	-4.19	-4.01
Delta	0.89	0.48	0.29
Range	1.58	0.67	0.3

GI50

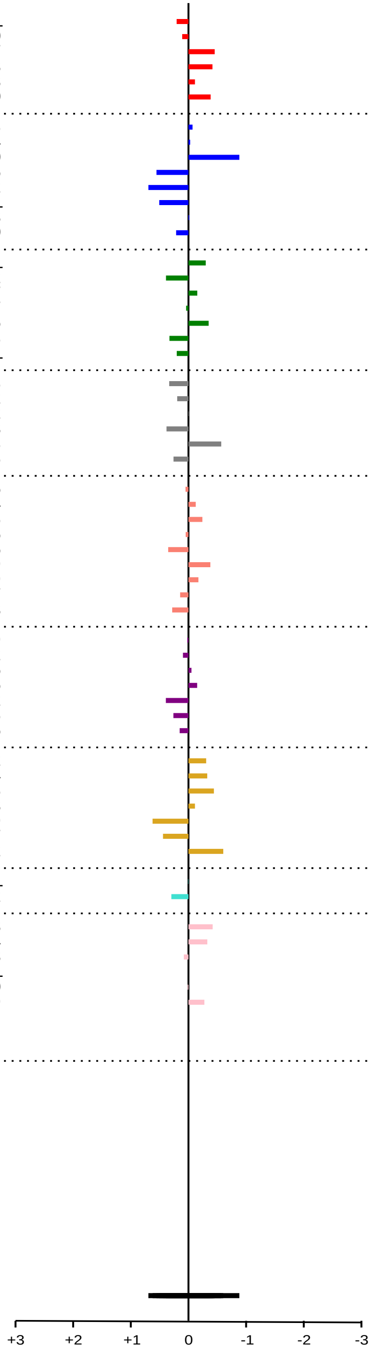

TGI

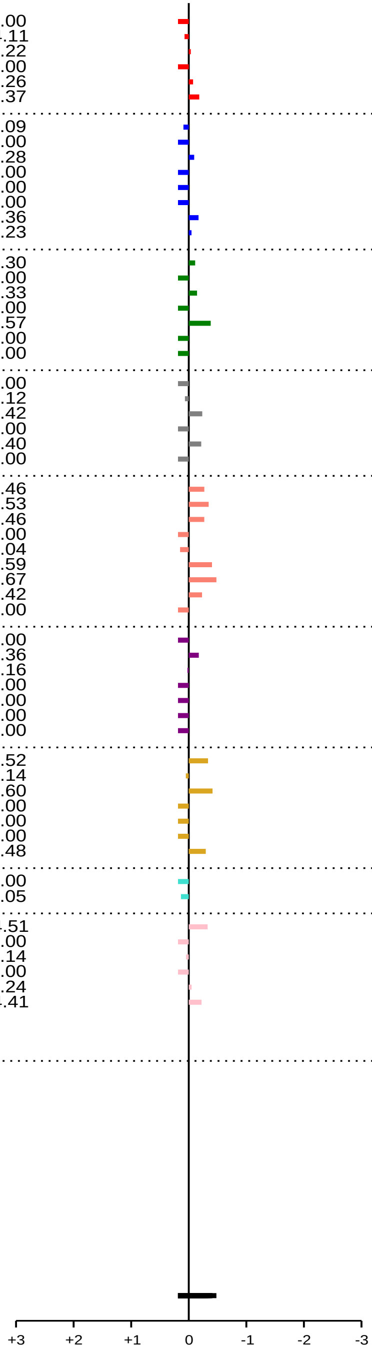

LC50

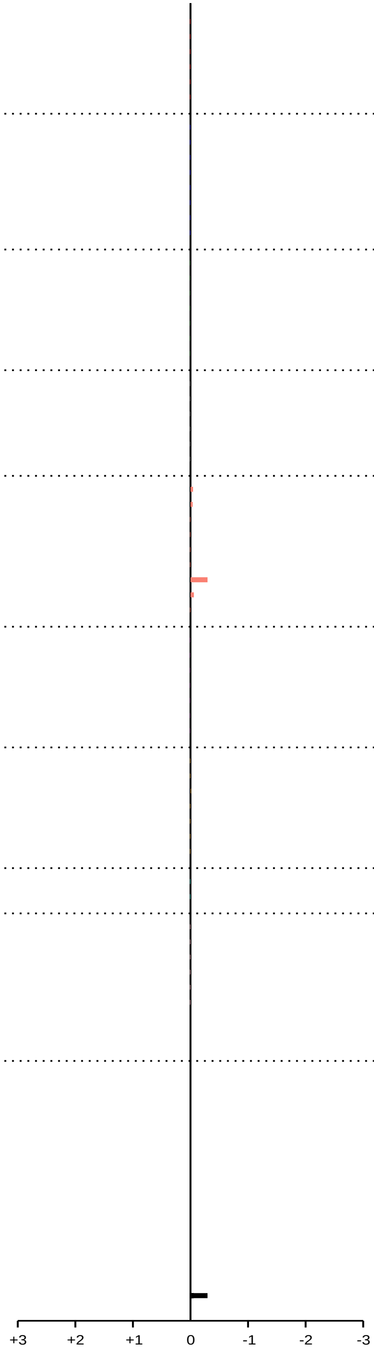

National Cancer Institute Developmental Therapeutics Program Dose Response Curves	NSC:766585/2	SSPL:0YQS	EXP. ID:1402RS24
	Report Date:July 13, 2015	Test Date:February 03, 2014	

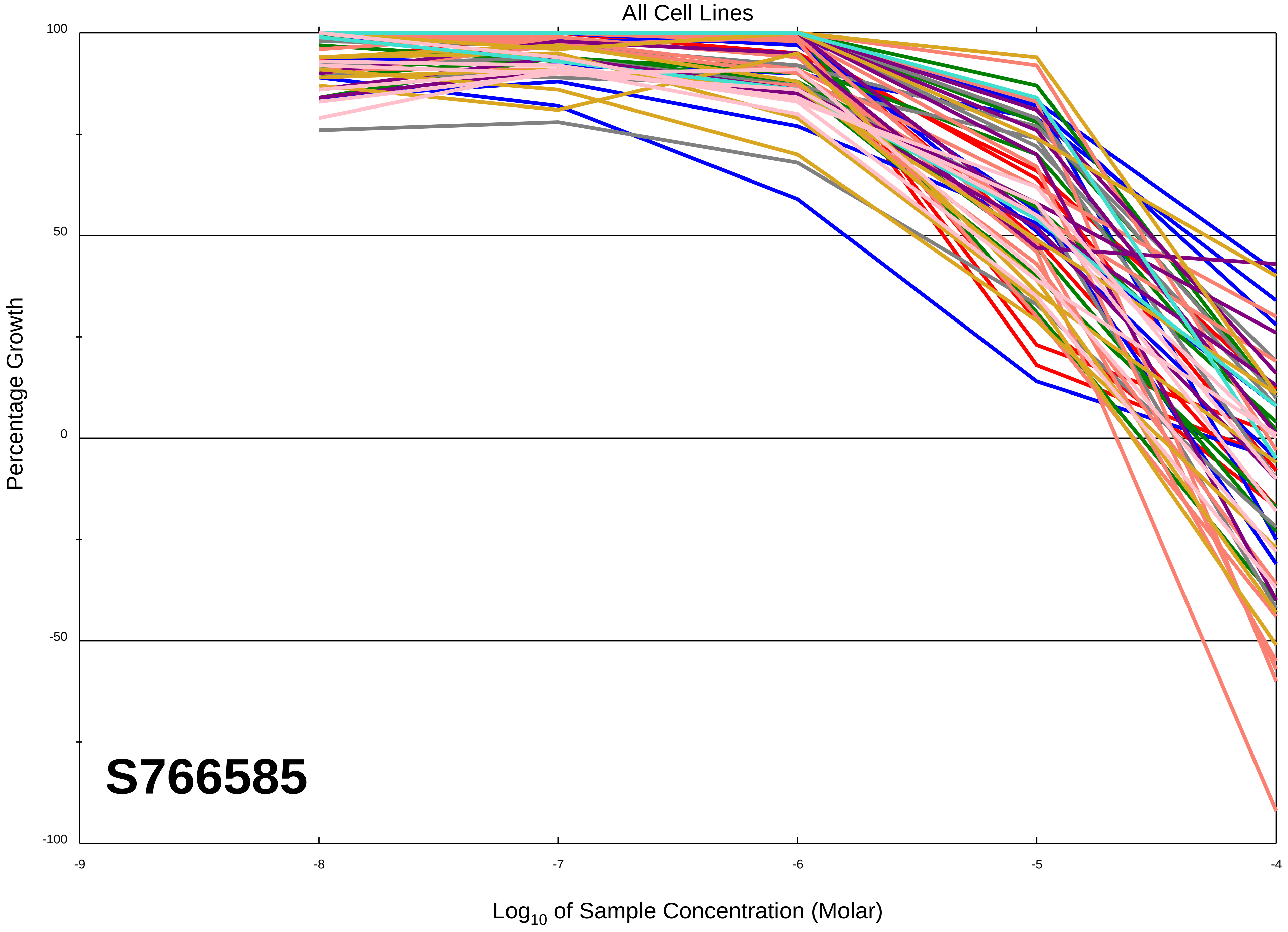

National Cancer Institute Developmental Therapeutics Program Dose Response Curves	NSC: 766634 / 2	SSPL: 0YQS	EXP. ID: 1302NS25
	Report Date: July 13, 2015	Test Date: February 19, 2013	

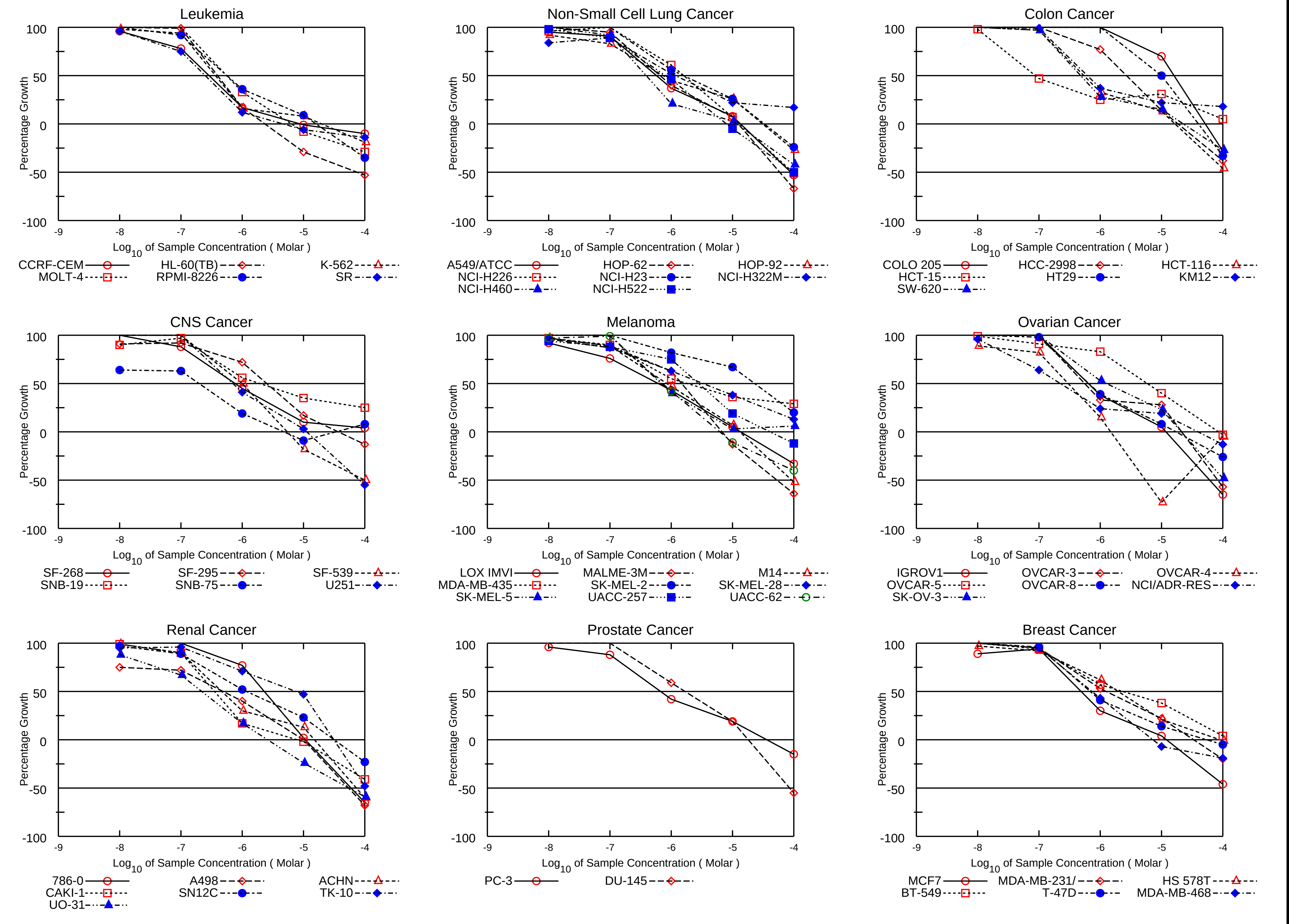

National Cancer Institute Developmental Therapeutics Program Mean Graphs	NSC :766634/2	Units :Molar	SSPL :0YQS	EXP. ID :1302NS25
	Report Date :July 13, 2015		Test Date :February 19, 2013	

Panel/Cell Line	Log_{10}GI50	Log_{10}TGI	Log_{10}LC50
**Leukemia**			
CCRF-CEM	-6.54	-5.07	> -4.00
HL-60(TB)	-6.41	-5.63	-4.11
K-562	-6.44	-4.71	> -4.00
MOLT-4	-6.23	-5.20	> -4.00
RPMI-8226	-6.24	-4.79	> -4.00
SR	-6.60	-5.33	> -4.00
**Non-Small Cell Lung Cancer**			
A549/ATCC	-6.24	-4.87	-4.05
HOP-62	-6.18	-4.91	-4.23
HOP-92	-6.14	-4.51	> -4.00
NCI-H226	-5.79	-4.87	-4.02
NCI-H23	-5.80	-4.48	> -4.00
NCI-H322M	-5.94	> -4.00	> -4.00
NCI-H460	-6.41	-4.93	> -4.00
NCI-H522	-6.10	-5.11	-4.00
**Colon Cancer**			
COLO 205	-4.79	-4.28	> -4.00
HCC-2998	-5.57	-4.73	> -4.00
HCT-116	-6.26	-4.78	> -4.00
HCT-15	-7.06	> -4.00	> -4.00
HT29	-5.00	-4.40	> -4.00
KM12	-6.21	> -4.00	> -4.00
SW-620	-6.32	-4.64	> -4.00
**CNS Cancer**			
SF-268	-6.11	> -4.00	> -4.00
SF-295	-5.60	-4.43	> -4.00
SF-539	-5.99	-5.26	-4.01
SNB-19	-5.71	> -4.00	> -4.00
SNB-75	-6.71		> -4.00
U251	-6.14	-4.95	-4.09
**Melanoma**			
LOX IMVI	-6.20	-4.86	> -4.00
MALME-3M	-5.83	-5.17	-4.27
M14	-6.07	-4.89	-4.03
MDA-MB-435	-5.72	> -4.00	> -4.00
SK-MEL-2	-4.64	> -4.00	> -4.00
SK-MEL-28	-5.48	> -4.00	> -4.00
SK-MEL-5	-6.16	> -4.00	> -4.00
UACC-257	-5.55	-4.40	> -4.00
UACC-62	-6.13	-5.21	> -4.00
**Ovarian Cancer**			
IGROV1	-6.17	-4.93	-4.22
OVCAR-3	-6.24	-4.67	-4.08
OVCAR-4	-6.52	-5.83	
OVCAR-5	-5.22	-4.06	> -4.00
OVCAR-8	-6.18	-4.77	> -4.00
NCI/ADR-RES	-6.65	-4.41	> -4.00
SK-OV-3	-5.91	-4.68	> -4.00
**Renal Cancer**			
786-0	-5.64	-4.97	-4.23
A498	-6.31	-5.01	-4.26
ACHN	-6.33	-4.82	-4.14
CAKI-1	-6.45	-5.11	> -4.00
SN12C	-5.92	-4.50	> -4.00
TK-10	-5.12	-4.50	> -4.00
UO-31	-6.66	-5.59	-4.25
**Prostate Cancer**			
PC-3	-6.18	-4.44	> -4.00
DU-145	-5.78	-4.74	-4.07
**Breast Cancer**			
MCF7	-6.32	-4.93	> -4.00
MDA-MB-231/ATCC	-5.90	-4.47	> -4.00
HS 578T	-5.71	-4.03	> -4.00
BT-549	-5.64	> -4.00	> -4.00
T-47D	-6.16	-4.25	> -4.00
MDA-MB-468	-6.14	-5.14	> -4.00
_MID	-6.03	-4.67	-4.04
Delta	1.03	1.16	0.23
Range	2.42	1.83	0.27

Axis scale (GI50, TGI, LC50): +3 +2 +1 0 -1 -2 -3

National Cancer Institute Developmental Therapeutics Program Dose Response Curves	NSC:766634/2	SSPL:0YQS	EXP. ID:1302NS25
	Report Date:July 13, 2015	Test Date:February 19, 2013	

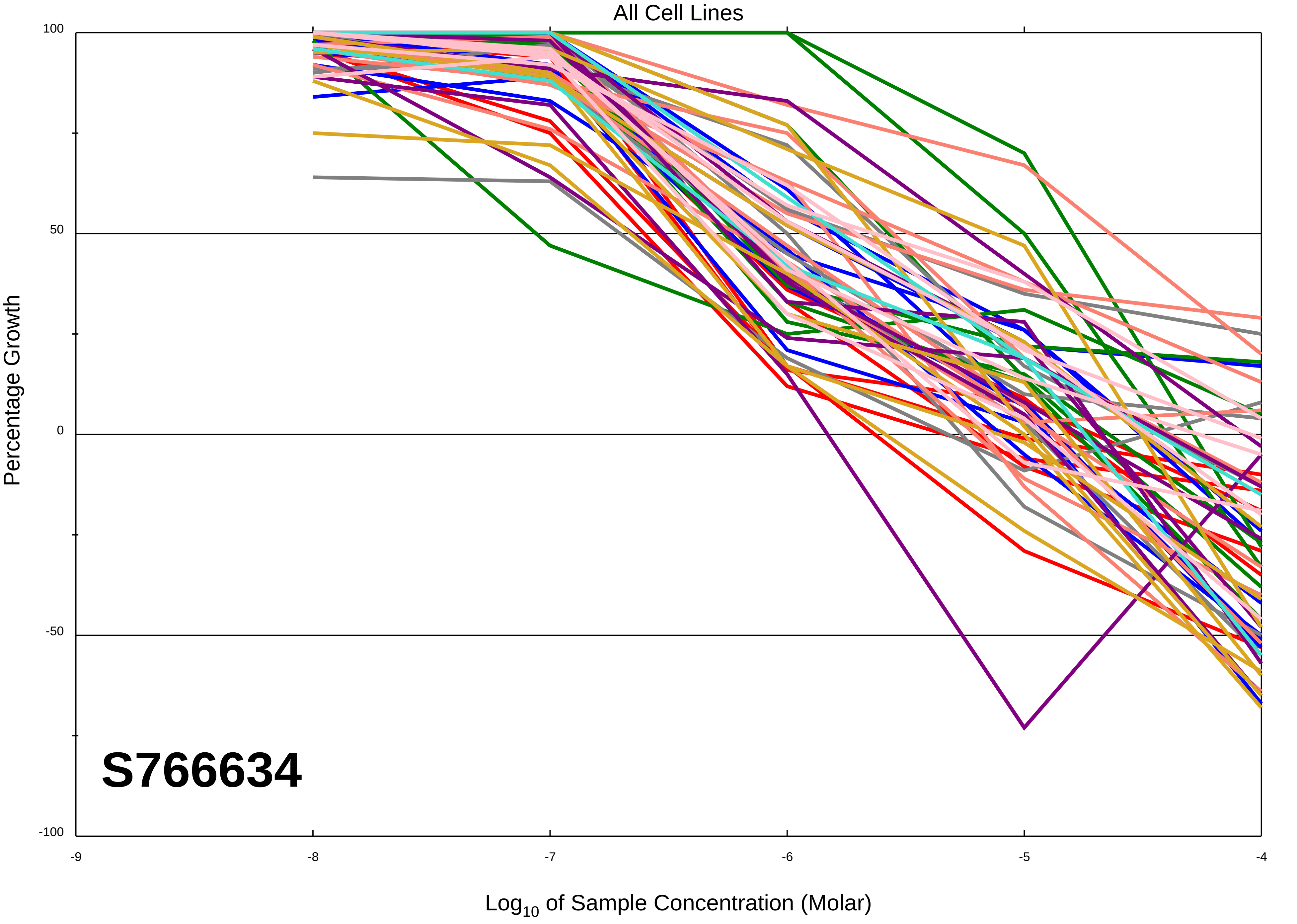

National Cancer Institute Developmental Therapeutics Program Dose Response Curves	NSC: 766634 / 2	SSPL: 0YQS	EXP. ID: 1306RS02
	Report Date: July 13, 2015	Test Date: June 03, 2013	

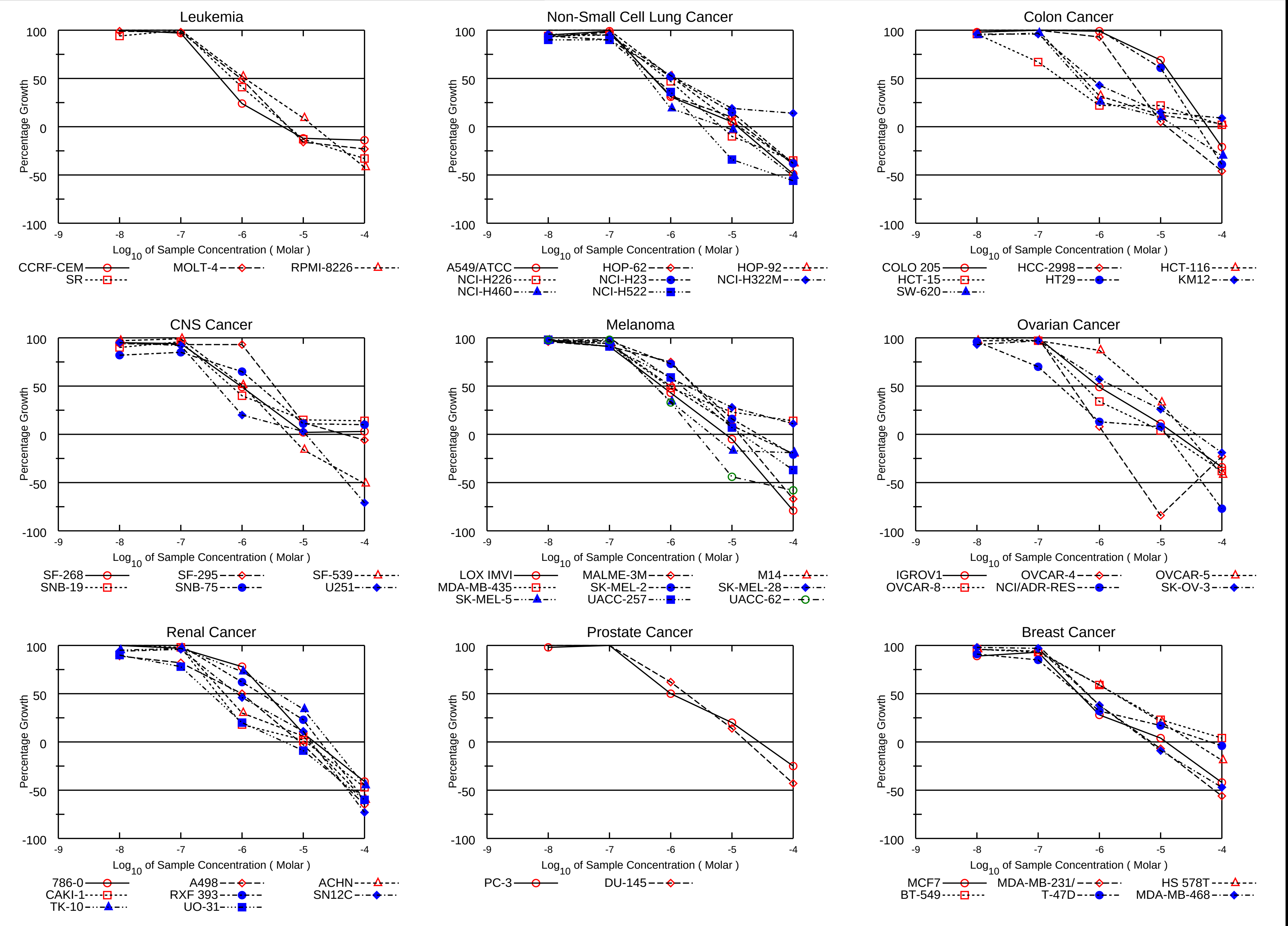

National Cancer Institute Developmental Therapeutics Program Mean Graphs	NSC :766634/2	Units :Molar	SSPL :0YQS	EXP. ID :1306RS02
	Report Date :July 13, 2015		Test Date :June 03, 2013	

Panel/Cell Line	Log_{10}GI50	Log_{10}TGI	Log_{10}LC50
**Leukemia**			
CCRF-CEM	-6.36	-5.33	> -4.00
MOLT-4	-6.02	-5.25	> -4.00
RPMI-8226	-5.96	-4.82	> -4.00
SR	-6.15	-5.24	> -4.00
**Non-Small Cell Lung Cancer**			
A549/ATCC	-6.28	-4.91	> -4.00
HOP-62	-6.27	-4.79	> -4.00
HOP-92	-5.95	-4.89	> -4.00
NCI-H226	-6.07	-5.17	> -4.00
NCI-H23	-5.95	-4.72	> -4.00
NCI-H322M	-5.90	> -4.00	> -4.00
NCI-H460	-6.39	-5.14	-4.03
NCI-H522	-6.26	-5.49	-4.28
**Colon Cancer**			
COLO 205	-4.79	-4.24	> -4.00
HCC-2998	-5.52	-4.91	> -4.00
HCT-116	-6.27	> -4.00	> -4.00
HCT-15	-6.63	> -4.00	> -4.00
HT29	-4.89	-4.39	> -4.00
KM12	-6.14	> -4.00	> -4.00
SW-620	-6.34	-4.75	> -4.00
**CNS Cancer**			
SF-268	-6.03	> -4.00	> -4.00
SF-295	-5.47	-4.32	> -4.00
SF-539	-5.99	-5.24	-4.02
SNB-19	-6.18	> -4.00	> -4.00
SNB-75	-5.72	> -4.00	> -4.00
U251	-6.42	-4.96	-4.28
**Melanoma**			
LOX IMVI	-6.14	-5.11	-4.39
MALME-3M	-5.62	-4.88	-4.22
M14	-6.00	-4.68	> -4.00
MDA-MB-435	-6.04	> -4.00	> -4.00
SK-MEL-2	-5.60	-4.58	> -4.00
SK-MEL-28	-5.72	> -4.00	> -4.00
SK-MEL-5	-6.26	-5.34	> -4.00
UACC-257	-5.82	-4.84	> -4.00
UACC-62	-6.27	-5.57	-4.56
**Ovarian Cancer**			
IGROV1	-6.01	-4.75	> -4.00
OVCAR-4	-6.45	-5.91	
OVCAR-5	-5.32	-4.56	> -4.00
OVCAR-8	-6.25	-4.91	> -4.00
NCI/ADR-RES	-6.65	-4.90	-4.31
SK-OV-3	-5.77	-4.43	> -4.00
**Renal Cancer**			
786-0	-5.60	-4.83	> -4.00
A498	-6.01	-5.06	-4.24
ACHN	-6.28	-4.90	-4.15
CAKI-1	-6.40	-4.96	> -4.00
RXF 393	-5.69	-4.72	-4.12
SN12C	-6.09	-4.87	-4.27
TK-10	-5.41	-4.57	> -4.00
UO-31	-6.51	-5.30	-4.20
**Prostate Cancer**			
PC-3	-6.01	-4.55	> -4.00
DU-145	-5.75	-4.76	> -4.00
**Breast Cancer**			
MCF7	-6.33	-4.90	> -4.00
MDA-MB-231/ATCC	-6.19	-5.16	-4.13
HS 578T	-5.77	-4.48	> -4.00
BT-549	-5.75	> -4.00	> -4.00
T-47D	-6.34	-4.19	> -4.00
MDA-MB-468	-6.20	-5.19	> -4.00
_MID	-6.0	-4.74	-4.06
Delta	0.65	1.17	0.5
Range	1.86	1.91	0.56

GI50

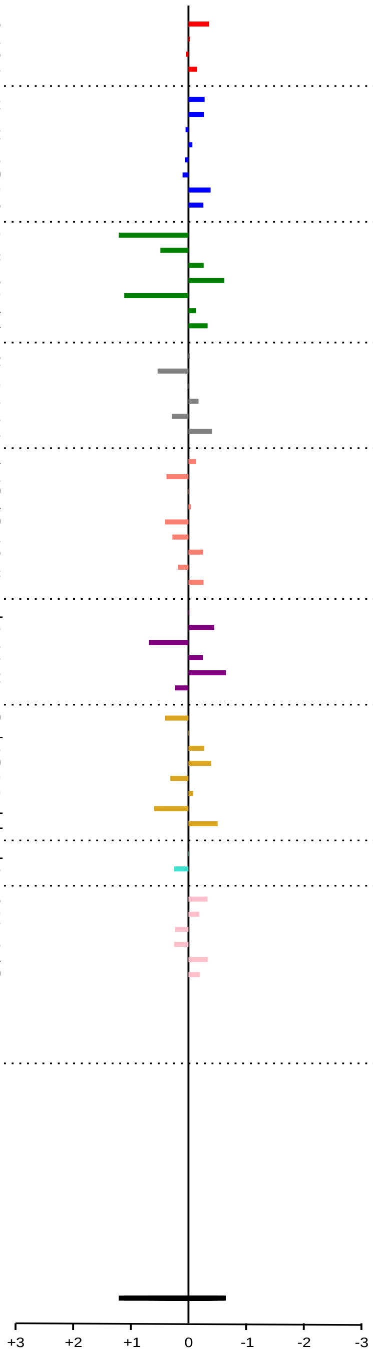

TGI

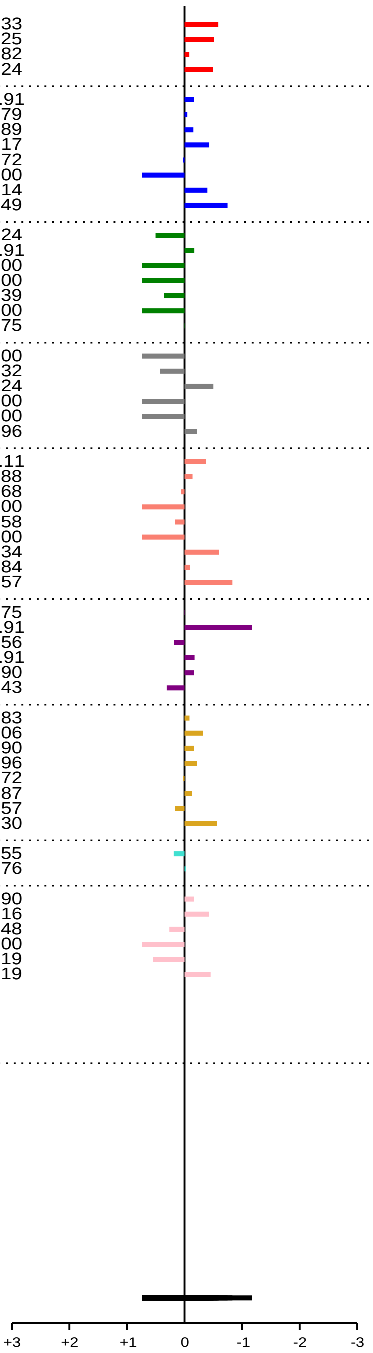

LC50

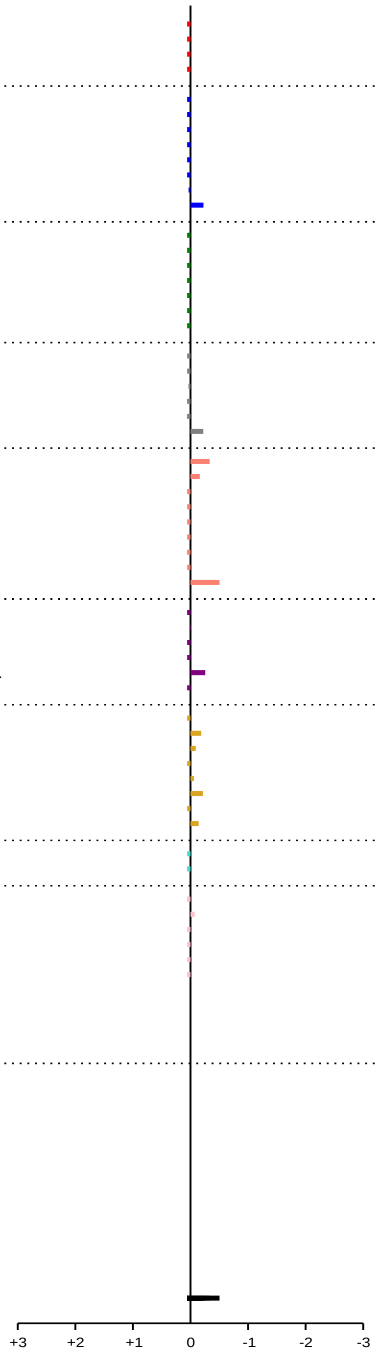

National Cancer Institute Developmental Therapeutics Program Dose Response Curves	NSC:766634/2	SSPL:0YQS	EXP. ID:1306RS02
	Report Date:July 13, 2015	Test Date:June 03, 2013	

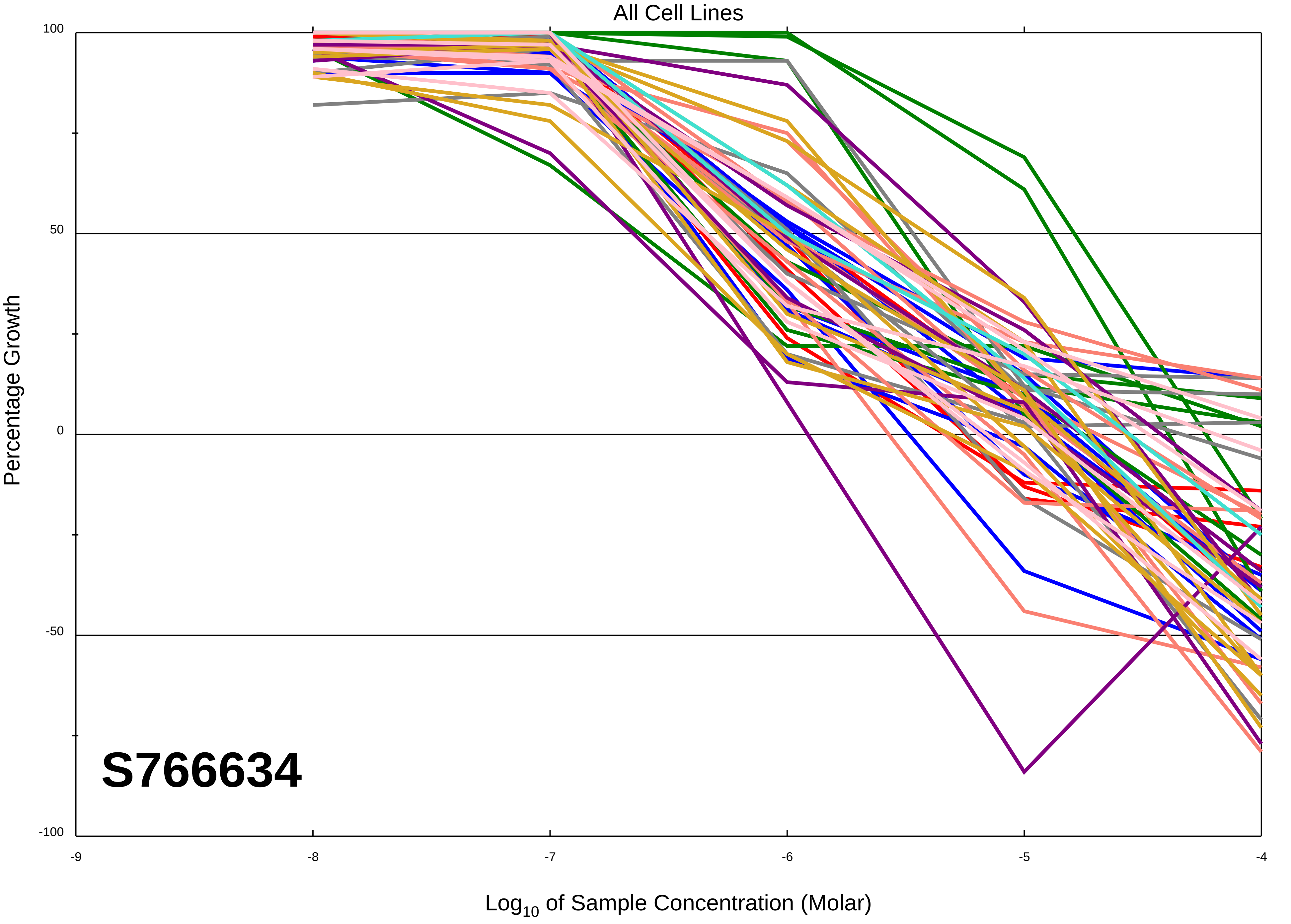

National Cancer Institute Developmental Therapeutics Program Dose Response Curves	NSC: 766672 / 2	SSPL: 0YQS	EXP. ID: 1302NS28
	Report Date: July 13, 2015	Test Date: February 25, 2013	

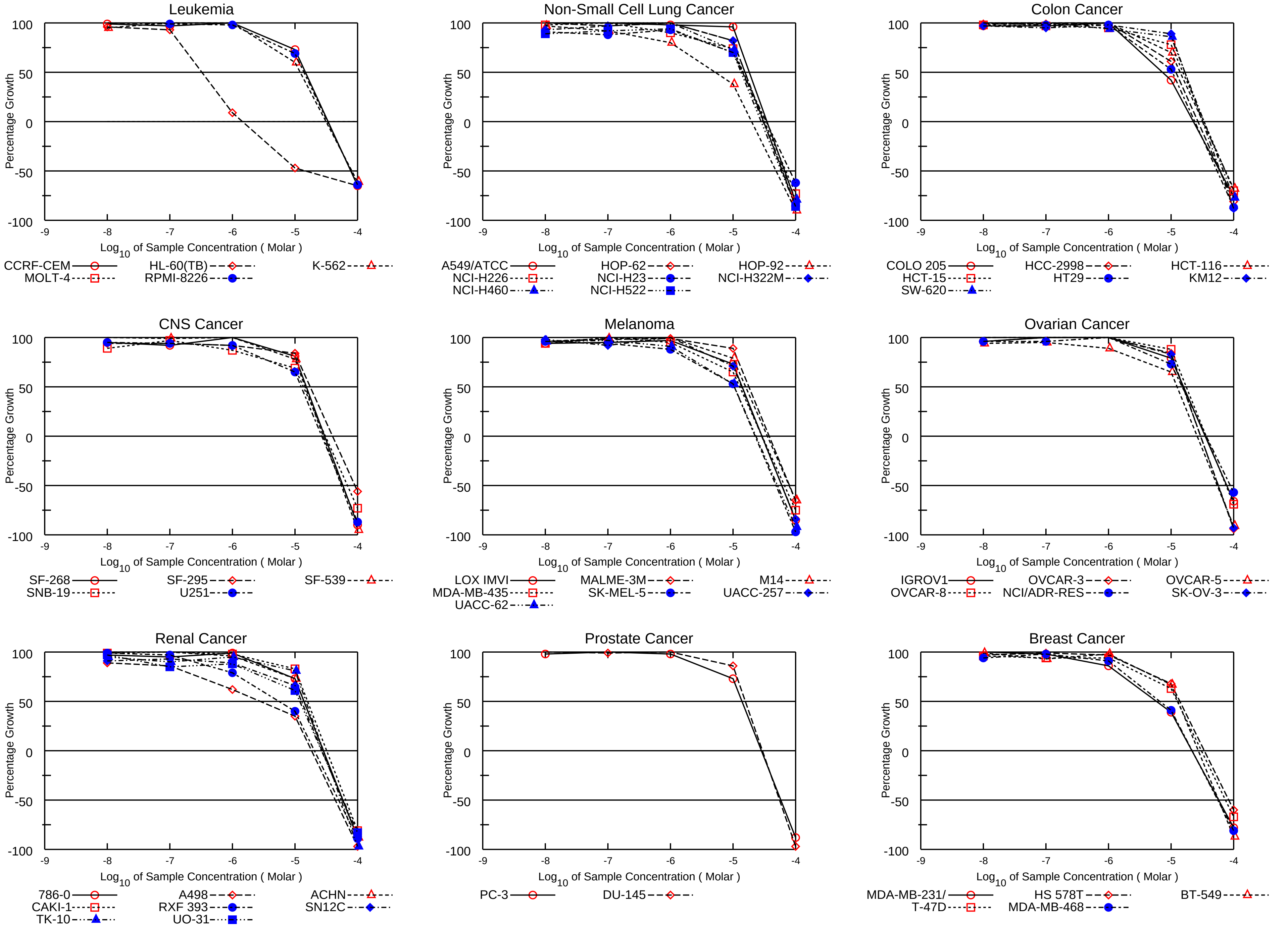

National Cancer Institute Developmental Therapeutics Program Mean Graphs	NSC :766672/2	Units :Molar	SSPL :0YQS	EXP. ID :1302NS28
	Report Date :July 13, 2015		Test Date :February 25, 2013	

Panel/Cell Line	Log_{10}GI50	Log_{10}TGI	Log_{10}LC50
**Leukemia**			
CCRF-CEM	-4.83	-4.47	-4.11
HL-60(TB)	-6.49	-5.84	-4.81
K-562	-4.91	-4.50	-4.09
MOLT-4			
RPMI-8226	-4.86	-4.48	-4.10
**Non-Small Cell Lung Cancer**			
A549/ATCC	-4.74	-4.46	-4.17
HOP-62	-4.81	-4.51	-4.22
HOP-92	-5.29	-4.70	-4.31
NCI-H226	-4.84	-4.50	-4.16
NCI-H23	-4.85	-4.47	-4.09
NCI-H322M	-4.81	-4.51	-4.21
NCI-H460	-4.85	-4.52	-4.19
NCI-H522	-4.87	-4.55	-4.23
**Colon Cancer**			
COLO 205	-5.14	-4.64	-4.22
HCC-2998	-4.92	-4.57	-4.22
HCT-116	-4.86	-4.49	-4.13
HCT-15	-4.81	-4.47	-4.14
HT29	-4.98	-4.62	-4.26
KM12	-4.78	-4.50	-4.22
SW-620	-4.78	-4.47	-4.17
**CNS Cancer**			
SF-268	-4.82	-4.53	-4.23
SF-295	-4.76	-4.40	-4.04
SF-539	-4.84	-4.55	-4.26
SNB-19	-4.87	-4.52	-4.17
U251	-4.90	-4.57	-4.24
**Melanoma**			
LOX IMVI	-4.86	-4.54	-4.22
MALME-3M	-4.75	-4.42	-4.10
M14	-4.80	-4.45	-4.10
MDA-MB-435	-4.89	-4.53	-4.18
SK-MEL-5	-4.98	-4.65	-4.31
UACC-257	-4.87	-4.54	-4.22
UACC-62	-4.98	-4.63	-4.29
**Ovarian Cancer**			
IGROV1	-4.80	-4.45	-4.11
OVCAR-3	-4.81	-4.53	-4.25
OVCAR-5	-4.90	-4.58	-4.26
OVCAR-8	-4.76	-4.44	-4.12
NCI/ADR-RES	-4.82	-4.44	-4.06
SK-OV-3	-4.81	-4.53	-4.25
**Renal Cancer**			
786-0	-4.86	-4.54	-4.23
A498	-5.56	-4.73	-4.35
ACHN	-4.86	-4.55	-4.23
CAKI-1	-4.80	-4.49	-4.19
RXF 393	-5.26	-4.69	-4.30
SN12C	-4.90	-4.57	-4.24
TK-10	-4.83	-4.54	-4.26
UO-31	-4.93	-4.58	-4.23
**Prostate Cancer**			
PC-3	-4.86	-4.55	-4.24
DU-145	-4.80	-4.53	-4.26
**Breast Cancer**			
MDA-MB-231/ATCC	-5.23	-4.67	-4.24
HS 578T	-4.86	-4.47	-4.07
BT-549	-4.89	-4.56	-4.24
T-47D	-4.90	-4.52	-4.13
MDA-MB-468	-5.18	-4.66	-4.25
_MID	-4.93	-4.56	-4.21
Delta	1.56	1.28	0.6
Range	1.75	1.44	0.77

GI50

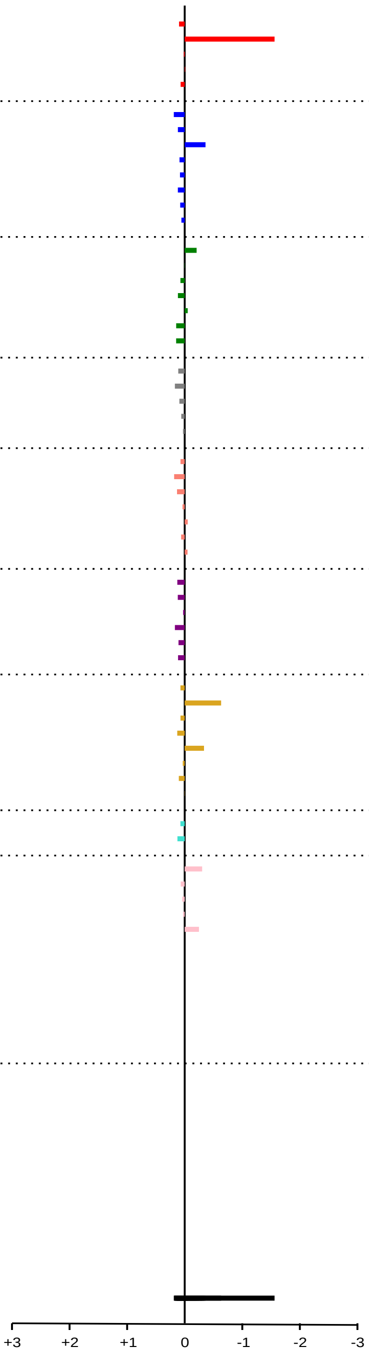

TGI

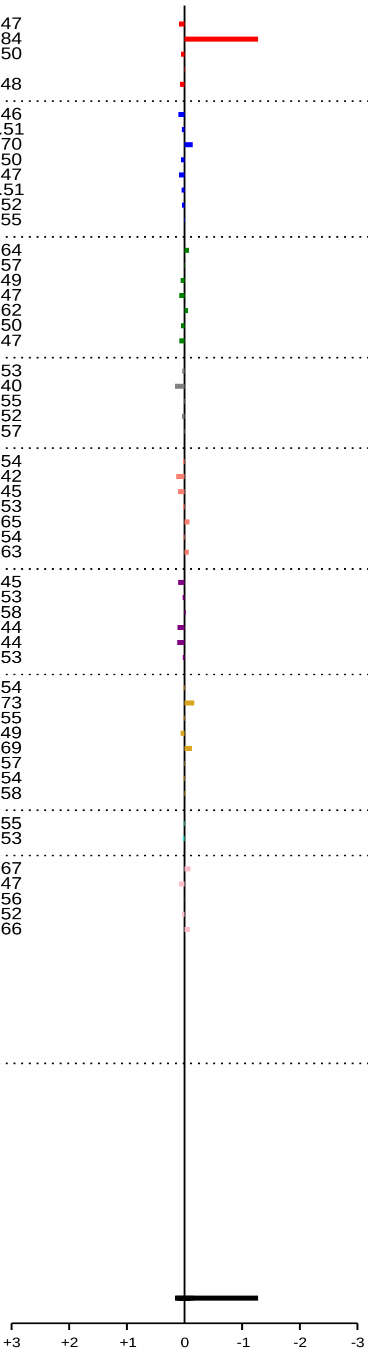

LC50

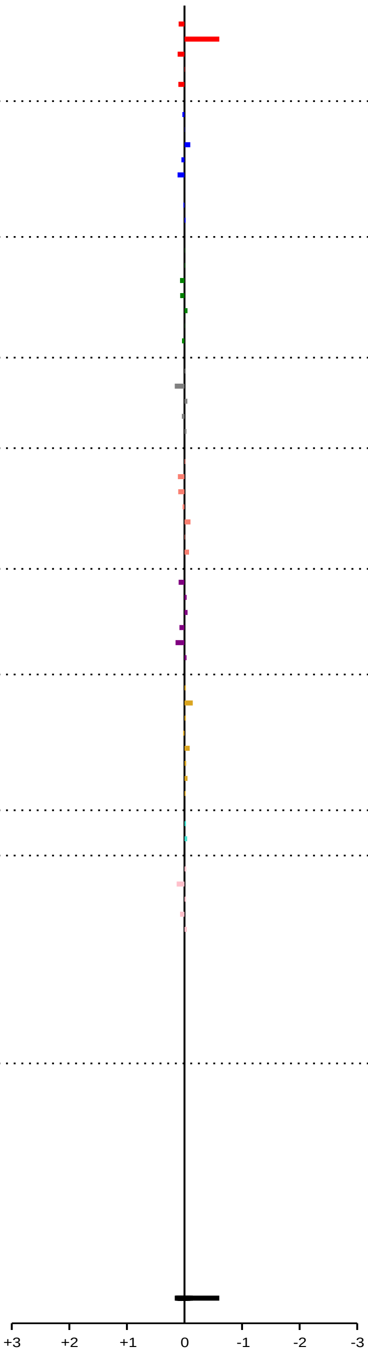

**National Cancer Institute Developmental Therapeutics Program Dose Response Curves**	NSC:766672/2	SSPL:0YQS	EXP. ID:1302NS28
	Report Date:July 13, 2015	Test Date:February 25, 2013	

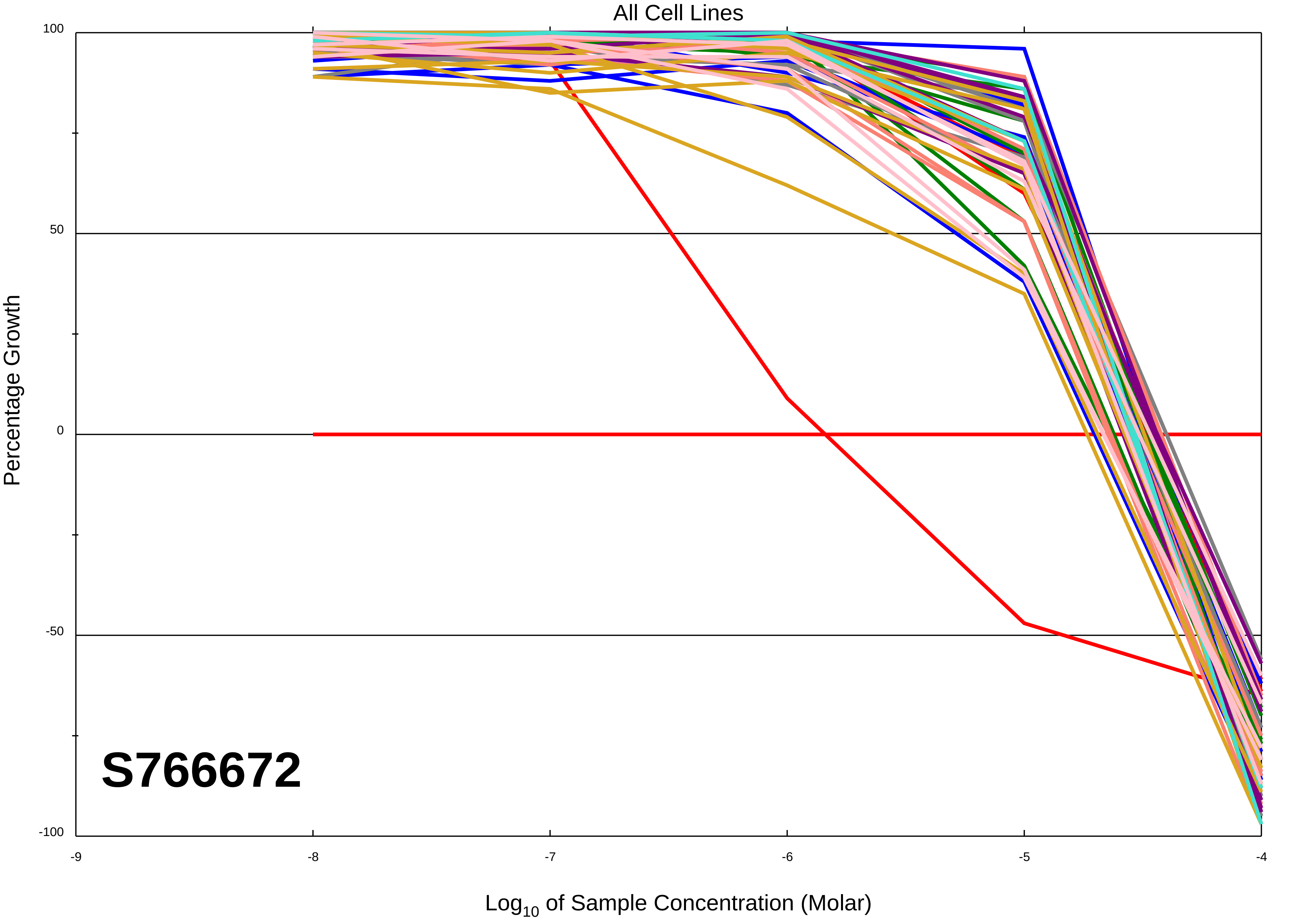

National Cancer Institute Developmental Therapeutics Program Dose Response Curves	NSC: 766672 / 2	SSPL: 0YQS	EXP. ID: 1306RS02
	Report Date: July 13, 2015	Test Date: June 03, 2013	

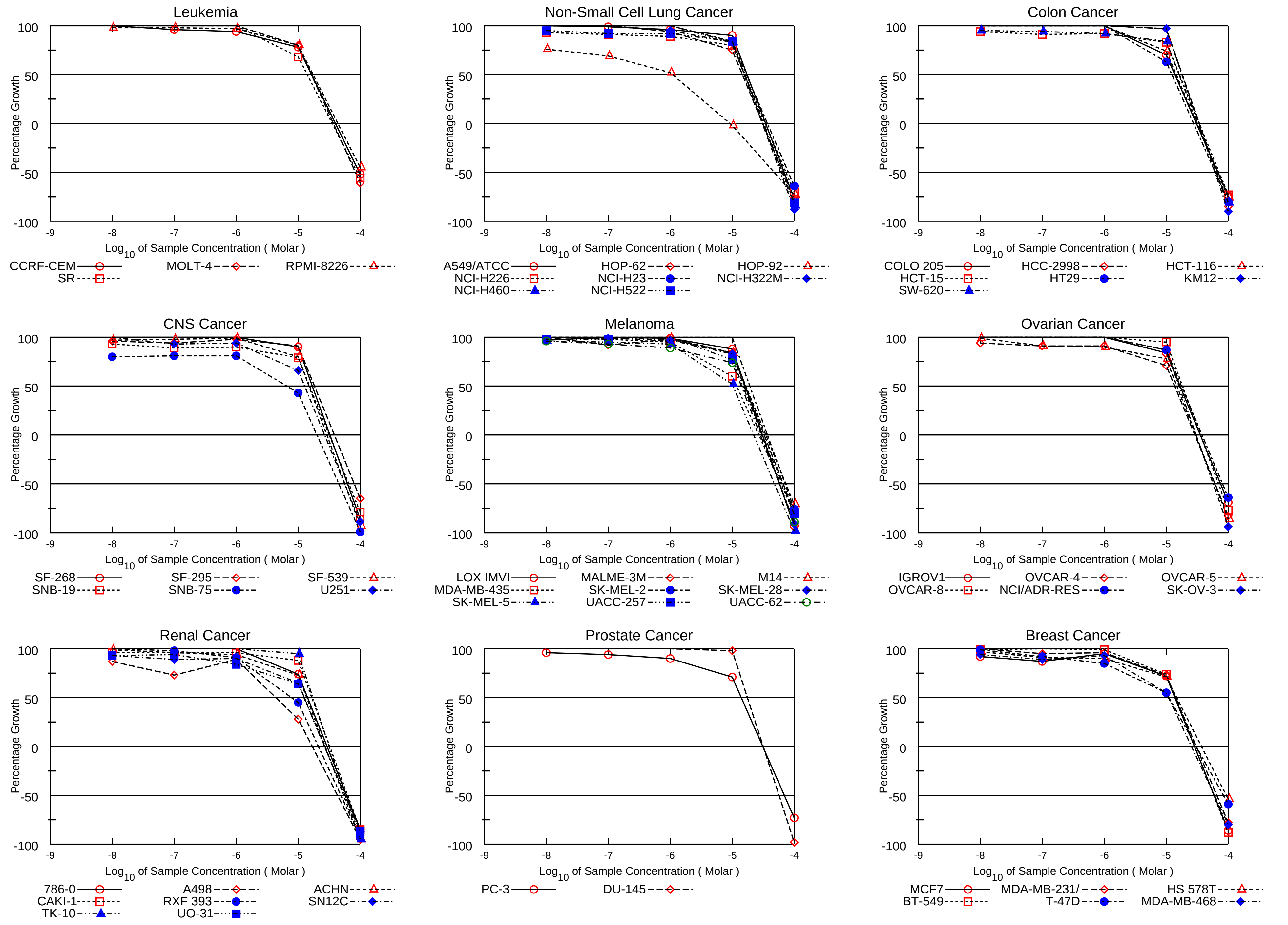

National Cancer Institute Developmental Therapeutics Program Mean Graphs	NSC :766672/2	Units :Molar	SSPL :0YQS	EXP. ID :1306RS02
	Report Date :July 13, 2015		Test Date :June 03, 2013	

Panel/Cell Line	Log_{10}GI50	Log_{10}TGI	Log_{10}LC50
**Leukemia**			
CCRF-CEM	-4.78	-4.40	-4.02
MOLT-4	-4.79	-4.43	-4.08
RPMI-8226	-4.76	-4.36	> -4.00
SR	-4.85	-4.45	-4.05
**Non-Small Cell Lung Cancer**			
A549/ATCC	-4.76	-4.46	-4.16
HOP-62	-4.84	-4.51	-4.18
HOP-92	-5.96	-5.04	-4.32
NCI-H226	-4.80	-4.47	-4.13
NCI-H23	-4.77	-4.43	-4.09
NCI-H322M	-4.81	-4.51	-4.22
NCI-H460	-4.80	-4.50	-4.20
NCI-H522	-4.79	-4.49	-4.19
**Colon Cancer**			
COLO 205	-4.86	-4.52	-4.17
HCC-2998	-4.74	-4.47	-4.19
HCT-116	-4.84	-4.51	-4.17
HCT-15	-4.79	-4.47	-4.15
HT29	-4.91	-4.56	-4.21
KM12	-4.75	-4.48	-4.21
SW-620	-4.79	-4.49	-4.19
**CNS Cancer**			
SF-268	-4.77	-4.49	-4.20
SF-295	-4.74	-4.42	-4.10
SF-539	-4.83	-4.54	-4.25
SNB-19	-4.82	-4.50	-4.18
SNB-75	-5.19	-4.70	-4.34
U251	-4.90	-4.57	-4.25
**Melanoma**			
LOX IMVI	-4.79	-4.51	-4.24
MALME-3M	-4.79	-4.48	-4.18
M14	-4.78	-4.46	-4.13
MDA-MB-435	-4.93	-4.57	-4.21
SK-MEL-2	-4.70	-4.42	-4.15
SK-MEL-28	-4.81	-4.52	-4.24
SK-MEL-5	-4.98	-4.65	-4.32
UACC-257	-4.83	-4.51	-4.20
UACC-62	-4.85	-4.54	-4.24
**Ovarian Cancer**			
IGROV1	-4.78	-4.45	-4.12
OVCAR-4	-4.87	-4.54	-4.21
OVCAR-5	-4.83	-4.52	-4.22
OVCAR-8	-4.74	-4.45	-4.16
NCI/ADR-RES	-4.75	-4.42	-4.09
SK-OV-3	-4.80	-4.52	-4.24
**Renal Cancer**			
786-0	-4.85	-4.54	-4.22
A498	-5.36	-4.77	-4.36
ACHN	-4.86	-4.57	-4.27
CAKI-1	-4.78	-4.49	-4.20
RXF 393	-5.11	-4.67	-4.31
SN12C	-4.90	-4.57	-4.24
TK-10	-4.76	-4.50	-4.24
UO-31	-4.91	-4.58	-4.25
**Prostate Cancer**			
PC-3	-4.85	-4.51	-4.16
DU-145	-4.75	-4.50	-4.25
**Breast Cancer**			
MCF7	-4.86	-4.54	-4.23
MDA-MB-231/ATCC	-4.85	-4.52	-4.19
HS 578T	-4.83	-4.43	-4.03
BT-549	-4.85	-4.54	-4.24
T-47D	-4.96	-4.52	-4.08
MDA-MB-468	-4.97	-4.59	-4.22
_MID	-4.86	-4.52	-4.19
Delta	1.1	0.52	0.17
Range	1.26	0.68	0.36

GI50

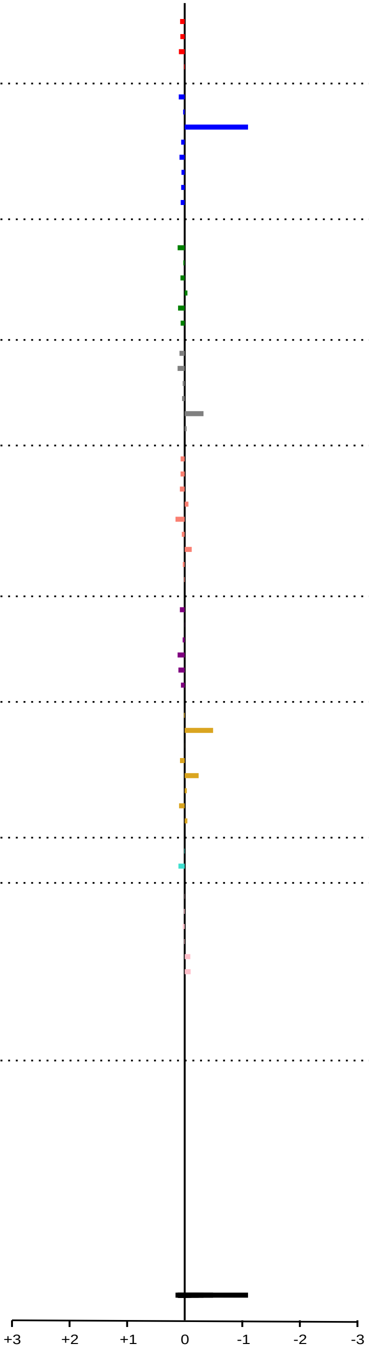

TGI

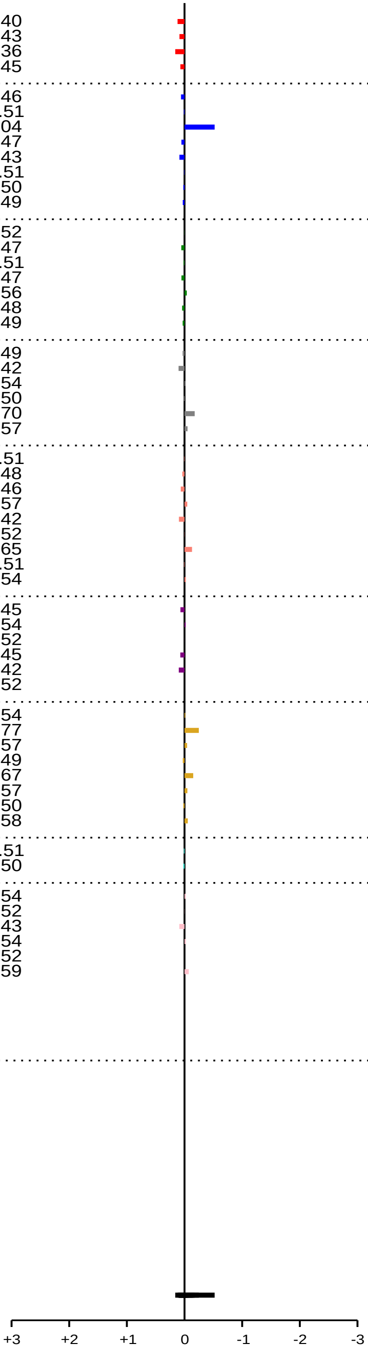

LC50

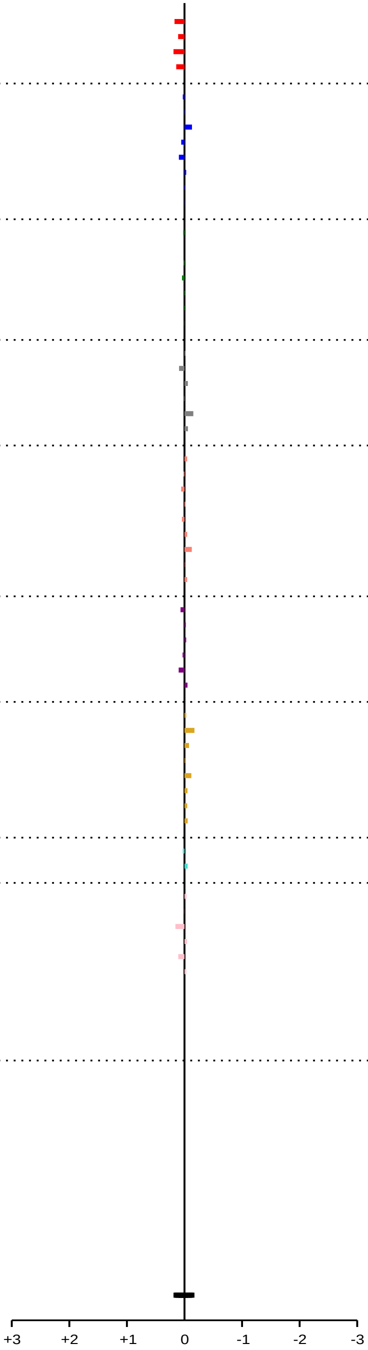

National Cancer Institute Developmental Therapeutics Program Dose Response Curves	NSC:766672/2	SSPL:0YQS	EXP. ID:1306RS02
	Report Date:July 13, 2015	Test Date:June 03, 2013	

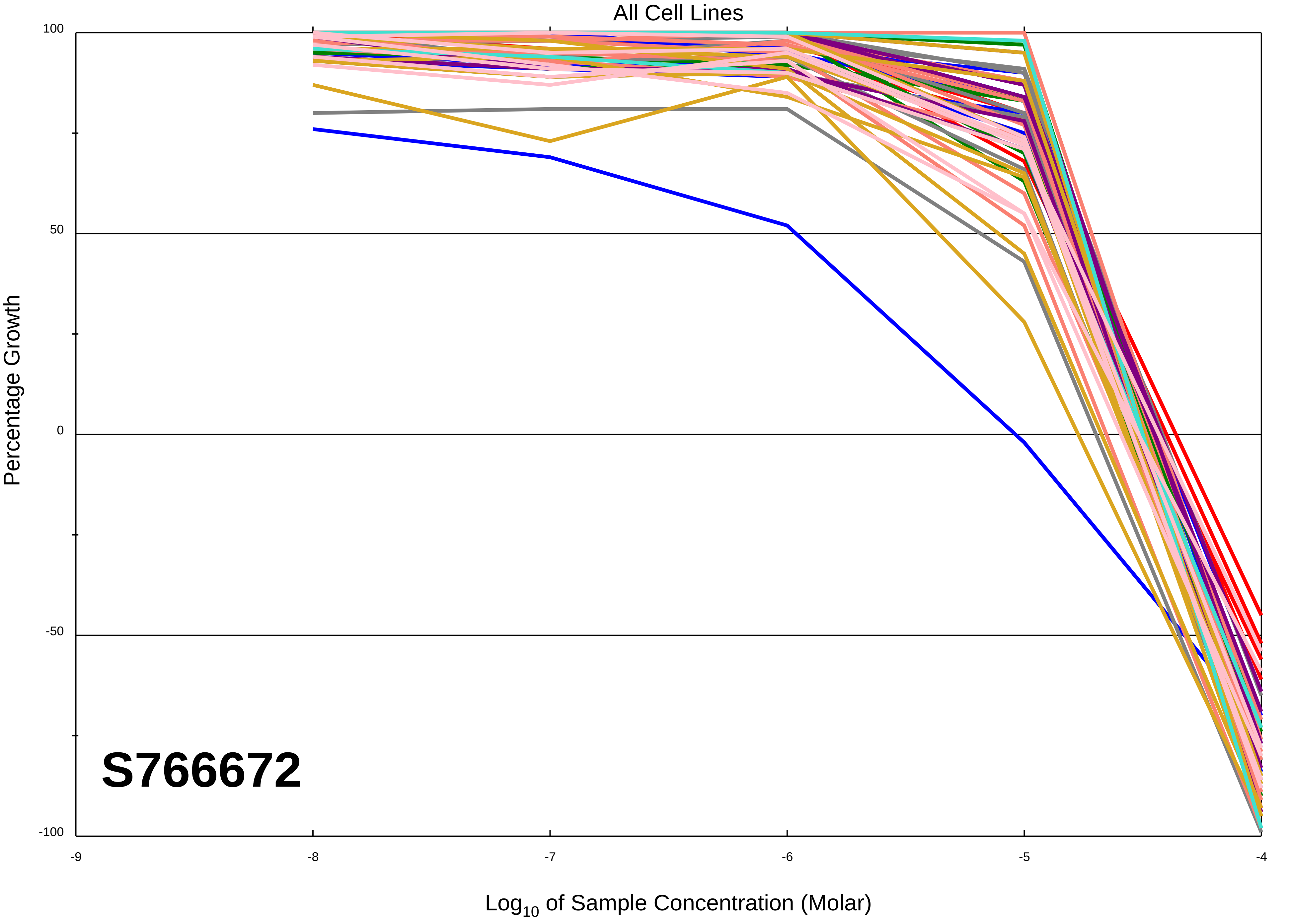

National Cancer Institute Developmental Therapeutics Program<br>Dose Response Curves	NSC: 766692 / 2	SSPL: 0YQS	EXP. ID: 1302NS28
	Report Date: July 13, 2015	Test Date: February 25, 2013	

Leukemia

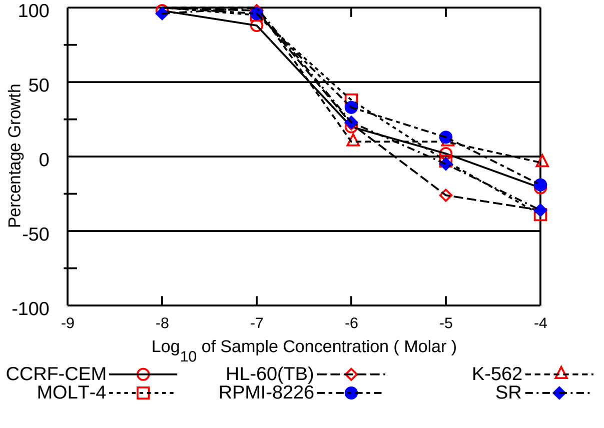

Non-Small Cell Lung Cancer

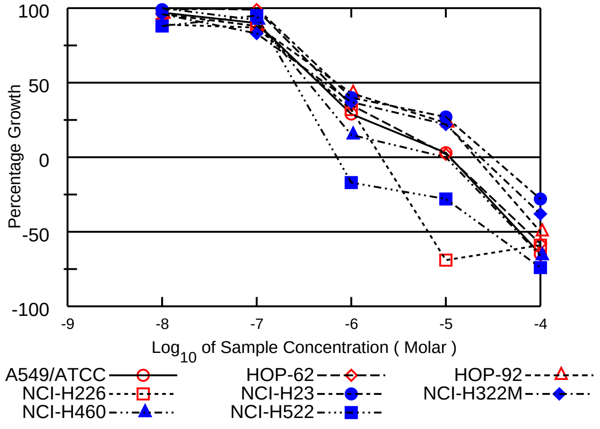

Colon Cancer

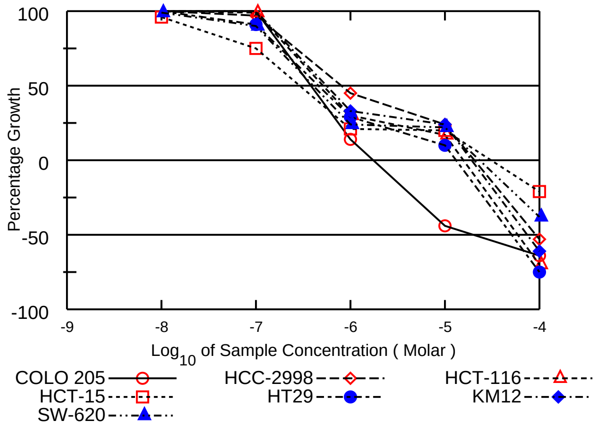

CNS Cancer

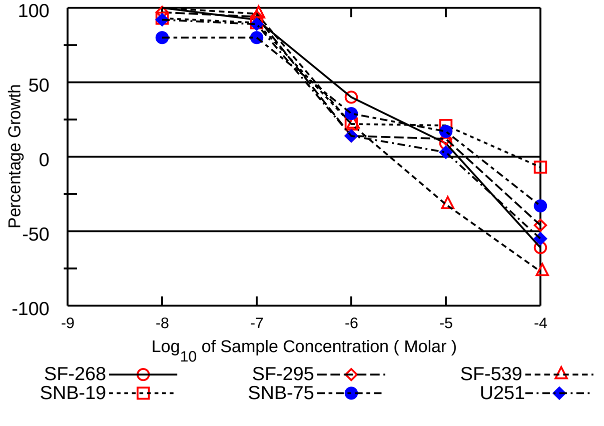

Melanoma

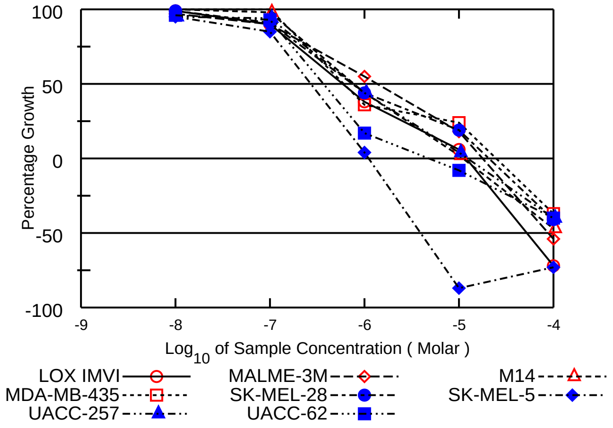

Ovarian Cancer

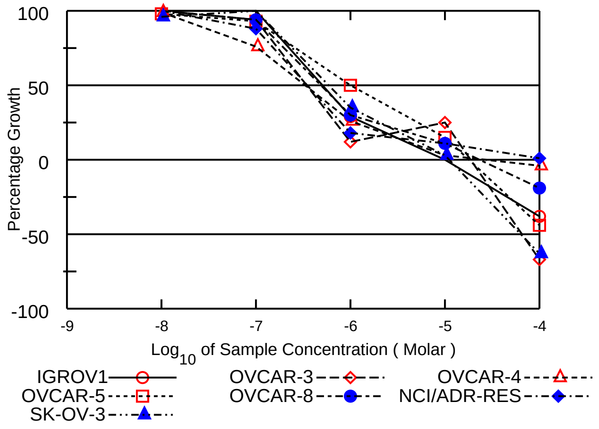

Renal Cancer

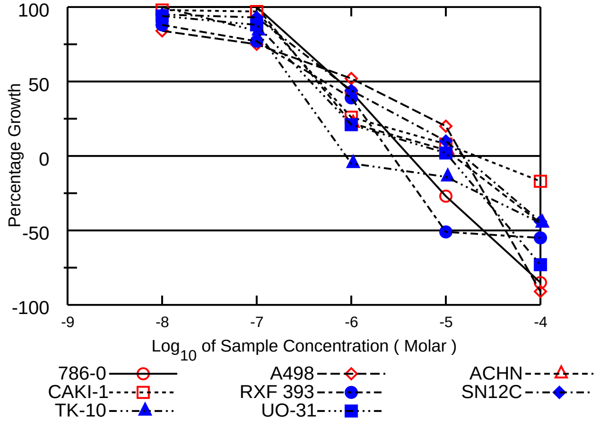

Prostate Cancer

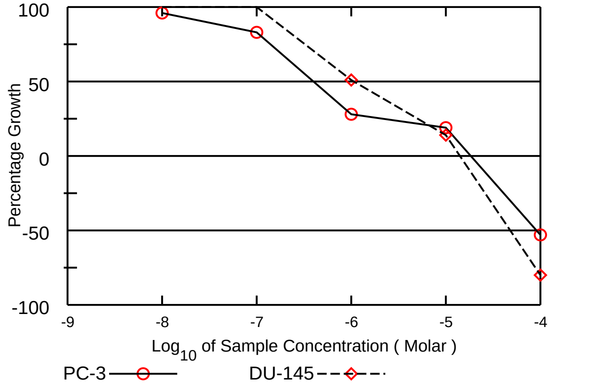

Breast Cancer

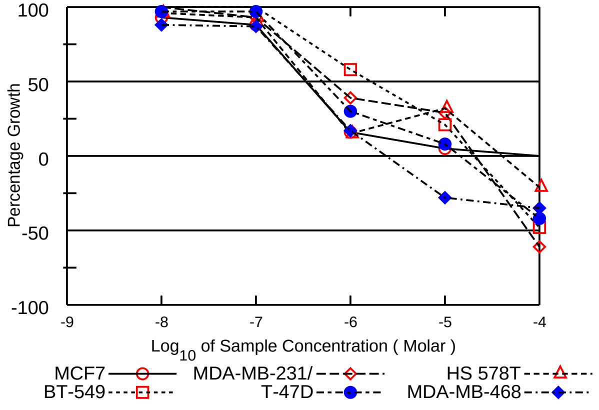

National Cancer Institute Developmental Therapeutics Program Mean Graphs	NSC :766692/2	Units :Molar	SSPL :0YQS	EXP. ID :1302NS28
	Report Date :July 13, 2015		Test Date :February 25, 2013	

Panel/Cell Line	Log_{10}GI50	Log_{10}TGI	Log_{10}LC50
**Leukemia**			
CCRF-CEM	-6.44	-4.90	> -4.00
HL-60(TB)	-6.37	-5.55	> -4.00
K-562	-6.33	-4.26	> -4.00
MOLT-4	-6.21	-5.07	> -4.00
RPMI-8226	-6.27	-4.59	> -4.00
SR	-6.34	-5.19	> -4.00
**Non-Small Cell Lung Cancer**			
A549/ATCC	-6.34	-4.96	-4.22
HOP-62	-6.23	-4.97	-4.13
HOP-92	-6.16	-4.69	-4.01
NCI-H226	-6.33	-5.69	-5.19
NCI-H23	-6.16	-4.51	> -4.00
NCI-H322M	-6.28	-4.64	> -4.00
NCI-H460	-6.46	-4.99	-4.24
NCI-H522	-6.60	-6.15	-4.52
**Colon Cancer**			
COLO 205	-6.38	-5.75	-4.70
HCC-2998	-6.10	-4.69	-4.04
HCT-116	-6.29	-4.81	-4.23
HCT-15	-6.54	-4.52	> -4.00
HT29	-6.34	-4.88	-4.29
KM12	-6.25	-4.72	-4.13
SW-620	-6.40	-4.63	> -4.00
**CNS Cancer**			
SF-268	-6.19	-4.87	-4.16
SF-295	-6.45	-4.80	> -4.00
SF-539	-6.38	-5.60	-4.60
SNB-19	-6.41	-4.25	> -4.00
SNB-75	-6.41	-4.66	> -4.00
U251	-6.48	-4.94	-4.08
**Melanoma**			
LOX IMVI	-6.23	-4.92	-4.28
MALME-3M	-5.88	-4.75	-4.06
M14	-6.11	-4.96	> -4.00
MDA-MB-435	-6.21	-4.60	> -4.00
SK-MEL-28	-6.12	-4.68	> -4.00
SK-MEL-5	-6.57	-5.95	-5.41
UACC-257	-6.11	-4.90	> -4.00
UACC-62	-6.43	-5.32	> -4.00
**Ovarian Cancer**			
IGROV1	-6.28	-4.99	> -4.00
OVCAR-3	-6.46	-4.73	-4.19
OVCAR-4	-6.48	-4.59	> -4.00
OVCAR-5	-6.00	-4.74	> -4.00
OVCAR-8	-6.31	-4.65	> -4.00
NCI/ADR-RES	-6.45	> -4.00	> -4.00
SK-OV-3	-6.23	-4.96	-4.19
**Renal Cancer**			
786-0	-6.12	-5.39	-4.61
A498	-5.94	-4.82	-4.37
ACHN	-6.33	-4.91	> -4.00
CAKI-1	-6.34	-4.68	> -4.00
RXF 393	-6.29	-5.57	-5.01
SN12C	-6.12	-4.82	> -4.00
TK-10	-6.61	-6.06	> -4.00
UO-31	-6.43	-4.97	-4.31
**Prostate Cancer**			
PC-3	-6.40	-4.74	-4.04
DU-145	-5.97	-4.85	-4.32
**Breast Cancer**			
MCF7	-6.47	> -4.00	> -4.00
MDA-MB-231/ATCC	-6.20	-4.68	-4.12
HS 578T	-6.45	-4.40	> -4.00
BT-549	-5.79	-4.70	> -4.00
T-47D	-6.30	-4.84	> -4.00
MDA-MB-468	-6.47	-5.62	> -4.00
_MID	-6.3	-4.91	-4.16
Delta	0.31	1.24	1.25
Range	0.82	2.15	1.41

Axis (GI50, TGI, LC50): +3 +2 +1 0 -1 -2 -3

National Cancer Institute Developmental Therapeutics Program<br>Dose Response Curves	NSC:766692/2	SSPL:0YQS	EXP. ID:1302NS28
	Report Date:July 13, 2015	Test Date:February 25, 2013	

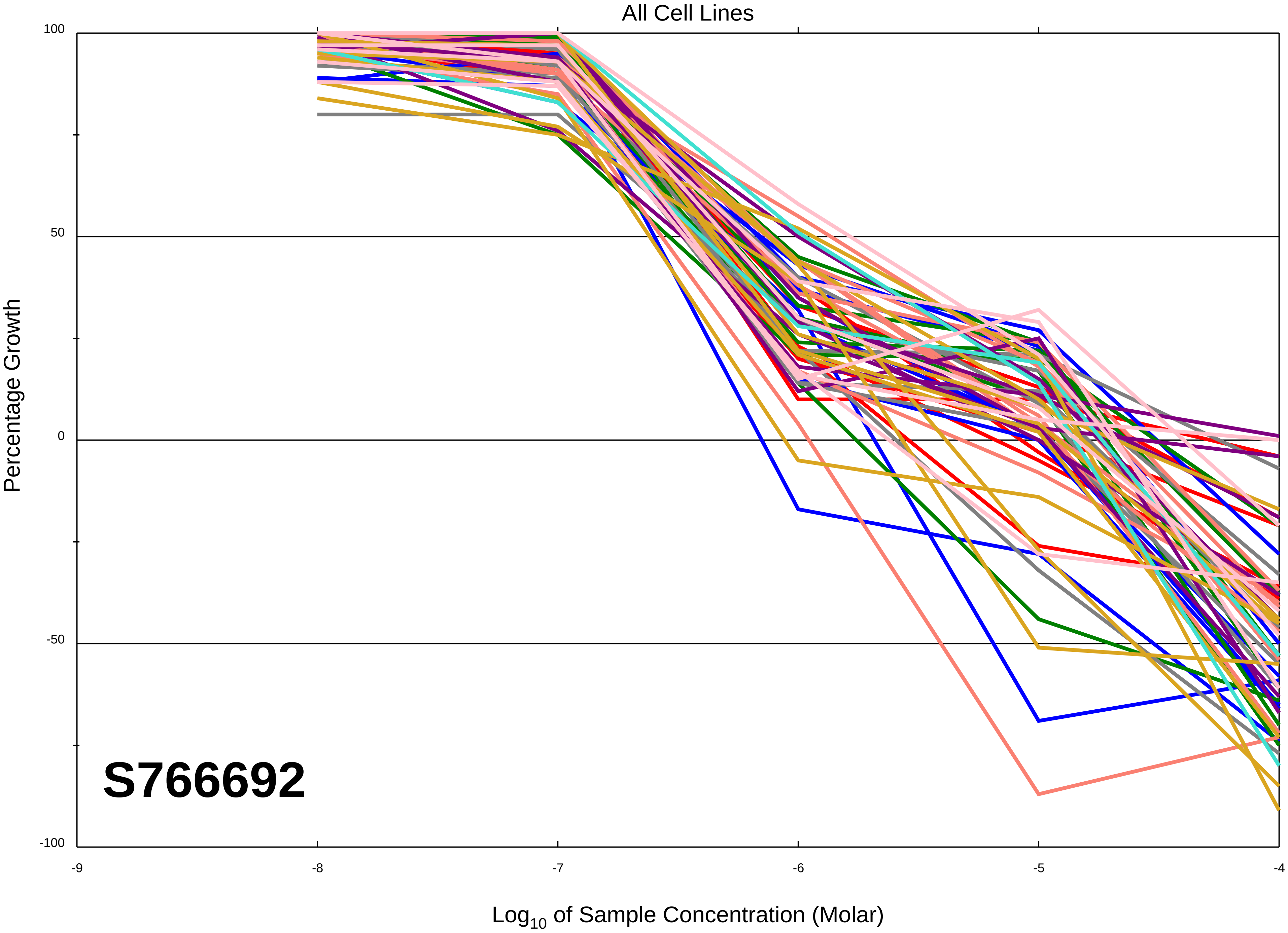

National Cancer Institute Developmental Therapeutics Program<br>Dose Response Curves	NSC: 766692 / 2	SSPL: 0YQS	EXP. ID: 1306RS02
	Report Date: July 13, 2015	Test Date: June 03, 2013	

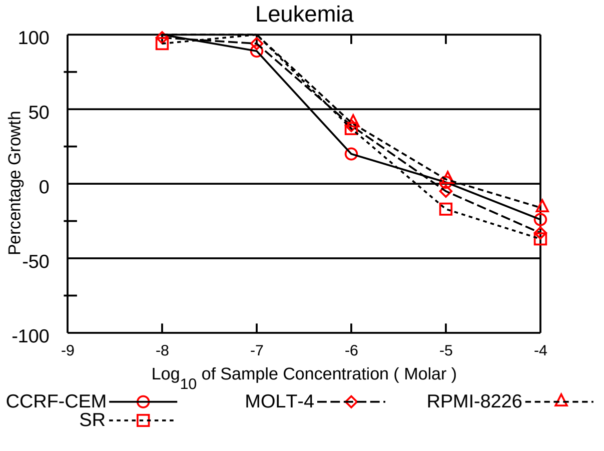

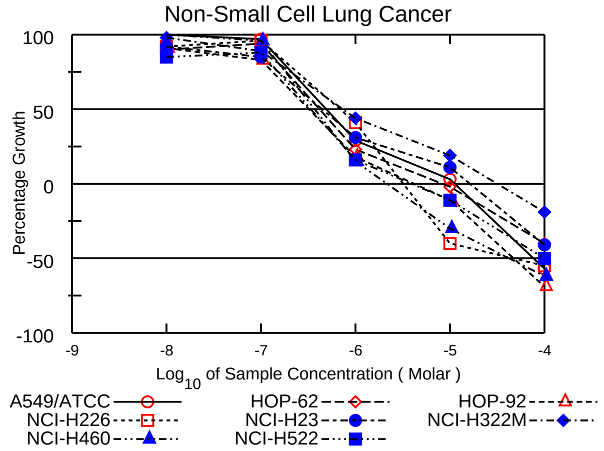

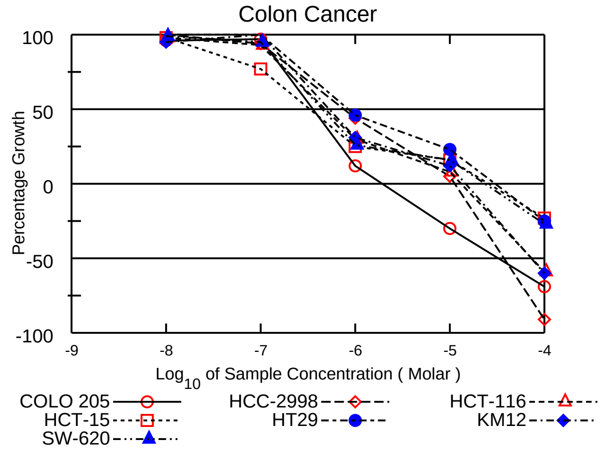

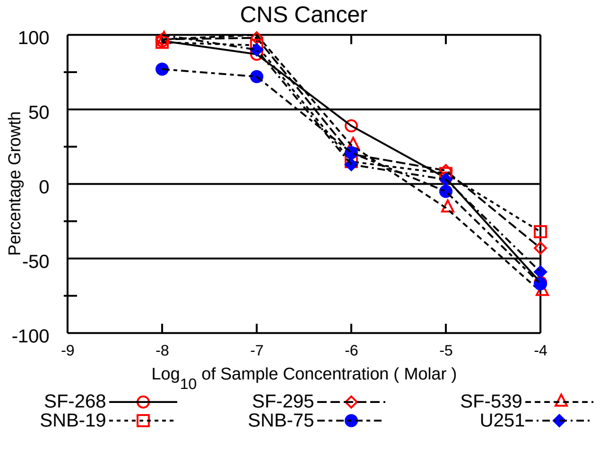

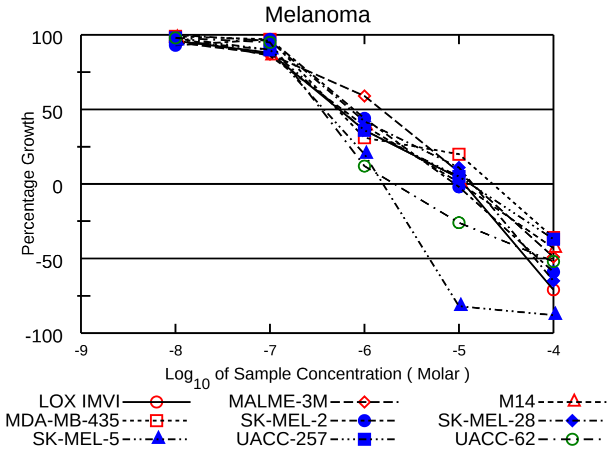

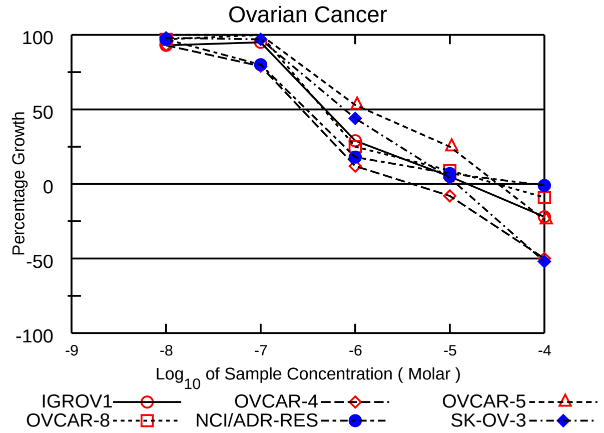

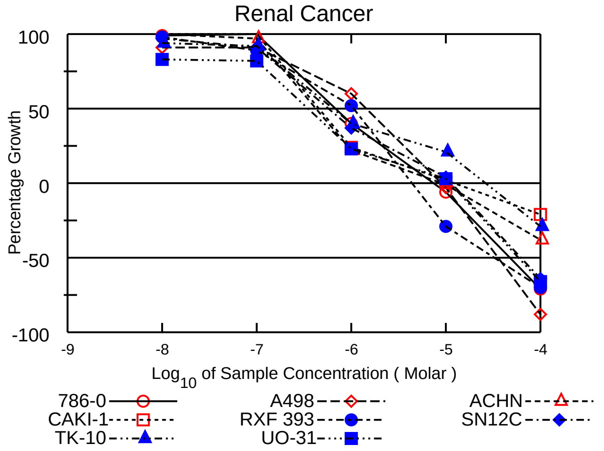

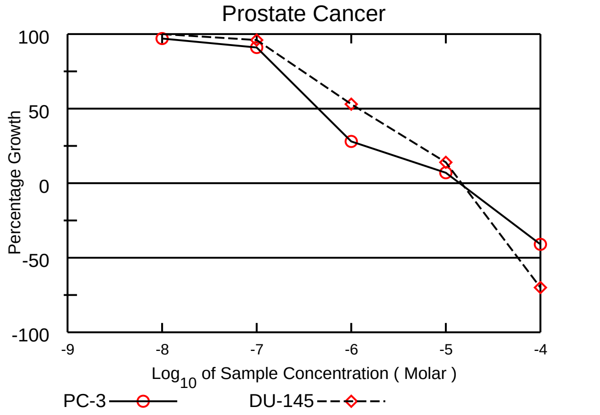

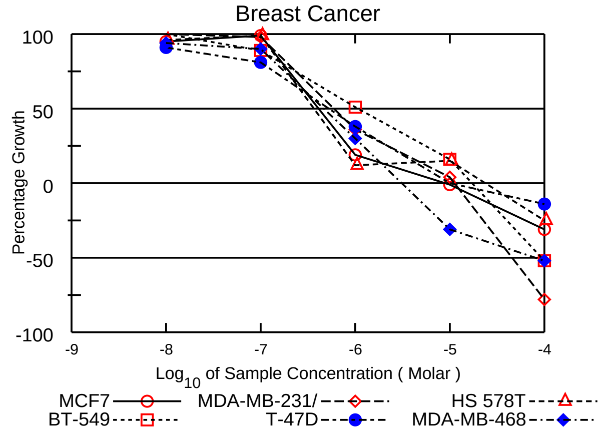

National Cancer Institute Developmental Therapeutics Program Mean Graphs	NSC :766692/2	Units :Molar	SSPL :0YQS	EXP. ID :1306RS02
	Report Date :July 13, 2015		Test Date :June 03, 2013	

Panel/Cell Line	Log_{10}GI50	Log_{10}TGI	Log_{10}LC50
**Leukemia**			
CCRF-CEM	-6.44	-4.96	> -4.00
MOLT-4	-6.20	-5.12	> -4.00
RPMI-8226	-6.15	-4.85	> -4.00
SR	-6.19	-5.31	> -4.00
**Non-Small Cell Lung Cancer**			
A549/ATCC	-6.31	-4.95	-4.11
HOP-62	-6.38	-5.07	> -4.00
HOP-92	-6.49	-5.37	-4.32
NCI-H226	-6.16	-5.49	-4.32
NCI-H23	-6.35	-4.79	> -4.00
NCI-H322M	-6.14	-4.49	> -4.00
NCI-H460	-6.43	-5.67	-4.38
NCI-H522	-6.47	-5.41	-4.00
**Colon Cancer**			
COLO 205	-6.45	-5.71	-4.49
HCC-2998	-6.11	-4.95	-4.43
HCT-116	-6.32	-4.88	-4.13
HCT-15	-6.48	-4.58	> -4.00
HT29	-6.07	-4.52	> -4.00
KM12	-6.27	-4.83	-4.13
SW-620	-6.34	-4.63	> -4.00
**CNS Cancer**			
SF-268	-6.23	-4.95	-4.23
SF-295	-6.39	-4.84	> -4.00
SF-539	-6.32	-5.37	-4.39
SNB-19	-6.45	-4.83	> -4.00
SNB-75	-6.56	-5.19	-4.28
U251	-6.48	-4.96	-4.14
**Melanoma**			
LOX IMVI	-6.27	-4.94	-4.28
MALME-3M	-5.82	-4.86	> -4.00
M14	-6.23	-4.97	> -4.00
MDA-MB-435	-6.29	-4.65	> -4.00
SK-MEL-2	-6.12	-5.04	-4.16
SK-MEL-28	-6.14	-4.85	-4.20
SK-MEL-5	-6.43	-5.81	-5.32
UACC-257	-6.26	-4.89	> -4.00
UACC-62	-6.46	-5.68	-4.09
**Ovarian Cancer**			
IGROV1	-6.32	-4.82	> -4.00
OVCAR-4	-6.57	-5.39	-4.01
OVCAR-5	-5.89	-4.49	> -4.00
OVCAR-8	-6.34	-4.50	> -4.00
NCI/ADR-RES	-6.51	-4.10	> -4.00
SK-OV-3	-6.11	-4.94	-4.04
**Renal Cancer**			
786-0	-6.16	-5.13	-4.32
A498	-5.84	-5.05	-4.45
ACHN	-6.37	-5.06	> -4.00
CAKI-1	-6.33	-4.92	> -4.00
RXF 393	-5.97	-5.36	-4.49
SN12C	-6.24	-4.95	-4.20
TK-10	-6.20	-4.58	> -4.00
UO-31	-6.45	-4.96	-4.24
**Prostate Cancer**			
PC-3	-6.35	-4.85	> -4.00
DU-145	-5.92	-4.84	-4.24
**Breast Cancer**			
MCF7	-6.39	-5.04	> -4.00
MDA-MB-231/ATCC	-6.22	-4.95	-4.34
HS 578T	-6.43	-4.63	> -4.00
BT-549	-5.97	-4.76	-4.03
T-47D	-6.27	-5.00	> -4.00
MDA-MB-468	-6.33	-5.51	-4.11
_MID	-6.27	-4.99	-4.14
Delta	0.3	0.82	1.18
Range	0.75	1.71	1.32

GI50 / TGI / LC50 axis scale: +3 +2 +1 0 -1 -2 -3

National Cancer Institute Developmental Therapeutics Program Dose Response Curves	NSC:766692/2	SSPL:0YQS	EXP. ID:1306RS02
	Report Date:July 13, 2015	Test Date:June 03, 2013	

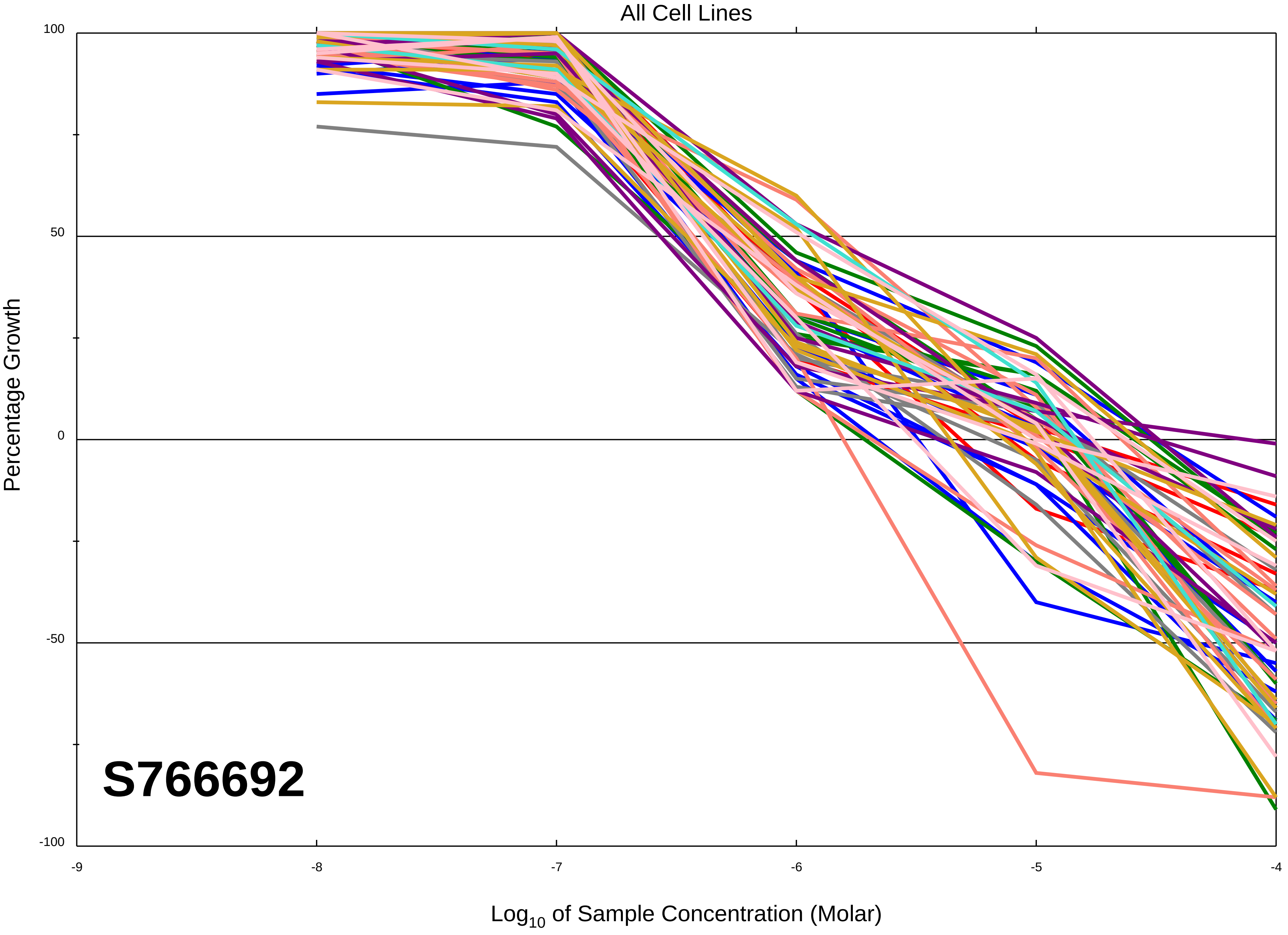

National Cancer Institute Developmental Therapeutics Program<br>Dose Response Curves	NSC: 766722 / 2	SSPL: 0YQS	EXP. ID: 1302NS28
	Report Date: July 13, 2015	Test Date: February 25, 2013	

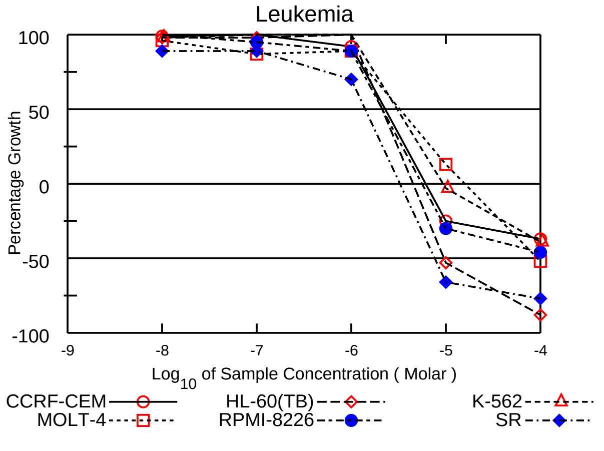

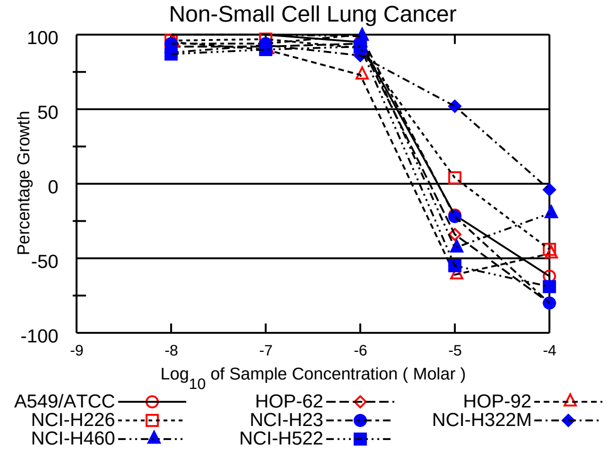

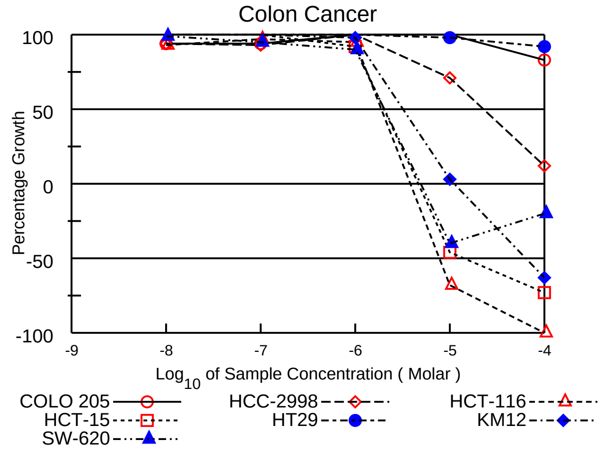

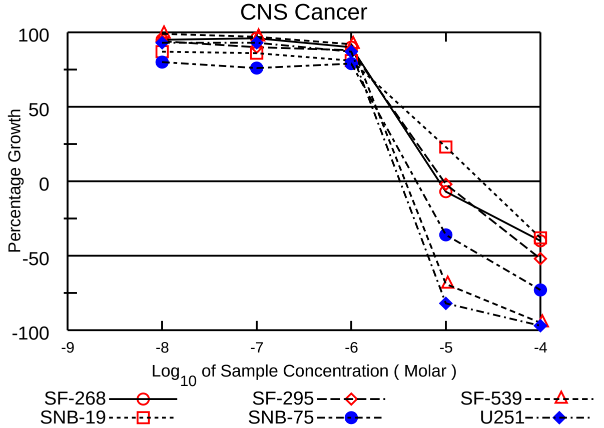

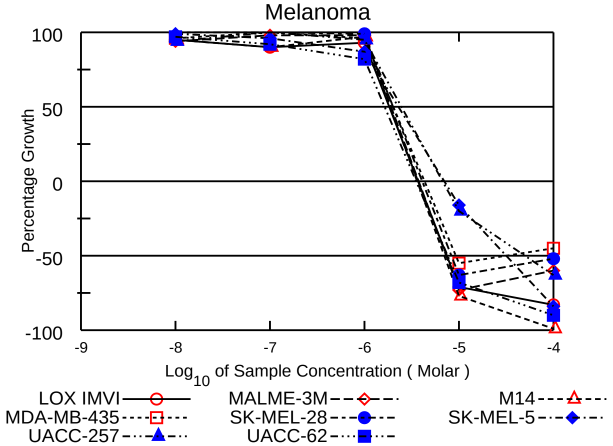

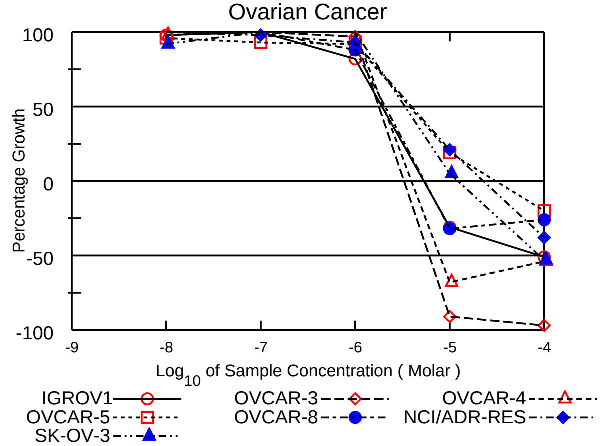

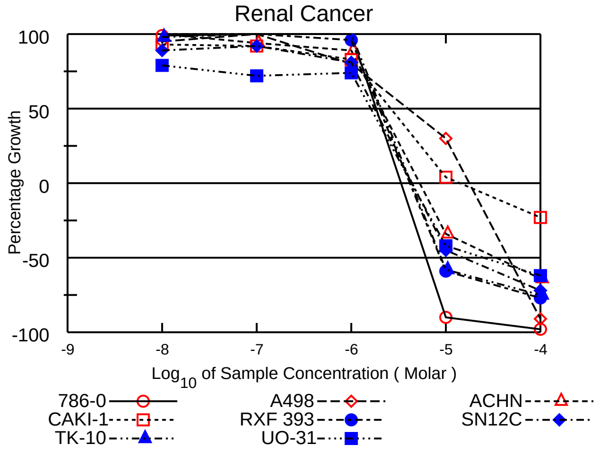

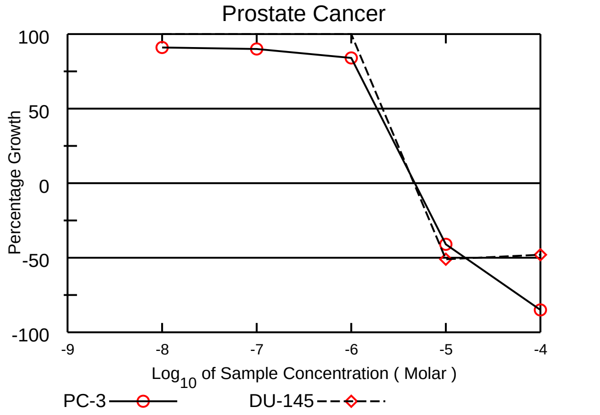

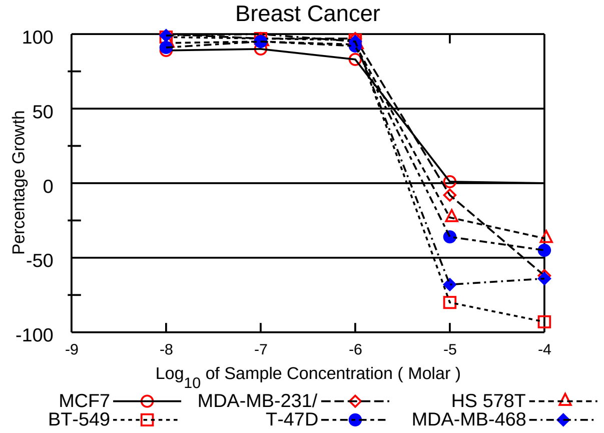

National Cancer Institute Developmental Therapeutics Program Mean Graphs	NSC :766722/2	Units :Molar	SSPL :0YQS	EXP. ID :1302NS28
	Report Date :July 13, 2015		Test Date :February 25, 2013	

Panel/Cell Line	Log_{10}GI50	Log_{10}TGI	Log_{10}LC50
**Leukemia**			
CCRF-CEM	-5.64	-5.22	> -4.00
HL-60(TB)	-5.67	-5.34	-5.02
K-562	-5.51	-5.03	> -4.00
MOLT-4	-5.49	-4.80	-4.04
RPMI-8226	-5.67	-5.25	> -4.00
SR	-5.85	-5.49	-5.12
**Non-Small Cell Lung Cancer**			
A549/ATCC	-5.61	-5.18	-4.29
HOP-62	-5.66	-5.26	-4.65
HOP-92	-5.83	-5.45	
NCI-H226	-5.53	-4.92	> -4.00
NCI-H23	-5.59	-5.18	-4.51
NCI-H322M	-4.96	-4.07	> -4.00
NCI-H460	-5.66	-5.31	> -4.00
NCI-H522	-5.71	-5.37	-5.03
**Colon Cancer**			
COLO 205	> -4.00	> -4.00	> -4.00
HCC-2998	-4.64	> -4.00	> -4.00
HCT-116	-5.72	-5.42	-5.11
HCT-15	-5.70	-5.33	-4.86
HT29	> -4.00	> -4.00	> -4.00
KM12	-5.49	-4.96	-4.20
SW-620	-5.69	-5.31	> -4.00
**CNS Cancer**			
SF-268	-5.59	-5.07	> -4.00
SF-295	-5.58	-5.02	-4.05
SF-539	-5.74	-5.43	-5.12
SNB-19	-5.46	-4.62	> -4.00
SNB-75	-5.75	-5.31	-4.62
U251	-5.78	-5.49	-5.19
**Melanoma**			
LOX IMVI	-5.74	-5.43	-5.13
MALME-3M	-5.72	-5.43	-5.13
M14	-5.73	-5.44	-5.16
MDA-MB-435	-5.70	-5.37	
SK-MEL-28	-5.70	-5.39	-5.08
SK-MEL-5	-5.64	-5.15	-4.50
UACC-257	-5.61	-5.18	-4.31
UACC-62	-5.79	-5.45	-5.12
**Ovarian Cancer**			
IGROV1	-5.72	-5.28	-4.03
OVCAR-3	-5.75	-5.48	-5.22
OVCAR-4	-5.69	-5.40	-5.10
OVCAR-5	-5.43	-4.51	> -4.00
OVCAR-8	-5.68	-5.27	> -4.00
NCI/ADR-RES	-5.40	-4.64	> -4.00
SK-OV-3	-5.47	-4.91	-4.05
**Renal Cancer**			
786-0	-5.74	-5.47	-5.21
A498	-5.40	-4.75	-4.34
ACHN	-5.68	-5.28	-4.47
CAKI-1	-5.58	-4.86	> -4.00
RXF 393	-5.70	-5.38	-5.06
SN12C	-5.75	-5.35	-4.81
TK-10	-5.63	-5.34	-5.05
UO-31	-5.79	-5.36	-4.61
**Prostate Cancer**			
PC-3	-5.73	-5.33	-4.79
DU-145	-5.66	-5.33	
**Breast Cancer**			
MCF7	-5.60	> -4.00	> -4.00
MDA-MB-231/ATCC	-5.55	-5.08	-4.23
HS 578T	-5.63	-5.20	> -4.00
BT-549	-5.74	-5.46	-5.17
T-47D	-5.67	-5.28	> -4.00
MDA-MB-468	-5.72	-5.41	-5.11
_MID	-5.57	-5.12	-4.5
Delta	0.28	0.37	0.72
Range	1.85	1.49	1.22

Scale (GI50 / TGI / LC50): +3 +2 +1 0 -1 -2 -3

National Cancer Institute Developmental Therapeutics Program Dose Response Curves	NSC:766722/2	SSPL:0YQS	EXP. ID:1302NS28
	Report Date:July 13, 2015	Test Date:February 25, 2013	

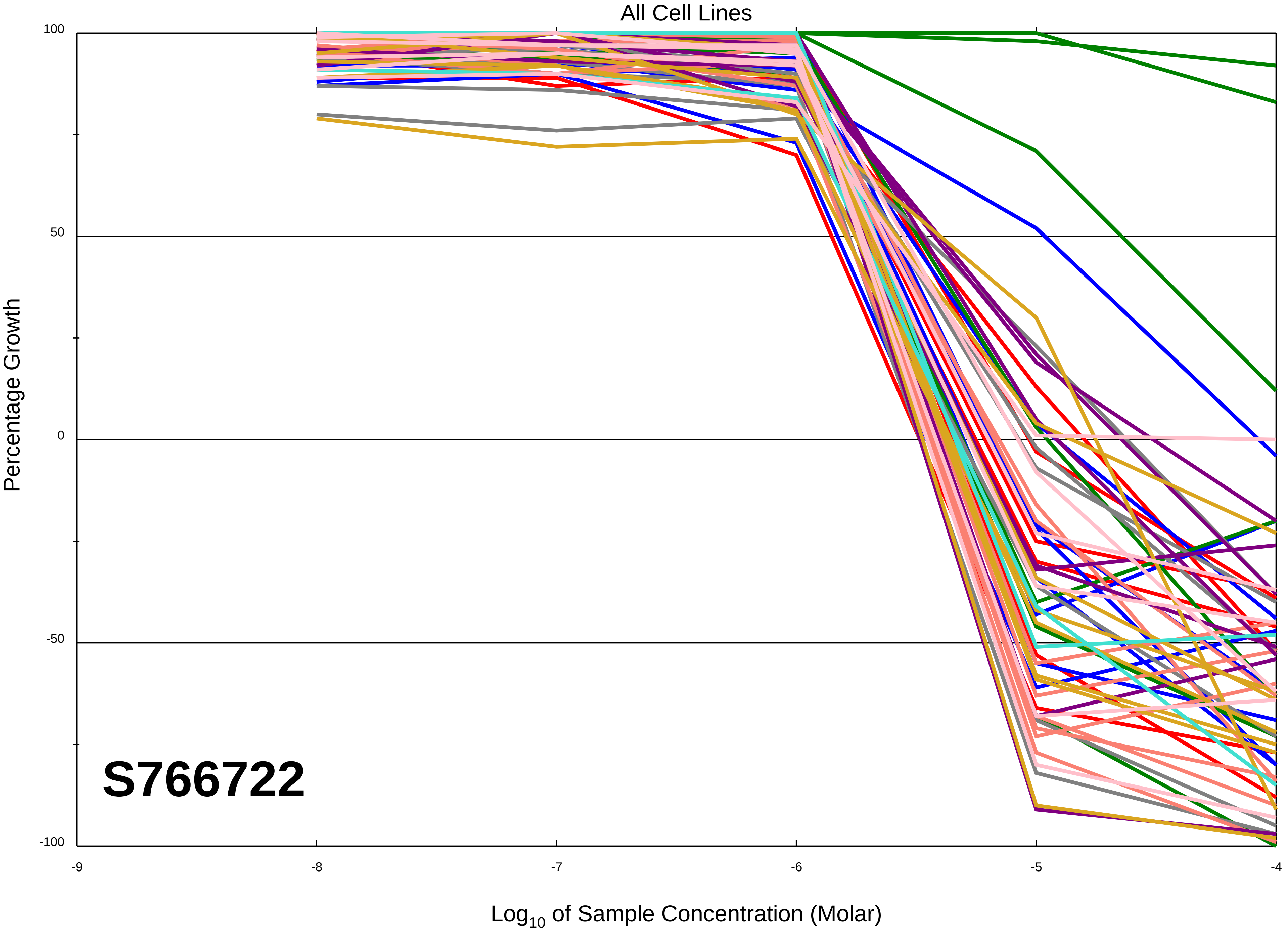

National Cancer Institute Developmental Therapeutics Program<br>Dose Response Curves	NSC: 766722 / 2	SSPL: 0YQS	EXP. ID: 1306RS02
	Report Date: July 13, 2015	Test Date: June 03, 2013	

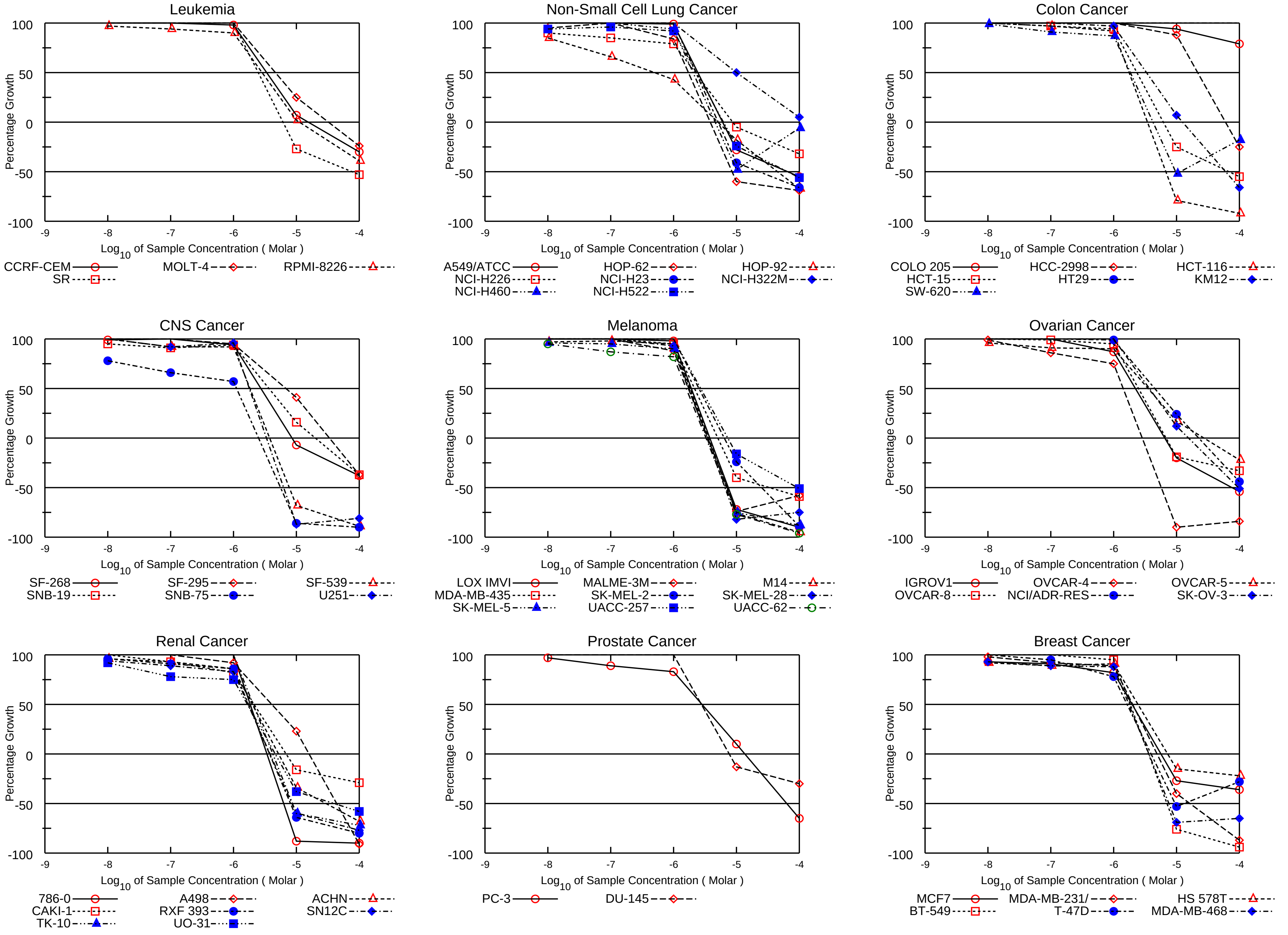

National Cancer Institute Developmental Therapeutics Program	NSC :766722/2	Units :Molar	SSPL :0YQS	EXP. ID :1306RS02
Mean Graphs	Report Date :July 13, 2015		Test Date :June 03, 2013	

Panel/Cell Line	Log_{10}GI50	GI50	Log_{10}TGI	TGI	Log_{10}LC50	LC50
**Leukemia**						
CCRF-CEM	-5.47		-4.82		> -4.00	
MOLT-4	-5.33		-4.49		> -4.00	
RPMI-8226	-5.55		-4.96		> -4.00	
SR	-5.61		-5.21		-4.12	
**Non-Small Cell Lung Cancer**						
A549/ATCC	-5.61		-5.22		-4.20	
HOP-62	-5.76		-5.42		-5.07	
HOP-92	-6.31		-5.29		-4.34	
NCI-H226	-5.66		-5.06		> -4.00	
NCI-H23	-5.63		-5.29		-4.64	
NCI-H322M	-5.00		> -4.00		> -4.00	
NCI-H460	-5.69		-5.34		> -4.00	
NCI-H522	-5.64		-5.21		-4.19	
**Colon Cancer**						
COLO 205	> -4.00		> -4.00		> -4.00	
HCC-2998	-4.66		-4.22		> -4.00	
HCT-116	-5.75		-5.46		-5.17	
HCT-15	-5.63		-5.21		-4.18	
HT29	> -4.00		> -4.00		> -4.00	
KM12	-5.47		-4.90		-4.22	
SW-620	-5.74		-5.38			
**CNS Cancer**						
SF-268	-5.57		-5.07		> -4.00	
SF-295	-5.16		-4.47		> -4.00	
SF-539	-5.74		-5.42		-5.11	
SNB-19	-5.43		-4.69		> -4.00	
SNB-75	-5.95		-5.60		-5.25	
U251	-5.75		-5.48		-5.20	
**Melanoma**						
LOX IMVI	-5.72		-5.42		-5.13	
MALME-3M	-5.77		-5.46		-5.15	
M14	-5.74		-5.45		-5.15	
MDA-MB-435	-5.67		-5.30		-4.47	
SK-MEL-2	-5.56		-5.18		-4.59	
SK-MEL-28	-5.76		-5.47		-5.18	
SK-MEL-5	-5.76		-5.45		-5.15	
UACC-257	-5.56		-5.13		-4.03	
UACC-62	-5.80		-5.48		-5.17	
**Ovarian Cancer**						
IGROV1	-5.65		-5.18		-4.13	
OVCAR-4	-5.85		-5.55		-5.24	
OVCAR-5	-5.45		-4.57		> -4.00	
OVCAR-8	-5.61		-5.17		> -4.00	
NCI/ADR-RES	-5.34		-4.64		> -4.00	
SK-OV-3	-5.42		-4.81		-4.02	
**Renal Cancer**						
786-0	-5.71		-5.46		-5.20	
A498	-5.39		-4.80		-4.36	
ACHN	-5.70		-5.28		-4.53	
CAKI-1	-5.67		-5.16		> -4.00	
RXF 393	-5.76		-5.43		-5.09	
SN12C	-5.77		-5.42		-5.07	
TK-10	-5.65		-5.36		-5.06	
UO-31	-5.78		-5.33		-4.38	
**Prostate Cancer**						
PC-3	-5.55		-4.87		-4.20	
DU-145	-5.53		-5.11		> -4.00	
**Breast Cancer**						
MCF7	-5.70		-5.25		> -4.00	
MDA-MB-231/ATCC	-5.70		-5.31		-4.78	
HS 578T	-5.62		-5.15		> -4.00	
BT-549	-5.74		-5.44		-5.15	
T-47D	-5.78		-5.40			
MDA-MB-468	-5.76		-5.44			-5.12
_MID	-5.56		-5.12		-4.48	
Delta	0.75		0.48		0.77	
Range	2.31		1.6		1.25	

+3 +2 +1 0 -1 -2 -3

National Cancer Institute Developmental Therapeutics Program Dose Response Curves	NSC:766722/2	SSPL:0YQS	EXP. ID:1306RS02
	Report Date:July 13, 2015	Test Date:June 03, 2013	

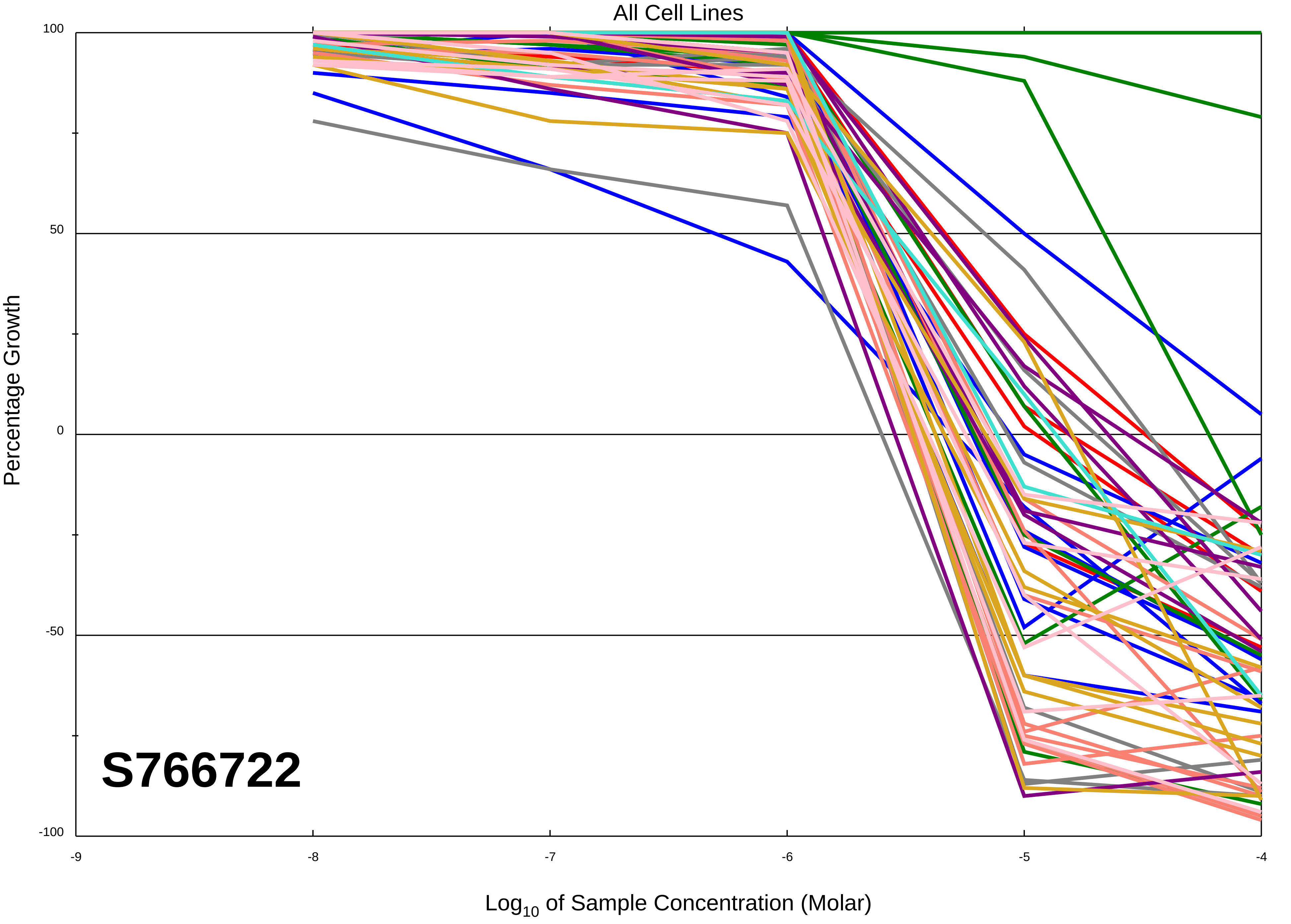

National Cancer Institute Developmental Therapeutics Program<br>Dose Response Curves	NSC: 766755 / 2	SSPL: 0YQS	EXP. ID: 1302NS28
	Report Date: July 13, 2015	Test Date: February 25, 2013	

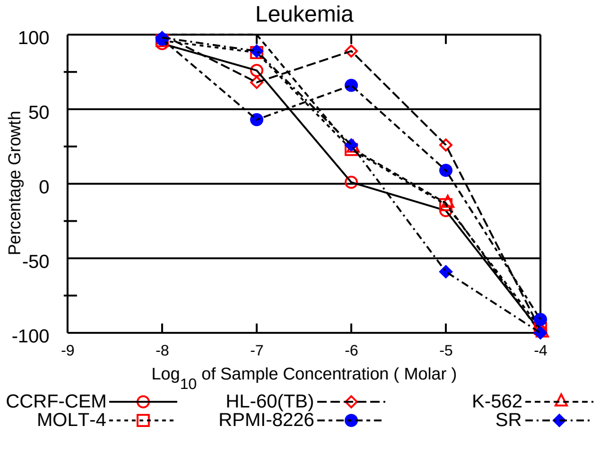

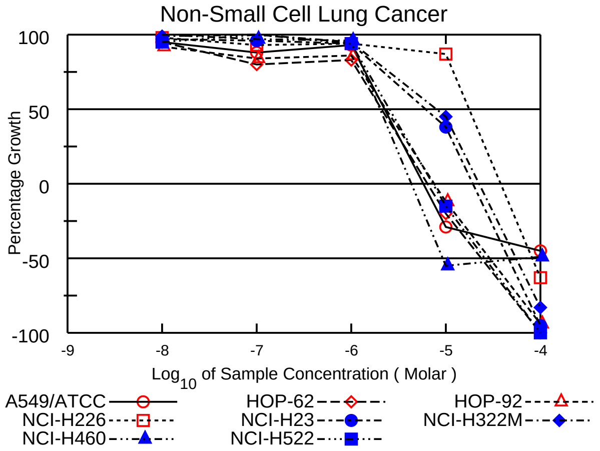

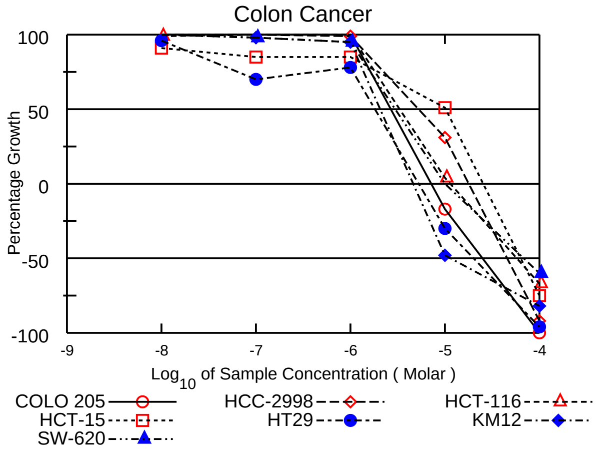

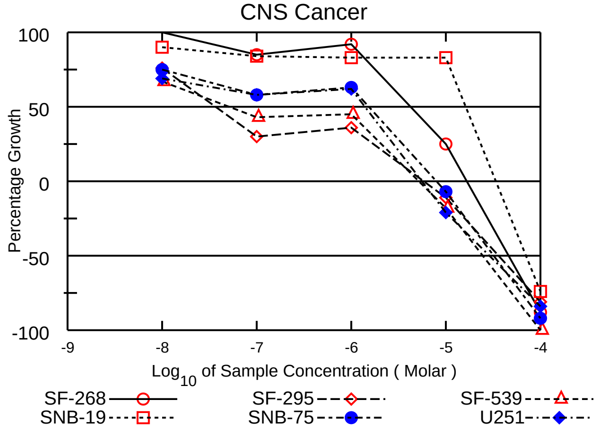

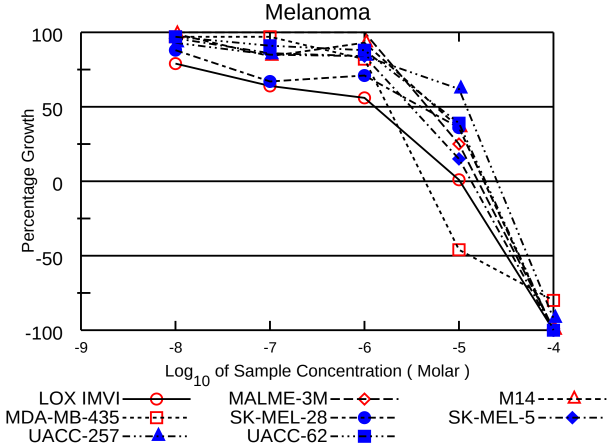

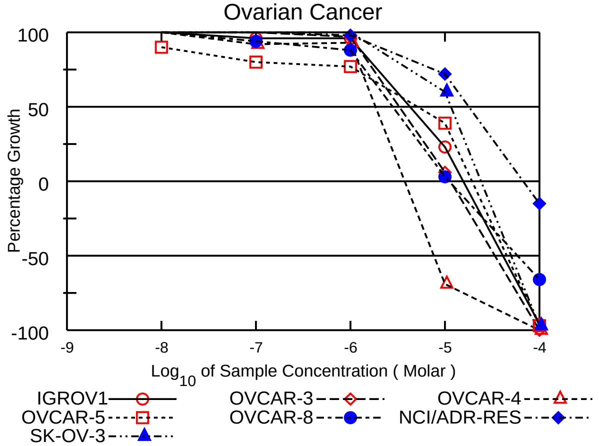

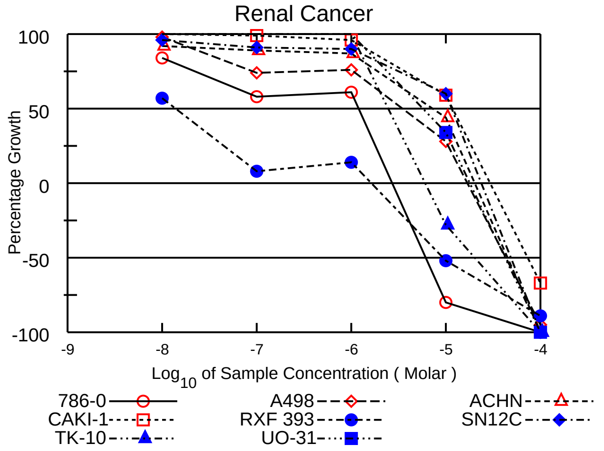

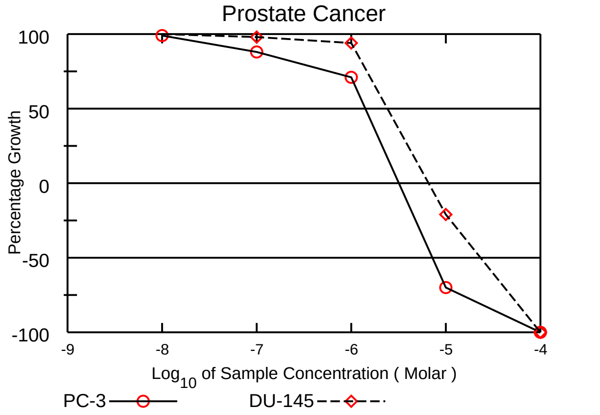

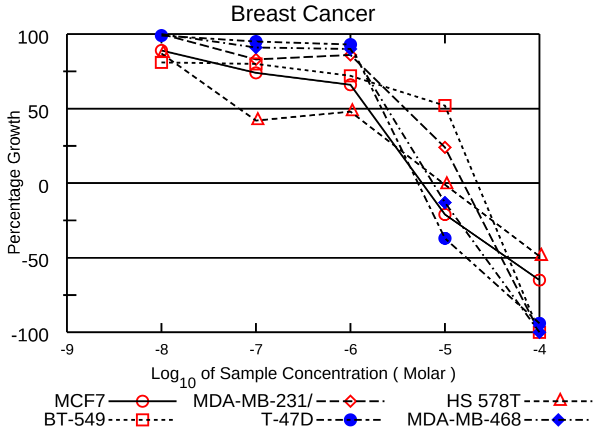

National Cancer Institute Developmental Therapeutics Program Mean Graphs	NSC :766755/2	Units :Molar	SSPL :0YQS	EXP. ID :1302NS28
	Report Date :July 13, 2015		Test Date :February 25, 2013	

Panel/Cell Line	Log_{10}GI50	Log_{10}TGI	Log_{10}LC50
**Leukemia**			
CCRF-CEM	-6.66	-5.94	-4.60
HL-60(TB)	-5.38	-4.79	-4.39
K-562	-6.31	-5.34	-4.57
MOLT-4	-6.41	-5.37	-4.56
RPMI-8226		-4.91	-4.41
SR	-6.38	-5.69	-5.11
**Non-Small Cell Lung Cancer**			
A549/ATCC	-5.64	-5.24	> -4.00
HOP-62	-5.68	-5.19	-4.62
HOP-92	-5.63	-5.12	-4.54
NCI-H226	-4.75	-4.42	-4.09
NCI-H23	-5.21	-4.71	-4.34
NCI-H322M	-5.09	-4.65	-4.26
NCI-H460	-5.70	-5.36	
NCI-H522	-5.60	-5.14	-4.59
**Colon Cancer**			
COLO 205	-5.56	-5.15	-4.61
HCC-2998	-5.28	-4.75	-4.34
HCT-116	-5.48	-4.94	-4.24
HCT-15	-4.99	-4.60	-4.20
HT29	-5.74	-5.28	-4.70
KM12	-5.68	-5.33	-4.93
SW-620	-5.53	-5.00	-4.16
**CNS Cancer**			
SF-268	-5.37	-4.78	-4.34
SF-295	-7.43	-5.23	-4.44
SF-539	-7.28	-5.29	-4.61
SNB-19	-4.79	-4.47	-4.15
SNB-75	-5.82	-5.10	-4.50
U251	-5.86	-5.25	-4.54
**Melanoma**			
LOX IMVI	-5.90	-4.99	-4.49
MALME-3M	-5.29	-4.80	-4.39
M14	-5.24	-4.73	-4.37
MDA-MB-435	-5.75	-5.36	-4.88
SK-MEL-28	-5.40	-4.73	-4.37
SK-MEL-5	-5.51	-4.87	-4.43
UACC-257	-4.92	-4.60	-4.27
UACC-62	-5.23	-4.72	-4.36
**Ovarian Cancer**			
IGROV1	-5.37	-4.81	-4.39
OVCAR-3	-5.48	-4.94	-4.47
OVCAR-4	-5.74	-5.43	-5.12
OVCAR-5	-5.28	-4.71	-4.34
OVCAR-8	-5.55	-4.95	-4.23
NCI/ADR-RES	-4.75	-4.17	> -4.00
SK-OV-3	-4.93	-4.62	-4.30
**Renal Cancer**			
786-0	-5.92	-5.57	-5.21
A498	-5.46	-4.77	-4.37
ACHN	-5.14	-4.69	-4.35
CAKI-1	-4.93	-4.53	-4.13
RXF 393	-7.86	-5.79	-5.04
SN12C	-4.94	-4.62	-4.31
TK-10	-5.59	-5.21	-4.70
UO-31	-5.19	-4.75	-4.37
**Prostate Cancer**			
PC-3	-5.85	-5.50	-5.14
DU-145	-5.62	-5.18	-4.63
**Breast Cancer**			
MCF7	-5.82	-5.24	-4.34
MDA-MB-231/ATCC	-5.42	-4.81	-4.40
HS 578T	-7.18	-5.02	> -4.00
BT-549	-4.98	-4.66	-4.33
T-47D	-5.67	-5.28	-4.77
MDA-MB-468	-5.62	-5.13	-4.58
_MID	-5.63	-5.0	-4.47
Delta	2.23	0.94	0.74
Range	3.11	1.77	1.21

GI50

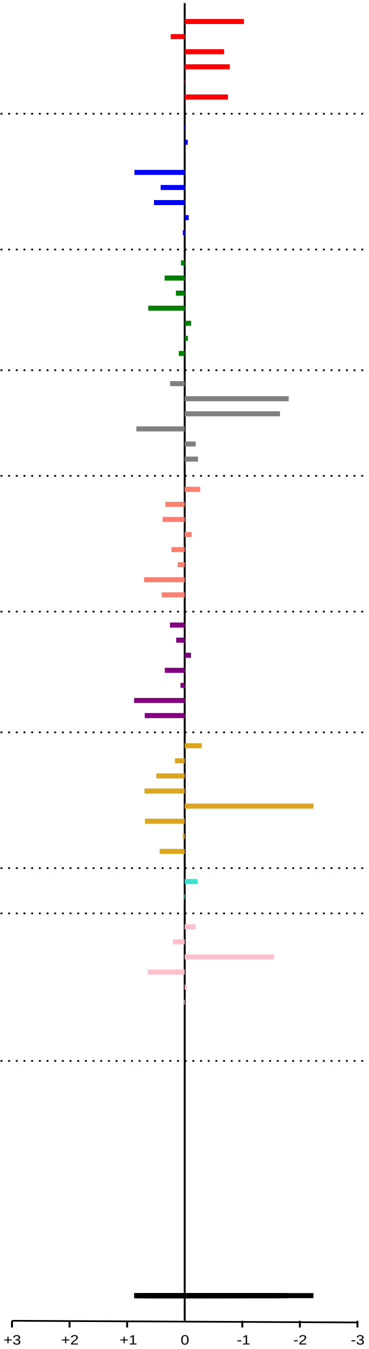

TGI

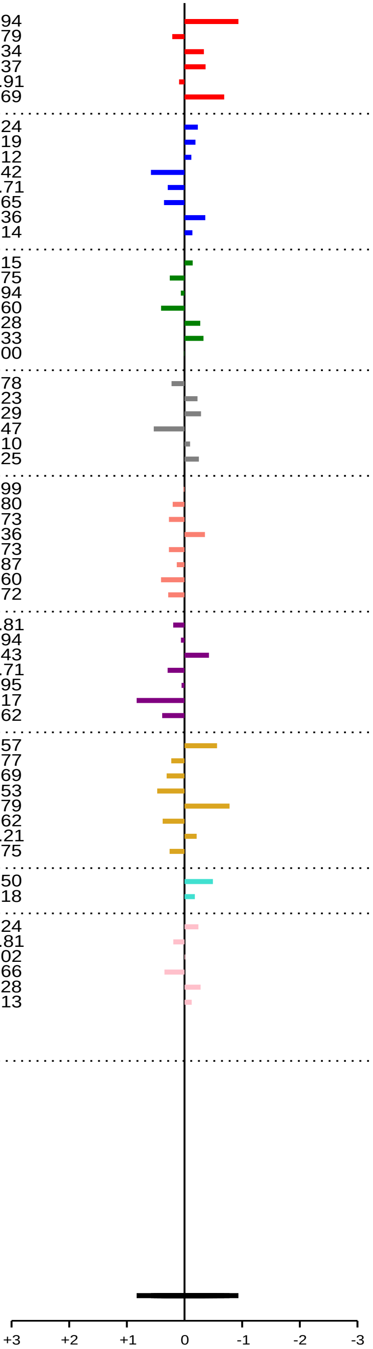

LC50

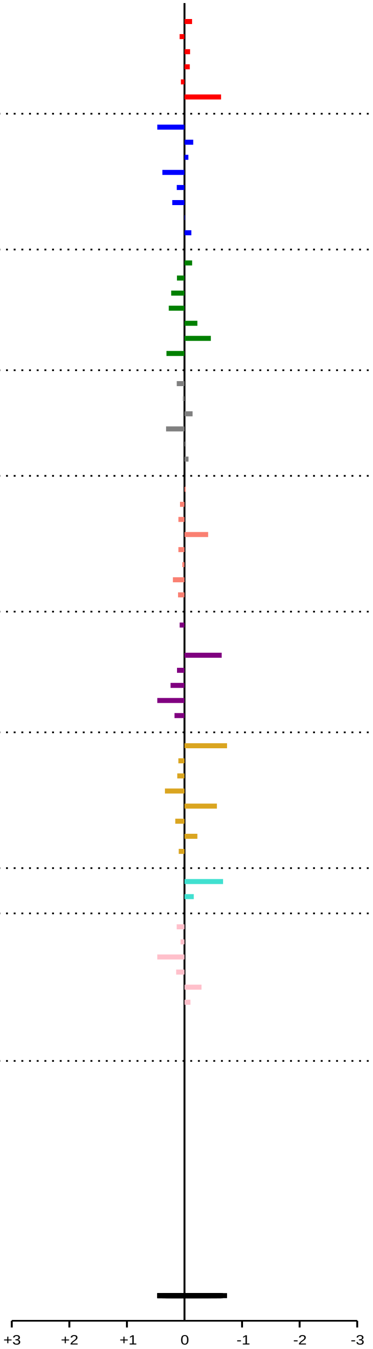

National Cancer Institute Developmental Therapeutics Program Dose Response Curves	NSC:766755/2	SSPL:0YQS	EXP. ID:1302NS28
	Report Date:July 13, 2015	Test Date:February 25, 2013	

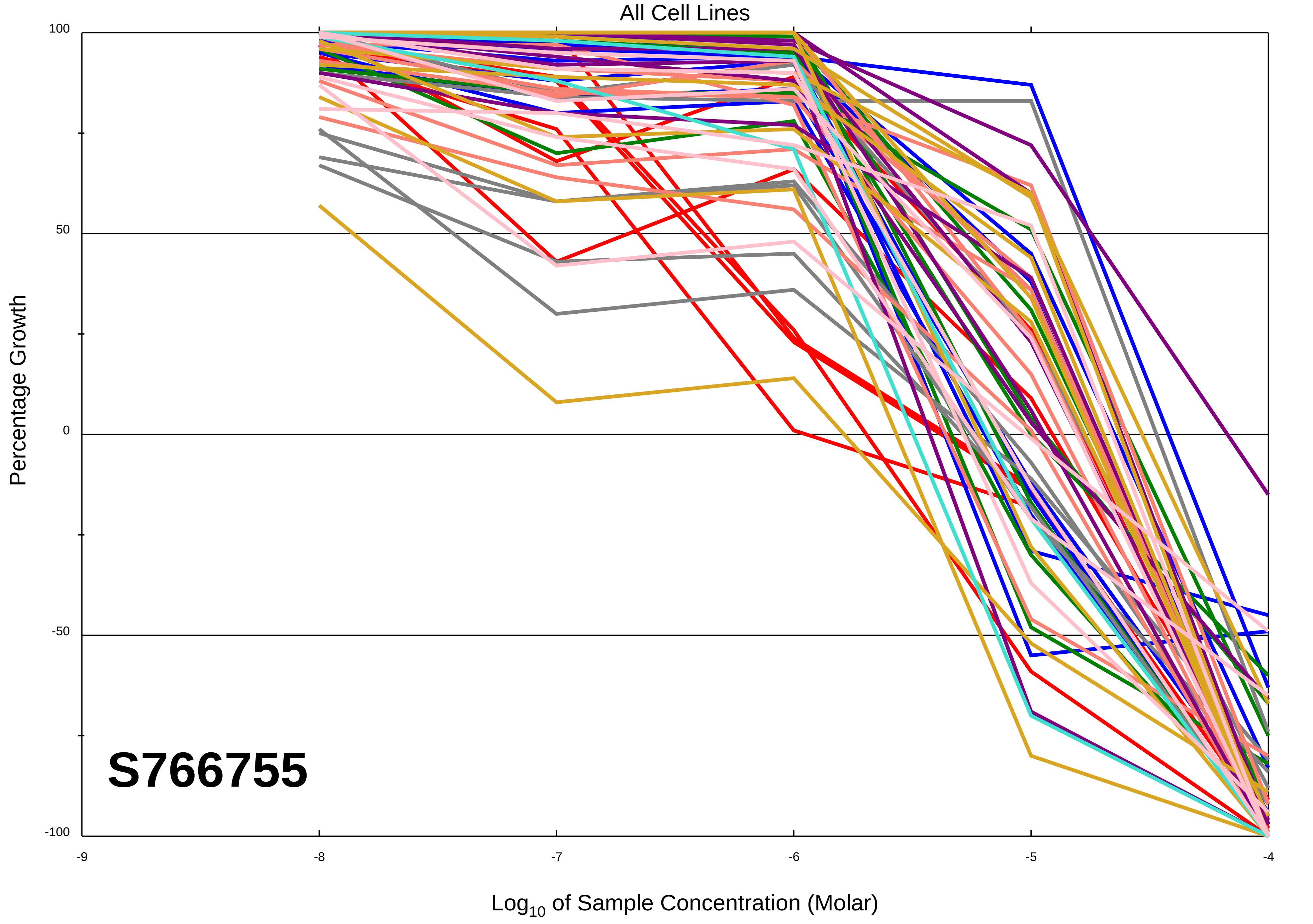

National Cancer Institute Developmental Therapeutics Program<br>Dose Response Curves	NSC: 766755 / 2	SSPL: 0YQS	EXP. ID: 1306RS02
	Report Date: July 13, 2015	Test Date: June 03, 2013	

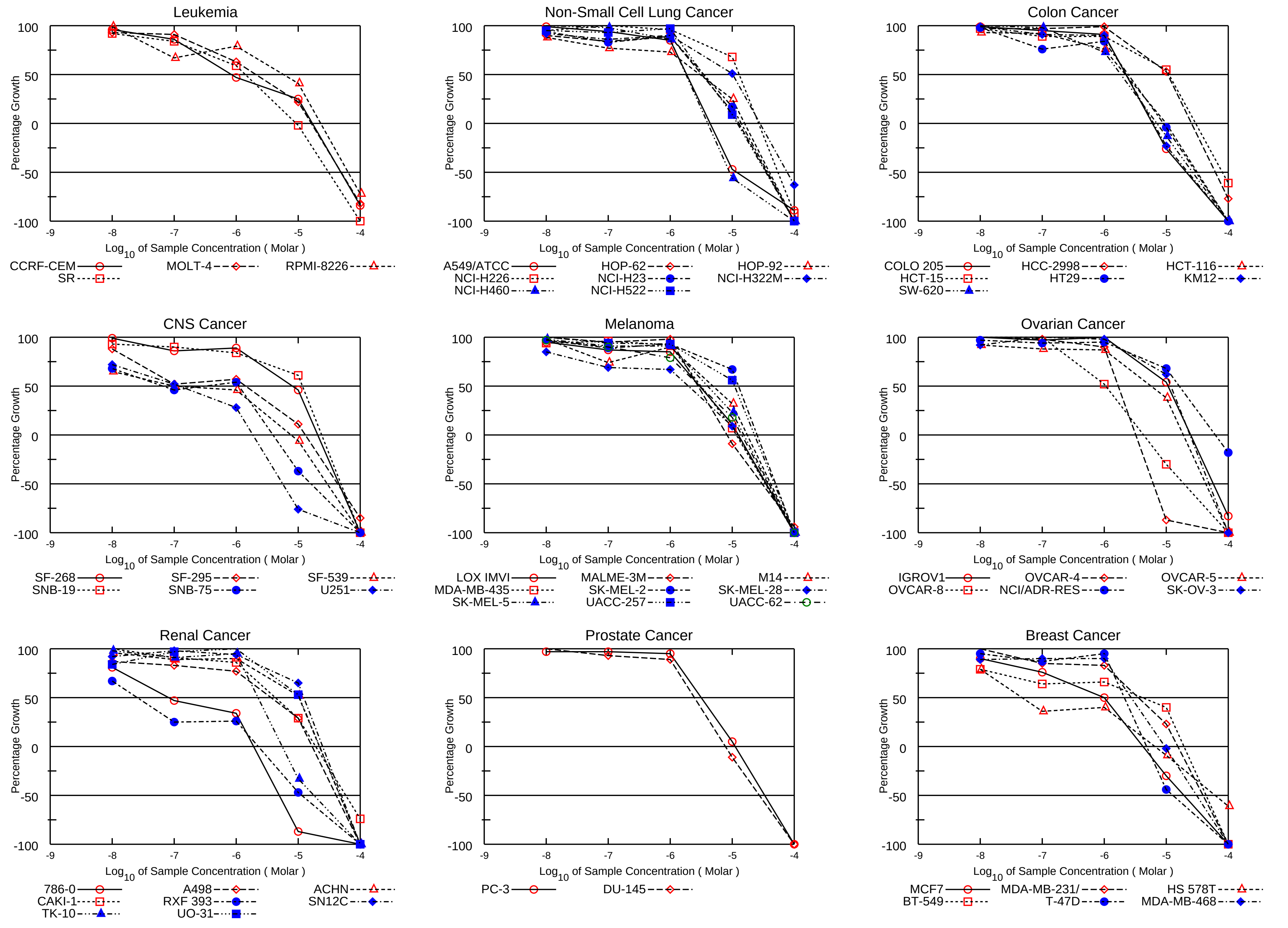

National Cancer Institute Developmental Therapeutics Program Mean Graphs	NSC :766755/2	Units :Molar	SSPL :0YQS	EXP. ID :1306RS02
	Report Date :July 13, 2015		Test Date :June 03, 2013	

Panel/Cell Line	Log_{10}GI50	Log_{10}TGI	Log_{10}LC50
**Leukemia**			
CCRF-CEM	-6.07	-4.77	-4.31
MOLT-4	-5.68	-4.79	-4.31
RPMI-8226	-5.24	-4.64	-4.20
SR	-5.85	-5.03	-4.51
**Non-Small Cell Lung Cancer**			
A549/ATCC	-5.73	-5.35	-4.92
HOP-62	-5.49	-4.89	-4.45
HOP-92	-5.51	-4.80	-4.40
NCI-H226	-4.89	-4.57	-4.26
NCI-H23	-5.46	-4.86	-4.43
NCI-H322M	-4.99	-4.55	-4.11
NCI-H460	-5.75	-5.39	-5.04
NCI-H522	-5.46	-4.92	-4.46
**Colon Cancer**			
COLO 205	-5.65	-5.22	-4.68
HCC-2998	-4.97	-4.59	-4.21
HCT-116	-5.66	-5.00	-4.50
HCT-15	-4.96	-4.53	-4.10
HT29	-5.61	-5.05	-4.52
KM12	-5.65	-5.21	-4.65
SW-620	-5.73	-5.15	-4.57
**CNS Cancer**			
SF-268	-5.10	-4.69	-4.34
SF-295	-5.84	-4.89	-4.36
SF-539	-7.02	-5.12	-4.53
SNB-19	-4.93	-4.62	-4.31
SNB-75		-5.41	-4.79
U251	-6.91	-5.73	-5.25
**Melanoma**			
LOX IMVI	-5.53	-4.90	-4.45
MALME-3M	-5.55	-5.09	-4.52
M14	-5.31	-4.76	-4.38
MDA-MB-435	-5.51	-4.93	-4.47
SK-MEL-2	-4.90	-4.60	-4.30
SK-MEL-28	-5.71	-4.92	-4.46
SK-MEL-5	-5.40	-4.81	-4.41
UACC-257	-4.96	-4.64	-4.32
UACC-62	-5.52	-4.85	-4.42
**Ovarian Cancer**			
IGROV1	-4.97	-4.61	-4.24
OVCAR-4	-5.77	-5.49	-5.21
OVCAR-5	-5.24	-4.72	-4.36
OVCAR-8	-5.97	-5.37	-4.72
NCI/ADR-RES	-4.79	-4.21	> -4.00
SK-OV-3	-4.93	-4.62	-4.31
**Renal Cancer**			
786-0	-7.10	-5.72	-5.31
A498	-5.44	-4.78	-4.39
ACHN	-4.99	-4.66	-4.33
CAKI-1	-5.37	-4.72	-4.24
RXF 393	-7.60	-5.64	-4.95
SN12C	-4.91	-4.61	-4.30
TK-10	-5.65	-5.26	-4.75
UO-31	-4.98	-4.65	-4.33
**Prostate Cancer**			
PC-3	-5.50	-4.96	-4.48
DU-145	-5.61	-5.11	-4.56
**Breast Cancer**			
MCF7	-6.01	-5.37	-4.71
MDA-MB-231/ATCC	-5.45	-4.81	-4.41
HS 578T	-7.33	-5.19	-4.21
BT-549	-5.38	-4.71	-4.36
T-47D	-5.68	-5.32	-4.90
MDA-MB-468	-5.56	-5.02	-4.51
_MID	-5.58	-4.94	-4.49
Delta	2.02	0.79	0.82
Range	2.81	1.52	1.31

GI50

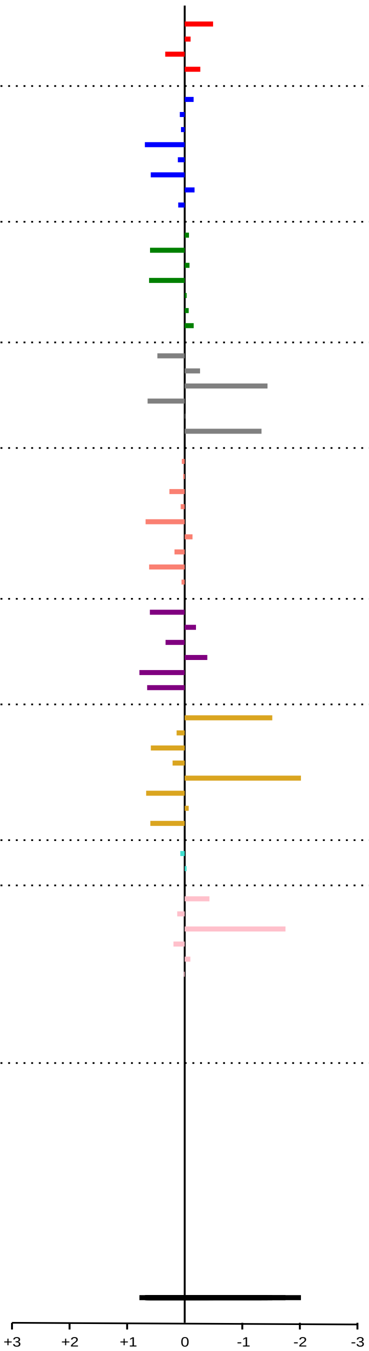

TGI

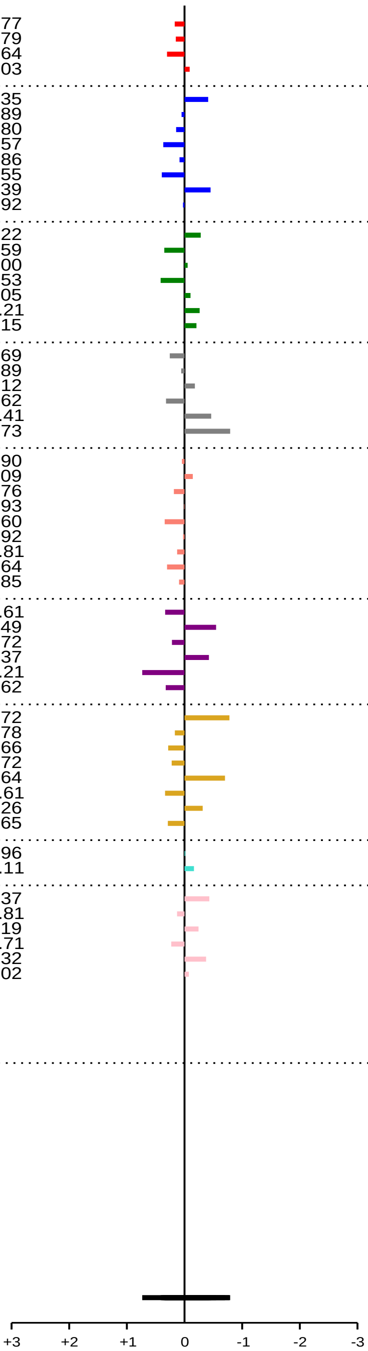

LC50

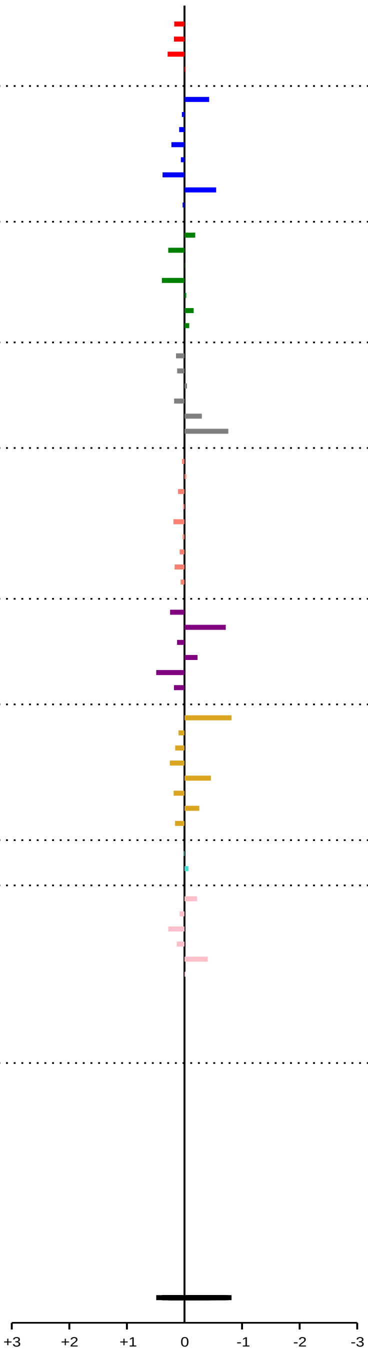

National Cancer Institute Developmental Therapeutics Program Dose Response Curves	NSC:766755/2	SSPL:0YQS	EXP. ID:1306RS02
	Report Date:July 13, 2015	Test Date:June 03, 2013	

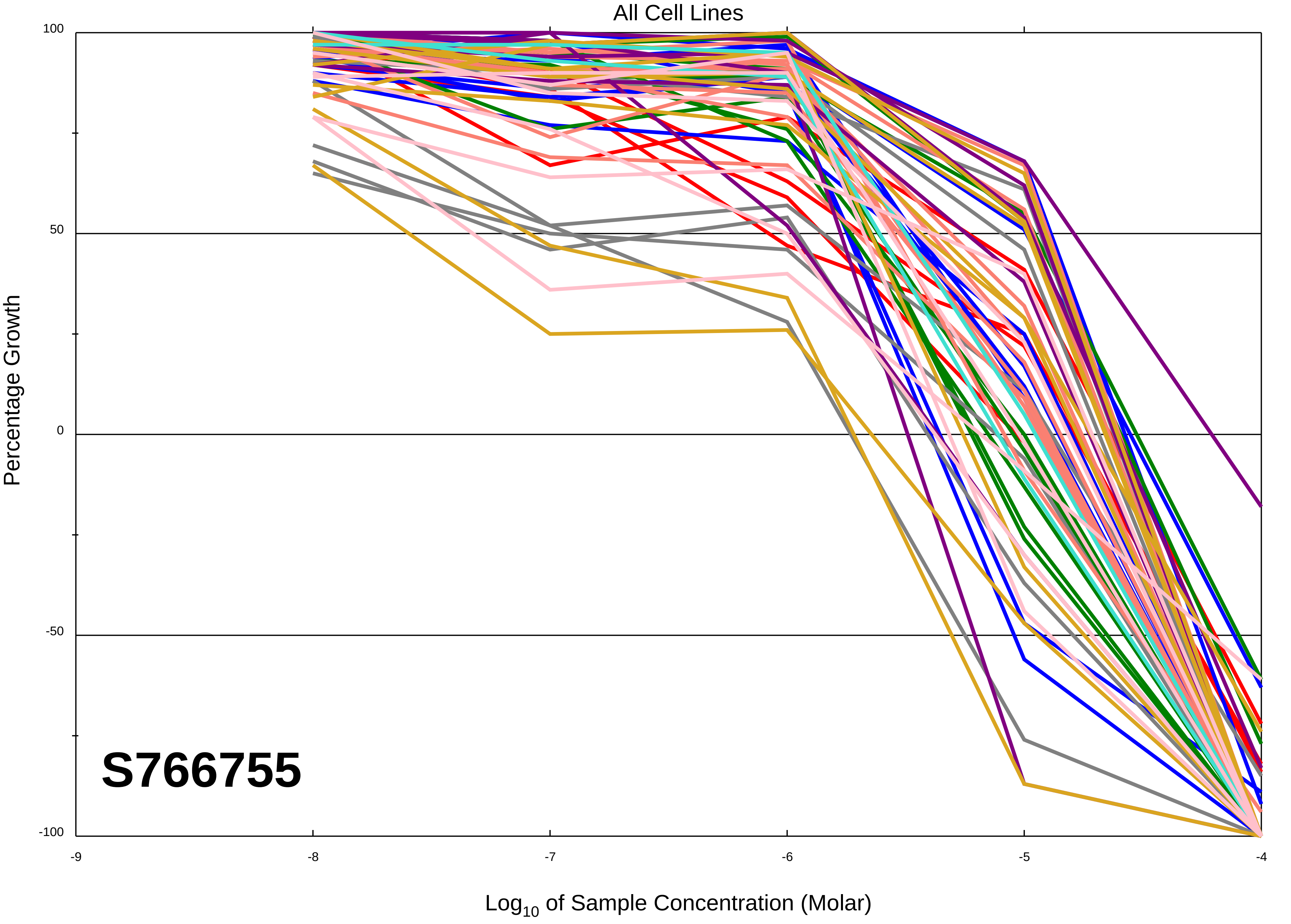

Supplement: S1 Text — (PDF) [file ppat.1005763.s006.pdf]
